# Silica gel and microwave-promoted synthesis of dihydropyrrolizines and tetrahydroindolizines from enaminones

**DOI:** 10.3762/bjoc.17.170

**Published:** 2021-10-13

**Authors:** Robin Klintworth, Garreth L Morgans, Stefania M Scalzullo, Charles B de Koning, Willem A L van Otterlo, Joseph P Michael

**Affiliations:** 1 Molecular Sciences Institute, School of Chemistry, University of the Witwatersrand, PO Wits 2050, Johannesburg, South Africa; 2 Department of Chemistry and Polymer Science, Stellenbosch University, Matieland 7602, Stellenbosch, South Africa

**Keywords:** dihydropyrrolizines, enaminones, microwaves, silica gel, tetrahydroindolizines

## Abstract

A wide range of *N*-(ethoxycarbonylmethyl)enaminones, prepared by the Eschenmoser sulfide contraction between *N*-(ethoxycarbonylmethyl)pyrrolidine-2-thione and various bromomethyl aryl and heteroaryl ketones, underwent cyclization in the presence of silica gel to give ethyl 6-(hetero)aryl-2,3-dihydro-1*H*-pyrrolizine-5-carboxylates within minutes upon microwave heating in xylene at 150 °C. Instead of functioning as a nucleophile, the enaminone acted as an electrophile at its carbonyl group during the cyclization. Yields of the bicyclic products were generally above 75%. The analogous microwave-assisted reaction to produce ethyl 2-aryl-5,6,7,8-tetrahydroindolizine-3-carboxylates from (*E*)-ethyl 2-[2-(2-oxo-2-arylethylidene)piperidin-1-yl]acetates failed in nonpolar solvents, but occurred in ethanol at lower temperature and microwave power, although requiring much longer time. A possible mechanism for the cyclization is presented, and further functionalization of the newly created pyrrole ring in the dihydropyrrolizine core is described.

## Introduction

**Figure 1:**
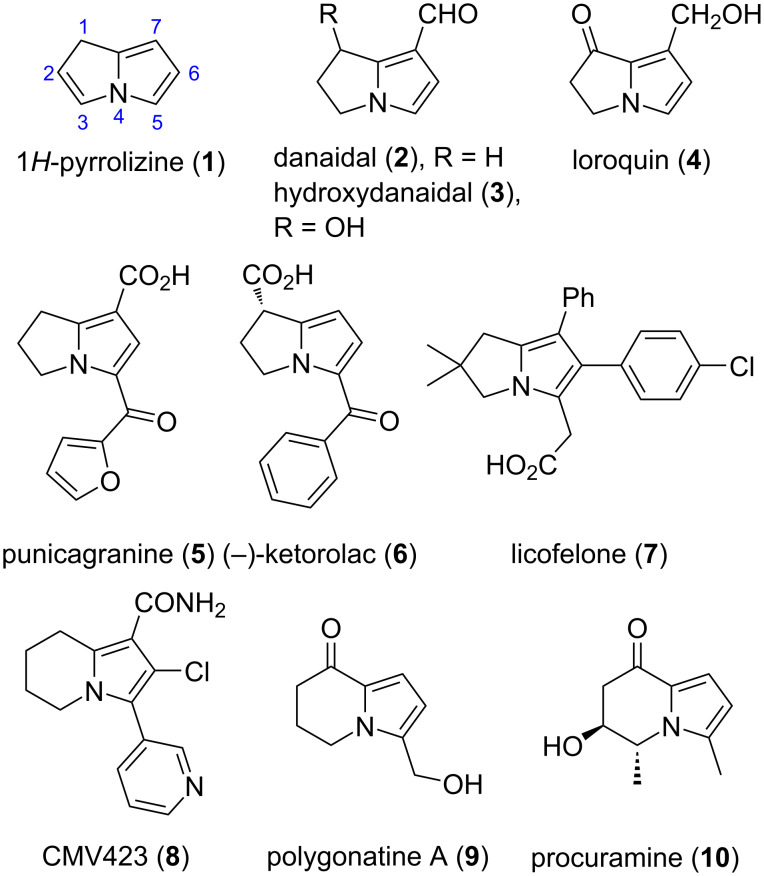
Examples of 2,3-dihydro-1*H*-pyrrolizines (**1**–**7**) and 5,6,7,8-tetrahydroindolizines (**8**–**10**).

Partially or fully saturated variants of the pyrrolizine nucleus **1** are widely occurring motifs in natural products. The hexahydropyrrolizine (pyrrolizidine) system, for example, forms the nucleus of literally hundreds of alkaloids, many of which have gained notoriety owing to their hepatotoxicity and neurological effects on livestock and humans [1–2]. The less commonly encountered 2,3-dihydro-1*H*-pyrrolizine framework occurs in the butterfly metabolites danaidal (**2**) and hydroxydanaidal (**3**) [3], as well as in plant alkaloids such as loroquin (**4**) [4] and the recently characterized pomegranate alkaloid punicagranine (**5**) (Figure 1) [5]. The 2,3-dihydro-1*H*-pyrrolizine motif is also a structural component of potential drugs with a variety of pharmacological effects [6], including anticancer activity [7]. Relevant examples include (−)-ketorolac (**6**) [8], which has reached the market as a nonsteroidal anti-inflammatory drug [6], and licofelone (**7**) [9–10], a promising co-drug for anticancer combination chemotherapy [7]. Related 5,6,7,8-tetrahydroindolizines such as CMV423 (**8**) [11], a lead compound for treating infections by human cytomegalovirus, and the natural products polygonatine A (**9**) [12] and procuramine (**10**) [13], have also attracted attention recently.

**Scheme 1:**
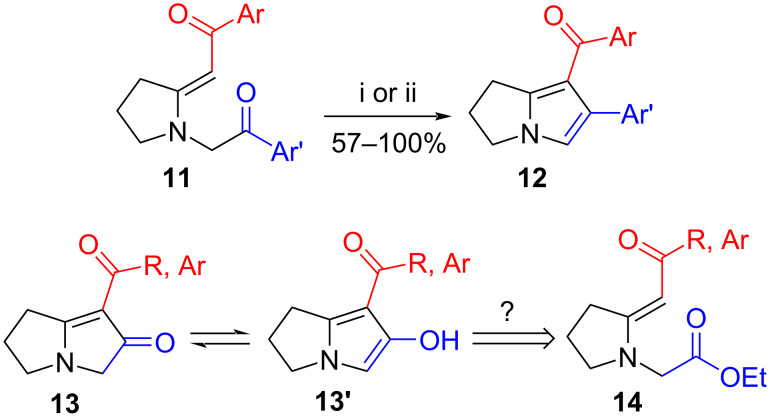
Previous [18] and proposed routes to 2,3-dihydro-1*H*-pyrrolizines from enaminones. Reagents and conditions: (i) AcOH–MeOH, rt, 18–24 h; (ii) SiO_2_, 90 °C, 1–2 h.

Our continuing efforts to exploit enaminones as building blocks for the synthesis of alkaloids and other nitrogen-containing heterocycles have largely concentrated on targets containing indolizidine and quinolizidine backbones [14]. The pyrrolizidine motif has up to now eluded us other than when it forms part of the tricyclic 2,3-dihydro-1*H*-pyrrolo[1,2-*a*]indole system [15], as in our route to aziridinomitosenes [16–17]. In the course of our attempts at the syntheses of aryl-bearing indolizidine alkaloids via *N*-phenacyl vinylogous amides such as **11**, however, we fortuitously found substituted 2,3-dihydro-1*H*-pyrrolizines **12** as unexpected products when intermediates **11** were exposed to acidic conditions, including treatment with acetic acid or even during chromatography on silica gel (Scheme 1) [18]. In these cyclizations the enaminone acts as an intramolecular nucleophile towards the phenacyl substituent, even though the nucleophilic character of the enamine component is expected to be suppressed by the “push–pull” effect arising from the electron-withdrawing carbonyl group [19–23]. The dual properties of enaminones as both nucleophiles and electrophiles have frequently been exploited in the synthesis of heterocyclic products, including pyrroles and related systems [22–28]. Nonetheless, encouraged by the ease of access to pyrrole-containing products of type **12** and their potential synthetic utility, we surmised that replacing the aroyl component of the *N*-phenacyl substituent by electrophilic groups such as esters, amides or nitriles might yield 2,3-dihydro-1*H*-pyrrolizin-6-ones **13** or their hydroxypyrrole tautomers **13’**. Our findings with *N*-(ethoxycarbonylmethyl)enaminones **14** are described in this article.

## Results and Discussion

**Scheme 2:**
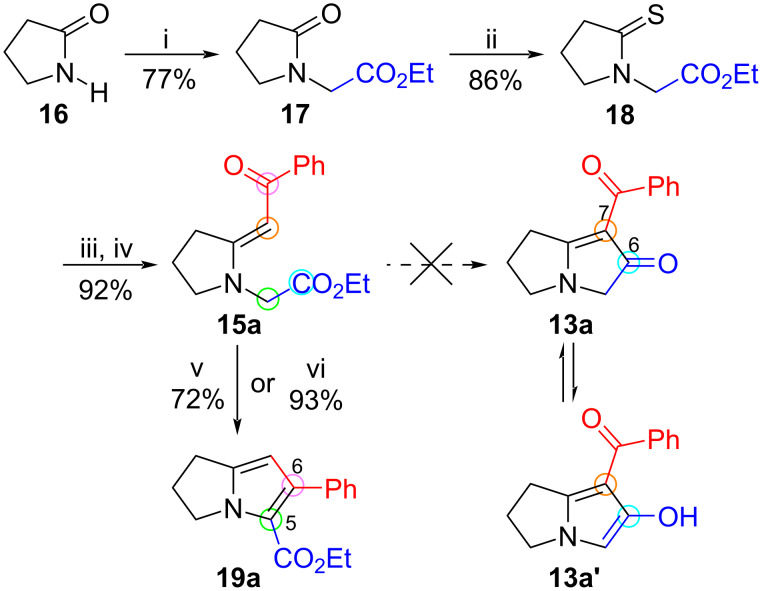
Synthesis of pyrrolizine **19a** from lactam **16** via enaminone **15a**. Reagents and conditions: (i) NaH, THF, rt, 2.3 h, then BrCH_2_CO_2_Et, rt, 16 h; (ii) Lawesson’s reagent, toluene, 80 °C, 18 h; (iii) BrCH_2_COPh, MeCN, rt, overnight; (iv) P(OEt)_3_, NEt_3_, MeCN, rt, 18 h; (v) AcOH, microwaves (150 W, 140 °C), 10 min; (vi) SiO_2_, xylene, microwaves (150 W, 150 °C), 3.5 min.

The benzoyl-containing enaminone **15a** (Ar = Ph) was selected as a model for investigating conditions for the cyclization. This compound was conveniently prepared by Eschenmoser sulfide contraction [29–30], as shown in Scheme 2. The route entailed initial alkylation of the anion of pyrrolidin-2-one (**16**) with ethyl bromoacetate to afford the known compound ethyl 2-(2-oxopyrrolidin-1-yl)acetate (**17**) [31–33] in 77% yield. Thionation of **17** with Lawesson’s reagent in toluene at 80 °C gave the thione **18** as a yellow oil (86%). Reaction of **18** with phenacyl bromide in acetonitrile at ambient temperature to produce the putative S-alkylated salt was complete within 18 hours, after which treatment with triethyl phosphite and triethylamine rapidly completed the sulfide contraction, giving (*E*)-enaminone **15a** in 92% yield after chromatographic purification. The geometry was inferred from its NOESY spectrum, which showed a weak but distinct correlation between the vinyl proton singlet (δ 5.66) and the methylene group flanked by nitrogen and the ester (δ 4.06). The *E*-geometry was also suggested by the chemical shift of the hydrogen atoms at C-3 of the pyrrolidine ring (δ 3.43), which showed anisotropic deshielding by the carbonyl group. In similar pyrrolidine-containing (*Z*)-enaminones these hydrogen atoms appear approximately 0.5–0.7 ppm upfield (ca. δ 2.5–2.7) compared to the chemical shifts of related (*E*)-enaminones [29,34].

In contrast to the corresponding *N*-phenacyl analogue **11** (Ar = Ar’ = Ph), the ester-containing enaminone **15a** proved to be completely stable during chromatography on silica gel or upon dissolution in acetic acid at room temperature (conditions under which we had observed cyclization of vinylogous amides **11** [18]), and spontaneous cyclization to the expected pyrrolizinone **13a** or tautomer **13a'** did not take place (Table 1, entry 1). More vigorous conditions were therefore investigated. Heating **15a** in acetic acid (150 W) for 10 minutes at 140 °C produced a new product in 72% yield (Table 1, entry 2). However, the isolated product was not the expected pyrrolizinone **13a/13a'**, but the 2,3-dihydro-1*H*-pyrrolizine ester **19a**. The expected signals for the ethyl ester were apparent in the ^1^H NMR spectrum, the lone pyrrole hydrogen appeared as a singlet at δ 5.95, and the ester carbonyl group occurred at δ 161.4 in the ^13^C NMR spectrum. In other words, the enaminone component did not act as a nucleophile towards the ester, as it did with the *N*-phenacylenaminones **11**. Instead, it functioned as an electrophile at its carbonyl group by condensation with the methylene unit α to the ester, thereby forming the C5–C6 bond of the azabicyclic system instead of the C6–C7 bond.

**Table 1:** Optimization of the cyclization of enaminone **15a** to pyrrolizine **19a**.

Entry	Solvent	Additive	Temp.^a^	Time	Yield of **19a** (%)

1	CH_3_CO_2_H	–	rt	18 h	–^b^
2	CH_3_CO_2_H	–	140 °C	10 min	72
3	EtOH	–	100 °C	10 min	–^b^
4	xylene	–	150 °C	10 min	–^b^
5	DMF	–	150 °C	10 min	–^b^
6	conc. HCl	–	rt	10 min	–^c^
7	conc. HCl	–	rt	18 h	–^d^
8	conc. HCl	–	50 °C	10 min	–^d^
9	CF_3_CO_2_H	–	rt	18 h	–^d^
10	MeSO_3_H	–	rt	10 min	–^c^
11	MeSO_3_H	–	rt	18 h	–^d^
12	MeSO_3_H	–	50 °C	10 min	–^d^
13	BF_3_·OEt_2_	–	rt	18 h	–^d^
14	EtOH	HCl (cat.)	rt	18 h	–^b^
15	EtOH	HCl (cat.)	100 °C	10 min	–^d^
16	CH_2_Cl_2_	AlCl_3_	rt	18 h	–^b^
17	CH_2_Cl_2_	AlCl_3_	50 °C	10 min	–^d^
18	CH_2_Cl_2_	BF_3_·OEt_2_	rt	18 h	–^b^
19	CH_2_Cl_2_	BF_3_·OEt_2_	50 °C	10 min	–^d^
20	CH_2_Cl_2_	Amberlyst 15	rt	24 h	–^c^
21	CH_2_Cl_2_	Amberlyst 15	50 °C	10 min	–^c^
22	xylene	Montmorillonite K10	rt	18 h	–^d^
23	xylene	Montmorillonite K10	150 °C	10 min	84
24	xylene	silica gel	rt	18 h	–^b^
25	toluene	silica gel	140 °C	8 min	90
26	xylene	silica gel	150 °C	3.5 min	93

^a^Microwave heating (150 W) in a capped tube. ^b^No reaction and no apparent decomposition. ^c^**15a** was protonated, but regenerated on treatment with aq NaHCO_3_ (TLC). ^d^Decomposition was observed.

A brief survey of alternative conditions for the cyclization revealed that the acidic conditions under which pyrrolizine formation took place were essential, although the nature of the acid proved to be critical (Table 1). Starting material was recovered unchanged when **15a** was heated in capped tubes under microwave conditions [35–37] for 10 minutes in solvents such as ethanol (100 °C), xylene (150 °C) or *N*,*N*-dimethylformamide (150 °C) (Table 1, entries 3–5). Dissolution in neat protic or Lewis acids (e.g., hydrochloric acid, trifluoroacetic acid, methanesulfonic acid, boron trifluoride etherate) resulted in decomposition if the reactions were left at room temperature overnight, or within ten minutes if the temperature was raised, even to 50 °C (Table 1, entries 6–13). The enaminone appeared to be protonated at room temperature, presumably on the oxygen site in accordance with well-established precedents [38–39]; and after ten minutes it could be recovered after neutralization with aqueous sodium hydrogen carbonate (Table 1, entries 6, 10). No apparent reaction occurred with dilute hydrochloric acid in ethanol (Table 1, entry 14) unless the solution was heated, in which case decomposition took place (Table 1, entry 15). Solutions of **15a** in dichloromethane also decomposed rapidly when treated with aluminum trichloride or boron trifluoride at 50 °C, although no reaction was apparent at room temperature even after 18 hours (Table 1, entries 16–19). When a solution of **15a** in dichloromethane was treated with Amberlyst 15, an ion exchange resin with a strongly acidic sulfonic acid [40], it was rapidly adsorbed and presumably protonated, but it could be displaced from the resin upon treatment with aqueous sodium hydrogen carbonate (Table 1, entries 20 and 21). More interestingly, microwave heating at 150 °C for no more than 10 minutes of a solution of **15a** in xylene with montmorillonite K10, an acidic layered aluminosilicate clay often used as a catalyst in microwave-assisted organic synthesis [41–44], also afforded the pyrrolizine **19a** in a good yield of 84%, although prolonged exposure even at ambient temperature led to decomposition (Table 1, entries 22 and 23). However, the most successful microwave-assisted reactions took place with the somewhat less acidic silica gel (200–400 mesh) as the promotor [45]. Mixing a solution of the enaminone in toluene or xylene with silica gel prior to microwave heating at 140–150 °C with moderate stirring for just a few minutes rapidly produced the product **19a** which, after evaporation of the solvent, was cleanly eluted from the adsorbent by chromatography on silica gel with ethyl acetate–hexane mixtures. Yields in toluene or xylene were typically around 90% (Table 1, entries 25 and 26). Although most reported microwave-promoted organic reactions on solid supports are performed without additional solvent [46–47], the combination of a nonpolar solvent with a polar heterogeneous catalyst can be effective as a consequence of the solid selectively absorbing the microwave energy and facilitating reaction at its surface, while the nonpolar solvent absorbs relatively little microwave energy and remains at a milder temperature.

**Scheme 3:**
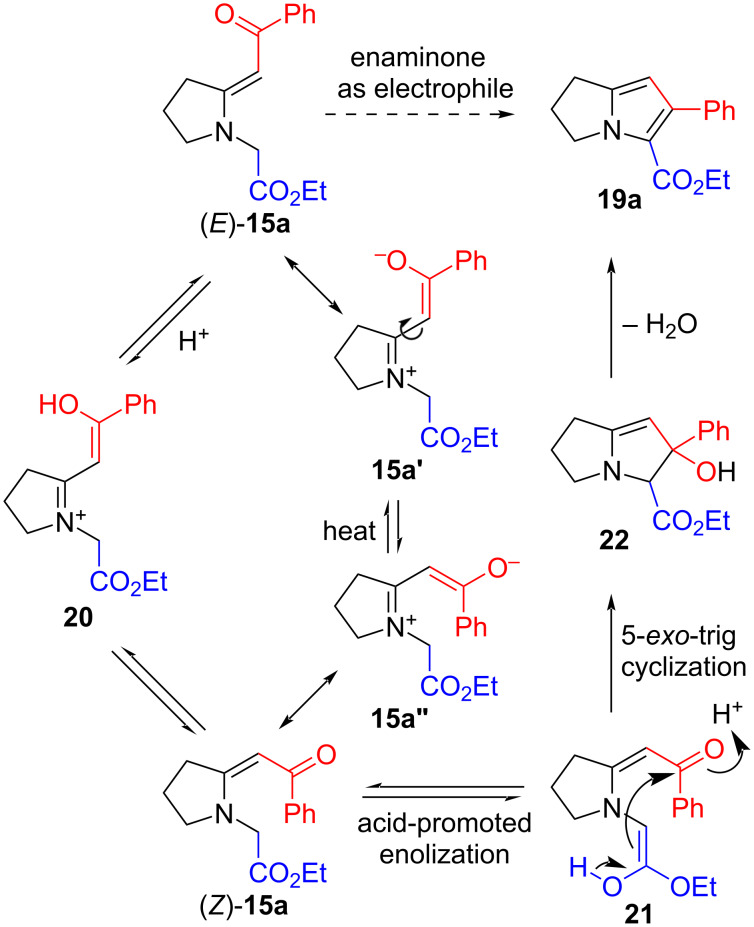
Proposed mechanism for the formation of pyrrolizidine **19a** from enaminone (*E*)-**15a**.

The weakly acidic conditions appear to serve several functions. Firstly, the *E*-geometry of reactant **15a** is obviously incorrect for the cyclization, which requires the nucleophilic methylene adjacent to the ester to approach close enough to the electrophilic carbonyl group of the enaminone for the intramolecular condensation to occur. It is possible that acid-induced isomerization to the *Z*-isomer (*Z*)-**15a**, which is required for cyclization, proceeds through the protonated intermediate **20**, in which the weakened double bond permits configurational equilibration between the geometric isomers [20] (Scheme 3). Alternatively, a purely thermal *E*-to-*Z* isomerization of the enaminone prior to acid-promoted cyclization cannot be ruled out, since conjugation in the push–pull system should weaken the C=C bond and lower its rotational barrier [20]. Similar enaminone isomerizations have been detected even at room temperature [48]. If thermal isomerization indeed takes place, then one could envisage rotation about the C–C single bond in the zwitterionic mesomer of (*E*)-**15a** (i.e., **15a'**) leading to the rotamer **15a"**, and hence (*Z*)-**15a**. It must be noted, however, that we never managed to obtain direct evidence, whether by TLC or NMR spectroscopy, for the short-lived intermediate (*Z*)-**15a**. Nonetheless, use of the weaker acids seems to be crucial, since with strong acids the protonation to **20** appears not to be reversible, and direct cyclization of **20** to **19a** cannot be occurring (cf. Table 1, entries 6–21). Secondly, the acid is also likely to facilitate enolization of the ester such that 5-*exo*-trig interactions of both the enol nucleophile and the ketone in the transient intermediate **21** bring about formation of the new five-membered ring intermediate **22**. Finally, acid-induced dehydration of **22** completes the formation of the pyrrole ring, resulting in conversion into the dihydropyrrolizine **19a**. This enaminone-based method for constructing a pyrrole ring is similar to that in our reported routes to lamellarin alkaloids, in which N-alkylation of (*Z*)-configured 3,4-dihydroisoquinoline-derived enaminones with ethyl bromoacetate under either conventional [49–50] or microwave [51] heating conditions yielded pyrrolo[2,1-*a*]isoquinoline products. A somewhat comparable cyclization of *N*-benzylenaminones has been reported under superbasic conditions [52], while base-induced pyrrole formation from *N*-(ethoxycarbonylmethyl)enamino esters (vinylogous urethanes) and the corresponding nitriles (vinylogous cyanamides) has also been described [53]. A similar base-induced cyclization of *N*-(alkoxycarbonylmethyl)-7-formylindoles to give pyrrolo[3,2,1-*hi*]indoles is also of interest [54]. Acid-induced cyclization akin to ours, and also assuming an in situ *E* to *Z* isomerization under the reaction conditions, was reported as recently as 2018 [55]. More interestingly, conventional heating in acetic acid of an enaminone bearing a phenacylsulfanyl substituent adjacent to nitrogen on the C=C bond produced ethyl pyrrolo[2,1-*b*]thiazole-5-carboxylates in moderate yield, while the corresponding microwave-assisted reaction in the same solvent gave high yields of pyrrolo[2,1-*b*]thiazol-6-ones [56]. The former results from reaction between the α-methylene position of the ester and the electrophilic carbonyl component of the enaminone, while the latter arises from nucleophilic attack of the enaminone at the ester’s carbonyl group. This appears to be a rare example of a change in reaction conditions swinging the outcome from one mode of enaminone reactivity to the other.

Following this optimized method, we undertook the synthesis of a range of enaminones **15** bearing a variety of aroyl and heteroaroyl substituents as well as *tert*-butyl as precursors for the silica gel-mediated cyclization reaction to form pyrrolizine products. The bromomethyl ketones required for the initial sulfide contraction were either purchased from commercial suppliers or prepared from the corresponding methyl ketones by reported procedures [57–65]. Results are summarized in Table 2. Triphenylphosphine and triethyl phosphite could be used interchangeably in the sulfur extrusion step. However, in most cases the co-elution of enaminones **15** with phosphorus-derived byproducts during chromatographic purification was unavoidable, and multiple chromatographic separations reduced the isolated yields. Such contamination with phosphorus-containing byproducts is a well-known problem in Eschenmoser condensations [30]. With particularly difficult separations we simply subjected the impure intermediates (for most of which characteristic ^1^H or ^13^C NMR spectroscopic signals could be observed, and suitable HRMS values could be measured; see Supporting Information File 1) to the optimized cyclization reaction conditions, since the contaminants did not inhibit pyrrolizine formation. Chromatographic purification of products **19** thereafter proved to be easier as they are less polar than the enaminones, and could generally be separated from other byproducts. In these cases, overall isolated yields of the dihydropyrrolizines **19** are reported over the two steps.

**Table 2:** Preparation of enaminones **15a–y**^a^, and cyclization to dihydropyrrolizines **19a–y**^b^.

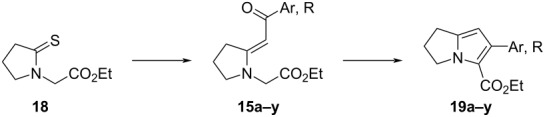					

Entry	Compound **15**/**19**	Ar or R	Yield of **15** (%)	Cyclization time (min)	Yield of **19** (%)

1	**a**	C_6_H_5_	92^c^	3.5	93
2	**b**	4-NO_2_-C_6_H_4_	99^c^	0.5	78
3	**c**	4-CN-C_6_H_4_	92^c^	3	92
4	**d**	4-F-C_6_H_4_	90^d^	5	98
5	**e**	4-Br-C_6_H_4_	93^c^	4.5	98
6	**f**	4-Me-C_6_H_4_	82^c^	4.5	89
7	**g**	4-MeO-C_6_H_4_	84^c^	5	96
8	**h**	3-NO_2_-C_6_H_4_	99^c^	3.5	77
9	**i**	3-MeO-C_6_H_4_	^d, e^	3.5	81^f^
10	**j**	3,4-(MeO)_2_-C_6_H_3_	^d, e^	5	89^f^
11	**k**	2-NO_2_-C_6_H_4_	87^c^	0.5	0^g^
12	**l**	2-I-C_6_H_4_	93^c^	10	0^g^
13	**m**	2-Br-C_6_H_4_	^c, e^	9	46^f, g^
14	**n**	2-Br-4,5-(MeO)_2_-C_6_H_2_	93^c^	9	35^g^
15	**o**	2-Cl-C_6_H_4_	92^c^	9	92
16	**p**	2-MeO-C_6_H_4_	^c, e^	9	82^f^
17	**q**	2,5-(MeO)_2_-C_6_H_3_	^d, e^	7	82^f^
18	**r**	2-F-C_6_H_4_	^d, e^	1.5	99^f^
19	**s**	naphthalen-1-yl	^d, e^	17	81^f^
20	**t**	styryl	88^c^	0.5	82
21	**u**	furan-2-yl	93^d^	5.5	100
22	**v**	thien-2-yl	^d, e^	4.5	99^f^
23	**w**	benzofuran-2-yl	93^d^	2.5	96
24	**x**	*N-*tosylindol-3-yl	88^d^	2	92
25	**y**	C(CH_3_)_3_	57^d^	19	81

^a^**18** (1 equiv), bromomethyl ketone (1.2 equiv), MeCN, rt, 18 h; then add PPh_3_ or P(OEt)_3_, NEt_3_, rt. ^b^SiO_2_ (500 wt %), xylene, MW (180 W), internal temperature ca. 150 °C, approximate cyclization time as stated. ^c^With P(OEt)_3_; ^d^With PPh_3_. ^e^Enaminone contaminated with P-containing residues; yield not determined. ^f^Yield calculated over two steps. ^g^Significant decomposition was observed.

The approximate time for completion of the cyclization of enaminones **15** to pyrrolizines **19** was determined by TLC monitoring of the reaction mixture after successive time intervals. A fairly accurate estimate of the reaction time as a function of the changing acyl substituent could thus be obtained to within 30 seconds. As can be seen from Table 2, we observed a distinct rate dependence on both the size and the electronic properties of aroyl substituents. In general, electron-withdrawing substituents produced an increase in reaction rate relative to the unsubstituted parent **15a**, while electron-donating substituents on the *para*-position of the aromatic ring tended to slow down the reaction (Table 2, entries 1–7). This is to be expected as a consequence of the mesomeric effects of the aryl substituents on the electrophilicity of the carbonyl group. Substituents in the *meta*-position, whether electron-withdrawing or electron-donating (Table 2, entries 8 and 9), had a negligible electronic influence on the carbonyl group, and the time in which cyclization was completed was essentially the same as for the unsubstituted phenyl precursor **15a**. For the 3,4-dimethoxyphenyl substrate **15j** containing both *meta*- and *para*-substituents, only the latter affected the rate, and the cyclization time was the same as for the 4-methoxyphenyl system **15g** (Table 2, entry 10 vs 7).

The outcome was more complex with *ortho*-substituents on the aromatic ring, since both steric and electronic effects are likely to operate. When chemically labile groups are present, decomposition of the putative pyrrolizine product appears to be faster than the rate of cyclization of the enaminone. In particular, decomposition occurred during the attempted cyclization of the 2-nitrophenyl- and 2-iodophenylenaminones **15k** and **15l** even before all of the precursor had been consumed. In the 2-nitro case decomposition was noted within 30 seconds, while for the 2-iodo compound complete decomposition took place within 10 minutes (Table 2, entries 11 and 12). Less problematic were the *ortho-*bromo examples (Table 2, entries 13 and 14), although the cyclizations proceeded with significantly reduced yields and decomposition was still evident. 2-Bromophenylenaminone **15m** gave a disappointing yield of 46% for **19m** after a comparatively long reaction time of nine minutes, while the more electron-rich 2-bromo-4,5-dimethoxy analogue **15n** gave an even lower yield of 35% in the same reaction time. However, the 2-chlorophenyl, 2-methoxyphenyl and 2,5-dimethoxyphenylenaminones **15o–q** proved to be well behaved, and gave excellent yields of pyrrolizines **19o–q** (82–92%), although in relatively long times (7–9 minutes). Perhaps most surprising was the speed and efficiency of cyclization of the 2-fluorophenyl enaminone **15r**, which was prepared in situ prior to cyclization. The pyrrolizine **19r** was obtained within 90 seconds of microwave heating, and in 99% yield over the two steps. The rapidity of the condensation probably reflects both the inductive electron-withdrawing effect of fluorine and the minimal steric constraints offered by this small substituent. Other examples that illustrate the likely influence of steric factors are provided by the naphthalen-1-yl- and styrylenaminones **15s** and **15t**, cyclization of which required 17 minutes and 30 seconds, respectively. The bulkiness of the former hinders the approach of the nucleophile to the carbonyl site, while in the latter case insertion of the vinyl unit between the carbonyl and the aryl ring makes the electrophilic site in the enaminone significantly less crowded.

Cyclization of enaminones bearing heteroaromatic substituents proceeded very well (Table 2, entries 21–24). Reaction of the electron-rich furyl- and thiophenyl-bearing precursors **15u** and **15v** was slower than with the phenyl parent **15a**, once again reflecting the rate-retarding effect of the electron-donating rings. However, cyclization of both the benzo-fused heterocyclic systems **15w** and **15x** was surprisingly fast despite their greater steric bulk. The only successful pyrrolizine formation with an alkyl rather than an aryl substituent was with the *tert*-butylenaminone **15y**, which was itself prepared from thiolactam **18** and bromopinacolone in a rather poor yield of 57%. The cyclization afforded pyrrolizine **19y** in 81% yield, albeit in a markedly slow time of 19 minutes. Both the electron-donating nature and the size of this alkyl group probably slow down the reaction. We were unfortunately not successful with other nonaromatic substituents, including trifluoromethyl and ethoxycarbonyl groups, since the initial formation of the requisite enaminones (from **18** and 3-bromo-1,1,1-trifluoropropan-2-one or ethyl bromopyruvate, respectively) failed. Following up on reactions with these and other synthetically intriguing substituents will require a different method for making the necessary enaminones.

**Scheme 4:**
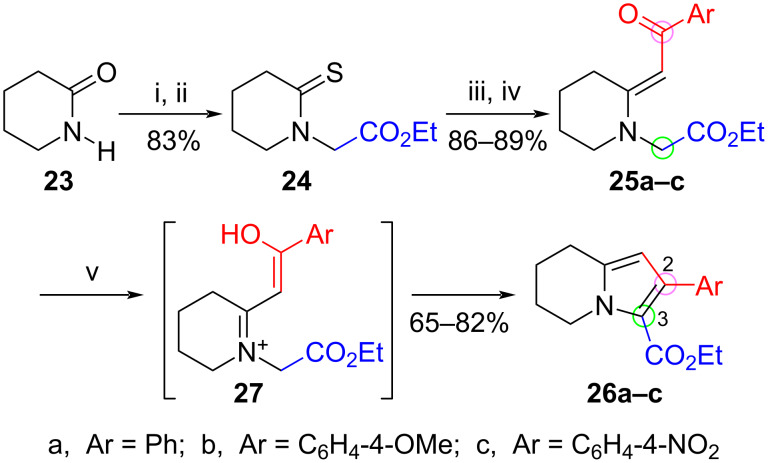
Synthesis of tetrahydroindolizines **26a–c** from lactam **23** via enaminones **25a–c**. Reagents and conditions: (i) NaH, THF, rt, 2.3 h, then BrCH_2_CO_2_Et, 0 °C–rt, 16 h; (ii) Lawesson’s reagent, PhMe, 80 °C, 18 h; (iii) BrCH_2_COAr, MeCN, rt, overnight; (iv) P(OEt)_3_, NEt_3_, MeCN, rt, 18 h; (v) SiO_2_, EtOH, microwaves (50 W, 100 °C), 1–3 h.

Our success with the microwave-assisted synthesis of 2,3-dihydro-1*H*-pyrrolizines from pyrrolidine-based enaminones suggested that the technique might also be suitable for preparing 5,6,7,8-tetrahydroindolizine analogues from the corresponding piperidine systems. In a preliminary investigation we prepared three such enaminones by the route shown in Scheme 4. Alkylation of piperidin-2-one (**23**) with ethyl bromoacetate followed by treatment of the resulting lactam [66–67] with Lawesson’s reagent in toluene at 80 °C gave the thione **24** in 83% overall yield. Reaction of **24** with phenacyl bromide and its 4-methoxy and 4-nitro congeners in acetonitrile, followed by sulfide contraction of the resulting thioiminium ether salts with triethylamine and triethyl phosphite, afforded the (*E*)-enaminones **25a–c** in yields of 86–89%. The *E*-geometry of **25a** was again confirmed by NOESY NMR spectroscopy, which showed an interaction between the vinyl hydrogen (δ 5.55) and the methylene unit adjacent to the ester (δ 3.96). The through-space anisotropic deshielding of C-3 in the ring (δ 3.32) by the carbonyl group also supported the assignment of the geometry.

Microwave heating of intermediate **25a** with silica gel in xylene under the cyclization conditions optimized for the formation of dihydropyrrolizines indeed produced the tetrahydroindolizine **26a**, but in an extremely disappointing yield of less than 30%. Significant decomposition was noted even when the temperature was reduced. From some exploratory TLC studies it appeared that, even in acetic acid at room temperature, protonation of **25a** was virtually instantaneous and irreversible, as evinced by the formation of a baseline spot for the acetate salt. Since the analogous five-membered enaminones **15** form an obvious baseline salt spot only with strong protic acids but not with acetic acid, it appears that protonation of the pyrrolidine-based enaminones with this weak acid must be readily reversible. Thus the critical isomerization equilibrium postulated to be essential for pyrrolizine formation (see Scheme 3) appears not to operate to any appreciable extent with the six-membered analogues, which readily lose the exocyclic double bond to give the iminium system **27** upon essentially irreversible protonation. Is this perhaps another example of the lower reactivity and greater relative stability of double bonds *exo*- to five-membered rings when compared with their six-membered counterparts, as hypothesized by H. C. Brown nearly seven decades ago [68–69]? In Brown’s own cautiously considered words, “Reactions which involve the formation or retention of an exo double bond in a 5-ring derivative will be favored over corresponding reactions which involve the formation or retention of an exo double bond in a 6-ring derivative. Reactions which involve the loss of an exo double bond will be favored in the 6-ring as compared to the corresponding 5-ring derivative” [69].

Since the presence of a proton donor even as weak as acetic acid was deleterious for tetrahydroindolizine formation from the piperidine-containing enaminone, we speculated that a simple polar protic solvent might be sufficiently “acidic” for the necessary *E*/*Z* equilibration and cyclization of **25a**, even though this solvent was unsuitable for the cyclization of the pyrrolidine enaminones (cf. Table 1, entry 3). We were pleased to find that heating a solution of **25a** in ethanol at 100 °C in a capped microwave tube, but at a much lower power setting (50 W), produced the desired product **26a** in 82% yield with little obvious decomposition (Scheme 4). However, the reaction required four hours for the complete consumption of the enaminone. Interestingly, adding silica gel to the reaction mixture shortened the reaction time to 90 minutes. Replacing ethanol with xylene or the aprotic dipolar solvent DMF in the absence of silica gel failed to provide any trace of **26a**, proving that cyclization was not simply a thermally induced reaction, and supporting our premise that a unique protic solvent effect is crucial for facilitating this specific cyclization process.

We extended the successful reaction to the 4-methoxyphenyl- and 4-nitrophenylenaminones **25b** and **25c**, which afforded tetrahydroindolizines **26b** (74%) and **26c** (65%). The reaction times were 1 and 3 hours, respectively. Unexpectedly, the electronic rate dependence that was observed for the five-membered ring enaminones (where electron-withdrawing substituents caused faster cyclization) was reversed for these six-membered systems. At this stage we have too few examples to ascertain whether this is a genuine effect for the six-membered enaminones, and it would be premature to draw inferences about the detailed course of the reaction. It nevertheless suggests that there might be subtle mechanistic differences at work for the two families of enaminones, and further investigations are warranted.

**Scheme 5:**
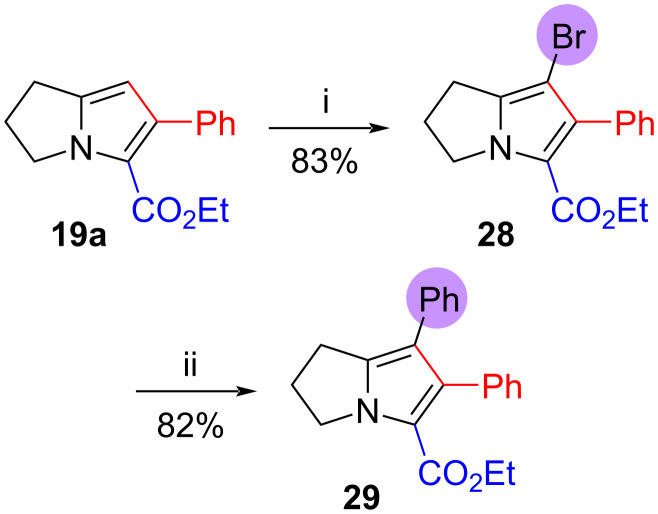
Further functionalization of dihydropyrrolizine **19a**. Reagents and conditions: (i) NBS, DMF, 0 °C, 1 h, then rt, 18 h; (ii) PhB(OH)_2_, Pd(PPh_3_)_4_ (cat.), Na_2_CO_3_, DMF, reflux, 20 h.

Finally, in view of our interest in the synthesis of natural products possessing fully substituted pyrrole rings (e.g., the lamellarin alkaloids [50–52,70]), we also demonstrated that dihydropyrrolizines such as **19a** could easily be functionalized on the unsubstituted pyrrole position (Scheme 5). This site was readily brominated with *N*-bromosuccinimide in *N*,*N*-dimethylformamide [71] to afford ethyl 7-bromo-6-phenyl-2,3-dihydro-1*H*-pyrrolizine-5-carboxylate (**28**) in 83% yield. Palladium(0)-catalyzed Suzuki–Miyaura coupling of **28** with phenylboronic acid efficiently yielded ethyl 6,7-diphenyl-2,3-dihydro-1*H*-pyrrolizine-5-carboxylate (**29**) (82%).

## Conclusion

A variety of *N*-(ethoxycarbonylmethyl) vinylogous amides prepared in three steps from pyrrolidin-2-one (**16**) or piperidin-2-one (**23**) underwent cyclization to yield 2,3-dihydro-1*H*-pyrrolizines **19** or 5,6,7,8-tetrahydroindolizines **26**, respectively, when subjected to microwave heating with silica gel in suitable solvents. The enaminone acted as an electrophile in these cyclization reactions rather than as a nucleophile, as had previously been found with *N*-phenacyl analogues [18]. The formation of products **19** occurred within minutes at 150 °C in xylene, while formation of products **26** occurred in ethanol at 100 °C in a few hours. Yields were usually above 75% in both cases. Noteworthy features of the cyclization are that the reaction conditions are comparatively ‘green’, the products possess a rather uncommon biaryl axis with a pyrrole ring as one of the components, the pyrrole ring itself is created in an unusual manner, and further functionalization of the pyrrole ring is possible. Since both classes of azabicyclic product are well represented in compounds of pharmaceutical interest as well as in natural products, there is considerable synthetic potential in the transformation. Application of the methods described herein to the synthesis of lamellarin alkaloid analogues will be reported in due course.

## Supporting Information

File 1 Experimental details for the synthesis and characterization of all compounds, and copies of ^1^H NMR and ^13^C NMR spectra.
