# Supplementary material for: Silica gel and microwave-promoted synthesis of dihydropyrrolizines and tetrahydroindolizines from enaminones
Source: Beilstein J Org Chem. 2021 Oct 13;17:2543–52. doi: 10.3762/bjoc.17.170 (PMC8551872; doi:10.3762/bjoc.17.170)

BEILSTEIN JOURNAL OF ORGANIC CHEMISTRY

Supporting Information

for

Silica gel and microwave-promoted synthesis of dihydropyrrolizines and tetrahydroindolizines from enaminones

Robin Klintworth, Garreth L. Morgans, Stefania M. Scalzullo, Charles B. de Koning, Willem A. L. van Otterlo and Joseph P. Michael

Beilstein J. Org. Chem. **2021,** *17,* 2543–2552. doi:10.3762/bjoc.17.170

Experimental details for the synthesis and characterization of all compounds, and copies of ^{1}H NMR and ^{13}C NMR spectra

License and Terms: This is a supporting information file under the terms of the Creative Commons Attribution License (https://creativecommons.org/licenses/by/4.0). Please note that the reuse, redistribution and reproduction in particular requires that the author(s) and source are credited and that individual graphics may be subject to special legal provisions.
The license is subject to the *Beilstein Journal of Organic Chemistry* terms and conditions: (https://www.beilstein-journals.org/bjoc/terms)

Table of Contents

1. General remarks S2
2. Experimental details and characterization data S3
3. References S21
4. ^{1}H and ^{13}C NMR spectra S22

1. General remarks

Chemicals (other than those listed below) and deuterated solvents were purchased from commercial sources (Merck, Sigma-Aldrich) and used as received. Merck silica gel (particle size 0.063–0.200 mm) was used for conventional silica gel chromatography, and Merck silica gel (particle size 40–75 μm) for flash column chromatography and for the microwave-mediated cyclizations. Thin-layer chromatography (TLC) was carried out on Merck silica gel 60 F_{254} plates, and compounds were visualized using UV light and/or by exposure to iodine vapor. Solvents for reaction or chromatography were dried and purified, where necessary, by standard methods. Room temperature refers to ambient laboratory temperatures of 18–25 °C. Melting points were recorded on a JM 626 melting point apparatus with microscope and a digital thermometer. ^{1}H and ^{13}C{^{1}H} NMR spectra were recorded on Bruker Avance I 300 MHz, Avance III 400 MHz and Avance III 500 MHz spectrometers at frequencies of 300 MHz, 400 MHz and 500 MHz, respectively, for ^{1}H spectra; and at frequencies of 75 MHz, 101 MHz and 126 MHz, respectively, for ^{13}C spectra. Chemical shifts (δ) of ^{1}H signals recorded in $CDCl_3$ solution are reported as parts per million (ppm) downfield from Me_4Si as internal reference. Chemical shifts (δ) of ^{13}C signals are referenced to the central peak of $CDCl_3$ (77.16 ppm). High resolution mass spectra were obtained on a Bruker Compact Q-TOF mass spectrometer in electrospray positive ionization mode (ESI), or on a Thermo Electron Corporation DFS high resolution magnetic sector mass spectrometer in positive ion mode (EI). FT-IR spectra were recorded on a Bruker Tensor 27 spectrometer equipped with a diamond ATR unit. Microwave heating was performed in capped vials of appropriate size in a CEM Discover microwave reactor, and temperature was monitored by means of an external surface sensor.

The following bromomethyl aryl ketones were prepared from the corresponding methyl ketones by reported procedures: 2-bromo-1-(3,4-dimethoxyphenyl)ethanone [1]; 2-bromo-1-(2-iodophenyl)ethanone, 2-bromo-1-(2-bromophenyl)ethanone and 2-bromo-1-(2-chlorophenyl)ethanone [2]; 2-bromo-1-(2-bromo-4,5-dimethoxyphenyl)ethanone [3]; 2-bromo-1-(naphthalen-1-yl)ethanone [4]; (*E*)-1-bromo-4-phenylbut-3-en-2-one [5]; 2-bromo-1-(furan-2-yl)ethanone and 1-(benzofuran-2-yl)-2-bromoethanone [6]; 2-bromo-1-(thiophen-2-yl)ethanone [7]; 2-bromo-1-(1-toluenesulfonyl-1*H*-indol-3-yl)ethanone [8]; and 1-bromo-3,3-dimethylbutan-2-one (1-bromopinacolone) [9]. All other bromomethyl ketones were purchased from Sigma–Aldrich.

2. Experimental details and characterization data

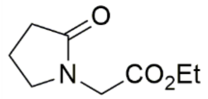

Ethyl 2-(2-oxopyrrolidin-1-yl)acetate (17): A solution of 2-pyrrolidinone (**16**, 5.00 g, 58.8 mmol) in THF (50 mL) was added dropwise over 20 min to a stirred suspension of NaH (60% dispersion in oil, 2.81 g, 70.3 mmol, 1.2 equiv) in THF (100 mL) under an inert atmosphere of Ar gas. The residual material in the dropping funnel was then rinsed into the reaction flask using additional THF (20 mL). Stirring was continued at room temperature for a further 2 h. Ethyl 2-bromoacetate (11.70 g, 70.1 mmol, 1.2 equiv) in THF (50 mL) was then added dropwise to the opaque white emulsion, and the reaction mixture was stirred under Ar at room temperature for 16 h. Water (100 mL) was then cautiously added to the mixture until bubbling ceased and all solids dissolved. The mixture was then extracted into EtOAc (100 mL). The organic phase was separated and the aqueous phase was re-extracted with EtOAc (2 × 50 mL). The combined organic phases were washed with brine (100 mL) and dried over anhydrous Na_2SO_4. The solvent was filtered and evaporated to give a crude yellow oil, which was purified using column chromatography (EtOAc) to afford lactam **17** (7.76 g, 77%) as a colorless oil; ^{1}H NMR (300 MHz, $CDCl_3$): δ = 4.12 (q, *J* = 7.1 Hz, 2H), 3.99 (s, 2H), 3.42 (t, *J* = 7.0 Hz, 2H), 2.36 (t, *J* = 8.1 Hz, 2H), 2.02 (quintet, *J* = 7.6 Hz, 2H), 1.23 (t, *J* = 7.2 Hz, 3H); ^{13}C NMR (75 MHz, $CDCl_3$): δ = 175.7, 168.7, 61.3, 47.8, 44.2, 30.4, 18.0, 14.2; IR (ATR): $\tilde{\nu}$ = 2982 (w), 2938 (w), 1742 (m), 1679 (s), 1289 (m), 1190 (s), 1023 (m) cm^{-1}; HRMS (EI): *m*/*z* calcd for $C_6H_{11}NO_3^+$: 171.0890 $[M]^+$; found: 171.0889. The NMR spectroscopic data agree with previously reported results [10].

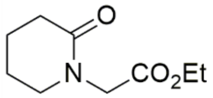

Ethyl 2-(2-oxopiperidin-1-yl)acetate: The above procedure was repeated with 2-piperidinone (**23**, 4.00 g, 40.4 mmol), NaH (60% dispersion in oil, 2.65 g, 66.3 mmol, 1.6 equiv) and ethyl 2-bromoacetate (6.0 mL, ca. 9.0 g, ca. 54 mmol). After addition of water to the reaction mixture, two clear phases formed. The solvents were then removed in vacuo to give an aqueous slurry, which was extracted into CH_2Cl_2 (30 mL). The phases were separated, and the aqueous phase was further extracted with CH_2Cl_2 (2 × 30 mL). The combined organic fractions were washed with brine (80 mL) and dried over anhydrous Na_2SO_4. The solvent was filtered and evaporated under reduced pressure until solids began to precipitate. Hexane (100 mL) was added, and the resulting suspension was heated until the solvent began to boil. After trituration of the solids in the hot solvent and subsequent cooling, they were collected by filtration, washed with additional hexane and dried to provide ethyl 2-(2-oxopiperidin-1-yl)acetate (6.90 g, 92%) as a white solid; m.p.: 75–76°C (lit. [11], 70–71°C); ^{1}H NMR (300 MHz, $CDCl_3$): δ = 4.19 (q, *J* = 7.1 Hz, 2H), 4.11 (s, 2H), 3.37 (br t, *J* = 5.9 Hz, 2H), 2.43 (br t, *J* = 6.0 Hz, 2H), 1.91–1.81 (m, 4H), 1.28 (t, *J* = 7.2 Hz, 3H). ^{13}C NMR (75 MHz, $CDCl_3$): δ = 170.5, 169.2, 61.1, 49.2, 48.7, 32.1, 23.2, 21.4, 14.2; IR (ATR): $\tilde{\nu}$ = 2954 (w), 2906 (w), 2868 (w), 1739 (s), 1627 (s), 1495 (m), 1332 (m), 1286 (m), 1256 (m), 1207 (s), 1178 (s), 1162 (s), 1022 (m), 993 (m), 965 (m) cm^{-1}; HRMS (EI): *m*/*z* calcd for $C_9H_{15}NO_3^+$: 185.1046 $[M]^+$; found; 185.1044. The NMR spectroscopic data agree with previously reported results [11].

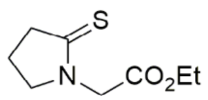

Ethyl 2-(2-thioxopyrrolidin-1-yl) acetate (18): Lawesson's reagent (7.00 g, 17.3 mmol) was added to a solution of ethyl 2-(2-oxopyrrolidin-1-yl)acetate (**17**, 5.00 g, 29.2 mmol) in toluene (1000 mL). The mixture was stirred at 80 °C under an inert atmosphere of Ar gas for 18 h, after which time reaction was deemed complete by TLC. The solvent was evaporated in vacuo and the crude residue was adsorbed onto silica gel (ca. 50 g) and purified by flash column chromatography (20–30% EtOAc in hexane) to provide thiolactam **18** (4.70 g, 25 mmol, 86%) as a yellow oil; ^{1}H NMR (500 MHz, $CDCl_3$): δ = 4.56 (s, 1H), 4.23 (q, *J* = 7.1 Hz, 1H), 3.83 (t, *J* = 7.3 Hz, 2H), 3.08 (t, *J* = 7.9 Hz, 1H), 2.13 (quintet, *J* = 7.8 Hz, 1H), 1.29 (t, *J* = 7.2 Hz, 2H); ^{13}C NMR (75 MHz, $CDCl_3$): δ = 203.9, 167.2, 61.7, 55.6, 49.1, 44.4, 19.9, 14.3; IR (ATR): $\tilde{\nu}$ = 2979 (w), 2919 (w), 2882 (w), 1738 (s), 1504 (s) 1328 (m), 1294 (m), 1224 (m), 1196 (s), 1129 (s), 1021 (s) cm^{-1}; HRMS (EI): *m/z* calcd for $C_8H_{13}NO_2S^+$: 187.0662 [M]$^+$; found: 187.0672.

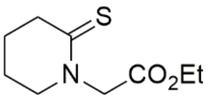

Ethyl 2-(2-thioxopiperidin-1-yl)acetate (24): The above procedure was repeated with ethyl 2-(2-oxopiperidin-1-yl)acetate (3.53 g, 19.1 mmol) in toluene (150 mL) and Lawesson's reagent (7.24 g, 17.9 mmol). After work-up and flash column chromatography, the thiolactam **24** (3.45 g, 90%) was obtained as a colorless oil; ^{1}H NMR (300 MHz, $CDCl_3$): δ = 4.71 (s, 2H), 4.22 (q, *J* = 7.1 Hz, 2H), 3.56 (t, *J* = 6.2 Hz, 2H), 3.01 (t, *J* = 6.4 Hz, 2H), 1.96 (quintet, *J* = 7.2 Hz, 2H), 1.79 (quintet, *J* = 7.3 Hz, 2H,), 1.30 (t, *J* = 7.1 Hz, 3H); ^{13}C NMR (75 MHz, $CDCl_3$): δ = 201.5, 166.9, 60.9, 55.7, 52.1, 41.0, 22.5, 20.2, 13.7; IR (ATR): $\tilde{\nu}$ = 2947 (w), 2870 (w), 1739 (s), 1510 (s), 1349 (s), 1329 (m), 1194 (s), 1162 (s), 1107 (s), 1051 (m), 1023 (s), 944 (m) cm^{-1}; HRMS (EI): *m/z* calcd for $C_9H_{15}NO_2^+$: 201.0818 [M]$^+$; found: 201.0822.

General method for the preparation of enaminones 15a–y and 25a–c: To solution of ethyl 2-(2-thioxopyrrolidin-1-yl)acetate (**18**, 250 mg, 1.34 mmol) or ethyl 2-(2-thioxopiperidin-1-yl)acetate (**24**, 250 mg, 1.24 mmol) in anhydrous MeCN (2 mL) was added the bromomethyl ketone (1.2 equiv), and the reaction mixture was stirred at room temperature overnight. A solution of triethyl phosphite (0.28 mL, *ca.* 270 mg, 1.6 mmol, 1.2–1.3 equiv) or triphenylphosphine (420 mg, 1.60 mmol, 1.2 equiv) (see *Table 2*) and triethylamine (0.23 mL, ca. 160 mg, 1.6 mmol, 1.2–1.3 equiv) in MeCN (5 mL) was then added dropwise over 15 min, and the reaction mixture was then left at room temperature for a further 18 h. The solvent was removed in vacuo, and the crude extract was purified by flash column chromatography (EtOAc–hexane 2:3) to afford the corresponding (*E*)-ethyl 2-[2-(2-aryl/heteroaryl-2-oxoethylidene)pyrrolidin-1-yl]acetates (**15**) or (*E*)-ethyl 2-[2-(2-aryl-2-oxoethylidene)piperidin-1-yl]acetates (**25**). In several cases the enaminones could not be obtained free of phosphorus-containing contaminants, and the impure products were used directly in the cyclization step (see Table 2 in the main article and additional details below). The following compounds were obtained by this method.

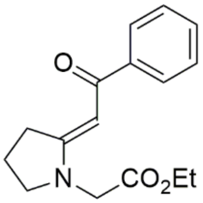

(*E*)-Ethyl 2-[2-(2-oxo-2-phenylethylidene)pyrrolidin-1-y]acetate (15a): Prepared according to the general procedure from thiolactam **18** and 2-bromo-1-phenylethanone (319 mg, 1.60 mmol), with triethyl phosphite for the sulfide contraction. Enaminone **15a** (335 mg, 92%) was obtained as a colorless solid; m.p.: 82-83°C; ^{1}H NMR (300 MHz, $CDCl_3$): δ = 7.84 (dd, *J* = 7.7, 1.8 Hz, 2H), 7.46-7.32 (m, 3H), 5.66 (s, 1H), 4.24 (q, *J*= 7.1 Hz, 2H), 4.06 (s, 2H), 3.56 (t, *J* = 7.3 Hz, 2H), 3.43 (t, *J* = 7.8 Hz, 2H), 2.08 (quintet, *J* = 7.6 Hz, 2H), 1.29 (t, *J* = 7.1 Hz, 3H); ^{13}C NMR (75 MHz, $CDCl_3$): δ = 188.2, 168.1, 167.4, 141.8, 130.6, 128.1, 127.3, 87.3, 61.7, 53.8, 48.2, 33.5, 21.3, 14.3; IR (ATR): $\tilde{\nu}$ = 3060 (w), 2980 (w), 2877 (w), 1736 (m), 1714 (m), 1578 (m), 1524 (m), 1479 (s), 1431 (m), 1302 (m), 1200 (s), 1023 (m), 927 (m), 847 (m), 761 (m), 710 (s), 649 (m) cm^{-1}; HRMS (ESI): *m*/*z* calcd for $C_{16}H_{19}NO_3^+$: 274.1438 $[M + H]^+$; found: 274.1447.

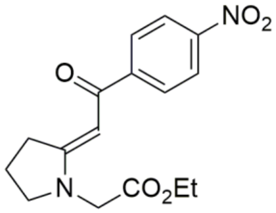

(*E*)-Ethyl 2-{2-[2-(4-nitrophenyl)-2-oxoethylidene]pyrrolidin-1-yl}acetate (15b): Prepared according to the general procedure from thiolactam **18** and 2-bromo-1-(4-nitrophenyl)ethanone (392 mg, 1.61 mmol), with triethyl phosphite for the sulfide contraction. Enaminone **15b** (424 mg, 99%) was obtained as a yellow solid; m.p. 136-138 °C; ^{1}H NMR (300 MHz, $CDCl_3$): δ = 8.16 (d, *J* = 8.8 Hz, 2H), 7.88 (d, *J* = 8.9 Hz, 2H), 5.53 (br s, 1H), 4.18 (q, *J* = 7.1 Hz, 2H), 4.03 (s, 2H), 3.55 (t, *J* = 7.3 Hz, 2H), 3.37 (t, *J* = 7.8 Hz, 2H), 2.05 (quintet, *J* = 7.4 Hz, 2H), 1.23 (t, *J* = 7.1 Hz, 3H);^{13}C NMR (75 MHz, $CDCl_3$): δ = 185.6, 169.0, 167.7, 149.0, 147.3, 128.3, 123.5, 87.2, 62.0, 54.2, 48.4, 34.0, 21.1, 14.4; HRMS (ESI): *m*/*z* calcd for $C_{16}H_{19}N_2O_5^+$: 319.1288 $[M + H]^+$; found: 319.1287.

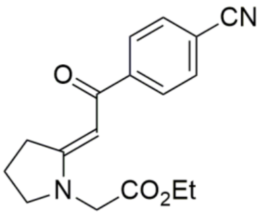

(*E*)-Ethyl 2-{2-[2-(4-cyanophenyl)-2-oxoethylidene]pyrrolidin-1-yl}acetate (15c): Prepared according to the general procedure from thiolactam **18** and 4-(2-bromoacetyl)benzonitrile (360 mg, 1.61 mmol), with triethyl phosphite for the sulfide contraction. Enaminone **15c** (368 mg, 92%) was obtained as a colorless solid; m.p. 116 °C; ^{1}H NMR (300 MHz, $CDCl_3$): δ = 7.90 (d, *J* = 8.3 Hz, 2H), 7.68 (d, *J* = 8.3 Hz, 2H), 5.58 (s, 1H), 4.25 (q, *J* = 7.1 Hz, 2H), 4.07 (s, 2H), 3.61 (t, *J* = 7.3 Hz, 2H), 3.43 (t, *J* = 7.7 Hz, 2H), 2.11 (quintet, *J* = 7.6 Hz, 2H), 1.30 (t, *J* = 7.1 Hz, 3H); ^{13}C NMR (75 MHz, $CDCl_3$): δ = 185.9, 168.9, 167.7, 145.6, 132.2, 127.9, 118.8, 113.9, 87.1, 62.0, 54.2, 48.4, 33.9, 21.2, 14.4; HRMS (ESI): *m*/*z* calcd for $C_{17}H_{19}N_2O_3^+$; 299.1390 $[M + H]^+$; found: 299.1389.

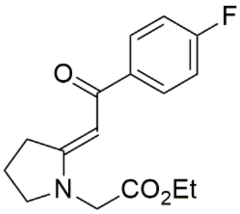

(*E*)-Ethyl 2-{2-[2-(4-fluorophenyl)-2-oxoethylidene]pyrrolidin-1-yl}acetate (15d): Prepared according to the general procedure from thiolactam **18** and 2-bromo-1-(4-fluorophenyl)ethanone (349 mg, 1.61 mmol), with triphenylphosphine for the sulfide contraction. Enaminone **15d** (350 mg, 90%) was obtained as a colorless solid; m.p. 80-81 °C; ^{1}H NMR (300 MHz, $CDCl_3$): δ = 7.95–7.73 (m, 2H), 7.14–6.80 (m, 2H), 5.61 (s, 1H), 4.24 (q, *J* = 7.1 Hz, 2H), 4.06 (s, 2H), 3.57 (t, *J* = 7.3 Hz, 2H), 3.41 (t, *J* = 7.5 Hz, 2H), 2.07 (quintet, *J* = 7.6 Hz, 2H), 1.29 (t, *J* = 7.2 Hz, 3H); ^{13}C NMR (75 MHz, $CDCl_3$): δ = 186.6, 168.0, 167.5, 164.4 (d, J_{C-F} = 250.1 Hz), 138.0 (d, J_{C-F} = 3.0 Hz), 129.6 (d, J_{C-F} = 8.7 Hz), 114.9 (d, J_{C-F} = 21.5

Hz), 86.8, 61.7, 53.8, 48.3, 33.5, 21.2, 14.3; HRMS (ESI): *m*/*z* calcd for $C_{16}H_{19}FNO_3^+$: 292.1343 [M + H]$^+$; found: 292.1383.

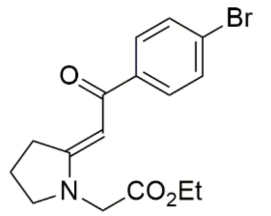

(*E*)-Ethyl 2-{2-[2-(4-bromophenyl)-2-oxoethylidene]pyrrolidin-1-yl}acetate (15e): Prepared according to the general procedure from thiolactam **18** and 2-bromo-1-(4-bromophenyl)ethanone (448 mg, 1.61 mmol), with triethyl phosphite for the sulfide contraction. Enaminone **15e** (437 mg, 93%) was obtained as a colorless solid; m.p. 122-123 °C; ^{1}H NMR (300 MHz, $CDCl_3$): δ = 7.70 (d, *J* = 8.5 Hz, 2H), 7.50 (d, *J* = 8.5 Hz, 2H), 5.58 (br s, 1H), 4.23 (q, *J* = 7.1 Hz, 2H), 4.05 (s, 2H), 3.57 (t, *J* = 7.3 Hz, 2H), 3.41 (t, *J* = 7.8 Hz, 2H), 2.08 (quintet, *J* = 7.6 Hz, 2H), 1.29 (t, *J* = 7.1 Hz, 3H). ^{13}C NMR (75 MHz, $CDCl_3$): δ = 186.8, 168.0, 167.9, 140.6, 131.4, 129.1, 125.2, 86.9, 61.8, 54.0, 48.4, 33.7, 21.3, 14.4. HRMS (ESI): *m*/*z* calcd for $C_{16}H_{19}BrNO_2^+$: 352.0543 [M + H]$^+$; found: 352.0560.

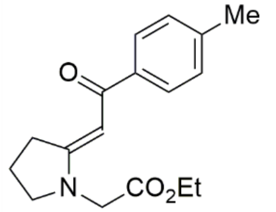

(*E*)-Ethyl 2-{2-[2-(4-methylphenyl)-2-oxoethylidene]pyrrolidin-1-yl}acetate (15f): Prepared according to the general procedure from thiolactam **18** and 2-bromo-1-(4-methylphenyl)ethanone (343 mg, 1.61 mmol), with triethyl phosphite for the sulfide contraction. Enaminone **15f** (315 mg, 82%) was obtained as a colorless solid; m.p. 95°C; ^{1}H NMR (500 MHz, $CDCl_3$): δ = 7.75 (d, *J* = 8.2 Hz, 2H), 7.18 (d, *J* = 7.9 Hz, 2H), 5.65 (s, 1H), 4.22 (q, *J* = 7.1 Hz, 2H), 4.04 (s, 2H), 3.54 (t, *J* = 7.2 Hz, 2H), 3.40 (t, *J* = 7.8 Hz, 2H), 2.36 (s, 3H), 2.05 (quintet, *J* = 7.5 Hz, 2H), 1.28 (d, *J* = 7.1 Hz, 3H); ^{13}C NMR (75 MHz, $CDCl_3$): δ = 187.9, 168.2, 167.0, 140.9, 139.0, 128.8, 127.4, 87.1, 61.6, 53.7, 48.2, 33.4, 21.5, 21.3, 14.3; HRMS (ESI): *m*/*z* calcd for $C_{17}H_{22}NO_3^+$: 288.1594 [M + H]$^+$; found: 288.1606.

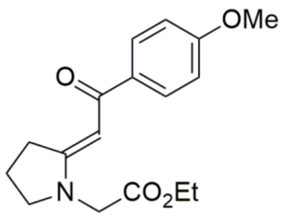

(*E*)-Ethyl 2-{2-[2-(4-methoxyphenyl)-2-oxoethylidene]pyrrolidin-1-yl}acetate (15g): Prepared according to the general procedure from thiolactam **18** and 2-bromo-1-(4-methoxyphenyl)ethanone (369 mg, 1.61 mmol), with triethyl phosphite for the sulfide contraction. Enaminone **15g** (340 mg, 84%) was obtained as a colorless solid; m.p. 81-83 °C; ^{1}H NMR (500 MHz, $CDCl_3$): δ = 7.83 (d, *J* = 8.8 Hz, 2H), 6.88 (d, *J* = 8.8 Hz, 2H), 5.64 (s, 1H), 4.22 (q, *J* = 7.1 Hz, 2H), 4.04 (s, 2H), 3.82 (s, 3H), 3.54 (t, *J* = 7.2 Hz, 2H), 3.40 (t, *J* = 7.8 Hz, 2H), 2.05 (quintet, *J* = 7.5 Hz, 2H), 1.28 (t, *J* = 7.1 Hz, 3H). ^{13}C NMR (126 MHz, $CDCl_3$): δ = 187.2, 168.3, 166.9, 161.8, 134.4, 129.3, 113.3, 86.9, 61.7, 55.4, 53.7, 48.3, 33.5, 21.4, 14.3; HRMS (ESI): *m*/*z* calcd for $C_{17}H_{22}NO_4^+$: requires 304.1543 [M + H]$^+$; found: 304.1558.

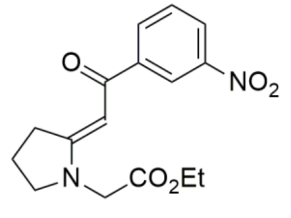

(*E*)-Ethyl 2-{2-[2-(3-nitrophenyl)-2-oxoethylidene]pyrrolidin-1-yl}acetate (15h): Prepared according to the general procedure from thiolactam **18** and 2-bromo-1-(3-nitrophenyl)ethanone (392 mg, 1.61 mmol), with triethyl phosphite for the sulfide contraction. Enaminone **15h** (420 mg, 99%) was obtained as a yellow solid; m.p. 126-127 °C; ^{1}H NMR (300 MHz, $CDCl_3$): δ = 8.63 (t, *J* = 2.1 Hz, 1H), 8.26 (ddd, *J* = 8.4, 2.3, 0.9 Hz, 1H), 8.18 (dt, *J* = 7.8, 1,2 Hz, 1H), 7.57 (t, *J* = 7.9 Hz, 1H), 5.64 (s, 1H), 4.27 (q, *J* = 7.1 Hz, 2H), 4.13 (s, 2H), 3.63 (t, *J* = 7.3 Hz, 2H), 3.45 (t, *J* = 7.8 Hz, 2H), 2.12 (quintet, *J* =

7.6 Hz, 2H), 1.32 (t, J = 7.1 Hz, 3H); ^{13}C NMR (75 MHz, $CDCl_3$); δ = 184.8, 168.9, 167.7, 148.1, 143.3, 133.3, 129.2, 125.0, 122.1, 86.4, 61.9, 54.1, 48.2, 33.8, 21.1, 14.2; HRMS (ESI): m/z calcd for $C_{16}H_{19}N_2O_5^+$: 319.1288 [M + H]$^+$; found: 319.1281.

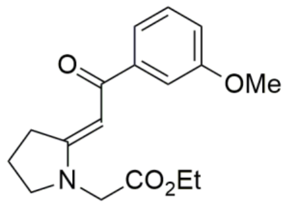

(*E*)-Ethyl 2-{2-[2-(3-methoxyphenyl)-2-oxoethylidene]pyrrolidin-1-yl}acetate (15i): Prepared according to the general procedure from thiolactam **18** and 2-bromo-1-(3-methoxyphenyl)ethanone (369 mg, 1.61 mmol), with triphenylphosphine for the sulfide contraction. Enaminone **15i** contaminated with phosphine-derived byproducts was obtained as a brown oil (450 mg), which was used without further purification in the cyclization step (vide infra). Discernible signals are as follows: ^{1}H NMR (300 MHz, $CDCl_3$): δ = 7.47–7.35 (m, 2H), 7.28 (t, J = 7.8 Hz, 1H), 6.97 (ddd, J = 8.1, 2.6, 0.9 Hz, 1H), 5.65 (s, 1H), 4.23 (q, J = 7.1 Hz, 2H), 4.05 (s, 2H), 3.84 (s, 3H), 3.56 (t, J = 7.3 Hz, 2H), 3.42 (t, J = 7.7 Hz, 2H), 2.07 (quintet, J = 7.6 Hz, 2H), 1.29 (t, J = 7.2 Hz, 3H); HRMS (ESI): m/z calcd for $C_{17}H_{22}NO_4^+$: 304.1543 [M + H]$^+$; found: 304.1549.

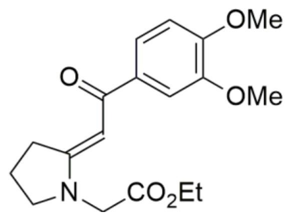

(*E*)-Ethyl 2-{2-[2-(3,4-dimethoxyphenyl)-2-oxoethylidene]pyrrolidin-1-yl}acetate (15j): Prepared according to the general procedure from thiolactam **18** and 2-bromo-1-(3,4-dimethoxyphenyl)ethanone[1] (417 mg, 1.61 mmol), with triethyl for the sulfide contraction. Enaminone **15j** contaminated with phosphite-derived byproducts was obtained as an amber oil (445 mg), which was used without further purification in the cyclization step (vide infra). Discernible signals are as follows: ^{1}H NMR (300 MHz, $CDCl_3$): δ = 7.54 (d, J = 1.4 Hz, 1H), 7.43 (dd, J = 8.3, 1.5 Hz, 1H), 6.84 (d, J = 8.3 Hz, 1H), 5.67 (s, 1H), 4.24 (q, J = 7.1 Hz, 2H), 4.06 (s, 2H), 3.93 (s, 3H), 3.91 (s, 3H), 3.56 (t, J = 7.2 Hz, 2H), 3.41 (t, J = 7.0 Hz, 2H), 2.07 (quintet, J = 7.4 Hz, 2H), 1.29 (t, J = 7.2 Hz, 3H); ^{13}C NMR (75 MHz, $CDCl_3$): δ = 187.0, 168.3, 166.8, 151.2, 148.7, 134.6, 120.5, 110.5, 109.9, 86.7, 61.6, 55.94, 55.87, 53.6, 48.3, 33.3, 21.3, 14.2; HRMS (ESI): m/z calcd for $C_{18}H_{24}NO_4^+$: 334.1649 [M + H]$^+$; found: 334.1642.

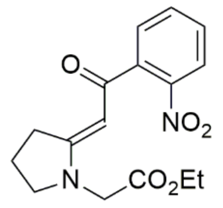

(*E*)-Ethyl 2-{2-[2-(2-nitrophenyl)-2-oxoethylidene]pyrrolidin-1-yl}acetate (15k): Prepared according to the general procedure from thiolactam **18** and 2-bromo-1-(2-nitrophenyl)ethanone (392 mg, 1.61 mmol), with triethyl phosphite for the sulfide contraction. Enaminone **15k** (370 mg, 87%) was obtained as an orange gum; ^{1}H NMR (300 MHz, $CDCl_3$): δ = 7.86 (d, J = 8.4 Hz, 1H), 7.58 (td, J = 7.4, 0.9 Hz, 1H), 7.51–7.41 (m, 2H), 5.13 (s, 1H), 4.22 (q, J = 7.1 Hz, 2H), 3.97 (s, 2H), 3.58 (t, J = 7.3 Hz, 2H), 3.38 (t, J = 7.8 Hz, 2H), 2.09 (quintet, J = 7.5 Hz, 2H), 1.28 (t, J = 7.1 Hz, 3H); ^{13}C NMR (75 MHz, $CDCl_3$): δ = 187.3, 167.9, 167.7, 147.6, 140.3, 132.9, 129.2, 128.7, 124.0, 89.2, 61.9, 54.0, 48.2, 33.7, 21.1, 14.3; HRMS (ESI): m/z calcd for $C_{16}H_{19}N_2O_5^+$: 319.1288 [M + H]$^+$; found: 319.1313.

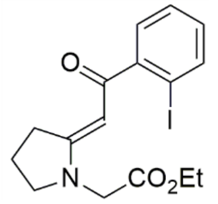

(*E*)-Ethyl 2-{2-[2-(2-iodophenyl)-2-oxoethylidene]pyrrolidin-1-yl}acetate (15l): Prepared according to the general procedure from thiolactam **18** and 2-bromo-1-(2-iodophenyl)ethanone[2] (377 mg, 1.61 mmol), with triethyl phosphite for the sulfide contraction. Enaminone **15l** (497 mg, 93%) was obtained as an amber oil; ^{1}H NMR (300 MHz, $CDCl_3$): δ = 7.81 (d, *J* = 7.9 Hz, 1H), 7.32 (d, *J* = 4.3 Hz, 2H), 6.99 (dt, *J* = 8.0, 4.6 Hz, 1H), 5.16 (s, 1H), 4.19 (q, *J* = 7.1 Hz, 2H), 4.00 (s, H), 3.56 (t, *J* = 7.3 Hz, 2H), 3.33 (br t, *J* = 7.3 Hz, 2H), 2.06 (quintet, *J* = 7.4 Hz, 2H), 1.26 (t, *J* = 7.1 Hz, 3H); ^{13}C NMR (75 MHz, $CDCl_3$): δ = 191.2, 167.6, 167.1, 148.8, 139.4, 129.6, 127.7, 127.5, 92.3, 90.2, 61.5, 53.6, 47.8, 33.3, 20.9, 14.1; HRMS (ESI): *m*/*z* calcd for $C_{16}H_{19}INO_3^+$: 400.0404 [M + H]$^+$; found: 400.0411.

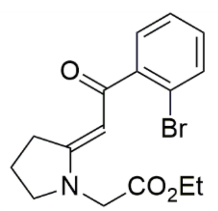

(*E*)-Ethyl 2-{2-[2-(2-bromophenyl)-2-oxoethylidene]pyrrolidin-1-yl}acetate (15m): Prepared according to the general procedure from thiolactam **18** and 2-bromo-1-(2-bromophenyl)ethanone[2] (447 mg, 1.61 mmol), with triethyl phosphite for the sulfide contraction. Enaminone **15m** contaminated with phosphite-derived by-product(s) was obtained as a brown oil (0.480 g), which was used without further purification in the cyclization step (vide infra). Discernible signals are as follows: ^{1}H NMR (300 MHz, $CDCl_3$): δ = 7.53 (dd, *J* = 7.9, 1.0 Hz, 1H), 7.36 (dd, *J* = 7.6, 1.8 Hz, 1H), 7.29 (dd, *J* = 7.4, 1.2 Hz, 1H), 7.16 (td, *J* = 7.6, 1.9 Hz, 1H), 5.23 (br s, 1H), 4.20 (q, *J* = 7.1 Hz, 2H), 4.00 (s, 2H), 3.56 (t, *J* = 7.3 Hz, 2H), 3.35 (t, *J* = 7.7 Hz, 2H), 2.08 (quintet, *J* = 7.6 Hz, 2H), 1.27 (t, *J* = 7.1 Hz, 3H); HRMS (ESI): calcd for $C_{16}H_{19}BrNO_3^+$: 352.0543 [M + H]$^+$; found: 352.0543.

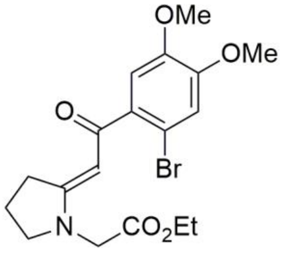

(*E*)-Ethyl 2-{2-[2-(2-bromo-4,5-dimethoxyphenyl)-2-oxoethylidene]pyrrolidin-1-yl}acetate (15n): Prepared according to the general procedure from thiolactam **18** and 2-bromo-1-(2-bromo-4,5-dimethoxyphenyl)ethanone[3] (544 mg, 1.61 mmol), with triethyl phosphite for the sulfide contraction. Enaminone **15n** (512 mg, 93%) slightly contaminated with P-derived by-product(s) was obtained as a cream-colored solid, and used as such in the cyclization step (vide infra); m.p. 66-69 °C; ^{1}H NMR (300 MHz, $CDCl_3$) δ 7.00 (s, 1H), 6.99 (s, 1H), 5.35 (s, 1H), 4.21 (q, *J* = 7.1 Hz, 2H), 4.02 (s, 2H), 3.88 (s, 3H), 3.86 (s, 3H), 3.57 (t, *J* = 7.3 Hz, 2H), 3.37 (t, *J* = 7.7 Hz, 2H), 2.09 (quintet, *J* = 7.5 Hz, 2H), 1.28 (t, *J* = 7.1 Hz, 3H). ^{13}C NMR (75 MHz, $CDCl_3$) δ 189.2, 168.0, 166.9, 149.9, 148.3, 137.5, 115.8, 112.3, 109.9, 91.6, 61.8, 56.4, 56.2, 53.8, 48.1, 33.6, 21.3, 14.3.

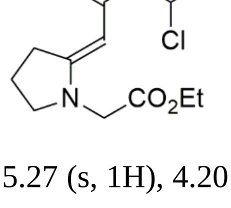

(*E*)-Ethyl 2-{2-[2-(2-chlorophenyl)-2-oxoethylidene]pyrrolidin-1-yl}acetate (15o): Prepared according to the general procedure from thiolactam **18** and 2-bromo-1-(2-chlorophenyl)ethanone[2] (357 mg, 1.53 mmol), with triethyl phosphite for the sulfide contraction. Enaminone **15o** (378 mg, 92%) was obtained as an amber oil; ^{1}H NMR (300 MHz, $CDCl_3$): δ = 7.41 (dd, *J* = 5.8, 3.5 Hz, 1H), 7.37–7.29 (m, 1H), 7.30–7.18 (m, 2H), 5.27 (s, 1H), 4.20 (q, *J* = 7.1 Hz, 2H), 3.99 (s, 2H), 3.56 (t, *J* = 7.3 Hz, 2H), 3.37 (t, *J* = 7.8 Hz, 2H), 2.07 (quintet, *J* = 7.5 Hz, 2H), 1.27 (t, *J* = 7.1 Hz, 3H); ^{13}C NMR (75 MHz, $CDCl_3$): δ = 189.0, 167.8, 167.1,

143.3, 130.5, 129.9, 129.7, 128.9, 126.6, 91.5, 61.7, 53.8, 48.0, 33.5, 21.1, 14.2; HRMS (ESI): *m/z* calcd for $C_{16}H_{19}ClNO_3^+$: 308.1048 [M + H]$^+$; found: 308.1052.

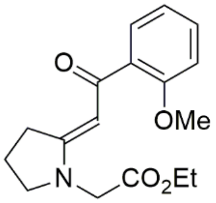

(*E*)-Ethyl 2-{2-[2-(2-methoxyphenyl)-2-oxoethylidene]pyrrolidin-1-yl}acetate (15p): Prepared according to the general procedure from thiolactam **18** and 2-bromo-1-(2-methoxyphenyl)ethanone (367 mg, 1.60 mmol), with triphenylphosphine for the sulfide contraction. Enaminone **15p** contaminated with byproduct(s) was obtained as an amber oil (456 mg), which was used without further purification in the cyclization step (vide infra). Discernible signals are as follows: ^{1}H NMR (300 MHz, $CDCl_3$): δ = 7.54 (dd, *J* = 7.5, 1.8 Hz, 1H), 7.31 (ddd, *J* = 8.3, 7.4, 1.8 Hz, 1H), 6.98–6.89 (m, 2H), 5.56 (s, 1H), 4.20 (q, J = 7.1 Hz, 2H), 3.98 (s, 2H), 3.83 (s, 3H), 3.52 (t, *J* = 7.2 Hz, 2H), 3.37 (t, *J* = 7.8 Hz, 2H), 2.04 (quintet, *J* = 7.6 Hz, 2H), 1.26 (t, *J* = 7.1 Hz, 3H); ^{13}C NMR (75 MHz, $CDCl_3$): δ = 188.9, 168.1, 166.1, 156.9, 133.1, 130.5, 129.6, 120.4, 111.5, 92.3, 61.4, 55.7, 53.5, 48.0, 33.4, 21.3, 14.2; HRMS (ESI): calcd for $C_{17}H_{22}NO_4^+$: 304.1543 [M + H]$^+$; found: 304.1543.

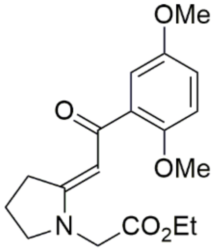

(*E*)-Ethyl 2-{2-[2-(2,5-dimethoxyphenyl)-2-oxoethylidene]pyrrolidin-1-yl}acetate (15q): Prepared according to the general procedure from thiolactam **18** and 2-bromo-1-(2,5-dimethoxyphenyl)ethanone (417 mg, 1.61 mmol), with triphenylphosphine for the sulfide contraction. Enaminone **15q** contaminated with phosphine derived byproducts was obtained as an amber oil (432 mg), which was used without further purification in the cyclization step (vide infra). Discernible signals are as follows: ^{1}H NMR (300 MHz, $CDCl_3$): δ = 7.18 (d, *J* = 2.6 Hz, 1H), 6.89 – 6.83 (m, 2H), 5.64 (s, 1H), 4.21 (q, *J* = 7.1 Hz, 2H), 4.00 (s, 2H), 3.80 (s, 3H), 3.78 (s, 3H), 3.54 (t, *J* = 7.3 Hz, 2H), 3.39 (t, *J* = 7.6 Hz, 2H), 2.06 (quintet, *J* = 7.6 Hz, 2H), 1.28 (t, *J* = 7.1 Hz, 3H); ^{13}C NMR (75 MHz, $CDCl_3$): δ = 188.3, 168.2, 166.5, 153.7, 151.5, 133.7, 116.9, 114.3, 113.7, 92.3, 61.6, 56.8, 55.9, 53.6, 48.2, 33.6, 21.4, 14.3; HRMS (ESI): *m/z* calcd for $C_{18}H_{24}NO_4^+$: 334.1649 [M + H]$^+$; found: 334.1674.

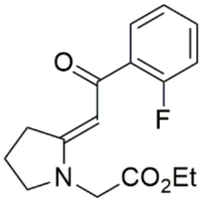

(*E*)-Ethyl 2-{2-[2-(2-fluorophenyl)-2-oxoethylidene]pyrrolidin-1-yl}acetate (15r): Prepared according to the general procedure from thiolactam **18** and 2-bromo-1-(2-fluorophenyl)ethanone (349 mg, 1.61 mmol), with triphenylphosphine for the sulfide contraction. Enaminone **15r** contaminated with phosphine-derived by-products was obtained as an amber oil (440 mg), which was used without further purification in the cyclization step (vide infra). The sample could not be unambiguously characterized by NMR spectroscopy. HRMS (EI): calcd for $C_{16}H_{19}FNO_3^+$: 292.1343 [M + H]$^+$; found: 292.1367.

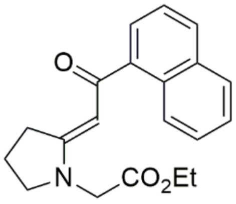

(*E*)-Ethyl 2-{2-[2-(naphthalen-1-yl)-2-oxoethylidene]pyrrolidin-1-yl}acetate (15s): Prepared according to the general procedure from thiolactam **18** and 2-bromo-1-(naphthalen-1-yl)ethanone[4] (399 mg, 1.60 mmol), with triphenylphosphine for the sulfide contraction. Enaminone **15s** contaminated with phosphine-derived byproducts was obtained as a brown oil (470 mg), which was used without further purification in

the cyclization step (vide infra). Discernible signals are as follows: ^{1}H NMR (300 MHz, $CDCl_3$): δ = 8.47–8.22 (m, 1H), 7.82 (d, *J* = 8.9 Hz, 2H), 7.58 (d, *J* = 7.0 Hz, 1H), 7.52–7.37 (m, 3H), 5.41 (s, 1H), 4.17 (q, *J* = 7.1 Hz, 2H), 3.95 (s, 2H), 3.58–3.49 (m, 2H), 3.44 (t, *J* = 7.8 Hz, 2H), 2.14–2.00 (m, 2H), 1.22 (t, *J* = 7.1 Hz, 3H); HRMS (ESI): *m*/*z* calcd for $C_{20}H_{22}NO_3^+$: 324.1594 [M + H]$^+$; found: 324.1610.

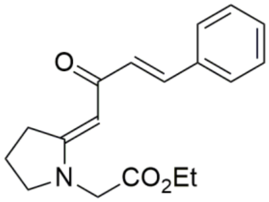

(*E*)-Ethyl 2-{2-[2-oxo-2-(styryl)ethylidene]pyrrolidin-1-yl}acetate (15t): Prepared according to the general procedure from thiolactam **18** and (*E*)-1-bromo-4-phenylbut-3-en-2-one[5] (362 mg, 1.61 mmol), with triethyl phosphite for the sulfide contraction. Enaminone **15t** (351 mg, 88%) was obtained as a pale brown solid; m.p. 90-94 °C; ^{1}H NMR (300 MHz, $CDCl_3$): δ = 7.52–7.38 (m, 3H), 7.30–7.19 (m, 3H), 6.68 (d, *J* = 15.8 Hz, 1H), 5.08 (s, 1H), 4.15 (q, *J* = 7.1 Hz, 2H), 3.94 (s, 2H), 3.45 (t, *J* = 7.3 Hz, 2H), 3.31 (t, *J* = 7.8 Hz, 2H), 1.96 (quintet, *J* = 7.5 Hz, 2H), 1.22 (t, *J* = 7.2 Hz, 3H); ^{13}C NMR (75 MHz, $CDCl_3$): δ = 185.7, 168.0, 167.1, 137.8, 136.0, 129.9, 129.1, 128.7, 127.9, 91.5, 61.7, 53.7, 48.1, 33.5, 21.2, 14.3; HRMS (ESI): *m*/*z* calcd for $C_{18}H_{22}NO_3^+$: 300.1594 [M + H]$^+$; found: 300.1587.

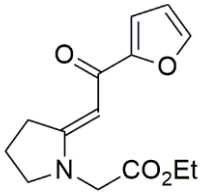

(*E*)-Ethyl 2-{2-[2-(furan-2-yl)-2-oxoethylidene]pyrrolidin-1-yl}acetate (15u): Prepared according to the general procedure from thiolactam **18** and 2-bromo-1-(furan-2-yl)ethanone[6] (304 mg, 1.61 mmol), with triethyl phosphite for the sulfide contraction. Enaminone **15u** (329 mg, 93%) was obtained as a cream-colored solid; m.p. 82-85 °C; ^{1}H NMR (300 MHz, $CDCl_3$): δ = 7.46-7.38 (m, 1H), 6.98 (dd, *J* = 3.4, 0.9 Hz, 1H), 6.42 (dd, *J* = 3.4, 1.7 Hz, 1H), 5.62 (s, 1H), 4.22 (q, *J* = 7.2 Hz, 2H),4.05 (s, 2H), 3.53 (t, *J* = 7.3 Hz, 2H), 3.38 (t, *J* = 7.8 Hz, 2H), 2.04 (quintet, *J* = 7.6 Hz, 2H), 1.28 (t, *J* = 7.1 Hz, 3H); ^{13}C NMR (75 MHz, $CDCl_3$): δ = 177.3, 168.1, 167.3, 155.8, 143.7, 112.6, 111.8, 86.4, 61.8, 53.7, 48.2, 33.5, 21.3, 14.3; HRMS (ESI): *m*/*z* calcd for $C_{14}H_{18}NO_4^+$: 264.1230 [M + H]$^+$; found: 264.1232.

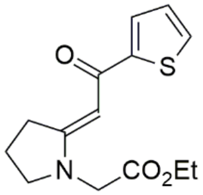

(*E*)-Ethyl 2-{2-[2-oxo-2-(thiophen-2-yl)ethylidene]pyrrolidin-1-yl}acetate (15v): Prepared according to the general procedure from thiolactam **18** and 2-bromo-1-(thiophen-2-yl)ethanone[7] (330 mg, 1.61 mmol), with triphenylphosphine for the sulfide contraction. Enaminone **15v** contaminated with phosphine-derived by-products was obtained as an amber oil (440 mg), which was used without further purification in the cyclization step (vide infra). A small analytical sample, obtained after a similar attempted synthesis of the target enaminone using triethyl phosphite as thiophile, could be recrystallized from cold diethyl ether, providing a low yield of pure material. Characterization data for this sample of **15v** are as follows: m.p. 78-79 °C; ^{1}H NMR (300 MHz, $CDCl_3$): δ = 7.48 (dd, *J*=3.7, 1.2 Hz, 1H), 7.36 (dd, *J*=5.0, 1.2 Hz, 1H), 6.98 (dd, *J*=5.0, 3.7 Hz, 1H), 5.50 (s, 1H), 4.17 (q, *J* = 7.1 Hz, 2H), 3.99 (s, 2H), 3.49 (t, *J* = 7.3 Hz, 2H), 3.33 (t, *J* = 7.8 Hz, 2H), 1.99 (quintet, *J* = 7.6 Hz, 2H), 1.23 (t, *J* = 7.2 Hz, 3H); ^{13}C NMR (75 MHz, $CDCl_3$): δ = 180.3, 167.9, 166.9, 148.8, 129.7, 127.5, 127.4, 86.5, 61.5, 53.6, 48.0, 33.3, 21.0, 14.1; HRMS (ESI): *m*/*z* calcd for $C_{14}H_{18}NO_3S^+$: 280.1002 [M + H]$^+$; found: 280.1020.

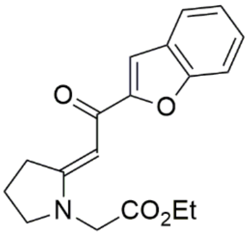

(*E*)-Ethyl 2-{2-[2-(benzofuran-2-yl)-2-oxoethylidene]pyrrolidin-1-yl}acetate (15w): Prepared according to the general procedure from thiolactam **18** and 1-(benzofuran-2-yl)-2-bromoethanone[6] (384 mg, 1.61 mmol), with triphenylphosphine for the sulfide contraction. Enaminone **15w** (389 mg, 93%) was obtained as a cream-colored solid; m.p. 90-91 °C; ^{1}H NMR (400 MHz, $CDCl_3$): δ = 7.63 (d, *J* = 7.8 Hz, 1H), 7.52 (d, *J* = 8.3 Hz, 1H), 7.40–7.31 (m, 2H), 7.24 (t, *J* = 7.5 Hz, 1H), 5.83 (s, 1H), 4.27 (q, *J* = 7.1 Hz, 2H), 4.12 (s, 2H), 3.58 (t, *J* = 7.3 Hz, 2H), 3.46 (t, *J* = 7.8 Hz, 2H), 2.09 (quintet, *J* = 7.6 Hz, 2H), 1.32 (t, *J* = 7.1 Hz, 3H); ^{13}C NMR (101 MHz, $CDCl_3$): δ = 178.0, 168.1, 167.9, 156.5, 155.1, 128.3, 126.2, 123.2, 122.5, 112.0, 108.3, 87.0, 61.8, 53.9, 48.3, 33.8, 21.2, 14.3; HRMS (ESI): *m*/*z* calcd for $C_{18}H_{20}NO_4^+$: 314.1387 [M + H]$^+$; found: 314.1401.

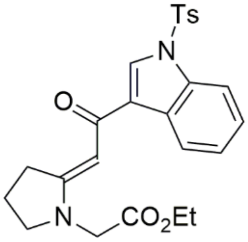

(*E*)-Ethyl 2-{2-[2-oxo-2-(1-tosyl-1*H*-indol-3-yl)ethylidene]pyrrolidin-1-yl}acetate (15x): Prepared according to the general procedure from thiolactam **18** and 2-bromo-1-(1-tosyl-1*H*-indol-3-yl)ethanone[8] (631 mg, 1.61 mmol), with triphenylphosphine for the sulfide contraction. Enaminone **15x** (548 mg, 88%) was obtained as a cream-colored solid; m.p. 143-146 °C; ^{1}H NMR (300 MHz, $CDCl_3$): δ = 8.38– 8.20 (m, 1H), 8.01 (s, 1H), 7.91 (dd, *J* = 7.0, 2.6 Hz, 1H), 7.80–7.70 (m, 2H), 7.35–7.22 (m, 2H), 7.19 (br d, *J* = 8.1 Hz, 2H), 5.54 (s, 1H), 4.27 (q, *J* = 7.1 Hz, 2H), 4.08 (s, 2H), 3.54 (t, *J* = 7.2 Hz, 2H), 3.42 (t, *J* = 7.7 Hz, 2H), 2.31 (s, 3H), 2.06 (quintet, *J* = 7.6 Hz, 2H), 1.33 (t, *J* = 7.1 Hz, 3H); ^{13}C NMR (75 MHz, $CDCl_3$): δ = 183.8, 168.1, 166.5, 145.4, 135.2, 135.0, 130.0, 129.0, 128.0, 127.0, 125.04, 124.97, 124.1, 123.1, 113.1, 88.5, 61.7, 53.6, 48.2, 33.5, 21.6, 21.3, 14.3; HRMS (ESI): *m*/*z* calcd for $C_{25}H_{27}N_2O_5S^+$: 467.1635 [M + H]$^+$; found: 467.1656.

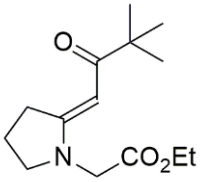

(*E*)-Ethyl 2-{2-[2-(*tert*-butyl)-2-oxoethylidene]pyrrolidin-1-yl}acetate (15y): Prepared according to the general procedure from thiolactam **18** and 1-bromo-3,3-dimethylbutan-2-one[9] (288 mg, 1.61 mmol), with triphenylphosphine for the sulfide contraction. Enaminone **15y** (192 mg, 57%) was obtained as a colorless oil; ^{1}H NMR (300 MHz, $CDCl_3$): δ = 5.17 (s, 1H), 4.22 (q, *J* = 7.2 Hz, 2H), 3.95 (s, 2H), 3.49 (t, *J* = 7.2 Hz, 2H), 3.24 (t, *J* = 7.7 Hz, 2H), 2.00 (quintet, *J* = 7.5 Hz, 2H), 1.29 (t, *J* = 7.2 Hz, 3H), 1.12 (s, 9H); ^{13}C NMR (75 MHz, $CDCl_3$): δ = 203.2, 168.5, 166.0, 86.0, 61.5, 53.5, 48.3, 42.6, 33.1, 27.9, 21.4, 14.4; HRMS (ESI): *m*/*z* calcd for $C_{14}H_{24}NO_3^+$: 254.1751 [M + H]$^+$; found: 254.1767.

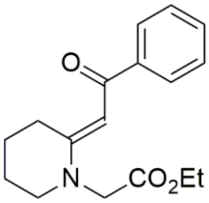

(*E*)-Ethyl 2-[2-(2-oxo-2-phenylethylidene)piperidin-1-yl]acetate (25a): Prepared according to the general procedure from thiolactam **24** and 2-bromo-1-phenylethanone (297 mg, 1.49 mmol), with triethyl phosphite for the sulfide contraction. Enaminone **25a** (317 mg, 89%) was obtained as an amber oil; ^{1}H NMR (300 MHz, $CDCl_3$): δ = 7.79 (dd, *J* = 7.6, 2.0 Hz, 2H), 7.45–7.24 (m, 3H), 5.55 (s, 1H), 4.23 (q, *J* = 7.1 Hz, 2H), 3.96 (s, 2H), 3.40 (t, *J* = 6.1 Hz, 2H), 3.32 (t, *J* = 6.5 Hz, 2H), 1.82 (quintet, *J* = 6.0 Hz, 2H), 1.70 (quintet, *J* = 6.3 Hz, 2H), 1.27 (t, *J* = 7.1 Hz, 3H); ^{13}C NMR (75 MHz, $CDCl_3$): δ = 187.5, 168.4, 164.3, 142.4, 130.1, 127.8,

126.9, 91.1, 61.2, 54.1, 51.7, 28.0, 22.8, 19.1, 14.0. HRMS (ESI): *m*/*z* calcd for $C_{17}H_{22}NO_3^+$: 288.1594 [M + H]$^+$; found: 288.1608.

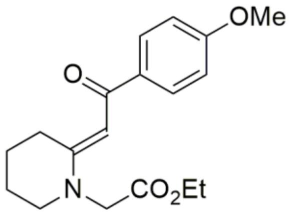

(*E*)-Ethyl 2-{2-[2-(4-methoxyphenyl)-2-oxoethylidene]piperidin-1-yl}acetate (25b): Prepared according to the general procedure from thiolactam **24** and 2-bromo-1-(4-methoxyphenyl)ethanone (341 mg, 1.49 mmol), with triethyl phosphite for the sulfide contraction. Enaminone **25b** (343 mg, 87%) was obtained as an amber oil; ^{1}H NMR (300 MHz, $CDCl_3$): δ = 7.79 (d, *J* = 8.8 Hz, 2H), 6.87 (d, *J* = 8.7 Hz, 2H), 5.56 (s, 1H), 4.26 (q, *J* = 7.1 Hz, 2H), 3.98 (s, 2H), 3.83 (s, 3H), 3.44 (t, *J* = 6.1 Hz, 2H), 3.32 (t, *J* = 6.5 Hz, 2H), 1.87 (quintet, *J* = 6.1 Hz, 2H), 1.73 (quintet, *J* = 6.4 Hz, 2H), 1.31 (t, *J* = 7.4 Hz, 3H); ^{13}C NMR (75 MHz, $CDCl_3$): δ = 186.6, 168.6, 163.7, 161.3, 135.0, 128.9, 113.0, 90.9, 61.2, 55.1, 54.2, 51.7, 27.9, 23.0, 19.3, 14.1; HRMS (ESI): *m*/*z* calcd for $C_{18}H_{24}NO_4^+$: 318.1700 [M + H]$^+$; found: 318.1716.

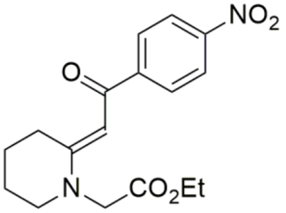

(*E*)-Ethyl 2-{2-[2-(4-nitrophenyl)-2-oxoethylidene]piperidin-1-yl}acetate (25c): Prepared according to the general procedure from thiolactam **24** and 2-bromo-1-(4-nitrophenyl)ethanone (364 mg, 1.49 mmol), with triethyl phosphite for the sulfide contraction. Enaminone **25c** (355 mg, 86%) was obtained as a yellow solid; m.p. 120-122 °C; ^{1}H NMR (300 MHz, $CDCl_3$): δ = 8.21 (d, *J* = 8.8 Hz, 2H), 7.89 (d, *J* = 8.8 Hz, 2H), 5.51 (s, 1H), 4.28 (q, *J* = 7.1 Hz, 2H), 4.05 (s, 2H), 3.50 (t, *J* = 6.1 Hz, 2H), 3.35 (t, *J* = 6.4 Hz, 2H), 1.91 (quintet, *J* = 5.8 Hz, 2H), 1.77 (quintet, *J* = 6.3 Hz, 2H), 1.31 (t, *J* = 7.1 Hz, 3H); ^{13}C NMR (75 MHz, $CDCl_3$): δ = 185.0, 168.2, 166.3, 148.6, 148.3, 128.0, 123.3, 91.0, 61.8, 54.5, 52.2, 28.5, 22.9, 19.1, 14.3; HRMS (ESI): *m*/*z* calcd for $C_{17}H_{21}N_2O_5^+$: 333.1445 [M + H]$^+$; found: 333.1458.

General method for the formation of ethyl 6-aryl-2,3-dihydro-1*H*-pyrrolizine-5-carboxylates (19): A mixture of enaminones **15** in xylene (5 mL/mmol) and flash silica gel (particle size 40–75 μm ; 500 wt % of starting material) in a capped microwave tube was irradiated under microwave conditions (150 W, 180 °C) for the stipulated time (see Table 2 in the main article) while maintaining moderate stirring. The reaction mixture was then transferred to a suitable flask with CH_2Cl_2, and the solvent was evaporated in vacuo. Further drying under vacuum provided the crude product adsorbed onto silica. (More silica was added as needed to provide a free-flowing, easily handled material). This mixture was then subjected to column chromatography with EtOAc–hexane mixtures as eluant to provide the corresponding ethyl 6-aryl-2,3-dihydro-1*H*-pyrrolizine-5-carboxylates **19**. The following products were prepared and characterized.

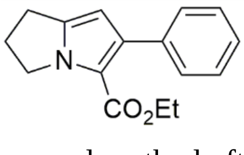

Ethyl 6-phenyl-2,3-dihydro-1*H*-pyrrolizine-5-carboxylate (19a): The product **19a** (87 mg, 93%) was obtained as an amber oil from (*E*)-ethyl 2-[2-(2-oxo-2-phenylethylidene)pyrrolidin-1-yl]acetate (**15a**, 100 mg, 0.37 mmol) according to the general method after 3.5 min of microwave irradiation; ^{1}H NMR (300 MHz, $CDCl_3$): δ = 7.50–7.43 (m, 2H), 7.38–7.24 (m, 3H), 5.95 (s, 1H), 4.33 (t, *J* = 7.2 Hz, 2H), 4.17 (q, *J* = 7.1 Hz, 2H), 2.88 (t, *J* = 7.5 Hz,

2H), 2.51 (quintet, J = 7.4 Hz, 2H), 1.17 (t, J = 7.1 Hz, 3H); ^{13}C NMR (75 MHz, $CDCl_3$): δ = 161.4, 142.3, 137.4, 136.7, 129.7, 127.4, 126.6, 114.3, 103.5, 59.5, 48.9, 26.8, 24.5, 14.2; IR (ATR): $\tilde{\nu}$ = 3065 (w), 2979 (w), 2902 (w), 1682 (s), 1461 (m), 1411 (m), 1360 (m), 1295 (m), 1281 (m), 1246 (s), 1177 (m), 1095 (s), 1037 (m), 797 (m), 758 (s), 696 (s) cm^{-1}; HRMS (EI): m/z calcd for $C_{16}H_{17}NO_2^+$: 255.1254 $[M]^+$; found: 255.1261.

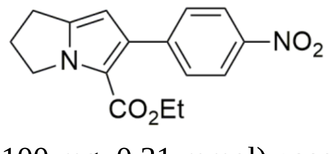

Ethyl 6-(4-nitrophenyl)-2,3-dihydro-1*H*-pyrrolizine-5-carboxylate (19b): The product **19b** (74 mg, 78%) was obtained as a bright yellow solid from (*E*)-ethyl 2-{2-[2-(4-nitrophenyl)-2-oxoethylidene]pyrrolidin-1-yl}acetate (**15b**, 100 mg, 0.31 mmol) according to the general method after 0.5 min of microwave irradiation; m.p. 75°C; ^{1}H NMR (300 MHz, $CDCl_3$): δ = 8.19 (d, J = 8.9 Hz, 2H), 7.62 (d, J = 8.9 Hz, 2H), 6.00 (s, 1H), 4.35 (t, J = 7.2 Hz, 2H), 4.21 (q, J = 7.1 Hz, 2H), 2.90 (t, J = 7.5 Hz, 2H), 2.54 (quintet, J = 7.4 Hz, 2H), 1.21 (t, J = 7.1 Hz, 3H); ^{13}C NMR (75 MHz, $CDCl_3$): δ = 161.0, 146.5, 143.8, 142.9, 134.7, 130.4, 122.9, 114.9, 103.7, 60.1, 49.2, 26.9, 24.6, 14.3; HRMS (ESI): m/z calcd for $C_{16}H_{17}N_2O_4^+$: 301.1183 $[M + H]^+$; found: 301.1178; m/z calcd for $C_{16}H_{16}N_2O_4Na^+$: 323.1002 $[M + Na]^+$; found: 323.0997.

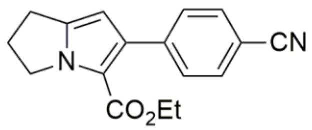

Ethyl 6-(4-cyanophenyl)-2,3-dihydro-1*H*-pyrrolizine-5-carboxylate (19c): The product **19c** (86 mg, 92%) was obtained as a white solid from (*E*)-ethyl 2-{2-[2-(4-cyanophenyl)-2-oxoethylidene]pyrrolidin-1-yl}acetate (**15c**, 100 mg, 0.34 mmol) according to the general method after 3 min of microwave irradiation; m.p. 83-85 °C; ^{1}H NMR (300 MHz, $CDCl_3$): δ = 7.61 (apparent d, J = 8.6 Hz, 2H), 7.56 (apparent d, J = 8.6 Hz, 2H), 5.96 (s, 1H), 4.34 (t, J = 7.2 Hz, 2H), 4.19 (q, J = 7.1 Hz, 2H), 2.89 (t, J = 7.5 Hz, 2H), 2.53 (quintet, J = 7.5 Hz, 2H), 1.20 (t, J = 7.1 Hz, 3H); ^{13}C NMR (75 MHz, $CDCl_3$): δ = 161.0, 142.8, 141.7, 135.2, 131.4, 130.4, 119.5, 114.8, 110.1, 103.6, 60.0, 49.2, 26.9, 24.6, 14.3; HRMS (ESI): m/z calcd for $C_{17}H_{17}N_2O_2^+$: 281.1285 $[M + H]^+$; found: 281.1291; m/z calcd for $C_{17}H_{16}N_2NaO_2^+$: 303.1104 $[M + Na]^+$; found: 303.1102.

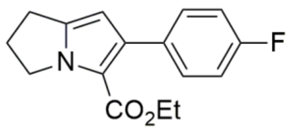

Ethyl 6-(4-fluorophenyl)-2,3-dihydro-1*H*-pyrrolizine-5-carboxylate (19d): The product **19d** (92 mg, 98%) was obtained as a colorless oil from (*E*)-ethyl 2-{2-[2-(4-fluorophenyl)-2-oxoethylidene]pyrrolidin-1-yl}acetate (**15d**, 100 mg, 0.34 mmol) according to the general method after 5 min of microwave irradiation; ^{1}H NMR (300 MHz, $CDCl_3$): δ = 7.54–7.38 (m, 2H), 7.14–6.85 (m, 2H), 5.91 (s, 1H), 4.31 (t, J = 7.2 Hz, 2H), 4.17 (q, J = 7.1 Hz, 2H), 2.86 (t, J = 7.5 Hz, 2H), 2.49 (quintet, J = 7.4 Hz, 2H), 1.18 (t, J = 7.1 Hz, 3H); ^{13}C NMR (75 MHz, $CDCl_3$): δ = 161.5 (d, J_{C-F} = 243.5 Hz), 161.3, 142.5, 136.4, 132.8 (d, J_{C-F} = 3.3 Hz), 131.3 (d, J_{C-F} = 7.9 Hz), 114.5, 114.3 (d, J_{C-F} = 21.4 Hz), 103.6, 59.7, 49.0, 26.8, 24.6, 14.3; HRMS (ESI): m/z calcd for $C_{16}H_{17}FNO_2^+$: 274.1238 $[M + H]^+$; found: 274.1252.

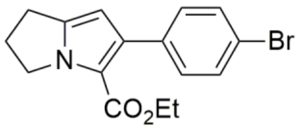

Ethyl 6-(4-bromophenyl)-2,3-dihydro-1*H*-pyrrolizine-5-carboxylate (19e): The product **19e** (92 mg, 98%) was obtained as an amber oil from (*E*)-ethyl 2-{2-[2-(4-bromophenyl)-2-oxoethylidene]pyrrolidin-1-yl}acetate (**15e**, 100 mg, 0.28 mmol) according to the general method after 4.5 min of microwave irradiation; ^{1}H NMR (300 MHz,

CDCl3): δ = 7.44 (apparent d, *J* = 8.2 Hz, 2H), 7.33 (apparent d, *J* = 8.4 Hz, 2H), 5.92 (s, 1H), 4.31 (t, *J* = 7.2 Hz, 2H), 4.18 (q, *J* = 7.1 Hz, 2H), 2.87 (t, *J* = 7.5 Hz, 2H), 2.50 (quintet, *J* = 7.4 Hz, 2H), 1.20 (d, *J* = 7.1 Hz, 3H); ^{13}C NMR (75 MHz, $CDCl_3$): δ = 161.3, 142.6, 136.1, 135.7, 131.4, 130.6, 120.7, 114.5, 103.5, 59.8, 49.1, 26.9, 24.6, 14.3; HRMS (ESI): *m/z* calcd for $C_{16}H_{17}BrNO_2^+$: 334.0437 $[M + H]^+$; found: 334.0429; *m/z* calcd for $C_{16}H_1BrNNa^+$: 356.0257 $[M + Na]^+$; found: 356.0256.

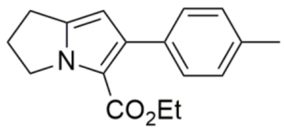

Ethyl 6-(4-methylphenyl)-2,3-dihydro-1*H*-pyrrolizine-5-carboxylate (19f): The product **19f** (84 mg, 89%) was obtained as a white crystalline solid from (*E*)-ethyl 2-{2-[2-(4-methylphenyl)-2-oxoethylidene]pyrrolidin-1-yl}acetate (**15f**, 100 mg, 0.35 mmol) according to the general method after 4.5 min of microwave irradiation; m.p. 73-74 °C; ^{1}H NMR (300 MHz, $CDCl_3$): δ = 7.37 (d, *J* = 8.1 Hz, 2H), 7.15 (d, *J* = 7.8 Hz, 2H), 5.94 (s, 1H), 4.32 (dd, *J* = 7.8, 6.6 Hz, 2H), 4.18 (q, *J* = 7.1 Hz, 2H), 2.87 (t, *J* = 7.5 Hz, 2H), 2.50 (quintet, *J* = 7.4 Hz, 2H), 2.37 (s, 3H), 1.20 (t, *J* = 7.1 Hz, 3H); ^{13}C NMR (75 MHz, $CDCl_3$): δ = 161.5, 142.4, 137.6, 136.3, 133.8, 129.6, 128.3, 114.4, 103.6, 59.6, 49.0, 26.9, 24.6, 21.3, 14.3; HRMS (ESI): *m/z* calcd for $C_{17}H_{20}NO_2^+$: 270.1489 $[M + H]^+$; found: 270.1508; *m/z* calcd for $C_{17}H_{19}NNaO_2^+$: 292.1308 $[M + Na]^+$; found: 292.1320.

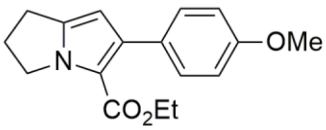

Ethyl 6-(4-methoxyphenyl)-2,3-dihydro-1*H*-pyrrolizine-5-carboxylate (19g): The product **19g** (91 mg, 96%) was obtained as a white crystalline solid from (*E*)-ethyl 2-{2-[2-(4-methoxyphenyl)-2-oxoethylidene]pyrrolidin-1-yl}acetate (**15g**, 101 mg, 0.33 mmol) according to the general method after 5 min of microwave irradiation; m.p. 71-72 °C; ^{1}H NMR (300 MHz, $CDCl_3$): δ = 7.41 (d, *J* = 8.8 Hz, 2H), 6.88 (d, *J* = 8.8 Hz, 2H), 5.92 (s, 1H), 4.31 (t, *J* = 7.2 Hz, 2H), 4.18 (q, *J* = 7.1 Hz, 2H), 3.82 (s, 3H), 2.86 (t, *J* = 7.5 Hz, 2H), 2.49 (quintet, *J* = 7.4 Hz, 2H), 1.20 (t, *J* = 7.1 Hz, 3H); ^{13}C NMR (75 MHz, $CDCl_3$): δ = 161.5, 158.6, 142.4, 137.3, 130.8, 129.2, 114.3, 113.0, 103.5, 59.6, 55.3, 49.0, 26.8, 24.6, 14.4; HRMS (ESI): calcd for $C_{17}H_{20}NO_3^+$: 286.1438 $[M + H]^+$; found: 286.1441; calcd for $C_{17}H_{19}NNaO_3^+$: 308.1257 $[M + Na]^+$; found: 308.1261.

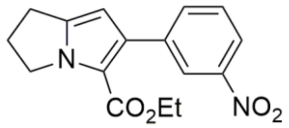

Ethyl 6-(3-nitrophenyl)-2,3-dihydro-1*H*-pyrrolizine-5-carboxylate (19h): The product **19h** (73 mg, 77%) was obtained as a bright yellow solid from (*E*)-ethyl 2-{2-[2-(3-nitrophenyl)-2-oxoethylidene]pyrrolidin-1-yl}acetate (**15h**, 100 mg, 0.31 mmol) according to the general method after 3.5 min of microwave irradiation; m.p. 109 - 110 °C; ^{1}H NMR (300 MHz, $CDCl_3$): δ = 8.35 (apparent t, *J* = 1.8 Hz, 1H), 8.10 (ddd, *J* = 8.2, 2.3, 1.0 Hz, 1H), 7.79 (ddd, *J* = 7.8, 1.5, 1.2 Hz, 1H), 7.47 (t, *J* = 8.0 Hz, 1H), 5.98 (s, 1H), 4.34 (t, *J* = 7.2 Hz, 2H), 4.18 (q, *J* = 7.1 Hz, 2H), 2.89 (t, *J* = 7.5 Hz, 2H), 2.52 (quintet, *J* = 7.4 Hz, 2H), 1.17 (t, *J* = 7.1 Hz, 3H); ^{13}C NMR (75 MHz, $CDCl_3$): δ = 161.0, 147.7, 142.8, 138.4, 135.8, 134.4, 128.3, 124.7, 121.4, 114.7, 103.5, 59.9, 49.1, 26.8, 24.5, 14.1; HRMS (ESI): *m/z* calcd for $C_{16}H_{17}N_2O_4^+$: 301.1183 $[M + H]^+$; found: 301.1177.

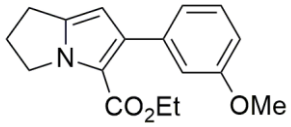

Ethyl 6-(3-methoxyphenyl)-2,3-dihydro-1*H*-pyrrolizine-5-carboxylate (19i): The product **19i** (103 mg, 81% over 2 steps based on thiolactam **18**) was obtained as colorless gel from a portion of the above impure sample of (*E*)-ethyl 2-{2-[2-(3-methoxyphenyl)-2-oxoethylidene]pyrrolidin-1-yl}acetate (**15i**, 150 mg) according to the general method

after 3.5 min of microwave irradiation; ^{1}H NMR (400 MHz, $CDCl_3$): δ = 7.28–7.20 (m, 1H), 7.12–6.94 (m, 2H), 6.83 (dd, *J* = 8.3, 2.6 Hz, 1H), 5.96 (s, 1H), 4.33 (t, *J* = 7.2 Hz, 2H), 4.18 (q, *J* = 7.1 Hz, 2H), 3.82 (s, 3H), 2.88 (t, *J* = 7.5 Hz, 2H), 2.51 (quintet, *J* = 7.4 Hz, 2H), 1.18 (t, *J* = 7.1 Hz, 3H); ^{13}C NMR (101 MHz, $CDCl_3$z): δ = 161.5, 159.0, 142.4, 138.2, 137.2, 128.5, 122.5, 115.5, 114.6, 112.4, 103.7, 59.7, 55.4, 49.0, 26.9, 24.6, 14.3; HRMS (ESI): *m*/*z* calcd for $C_{17}H_{20}NO_3^+$: 286.1438 [M + H]$^+$; found: 286.1436.

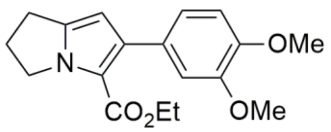

Ethyl 6-(3,4-dimethoxyphenyl)-2,3-dihydro-1*H*-pyrrolizine-5-carboxylate (19j): The product **19j** (105 mg, 89% over 2 steps based on thiolactam **18**) was obtained as an amber oil from a portion of the above impure sample of (*E*)-ethyl 2-{2-[2-(3,4-dimethoxyphenyl)-2-oxoethylidene]pyrrolidin-1-yl}acetate (**15j**, 124 mg) according to the general method after 5 min of microwave irradiation; ^{1}H NMR (400 MHz, $CDCl_3$): δ = 7.09–6.98 (m, 2H), 6.86 (d, *J* = 8.0 Hz, 1H), 5.95 (s, 1H), 4.33 (t, *J* = 7.2 Hz, 2H), 4.19 (q, *J* = 7.1 Hz, 2H), 3.90 (s, 3H), 3.89 (s, 3H), 2.88 (t, *J* = 7.5 Hz, 2H), 2.51 (quintet, *J* = 7.4 Hz, 2H), 1.20 (t, *J* = 7.1 Hz, 4H); ^{13}C NMR (101 MHz, $CDCl_3$): δ = 161.5, 148.12, 148.08, 142.4, 137.4, 129.6, 122.0, 114.4, 113.6, 110.6, 103.6, 59.6, 56.1, 56.0, 49.1, 26.9, 24.6, 14.5; HRMS (ESI): *m*/*z* calcd for $C_{18}H_{22}NO_4^+$: 316.1543 [M + H]$^+$; found: 316.1542.

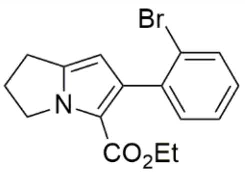

Ethyl 6-(2-bromophenyl)-2,3-dihydro-1*H*-pyrrolizine-5-carboxylate (19m): The product **19m** (64 mg, 46% over 2 steps based on thiolactam **18**) was obtained as colorless gel from a portion of the above impure sample of (*E*)-ethyl 2-{2-[2-(2-bromophenyl)-2-oxoethylidene]pyrrolidin-1-yl}acetate (**15m**, 150 mg) according to the general method after 9 min of microwave irradiation; ^{1}H NMR (300 MHz, $CDCl_3$): δ = 7.50 (dd, *J* = 7.7, 0.6 Hz, 1H), 7.26–7.13 (m, 2H), 7.04 (ddd, *J* = 8.1, 6.6, 2.4 Hz, 1H), 5.79 (s, 1H), 4.25 (t, *J* = 7.2 Hz, 2H), 3.98 (q, *J* = 7.1 Hz, 2H), 2.81 (t, *J* = 7.5 Hz, 2H), 2.44 (quintet, *J* = 7.5 Hz, 2H), 0.93 (t, *J* = 7.1 Hz, 3H); ^{13}C NMR (75 MHz, $CDCl_3$): δ = 161.1, 142.0, 138.5, 135.2, 132.1, 131.7, 128.2, 126.5, 124.4, 115.6, 103.5, 59.5, 48.5, 26.9, 24.6, 13.9; HRMS (ESI): *m*/*z* calcd for $C_{16}H_{17}BrNO_2^+$: 334.0437 [M + H]$^+$; found: 334.0437.

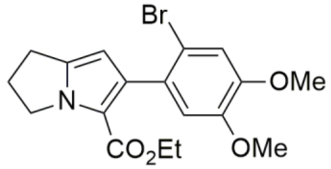

Ethyl 6-(2-bromo-4,5-dimethoxyphenyl)-2,3-dihydro-1*H*-pyrrolizine-5-carboxylate (19n): The product **19n** (34 mg, 35%) was obtained as a brown gel from (*E*)-ethyl 2-{2-[2-(2-bromo-4,5-dimethoxyphenyl)-2-oxoethylidene]pyrrolidin-1-yl}acetate (**15n**, 100 mg, 0.24 mmol) according to the general method after 9 min of microwave irradiation. The reaction mixture underwent significant concomitant decomposition even though reaction had not reached completion (TLC analysis); ^{1}H NMR (400 MHz, $CDCl_3$): δ = 7.07 (s, 1H), 6.83 (s, 1H), 5.89 (s, 1H), 4.34 (t, *J* = 7.2 Hz, 2H), 4.10 (q, *J* = 7.1 Hz, 2H), 3.89 (s, 3H), 3.83 (s, 3H), 2.90 (t, *J* = 7.5 Hz, 2H), 2.53 (quintet, *J* = 7.4 Hz, 2H), 1.08 (t, *J* = 7.1 Hz, 3H); ^{13}C NMR (101 MHz, $CDCl_3$): δ = 161.2, 148.5, 147.7, 142.0, 135.2, 130.7, 115.8, 115.1, 114.7, 114.5, 103.8, 59.6, 56.3, 56.1, 48.7, 27.0, 24.7, 14.2; HRMS (ESI): *m*/*z* calcd for $C_{18}H_{21}BrNO_4^+$: 394.0648 [M + H]$^+$; found: 394.0626; calcd for $C_{18}H_{20}BrNNaO_4^+$: 416.0468 [M + Na]$^+$; found: 416.0461.

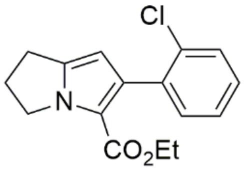

Ethyl 6-(2-chlorophenyl)-2,3-dihydro-1*H*-pyrrolizine-5-carboxylate (19o): The product **19o** (87 mg, 92%) was obtained as a white gel from (*E*)-ethyl 2-{2-[2-(2-chloro)-2-oxoethylidene]pyrrolidin-1-yl}acetate (**15o**, 100 mg, 0.33 mmol) according to the general method after 9 min of microwave irradiation; ^{1}H NMR (300 MHz, $CDCl_3$): δ = 7.43–7.36 (m, 1H), 7.33–7.26 (m, 1H), 7.26–7.17 (m, 2H), 5.90 (s, 1H), 4.34 (t, *J* = 7.2 Hz, 2H), 4.08 (q, *J* = 7.1 Hz, 2H), 2.90 (t, *J* = 7.5 Hz, 2H), 2.53 (quintet, *J* = 7.4 Hz, 2H), 1.02 (t, *J* = 7.1 Hz, 3H); ^{13}C NMR (75 MHz, $CDCl_3$): δ = 161.3, 142.2, 136.5, 134.0, 133.4, 132.0, 129.0, 128.1, 125.9, 115.8, 103.7, 59.6, 48.6, 27.0, 24.7, 14.0; HRMS (ESI): *m*/*z* calcd for $C_{16}H_{17}ClNO_2^+$: 290.0942 [M + H]$^+$; found: 290.0941. 290.0948.

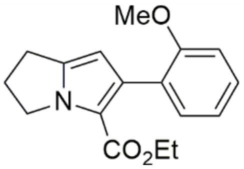

Ethyl 6-(2-methoxyphenyl)-2,3-dihydro-1*H*-pyrrolizine-5-carboxylate (19p): The product **19p** (103 mg, 82% over 2 steps based on thiolactam **18**) was obtained as an amber oil according to the general method from a portion of the above impure sample of (*E*)-ethyl 2-{2-[2-(2-methoxyphenyl)-2-oxoethylidene]pyrrolidin-1-yl}acetate (**15p**, 150 mg) according to the general method after 7 min of microwave irradiation; ^{1}H NMR (300 MHz, $CDCl_3$): δ = 7.30–7.20 (m, 2H), 7.00–6.79 (m, 2H), 5.92 (s, 1H), 4.31 (t, *J* = 7.2 Hz, 2H), 4.08 (q, *J* = 7.1 Hz, 2H), 3.77 (s, 3H), 2.87 (t, *J* = 7.5 Hz, 2H), 2.49 (quintet, *J* = 7.3 Hz, 2H), 1.05 (t, *J* = 7.1 Hz, 3H); ^{13}C NMR (75 MHz, $CDCl_3$): δ = 161.6, 157.1, 142.1, 132.4, 131.6, 128.2, 126.4, 120.0, 115.8, 110.4, 103.9, 59.4, 55.6, 48.6, 26.9, 24.7, 14.2; HRMS (ESI): *m*/*z* calcd for $C_{17}H_{20}NO_3^+$: 286.1438 [M + H]$^+$; found: 286.1440.

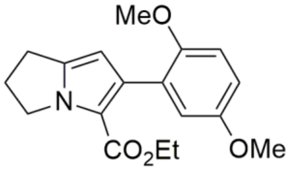

Ethyl 6-(2,5-dimethoxyphenyl)-2,3-dihydro-1*H*-pyrrolizine-5-carboxylate (19q): The product **19q** (87 mg, 82% over 2 steps based on thiolactam **18**) was obtained as an amber oil from a portion of the above impure sample of (*E*)-ethyl 2-{2-[2-(2,5-dimethoxyphenyl)-2-oxoethylidene]pyrrolidin-1-yl}acetate (**15q**, 100 mg) according to the general method after 7 min of microwave irradiation; ^{1}H NMR (300 MHz, $CDCl_3$): δ = 6.85 (d, *J* = 2.4 Hz, 1H), 6.83–6.78 (m, 2H), 5.92 (s, 1H), 4.31 (t, *J* = 7.2 Hz, 2H), 4.10 (q, *J* = 7.1 Hz, 2H), 3.77 (s, 3H), 3.72 (s, 3H), 2.87 (t, *J* = 7.5 Hz, 2H), 2.50 (quintet, *J* = 7.4 Hz, 2H), 1.07 (t, *J* = 7.1 Hz, 3H); ^{13}C NMR (75 MHz, $CDCl_3$): δ = 161.5, 153.0, 151.4, 142.0, 132.1, 127.3, 117.5, 115.8, 112.8, 111.4, 103.8, 59.4, 56.2, 55.8, 48.6, 26.9, 24.7, 14.2; HRMS (ESI): *m*/*z* calcd for $C_{18}H_{22}NO_4^+$: 316.1543 [M + H]$^+$; found: 316.1542; *m*/*z* calcd for $C_{18}H_{21}NNaO_4^+$: 338.1363 [M + Na]$^+$; found: 338.1372.

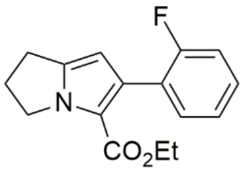

Ethyl 6-(2-fluorophenyl)-2,3-dihydro-1*H*-pyrrolizine-5-carboxylate (19r): The product **19r** (124 mg, 99% over 2 steps based on thiolactam **18**) was obtained as a pale pink solid from a portion of the above impure sample of (*E*)-ethyl 2-{2-[2-(2-fluorophenyl)-2-oxoethylidene]pyrrolidin-1-yl}acetate (**15r**, 150 mg) according to the general method after 1.5 min of microwave irradiation; m.p. 71-72 °C; ^{1}H NMR (300 MHz, $CDCl_3$): δ = 7.34 (td, *J* = 7.5, 1.8 Hz, 1H), 7.30–7.20 (m, 1H), 7.14–7.00 (m, 2H), 5.94 (s, 1H), 4.32 (t, *J* = 7.2 Hz, 2H), 4.13 (q, *J* = 7.1 Hz, 2H), 2.86 (t, *J* = 7.5 Hz, 2H), 2.49 (quintet, *J* = 7.3 Hz, 2H), 1.10 (t, *J* = 7.1 Hz, 3H);

^{13}C NMR (75 MHz, $CDCl_3$): δ = 161.2, 160.1 (d, J_{C-F} = 244.6 Hz), 142.3, 131.9 (d, J_{C-F} = 3.3 Hz), 129.4, 128.4 (d, J_{C-F} = 8.2 Hz), 125.0 (d, J_{C-F} = 15.6 Hz), 123.2 (d, J_{C-F} = 3.6 Hz), 115.7, 115.0 (d, J_{C-F} = 22.8 Hz), 103.7, 59.6, 48.7, 26.9, 24.6, 14.0; HRMS (ESI): *m*/*z* calcd for $C_{16}H_{17}FNO_2^+$: 274.1238 $[M + H]^+$; found: 274.1265.

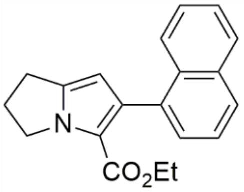

Ethyl 6-(naphthalen-1-yl)-2,3-dihydro-1*H*-pyrrolizine-5-carboxylate (19s): The product **19s** (106 mg, 81% over 2 steps based on thiolactam **18**) was obtained as an off-white solid from a portion of the above impure sample of (*E*)-ethyl 2-{2-[2-(naphthalen-1-yl)-2-oxoethylidene]pyrrolidin-1-yl}acetate (**15s**, 150 mg) according to the general method after 17 min of microwave irradiation; m.p. 136-138 °C; ^1H NMR (300 MHz, $CDCl_3$): δ = 7.95–7.71 (m, 3H), 7.50–7.30 (m, 4H), 5.99 (s, 1H), 4.39 (br q, *J* = 8.0 Hz, 2H), 3.83 (q, *J* = 7.1 Hz, 2H), 2.92 (t, *J* = 7.5 Hz, 2H), 2.55 (quintet, *J* = 7.3 Hz, 2H), 0.57 (t, *J* = 7.1 Hz, 3H); ^{13}C NMR (75 MHz, $CDCl_3$): δ = 161.5, 142.5, 135.6, 134.7, 133.4, 132.9, 128.0, 127.3, 127.1, 126.8, 125.4, 125.3, 125.0, 116.3, 104.7, 59.3, 48.6, 27.13, 24.7, 13.4; HRMS (ESI): *m*/*z* calcd for $C_{20}H_{20}NO_2^+$: 306.1489 $[M + H]^+$; found: 306.1492.

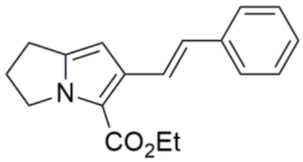

(*E*)-Ethyl 6-styryl-2,3-dihydro-1*H*-pyrrolizine-5-carboxylate (19t): The product **19t** (77 mg, 82%) was obtained as a white gel from (*E*)-ethyl 2-{2-[2-oxo-2-(styryl)ethylidene]pyrrolidin-1-yl}acetate (**15t**, 100 mg) according to the general method after 0.5 min of microwave irradiation; ^1H NMR (300 MHz, $CDCl_3$): δ = 7.86 (d, *J* = 16.4 Hz, 1H), 7.49 (d, *J* = 7.6 Hz, 2H), 7.32 (t, *J* = 7.5 Hz, 2H), 7.25–7.07 (m, 1H), 6.91 (d, *J* = 16.4 Hz, 1H), 6.24 (s, 1H), 4.34 (q, *J* = 7.1 Hz, 2H), 4.24 (t, *J* = 7.2 Hz, 2H), 2.82 (t, *J* = 7.5 Hz, 2H), 2.45 (quintet, *J* = 7.4 Hz, 2H), 1.41 (t, *J* = 7.1 Hz, 3H); ^{13}C NMR (75 MHz, $CDCl_3$): δ = 161.6, 143.3, 138.3, 134.3, 128.7, 128.1, 127.1, 126.4, 122.8, 115.8, 98.3, 59.9, 48.7, 26.8, 24.4, 14.7; HRMS (ESI): *m*/*z* calcd for $C_{18}H_{20}NO_2^+$: 282.1489 $[M + H]^+$; found: 282.1492. 282.1491.

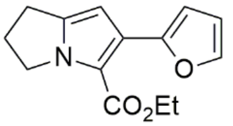

Ethyl 6-(furan-2-yl)-2,3-dihydro-1*H*-pyrrolizine-5-carboxylate (19u): The product **19u** (93 mg, ca. 100%) was obtained as a white crystalline solid from (*E*)-ethyl 2-{2-[2-(furan-2-yl)-2-oxoethylidene]pyrrolidin-1-yl}acetate (**15u**, 100 mg, 0.38 mmol) according to the general method after 5.5 min of microwave irradiation; m.p. 93 °C; ^1H NMR (300 MHz, $CDCl_3$): δ = 7.40 (dd, *J* = 1.8, 0.8 Hz, 1H), 7.05 (dd, *J* = 3.4, 0.8 Hz, 1H), 6.44 (dd, *J* = 3.4, 1.8 Hz, 1H), 6.29 (s, 1H), 4.32 and 4.30 (overlapping q, *J* = 7.2 Hz, and t, *J* = 7.2 Hz, 4H), 2.86 (t, *J* = 7.5 Hz, 2H), 2.48 (quintet, *J* = 7.5 Hz, 2H), 1.37 (t, *J* = 7.1 Hz, 3H); ^{13}C NMR (101 MHz, $CDCl_3$): δ = 161.0, 149.9, 142.7, 141.0, 126.6, 113.7, 111.5, 108.9, 101.3, 60.0, 49.4, 26.9, 24.6, 14.6; HRMS (ESI): *m*/*z* calcd for $C_{14}H_{16}NNaO_3^+$: 268.0944 $[M + Na]^+$; found: 268.0964.

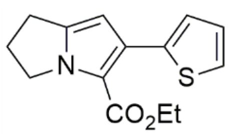

Ethyl 6-(thiophen-2-yl)-2,3-dihydro-1*H*-pyrrolizine-5-carboxylate (19v): The product **19v** (118 mg, 99% over 2 steps based on thiolactam **18**) was obtained as an amber oil from a portion of the above impure sample of (*E*)-ethyl 2-{2-[2-oxo-2-(thiophen-2-yl)ethylidene]pyrrolidin-1-yl}acetate (**15v**, 150 mg) according to the general method after 4.5

min of microwave irradiation; ^{1}H NMR (300 MHz, $CDCl_3$): δ = 7.37 (apparent d, *J* = 3.8 Hz, 1H), 7.23 (apparent d, *J* = 5.3 Hz, 1H), 7.06–6.93 (m, 1H), 6.08 (s, 1H), 4.36–4.21 (m, 4H), 2.84 (t, *J* = 7.5 Hz, 2H), 2.47 (quintet, *J* = 7.4 Hz, 2H), 1.30 (t, *J* = 7.1 Hz, 3H); ^{13}C NMR (75 MHz, $CDCl_3$): δ = 161.2, 142.4, 138.0, 129.2, 126.8, 126.7, 124.5, 114.5, 103.8, 59.9, 49.3, 26.7, 24.5, 14.4; HRMS (ESI): *m*/*z* calcd for $C_{14}H_{16}NO_2S^+$: 262.0896 [M + H]$^+$; found: 262.0896.

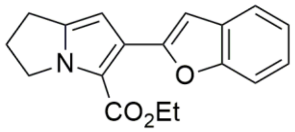

Ethyl 6-(benzofuran-2-yl)-2,3-dihydro-1*H*-pyrrolizine-5-carboxylate (19w): The product **19w** (90 mg, 96%) was obtained as a white solid from (*E*)-ethyl 2-{2-[2-(benzofuran-2-yl)-2-oxoethylidene]pyrrolidin-1-yl}acetate (**15w**, 100 mg, 0.32 mmol) according to the general method after 2.5 min of microwave irradiation; m.p. 106-107 °C); ^{1}H NMR (300 MHz, $CDCl_3$): δ = 7.55 (apparent d, *J* = 8.5 Hz, 1H), 7.52 (s, 1H), 7.45 (d, *J* = 7.8 Hz, 1H), 7.26–7.10 (m, 2H), 6.47 (s, 1H), 4.34 and 4.28 (overlapping q, *J* = 7.1 Hz, and t, *J* = 7.2 Hz, 4H), 2.84 (t, *J* = 7.5 Hz, 2H), 2.44 (quintet, *J* = 7.4 Hz, 2H), 1.36 (t, *J* = 7.1 Hz, 3H); ^{13}C NMR (75 MHz, $CDCl_3$): δ = 160.8, 154.2, 151.8, 142.7, 129.9, 126.0, 123.8, 122.5, 121.0, 114.8, 110.7, 105.1, 102.1, 60.1, 49.5, 26.8, 24.4, 14.6; HRMS (ESI): *m*/*z* calcd for $C_{18}H_{18}NO_3^+$: 296.1281 [M + H]$^+$; found: 296.1295.

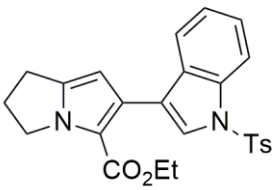

Ethyl 6-(1-tosyl-1*H*-indol-3-yl)-2,3-dihydro-1*H*-pyrrolizine-5-carboxylate (19x): The product **19x** (88 mg, 92%) was obtained as a white gel from (*E*)-ethyl 2-{2-[2-oxo-2-(1-tosyl-1*H*-indol-3-yl)ethylidene]pyrrolidin-1-yl}acetate (**15x**, 100 mg, 0.21 mmol) according to the general method after 2 min of microwave irradiation; ^{1}H NMR (300 MHz, $CDCl_3$): δ = 8.00 (d, *J* = 8.2 Hz, 1H), 7.78 (d, *J* = 8.3 Hz, 2H), 7.72 (s, 1H), 7.51 (d, *J* = 7.7 Hz, 1H), 7.28 (apparent td, *J* = 7.7, 1.2 Hz, 1H), 7.25–7.15 (m 3H), 6.05 (s, 1H), 4.35 (t, *J* = 7.2 Hz, 2H), 4.04 (q, *J* = 7.1 Hz, 2H), 2.88 (t, *J* = 7.5 Hz, 2H), 2.51 (quintet, *J* = 7.3 Hz, 2H), 2.30 (s, 3H), 0.86 (t, *J* = 7.1 Hz, 3H); ^{13}C NMR (75 MHz, $CDCl_3$): δ = 161.3, 144.8, 142.8, 135.6, 134.9, 131.3, 129.8, 127.0, 126.1, 125.0, 124.4, 123.1, 121.2, 118.6, 115.6, 113.6, 103.8, 59.7, 48.9, 26.9, 24.6, 21.6, 13.8; HRMS (ESI): *m*/*z* calcd for $C_{25}H_{25}N_2O_4S^+$: 449.1530 [M + H]$^+$; found: 449.1539.

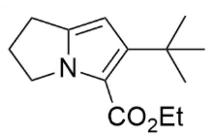

Ethyl 6-(*tert*-butyl)-2,3-dihydro-1*H*-pyrrolizine-5-carboxylate (19y): The product **19y** (75 mg, 81%) was obtained as an amber oil from (*E*)-ethyl 2-{2-[2-(*tert*-butyl)-2-oxoethylidene]pyrrolidin-1-yl}acetate (**15y**, 100 mg, 0.39 mmol) according to the general method after 19 min of microwave irradiation; ^{1}H NMR (300 MHz, $CDCl_3$): δ = 5.88 (s, 1H), 4.29 and 4.26 (superimposed q, *J* = 7.2 Hz, and br t, *J* = 7.2 Hz, 4H), 2.81 (t, *J* = 7.5 Hz, 2H), 2.42 (quintet, *J* = 7.4 Hz, 2H), 1.39 and 1.36 (superimposed s and t, *J* = 7.2 Hz, 12H); ^{13}C NMR (75 MHz, $CDCl_3$): δ = 161.2, 148.2, 140.9, 114.6, 100.8, 59.6, 50.0, 32.6, 30.6 (3 × C), 26.5, 24.7, 14.6; HRMS (ESI): *m*/*z* calcd for $C_{14}H_{22}NO_2^+$: 236.1645 [M + H]$^+$; found: 236.1640.

General method for the formation of ethyl 2-aryl-5,6,7,8-tetrahydroindolizine-3-carboxylates (26a-c): A mixture of enaminones **25a-c** dissolved in absolute EtOH (5 mL/mmol) and flash silica gel (5 × mass of starting material) in a capped microwave tube was irradiated under microwave conditions (50 W, 100 °C) for the stipulated time (see below) while maintaining moderate stirring. The reaction mixture was then transferred to a suitable flask with EtOH and the solvent was evaporated *in vacuo*. Further drying under vacuum provided the crude product adsorbed onto silica. (More silica was added as needed to provide a free-flowing, easily handled material). This mixture was then subjected to column chromatography with EtOAc–hexane mixtures as eluant to provide the corresponding ethyl 2-aryl-5,6,7,8-tetrahydroindolizine-3-carboxylates **26a-c**. The following three products were prepared and characterized.

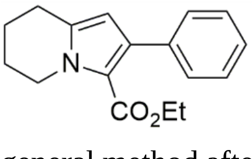

Ethyl 2-phenyl-5,6,7,8-tetrahydroindolizine-3-carboxylate (26a): The product **26a** (77 mg, 82%) was obtained as a colorless oil from (*E*)-ethyl 2-[2-(2-oxo-2-phenylethylidene)piperidin-1-yl]acetate (**25a**) (100 mg, 0.35 mmol) according to the general method after 1.5 h of microwave irradiation; ^{1}H NMR (300 MHz, $CDCl_3$): δ = 7.40 – 7.21 (m, 5H), 5.92 (s, 1H), 4.35 (t, *J* = 6.1 Hz, 2H), 4.08 (q, *J* = 7.1 Hz, 2H), 2.82 (t, *J* = 6.3 Hz, 2H), 2.03–1.93 (m, 2H), 1.88–1.77 (m, 2H), 1.03 (t, *J* = 7.1 Hz, 3H); ^{13}C NMR (75 MHz, $CDCl_3$): δ = 161.9, 137.4, 135.8, 133.9, 129.7, 127.4, 126.5, 117.5, 108.8, 59.5, 46.1, 24.1, 23.8, 20.2, 14.0; HRMS (ESI): *m*/*z* calcd for $C_{17}H_{20}NO_2^+$: 270.1489 $[M + H]^+$; found: 270.1483.

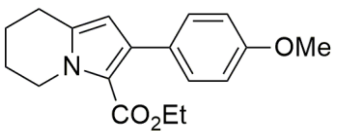

Ethyl 2-(4-methoxyphenyl)-5,6,7,8-tetrahydroindolizine-3-carboxylate (26b): The product **26b** (70 mg, 74%) was obtained as a colorless oil from (*E*)-ethyl 2{(2-[2-(4-methoxyphenyl)-2-oxoethylidene]piperidin-1-yl} acetate (**25b**) (100 mg, 0.32 mmol) according to the general method after 1 h of microwave irradiation; ^{1}H NMR (300 MHz, $CDCl_3$): δ = 7.23 (d, *J* = 8.7 Hz, 2H), 6.79 (d, *J* = 8.7 Hz, 2H), 5.81 (s, 1H), 4.26 (t, *J* = 6.1 Hz, 2H), 4.03 (q, *J* = 7.1 Hz, 2H), 3.74 (s, 3H), 2.73 (t, *J* = 6.3 Hz, 2H), 1.96–1.83 (m, 2H), 1.79–1.68 (m, 2H), 1.01 (t, *J* = 7.1 Hz, 3H); ^{13}C NMR (75 MHz, $CDCl_3$): δ = 161.9, 158.5, 135.8, 133.6, 130.8, 129.8, 117.3, 112.9, 108.8, 59.5, 55.4, 46.1, 24.1, 23.8, 20.2, 14.2; HRMS (ESI): *m*/*z* calcd for $C_{18}H_{22}NO_3^+$: 300.1594 $[M + H]^+$; found: 300.1587. 300.1594.

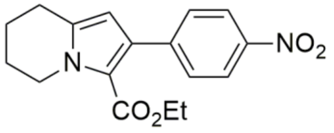

Ethyl 2-(4-nitrophenyl)-5,6,7,8-tetrahydroindolizine-3-carboxylate (26c): The product **26c** (61 mg, 65%) was obtained as a colorless oil from (*E*)-ethyl 2-{2-[2-(4-nitrophenyl)-2-oxoethylidene]piperidin-1-yl}acetate (**25c**) (100 mg, 0.30 mmol) according to the general method after 3 h of microwave irradiation; ^{1}H NMR (300 MHz, $CDCl_3$): δ = 8.17 (d, *J* = 8.9 Hz, 2H), 7.51 (d, *J* = 8.8 Hz, 2H), 5.95 (s, 1H), 4.36 (t, *J* = 6.1 Hz, 2H), 4.12 (q, *J* = 7.1 Hz, 2H), 2.83 (t, *J* = 6.3 Hz, 2H), 2.07–1.93 (m, 2H), 1.90–1.78 (m, 2H), 1.07 (t, *J* = 7.1 Hz, 3H); ^{13}C NMR (75 MHz, $CDCl_3$): δ = 161.3, 146.5, 144.6, 136.3, 131.3, 130.4, 122.8, 117.8, 108.8, 59.9, 46.3, 24.0, 23.6, 20.0, 14.1; HRMS (ESI): *m*/*z* calcd for $C_{17}H_{19}N_2O_4^+$: 315.1339 $[M + H]^+$; found: 315.1341.

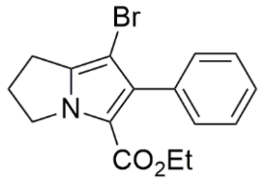

Ethyl 7-bromo-6-phenyl-2,3-dihydro-1*H*-pyrrolizine-5-carboxylate (28): *N*-Bromosuccinimide (338 mg, 1.90 mmol) in DMF (10 mL) was added dropwise to an ice-cold solution of ethyl 6-phenyl-2,3-dihydro-1*H*-pyrrolizine-5-carboxylate (**19a**) (323 mg, 1.27 mmol) in DMF (10 mL) under an atmosphere of Ar. The reaction mixture was stirred at 0 °C for 1 h, then warmed to room temperature and stirring was continued for 18 h. Water (20 mL) was added, and the mixture was extracted with Et_2O. The organic phase was separated, washed with water and saturated brine solution, dried (Na_2SO_4), filtered and evaporated *in vacuo*. The crude liquid obtained was purified by flash column chromatography (5% EtOAc/hexane) to afford the brominated dihydropyrrolizine **28** (354 mg, 83%) as a yellow solid; m.p. 75-77 °C; ^{1}H NMR (300 MHz, $CDCl_3$): δ = 7.35 (br s, 5H), 4.38 (t, *J* = 7.2 Hz, 2H), 4.09 (q, *J* = 7.1 Hz, 2H), 2.87 (t, *J* = 7.4 Hz, 2H), 2.52 (quintet, *J* = 7.4 Hz, 2H), 1.06 (t, *J* = 7.1 Hz, 3H); ^{13}C NMR (75 MHz, $CDCl_3$): δ = 160.8, 141.2, 135.1, 134.3, 130.6, 127.4, 127.3, 115.7, 91.4, 59.9, 50.0, 26.1, 24.4, 14.0; IR (ATR): $\tilde{\nu}$ = 3059 (w), 2980 (w), 2959 (w), 1684 (s), 1421 (m), 1365 (m), 1291 (m), 1254 (m), 1173 (m), 1113 (s), 773 (s), 700 (s), 672 (m) cm^{-1}; HRMS (EI): *m*/*z* calcd for $C_{16}H_{16}{}^{79}BrNO_2{}^+$: 333.0359 [M]$^+$; found: 333.0361.

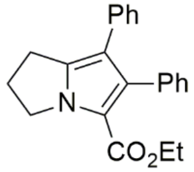

Ethyl 6,7-diphenyl-2,3-dihydro-1*H*-pyrrolizine-5-carboxylate (29): Phenylboronic acid (74 mg, 0.61 mmol) and tetrakis(triphenylphosphine)palladium(0) (76 mg, 0.066 mmol) were added to a solution of ethyl 7-bromo-6-phenyl-2,3-dihydro-1*H*-pyrrolizine-5-carboxylate (**28**) (160 mg, 0.48 mmol) in dry degassed DMF (7 mL) in an oven-dried 2-necked RB flask under an atmosphere of Ar. A degassed saturated aq. solution of Na_2CO_3 (170 mg, 1.60 mmol) in DMF (8 mL) was then added, and the mixture was heated at reflux for 20 h under an Ar atmosphere. On cooling, water was added to the vessel, and the mixture was extracted with Et_2O–EtOAc (2:1). The organic extract was washed with water, dried (Na_2SO_4), filtered and evaporated *vacuo*. The crude product was purified by flash column chromatography (10-30% EtOAc/hexane), and the material obtained was recrystallized from EtOH to give the dihydropyrrolizine **29** (130 mg, 82%) as a dark red cuboidal crystalline solid; m.p. 89-91 °C; ^{1}H NMR (300 MHz, $CDCl_3$): δ = 7.24 (s), 7.20-7.07 (m, 3H), 7.07-6.97 (m, 2H), 4.40 (t, *J* = 7.2 Hz, 2H), 4.10 (q, *J* = 7.1 Hz, 2H), 3.03 (t, *J* = 7.4 Hz, 2H), 2.55 (quintet, *J* = 7.5 Hz, 2H), 1.06 (t, *J* = 7.1 Hz, 3H); ^{13}C NMR (75 MHz, $CDCl_3$): δ = 161.6, 140.8, 135.9, 135.2, 134.2, 131.0, 128.7, 128.1, 127.5, 126.6, 125.4, 117.3, 115.8, 59.6, 48.9, 26.6, 25.1, 14.1; IR (ATR): $\tilde{\nu}$ = 3083 (w), 2971 (w), 2844 (w), 1676 (s), 1464 (w), 1419 (m), 1309 (m), 1220 (s), 1129 (m), 1093 (s), 1040 (m), 779 (m), 764 (m), 715 (s), 705 (s) cm^{-1}; HRMS (EI): *m*/*z* calcd for $C_{22}H_{21}NO_2{}^+$: 331.1567 [M]$^+$; found: 331.1574.

3. References

1. Fabbri, C.; Bietti, M.; Lanzalunga, O. *J. Org. Chem.* **2005**, *70*, 2720-2728.
2. Chapman, N. B.; Clarke, K.; Harvey, D. J. *J. Chem. Soc. (C)* **1971**, 1202-1206.
3. Thomé, I.; Besson, C.; Kleine, T.; Bolm, C. *Angew. Chem. Int. Ed.* **2013**, *52*, 7509-7513.
4. Low, D. W.; Pattison, G.; Wieczysty, M. D.; Churchill, G. H.; Lam, H. W. *Org. Lett.* **2012**, *14*, 2548-2551.
5. Li, X. C.; Ferreira, D.; Jacob, M. R.; Zhang, Q.; Khan, S. I.; ElSohly, H. N.; Nagle, D. G.; Smillie, T. J.; Khan, I. A.; Walker, L. A.; Clark, A. M. *J. Am. Chem. Soc.* **2004**, *126*, 6872-6873.
6. Laufer, S.; Striegel, H. G.; Neher, K.; Zechmeister, P.; Donat, C.; Stolingwa, K.; Baur, S.; Tries, S.; Kammermeier, T.; Dannhardt, G.; Kiefer, W. *Arch. Pharm. (Weinheim)* **1997**, *330*, 307-312.
7. Farag, A. M.; Hassaneen, H. M.; Abbas, I. M.; Shawali, A. S.; Algharib, M. S. *Phosphorus Sulfur Relat. Elem.* **1988**, *40*, 243-249.
8. Gharpure, S. J.; Anuradha, D.; Prasad, J. V. K.; Rao, P. S. *Eur. J. Org. Chem.* **2015**, *2015*, 86-90.
9. Dunn, N. L.; Ha, M.; Radosevich, A. T. *J. Am. Chem. Soc.* **2012**, *134*, 11330-11333.
10. Cioc, R. C.; Schaepkens van Riempst, L.; Schuckman, P.; Ruijter, E.; Orru, R. V. A. *Synthesis* **2017**, *49*, 1664-1674.
11. Chung, J. Y. L.; Hughes, D. L.; Zhao, D.; Song, Z.; Mathre, D. J.; Ho, G.-J.; McNamara, J. M.; Douglas, A. W.; Reamer, R. A.; Tsay, F.-R.; Varsolona, R.; McCauley, J.; Grabowski, E. J. J.; Reider, P. J. *J. Org. Chem.* **1996**, *61*, 215-222.

4. ^{1}H and ^{13}C NMR spectra

^{1}H NMR spectrum of ethyl 2-(2-oxopyrrolidin-1-yl)acetate (17) (300 MHz, $CDCl_3$)

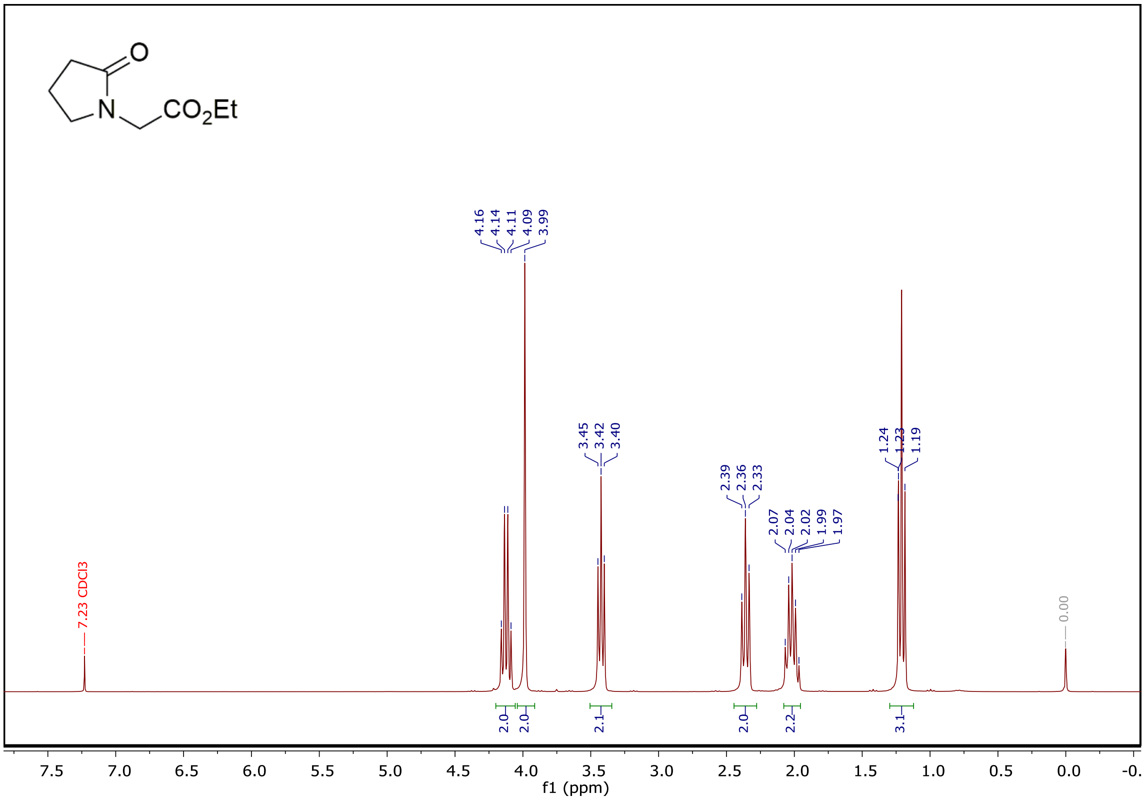

^{13}C NMR spectrum of ethyl 2-(2-oxopyrrolidin-1-yl)acetate (17) (75 MHz, $CDCl_3$)

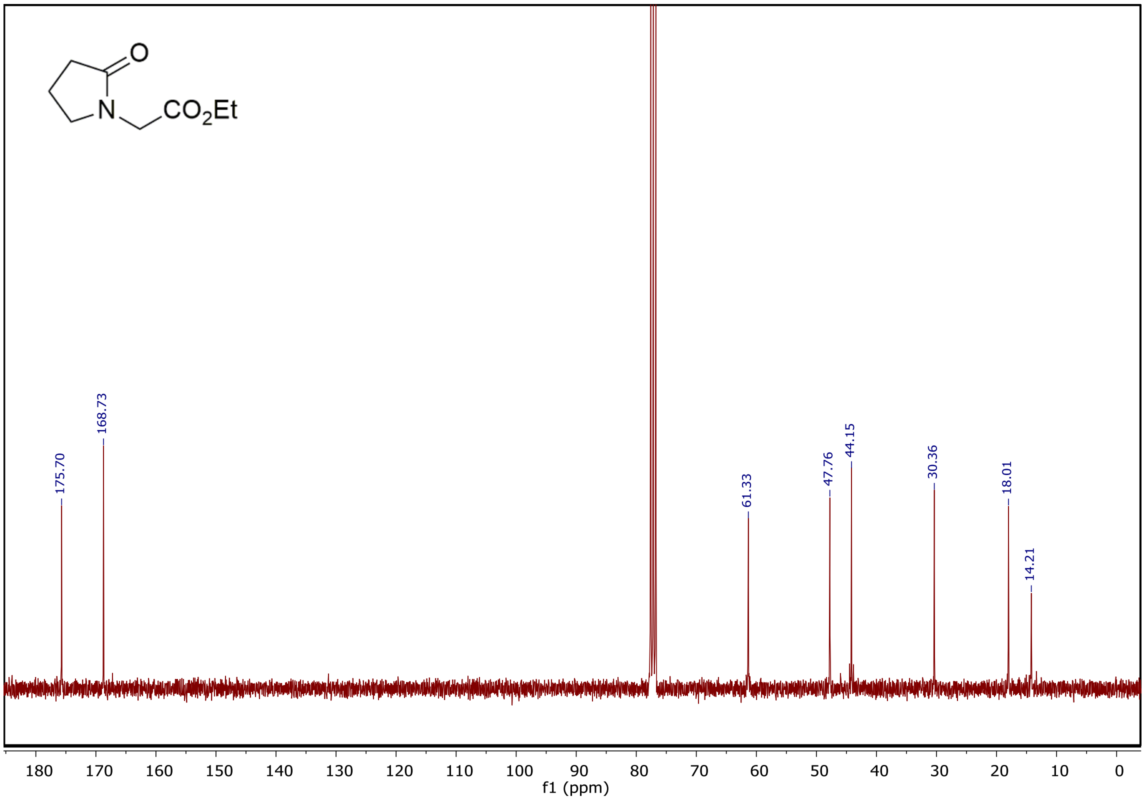

^{1}H NMR spectrum of ethyl 2-(2-oxopiperidin-1-yl)acetate (300 MHz, $CDCl_3$)

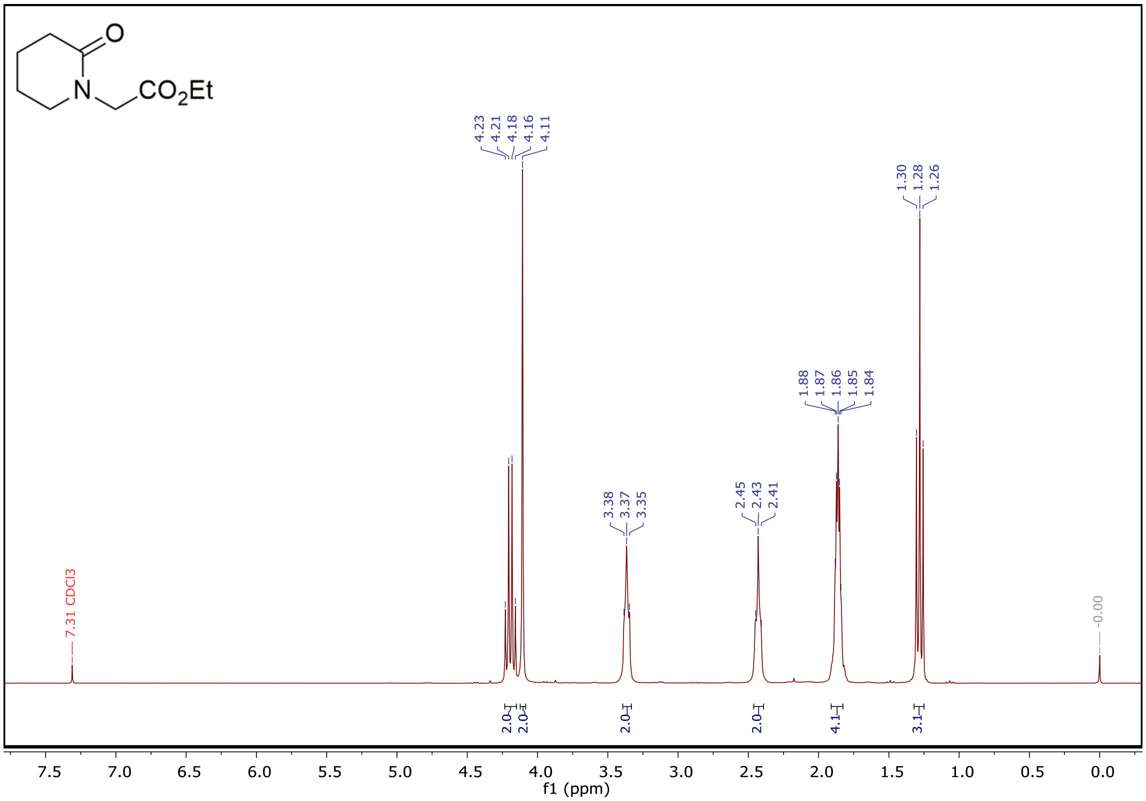

^{13}C NMR spectrum of ethyl 2-(2-oxopiperidin-1-yl)acetate (75 MHz, $CDCl_3$)

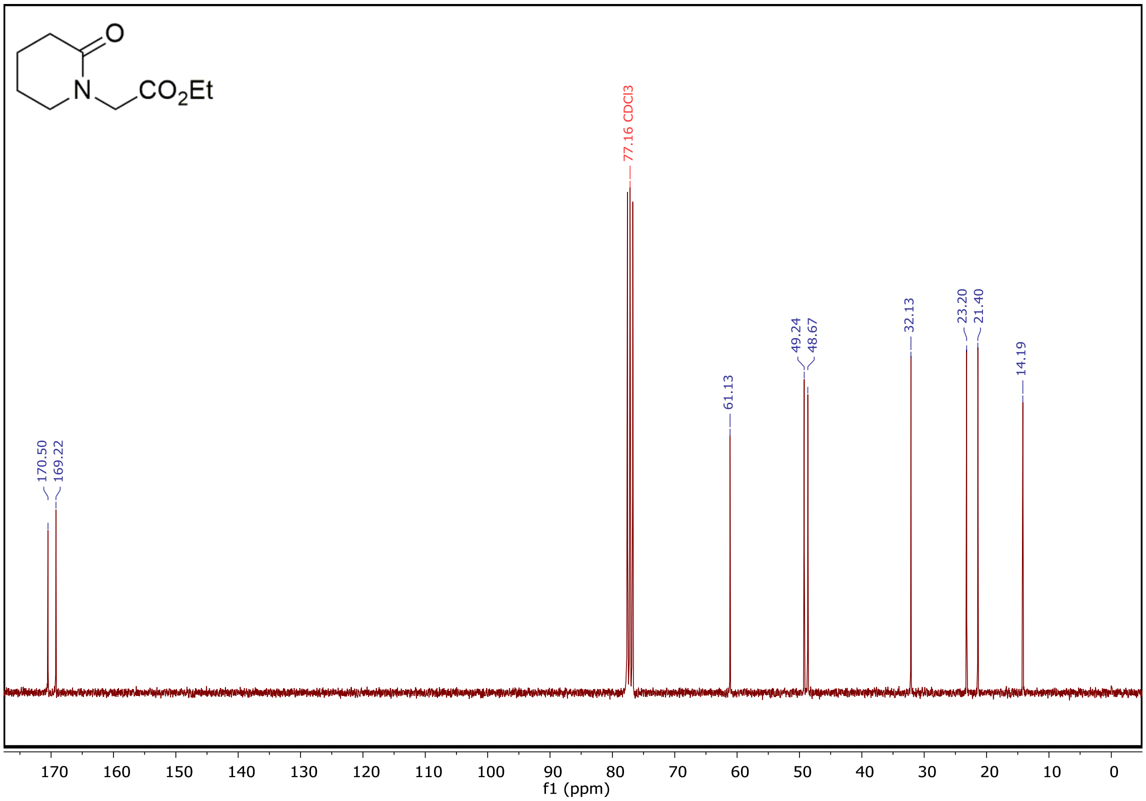

^{1}H NMR spectrum of ethyl 2-(2-thioxopyrrolidin-1-yl)acetate (18) (500 MHz, $CDCl_3$)

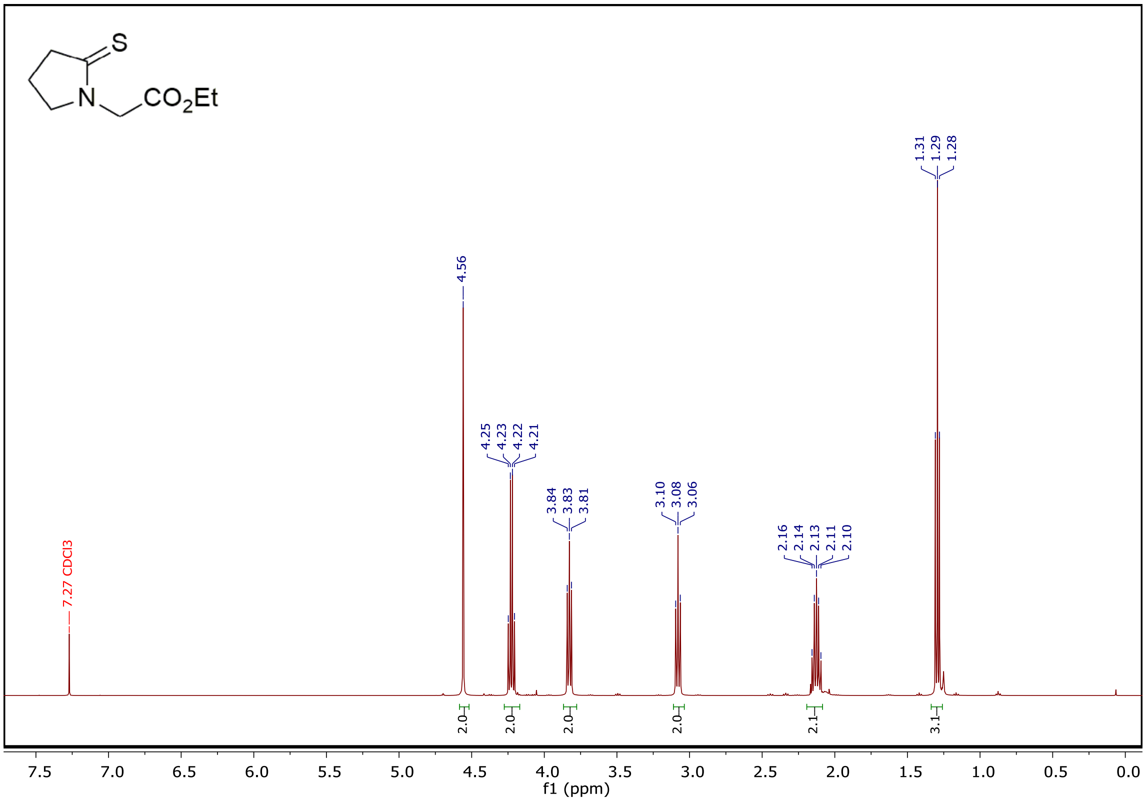

^{13}C NMR spectrum of ethyl 2-(2-thioxopyrrolidin-1-yl)acetate (18) (75 MHz, $CDCl_3$)

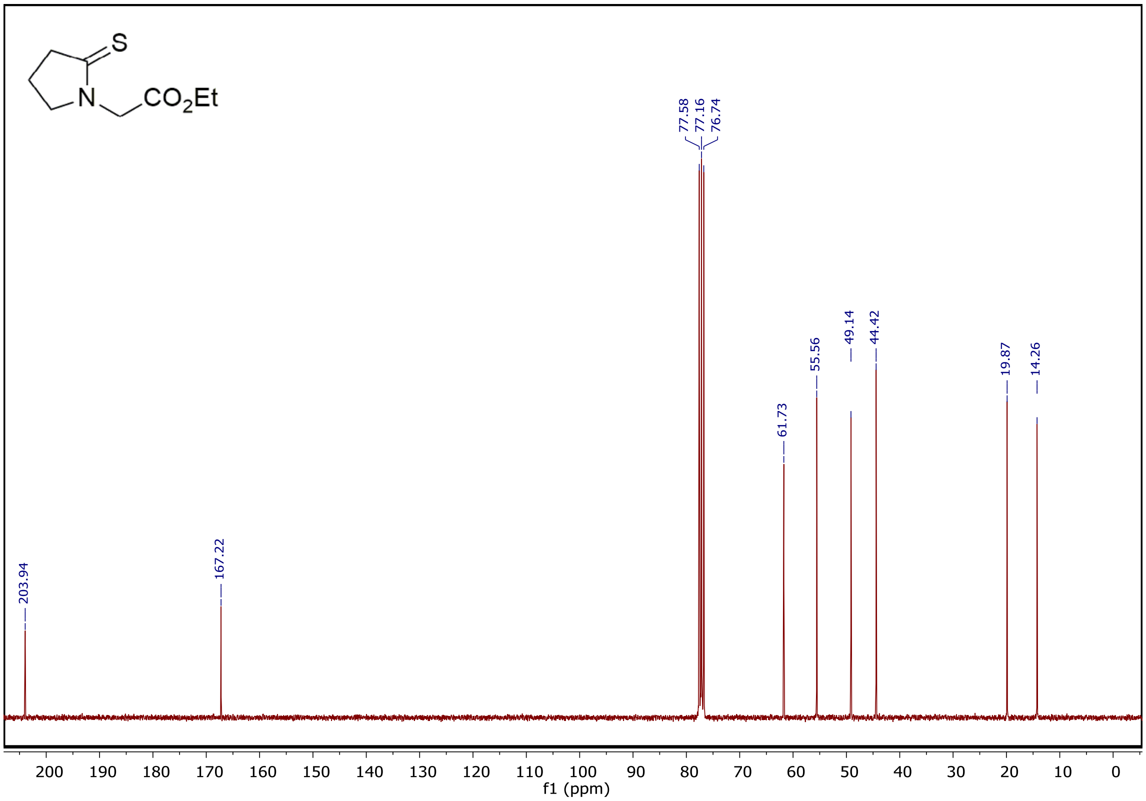

^{1}H NMR spectrum of ethyl 2-(2-thioxopiperidin-1-yl)acetate (24) (300 MHz, CDCl$_3$)

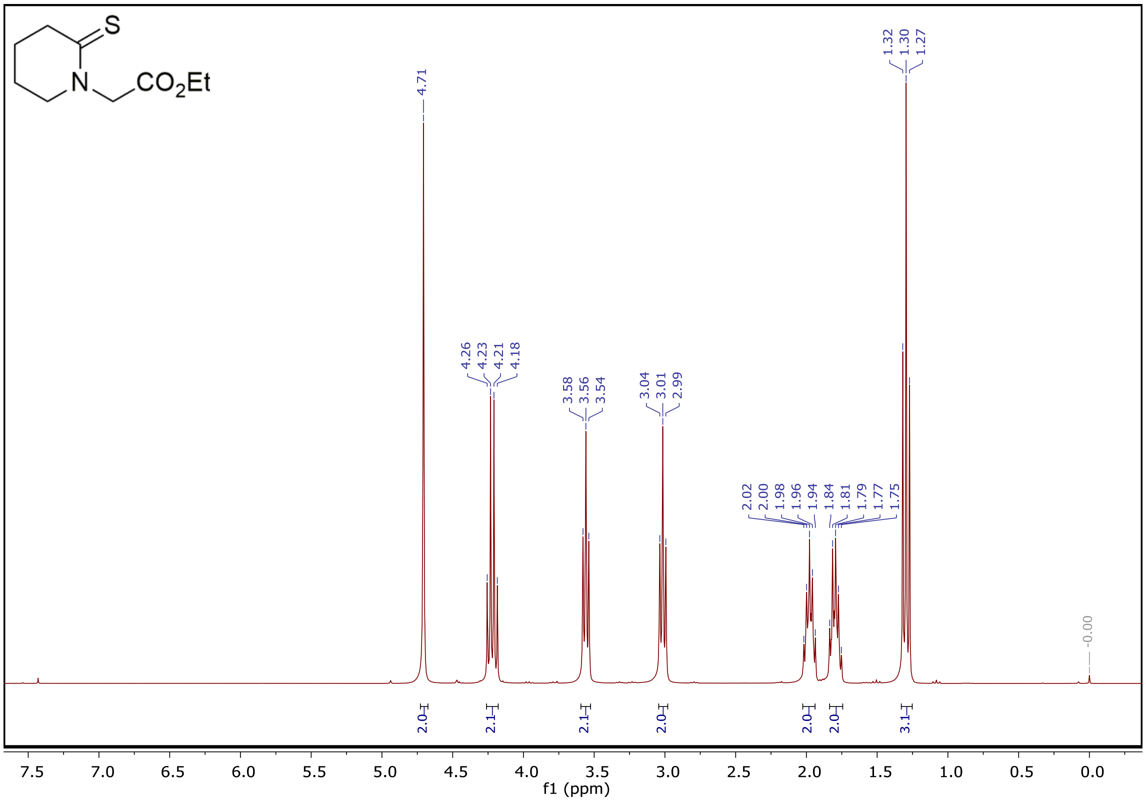

^{13}C NMR spectrum of ethyl 2-(2-thioxopiperidin-1-yl)acetate (24) (75 MHz, CDCl$_3$)

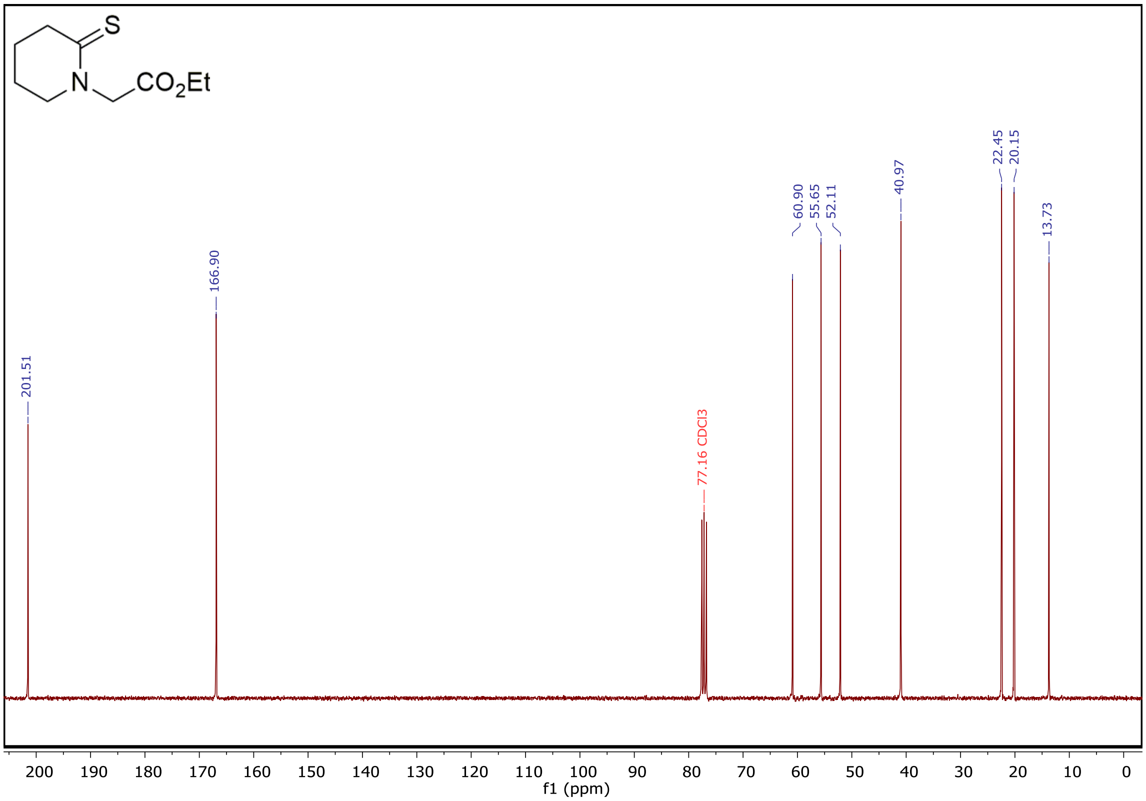

^{1}H NMR spectrum of (*E*)-ethyl 2-[2-(2-oxo-2-phenylethylidene)pyrrolidin-1-yl]acetate (15a) (300 MHz, $CDCl_3$)

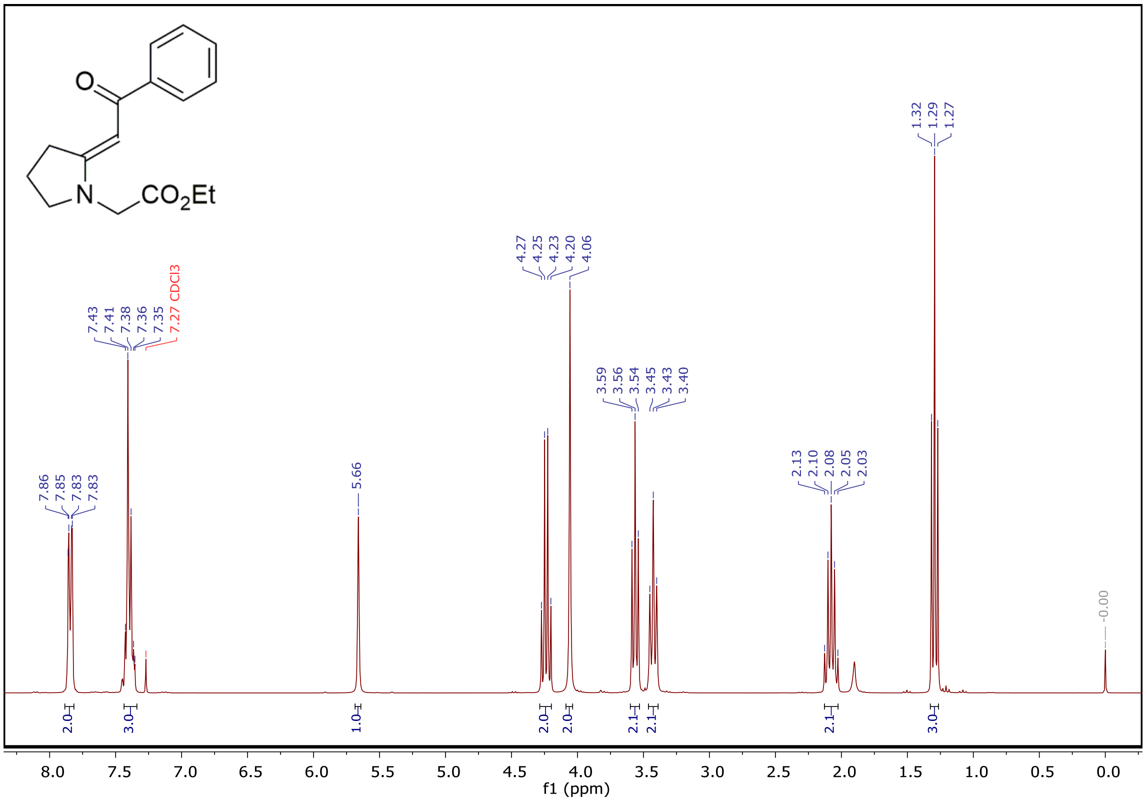

^{13}C NMR spectrum of (*E*)-ethyl 2-[2-(2-oxo-2-phenylethylidene)pyrrolidin-1-yl]acetate (15a) (75 MHz, $CDCl_3$)

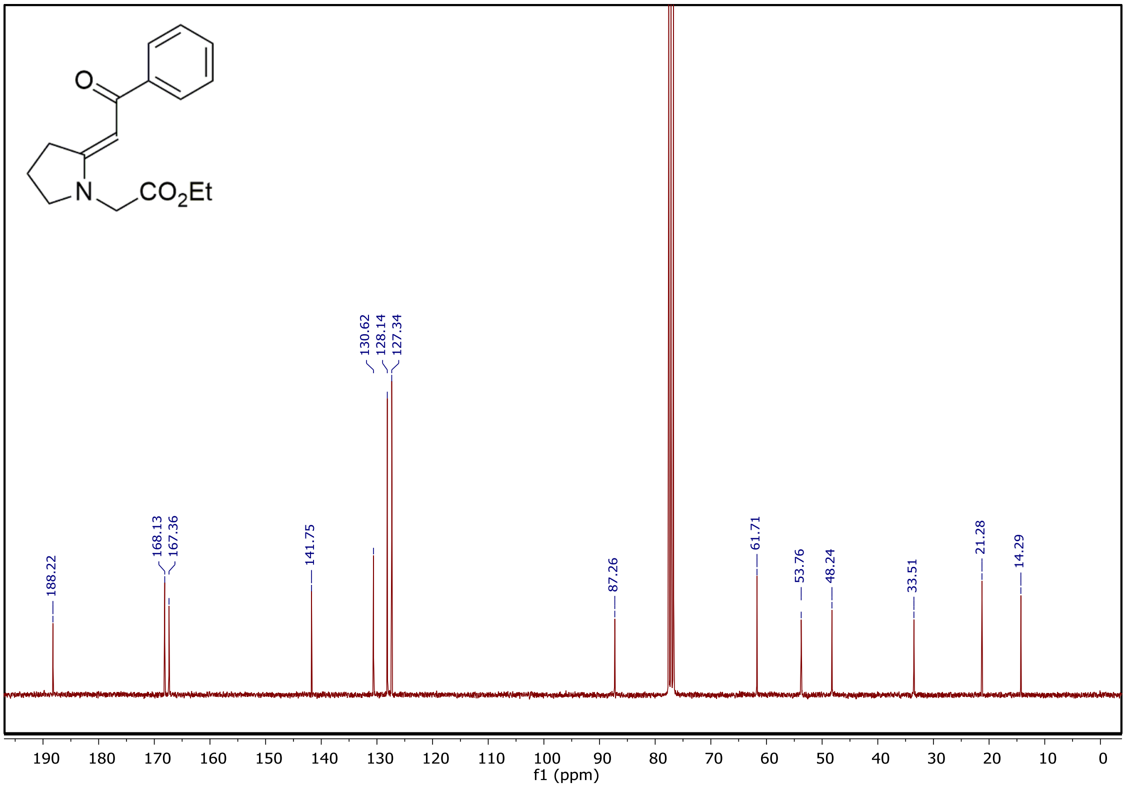

DEPT-135 spectrum of (*E*)-ethyl 2-[2-(2-oxo-2-phenylethylidene)pyrrolidin-1-yl]acetate (15a) (75 MHz, $CDCl_3$)

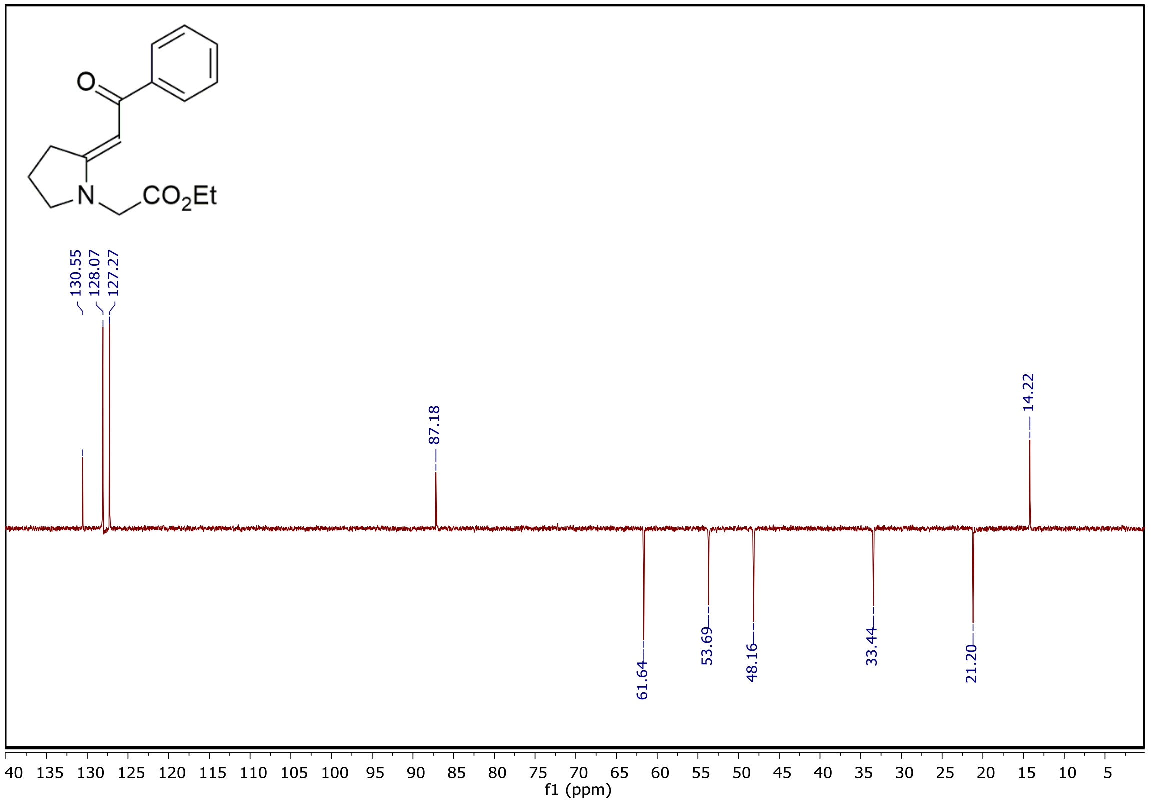

COSY NMR spectrum of (*E*)-ethyl 2-[2-(2-oxo-2-phenylethylidene)pyrrolidin-1-yl]acetate (15a) (300 MHz, $CDCl_3$)

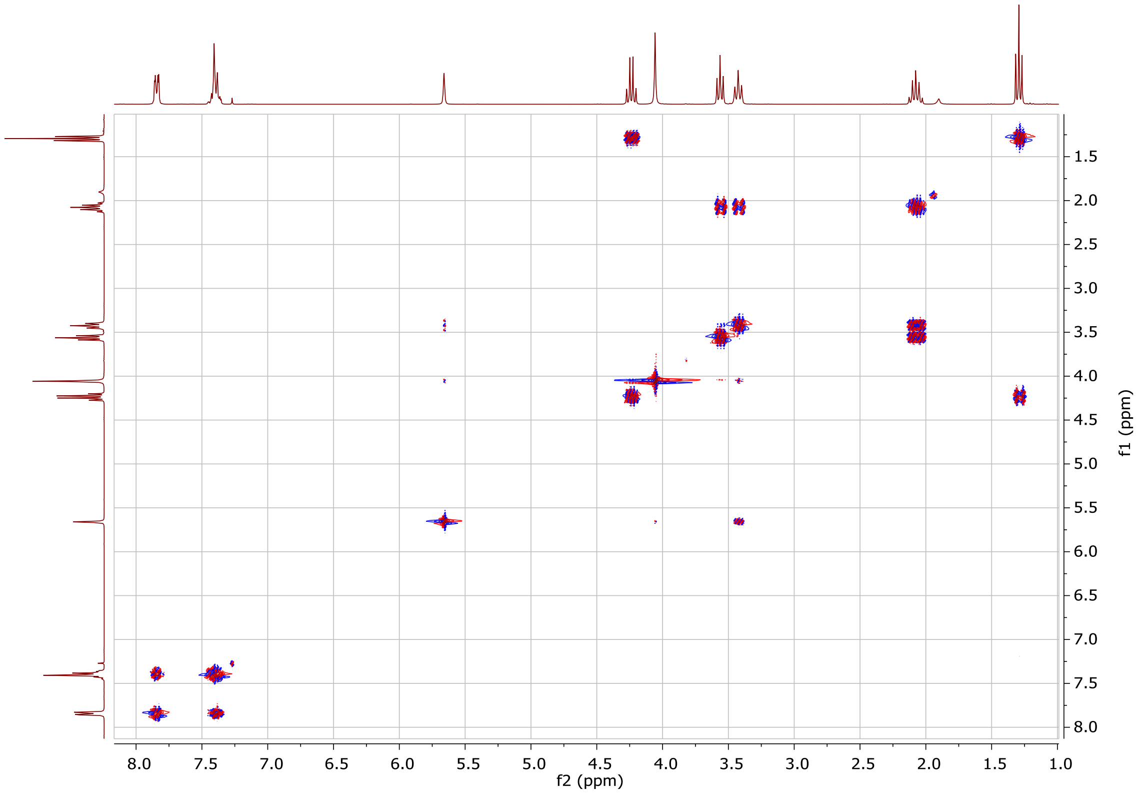

NOESY NMR spectrum of (*E*)-ethyl 2-[2-(2-oxo-2-phenylethylidene)pyrrolidin-1-yl]acetate (15a) (300 MHz, $CDCl_3$)

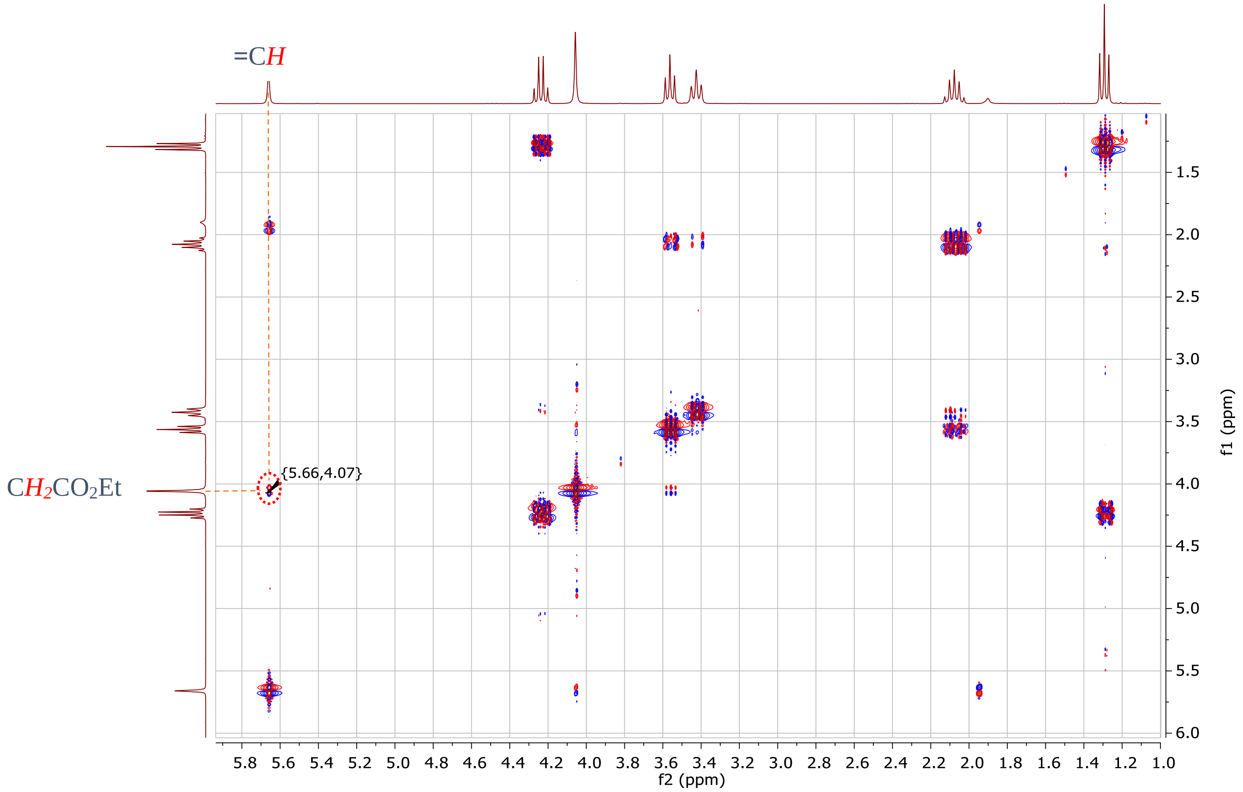

HSQC spectrum of (*E*)-ethyl 2-[2-(2-oxo-2-phenylethylidene)pyrrolidin-1-yl]acetate (15a) ($CDCl_3$)

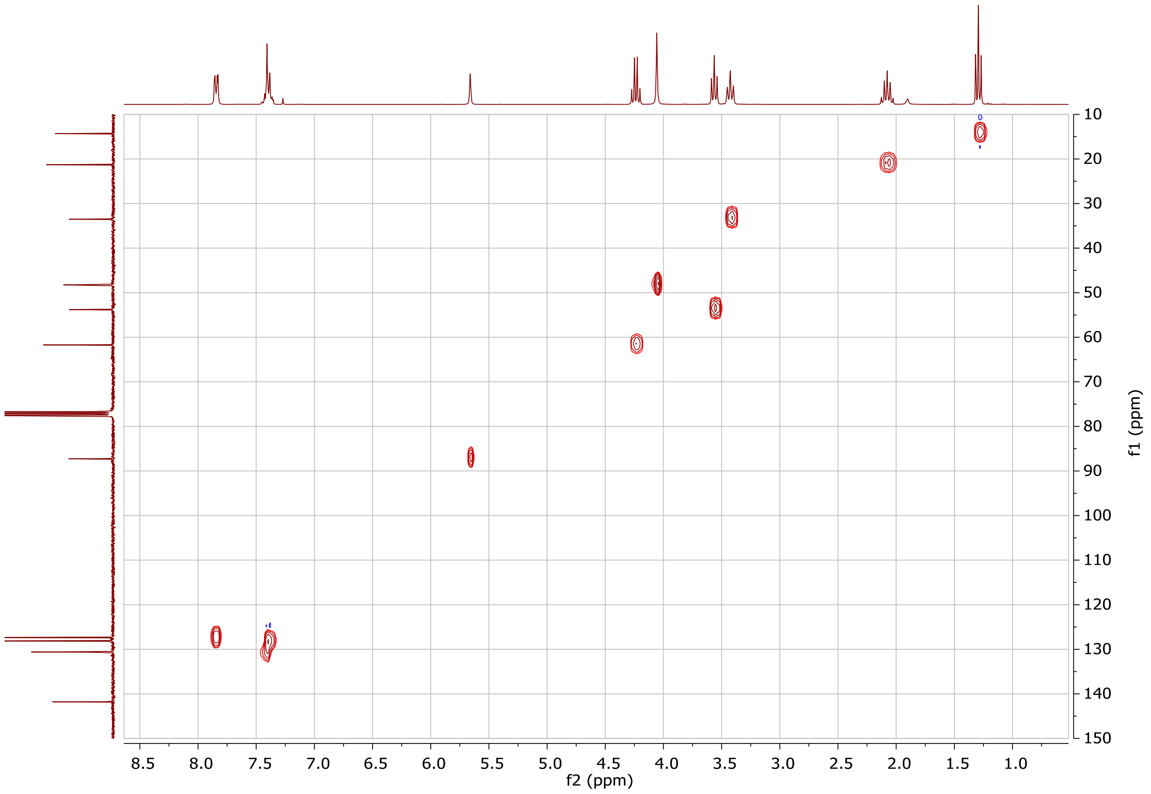

^{1}H NMR spectrum of (*E*)-ethyl 2-{2-[2-(4-nitrophenyl)-2-oxoethylidene]pyrrolidin-1-yl}acetate (15b) (300 MHz, $CDCl_3$)

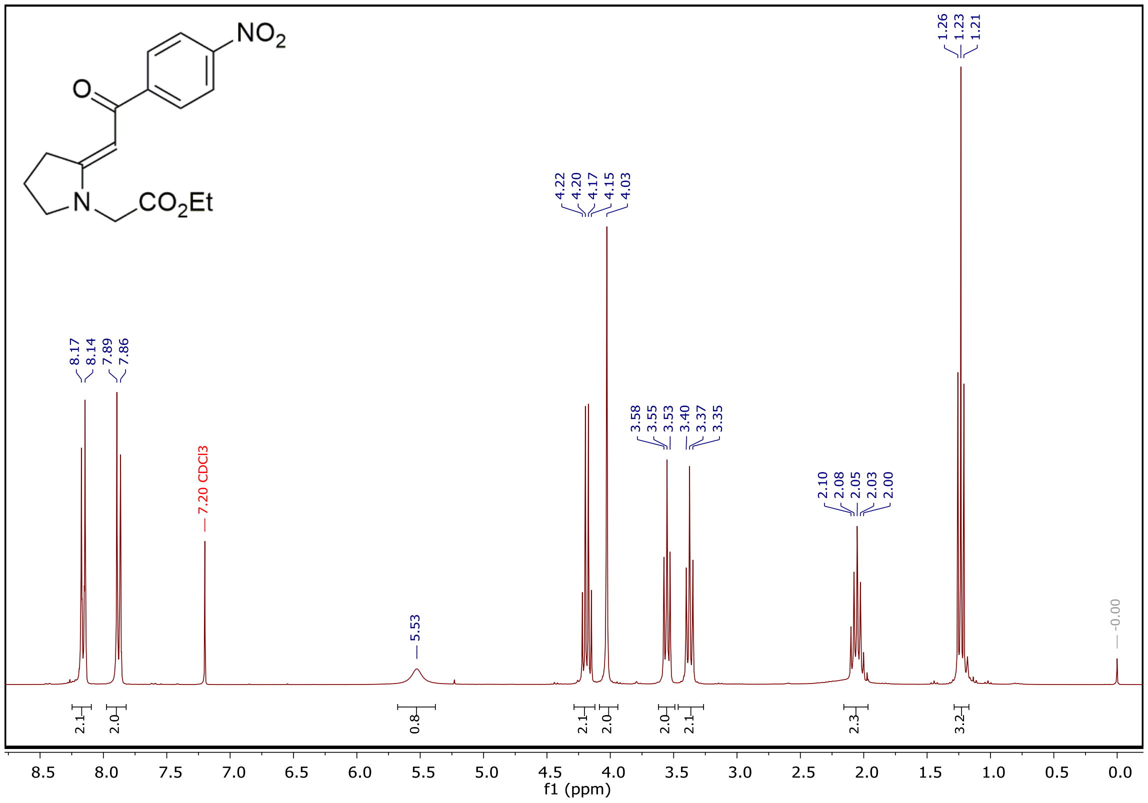

^{13}C NMR spectrum of (*E*)-ethyl 2-{2-[2-(4-nitrophenyl)-2-oxoethylidene]pyrrolidin-1-yl}acetate (15b) (75 MHz, $CDCl_3$)

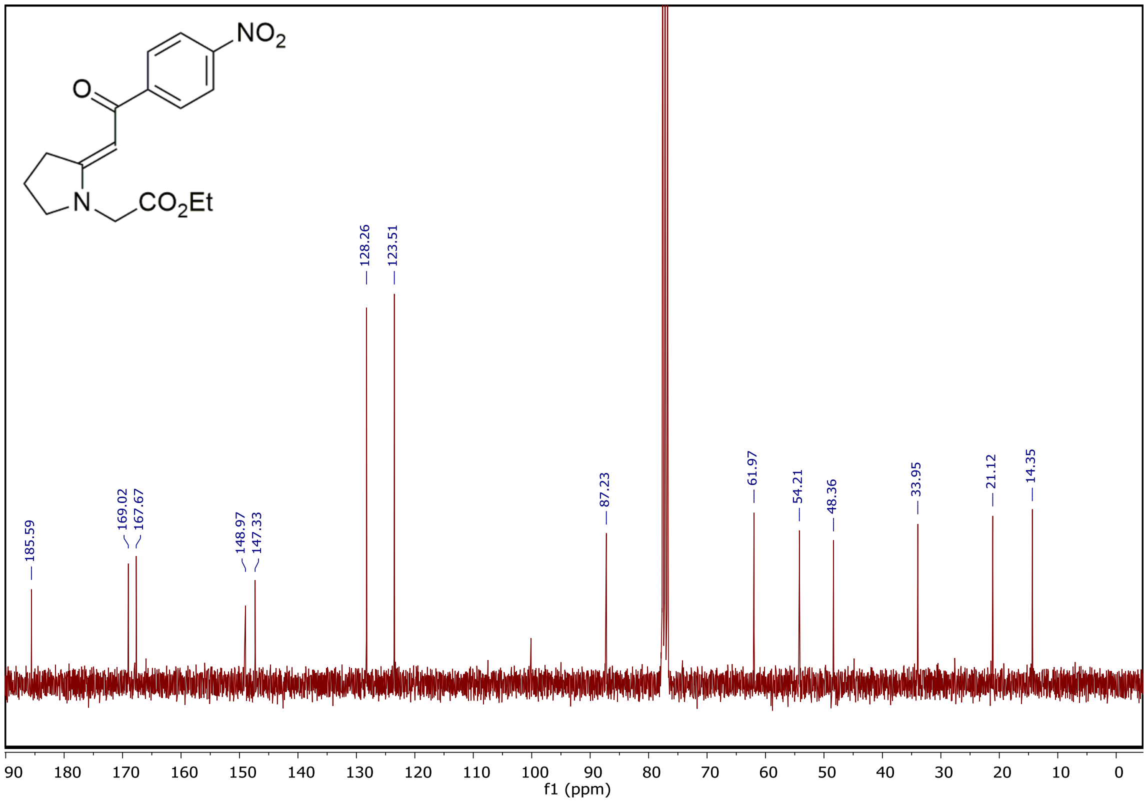

^{1}H NMR spectrum of (*E*)-ethyl 2-{2-[2-(4-cyanophenyl)-2-oxoethylidene]pyrrolidin-1-yl}acetate (15c) (300 MHz, $CDCl_3$)

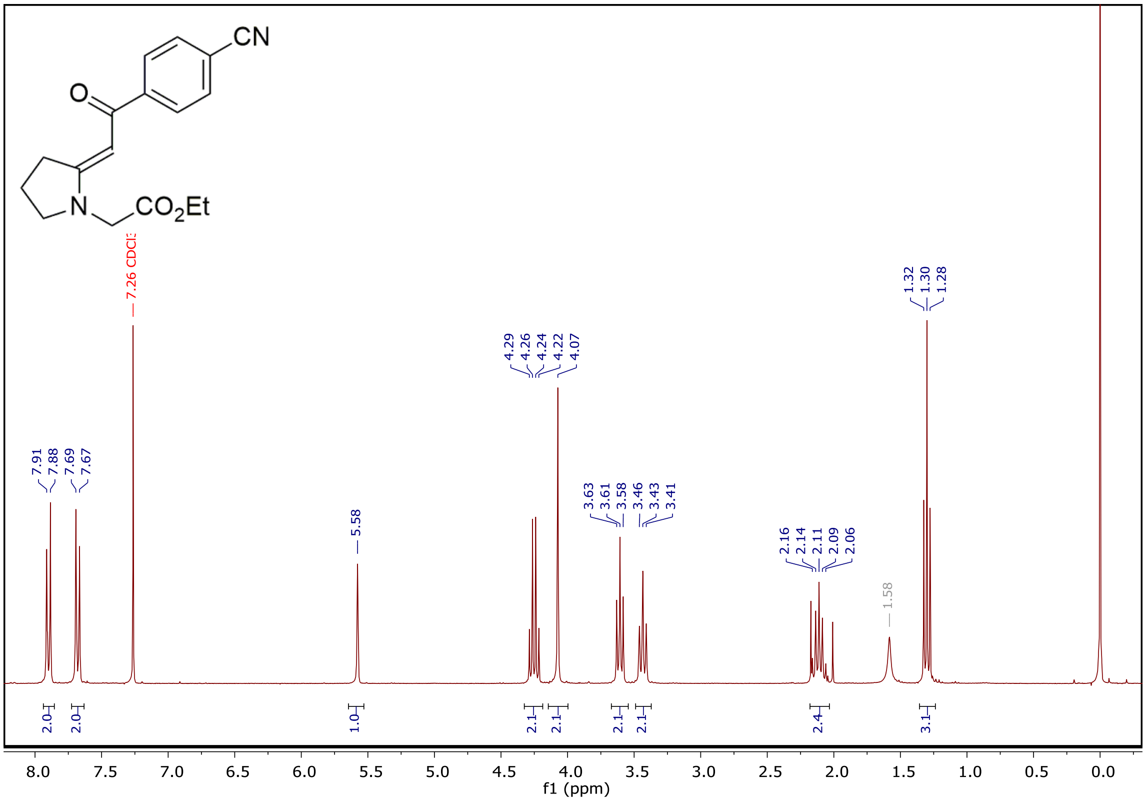

^{13}C NMR spectrum of (*E*)-ethyl 2-{2-[2-(4-cyanophenyl)-2-oxoethylidene]pyrrolidin-1-yl}acetate (15c) (75 MHz, $CDCl_3$)

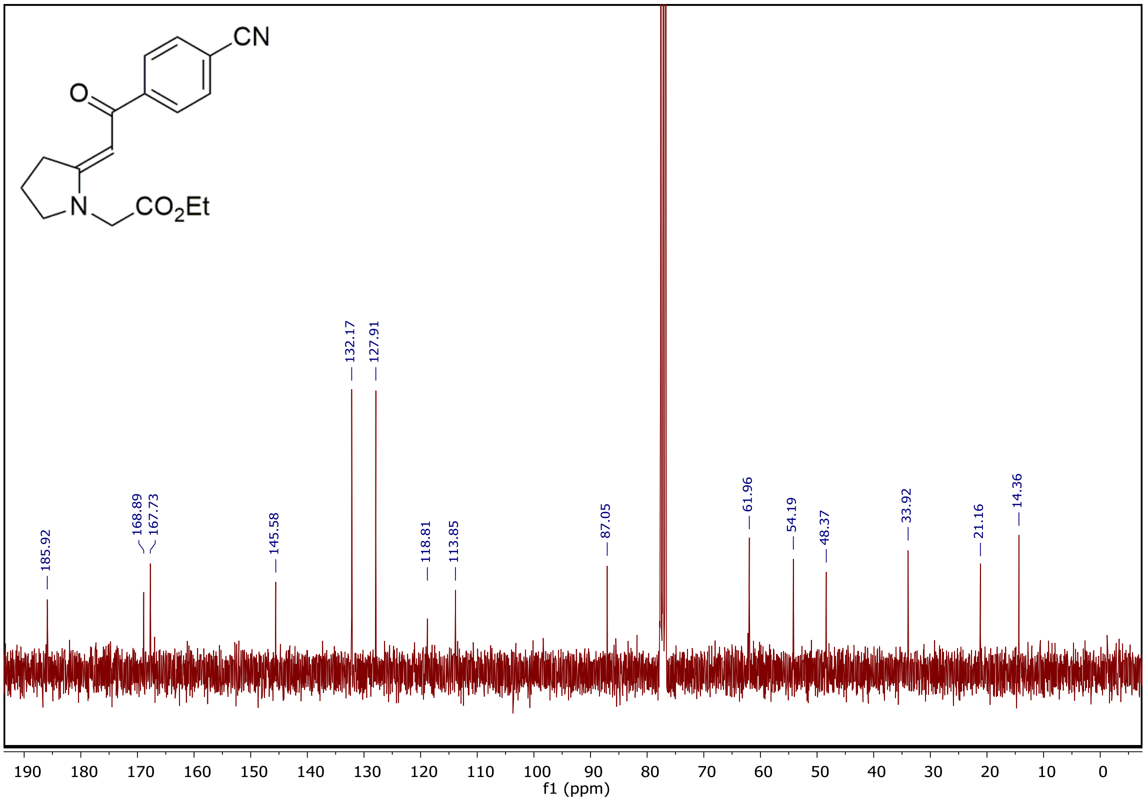

^{1}H NMR spectrum of (*E*)-ethyl 2-{2-[2-(4-fluorophenyl)-2-oxoethylidene]pyrrolidin-1-yl}acetate (15d) (300 MHz, $CDCl_3$)

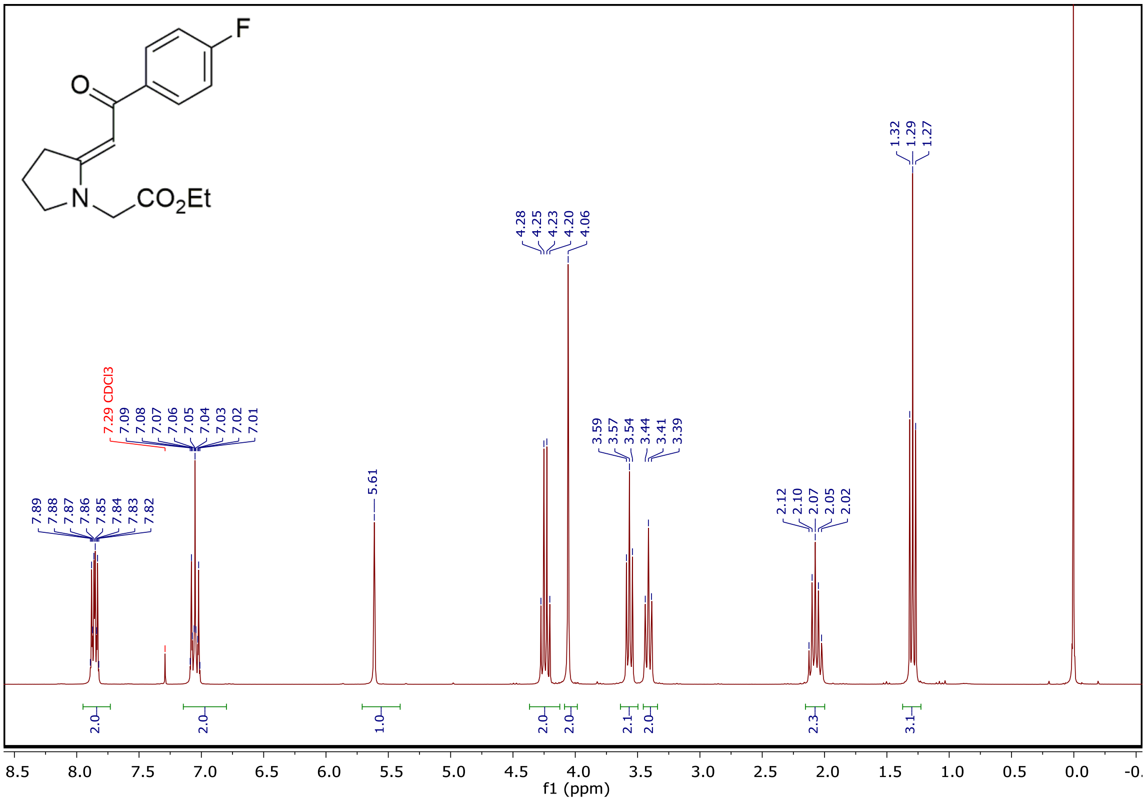

^{13}C NMR spectrum of (*E*)-ethyl 2-{2-[2-(4-fluorophenyl)-2-oxoethylidene]pyrrolidin-1-yl}acetate (15d) (75 MHz, $CDCl_3$)

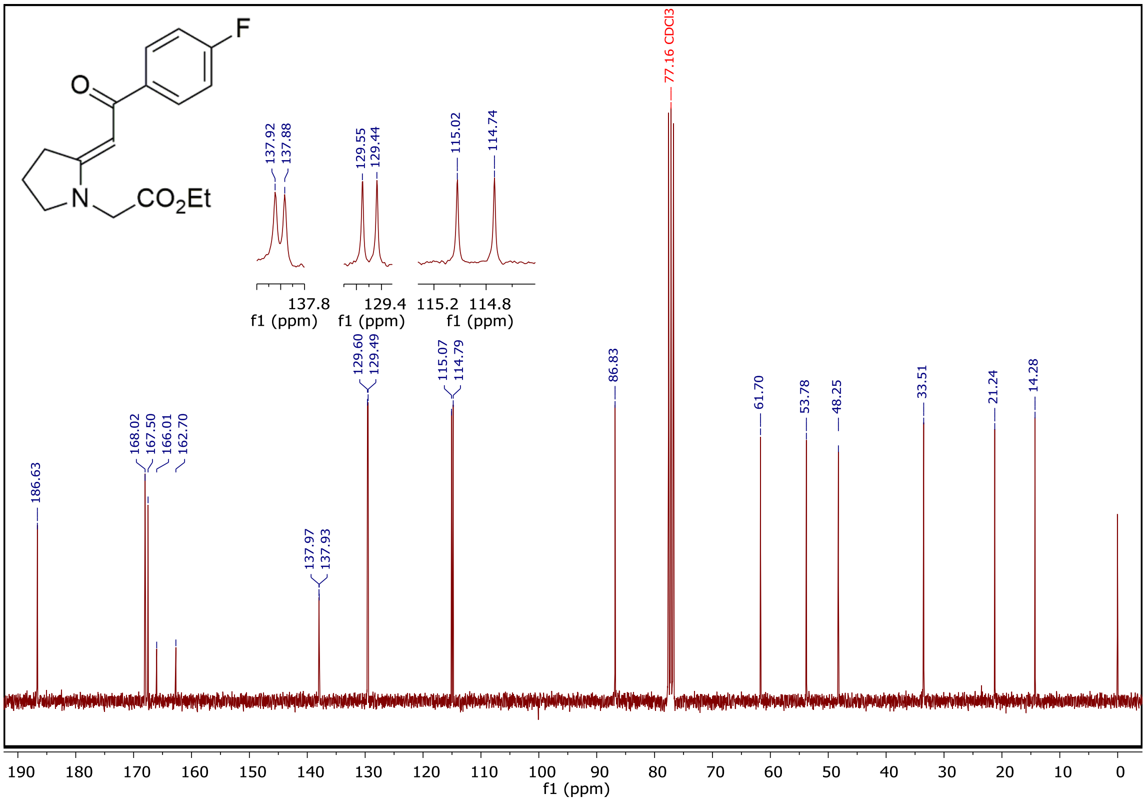

^{1}H NMR spectrum of (*E*)-ethyl 2-{2-[2-(4-bromophenyl)-2-oxoethylidene]pyrrolidin-1-yl}acetate (15e) (300 MHz, $CDCl_3$)

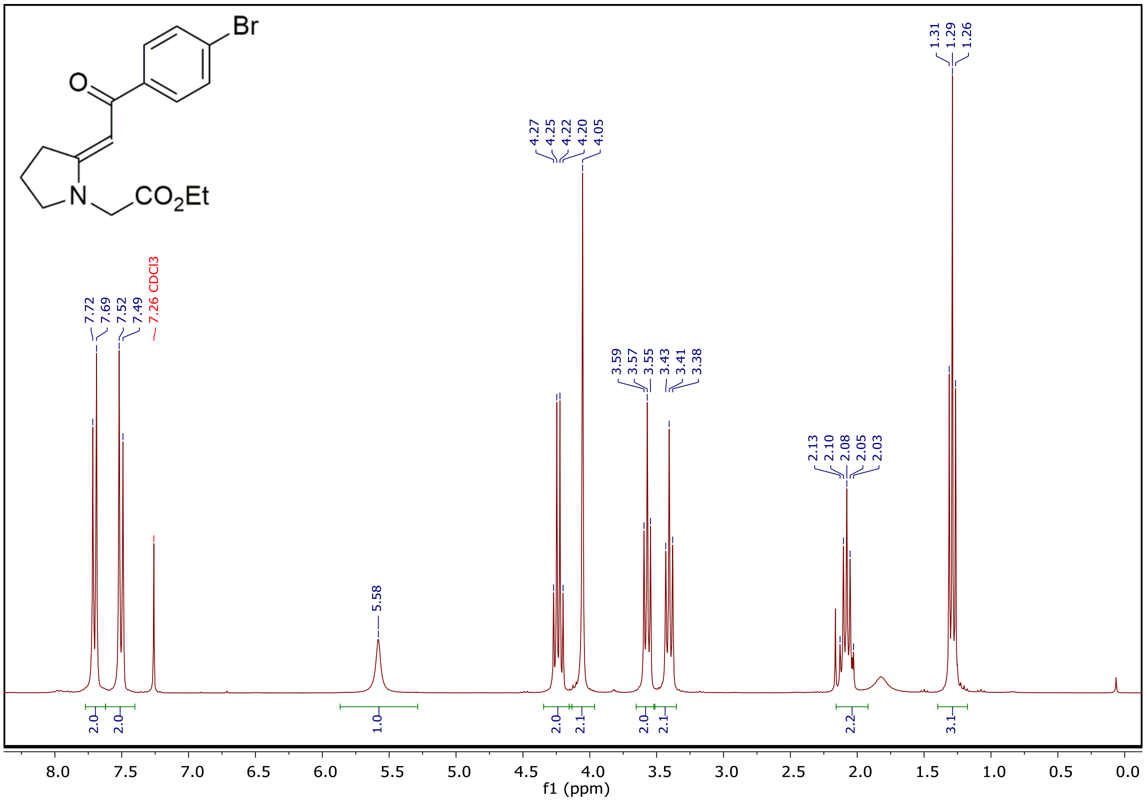

^{13}C NMR spectrum of (*E*)-ethyl 2-{2-[2-(4-bromophenyl)-2-oxoethylidene]pyrrolidin-1-yl}acetate (15e) (75 MHz, $CDCl_3$)

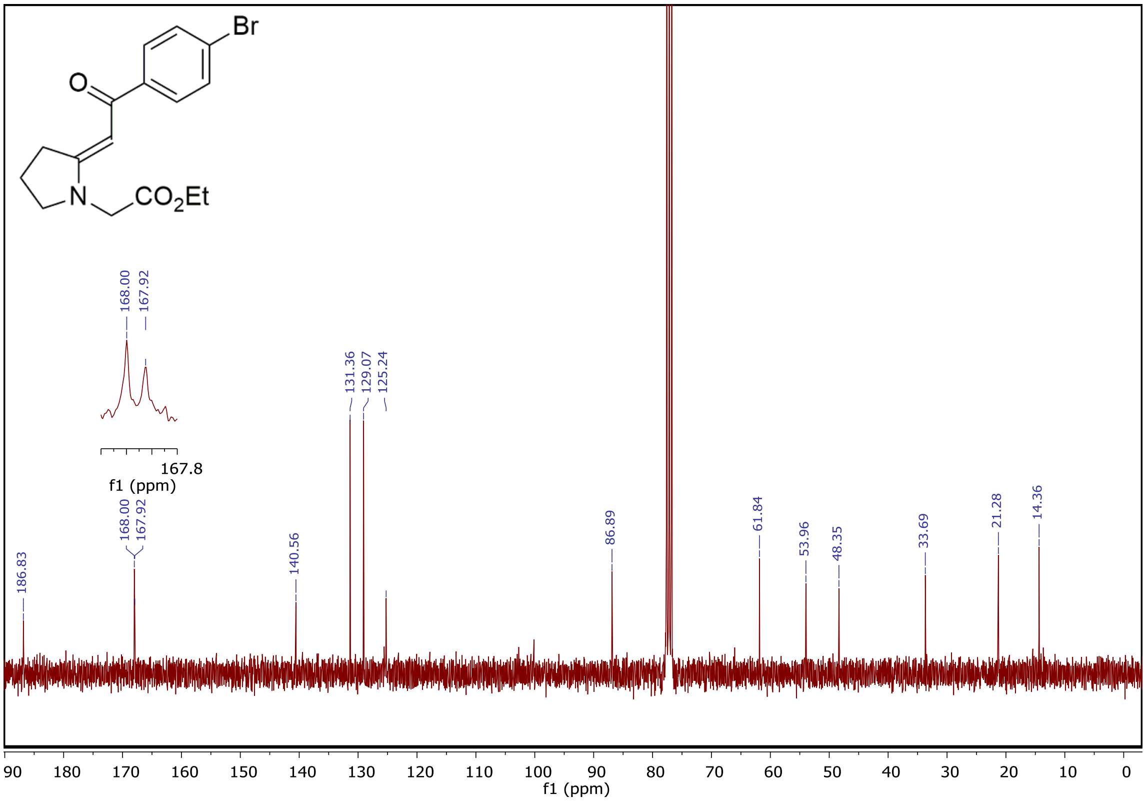

^{1}H NMR spectrum of (*E*)-ethyl 2-{2-[2-(4-methyphenyl)-2-oxoethylidene]pyrrolidin-1-yl}acetate (15f) (500 MHz, $CDCl_3$)

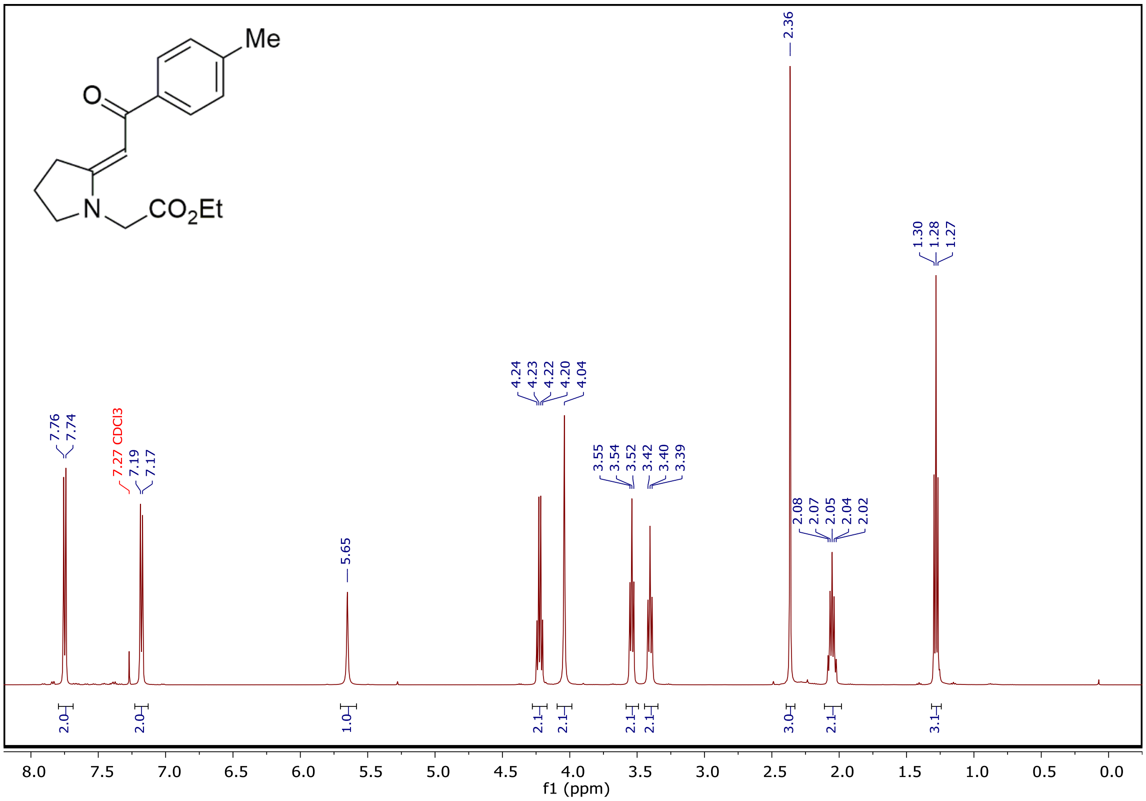

^{13}C NMR spectrum of (*E*)-ethyl 2-{2-[2-(4-methyphenyl)-2-oxoethylidene]pyrrolidin-1-yl}acetate (15f) (75 MHz, $CDCl_3$)

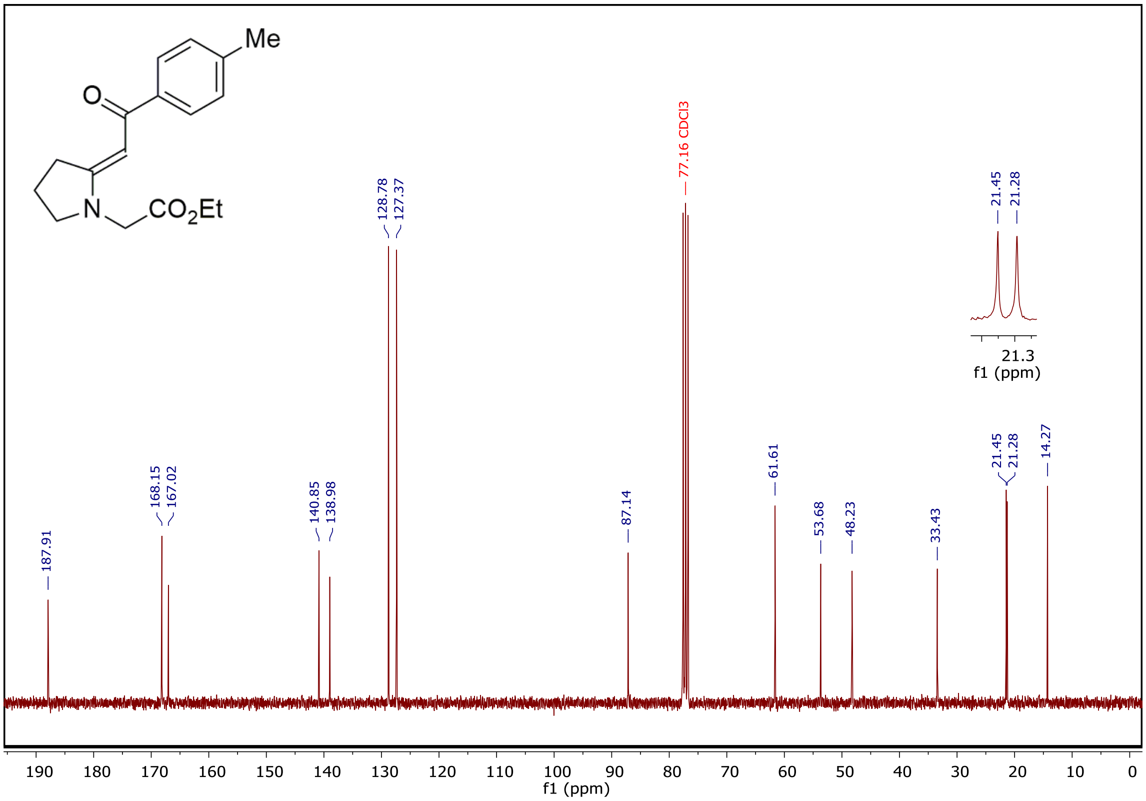

^{1}H NMR spectrum of (*E*)-ethyl 2-{2-[2-(4-methoxyphenyl)-2-oxoethylidene]pyrrolidin-1-yl}acetate (15g) (500 MHz, $CDCl_3$)

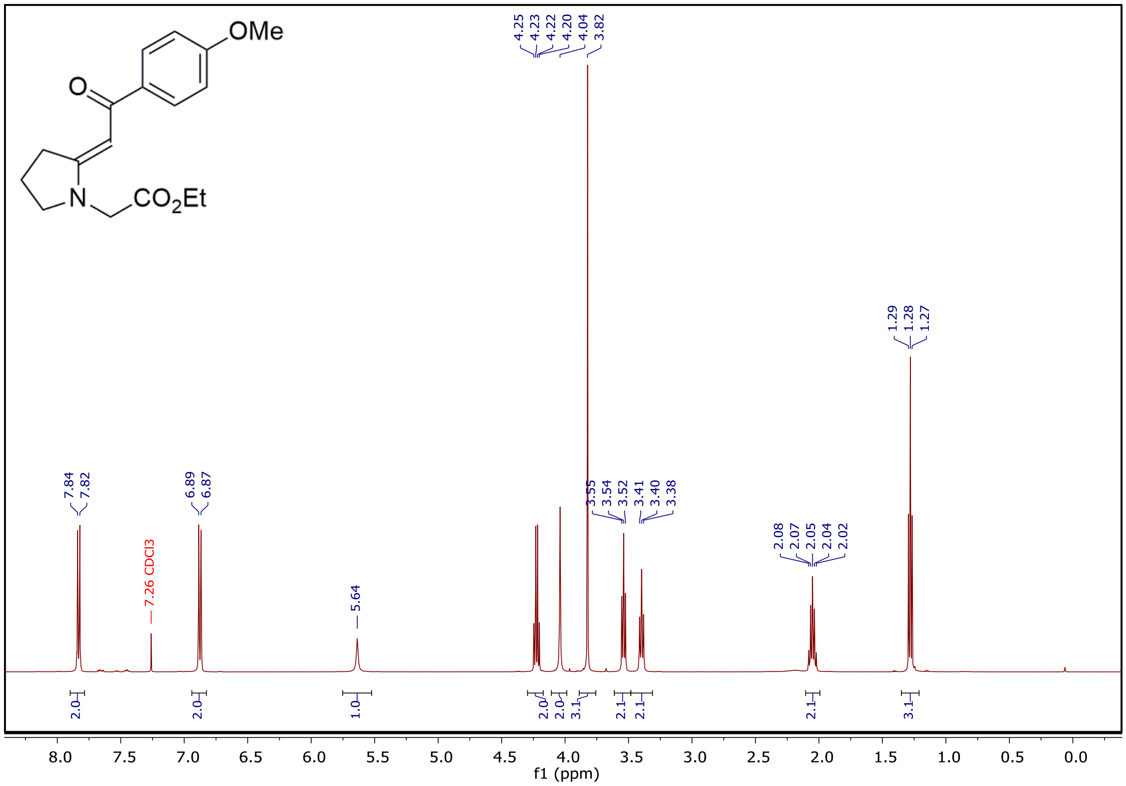

^{13}C NMR spectrum of (*E*)-ethyl 2-{2-[2-(4-methoxyphenyl)-2-oxoethylidene]pyrrolidin-1-yl}acetate (15g) (126 MHz, $CDCl_3$)

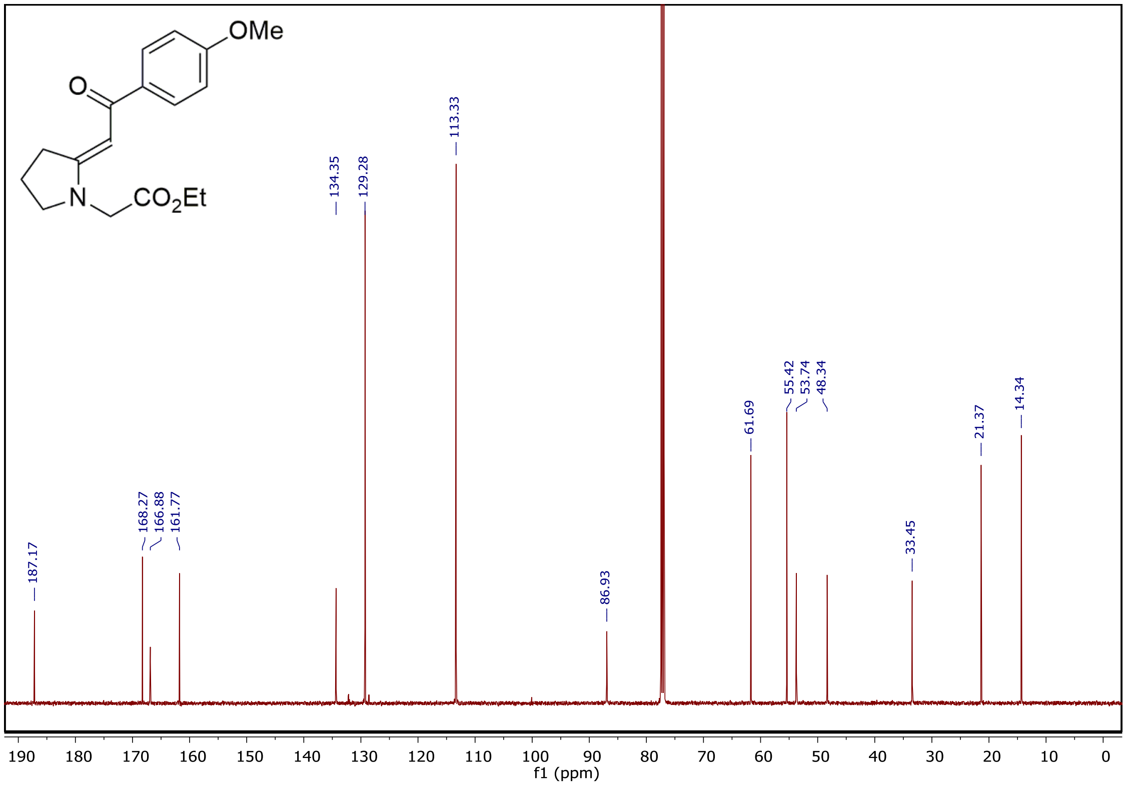

^{1}H NMR spectrum of (*E*)-ethyl 2-{2-[2-(3-nitrophenyl)-2-oxoethylidene]pyrrolidin-1-yl}acetate (15h) (300 MHz, $CDCl_3$)

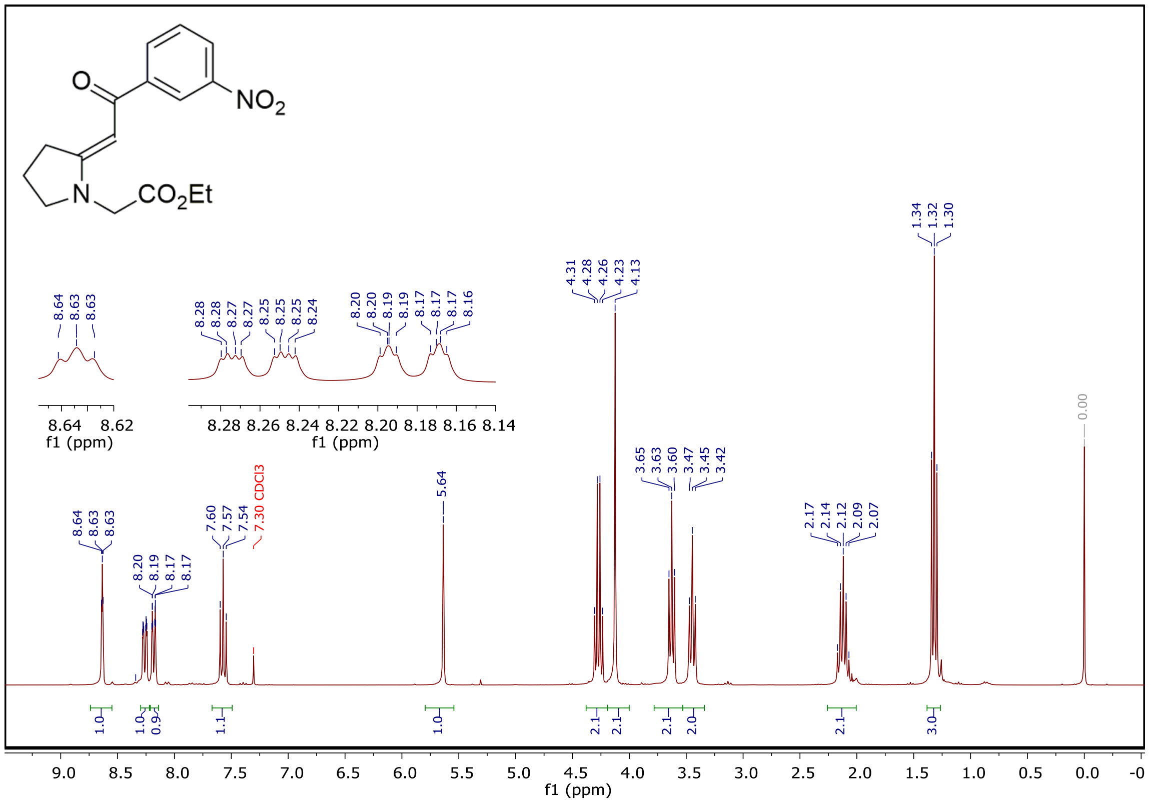

^{13}C NMR spectrum of (*E*)-ethyl 2-{2-[2-(3-nitrophenyl)-2-oxoethylidene]pyrrolidin-1-yl}acetate (15h) (75 MHz, $CDCl_3$)

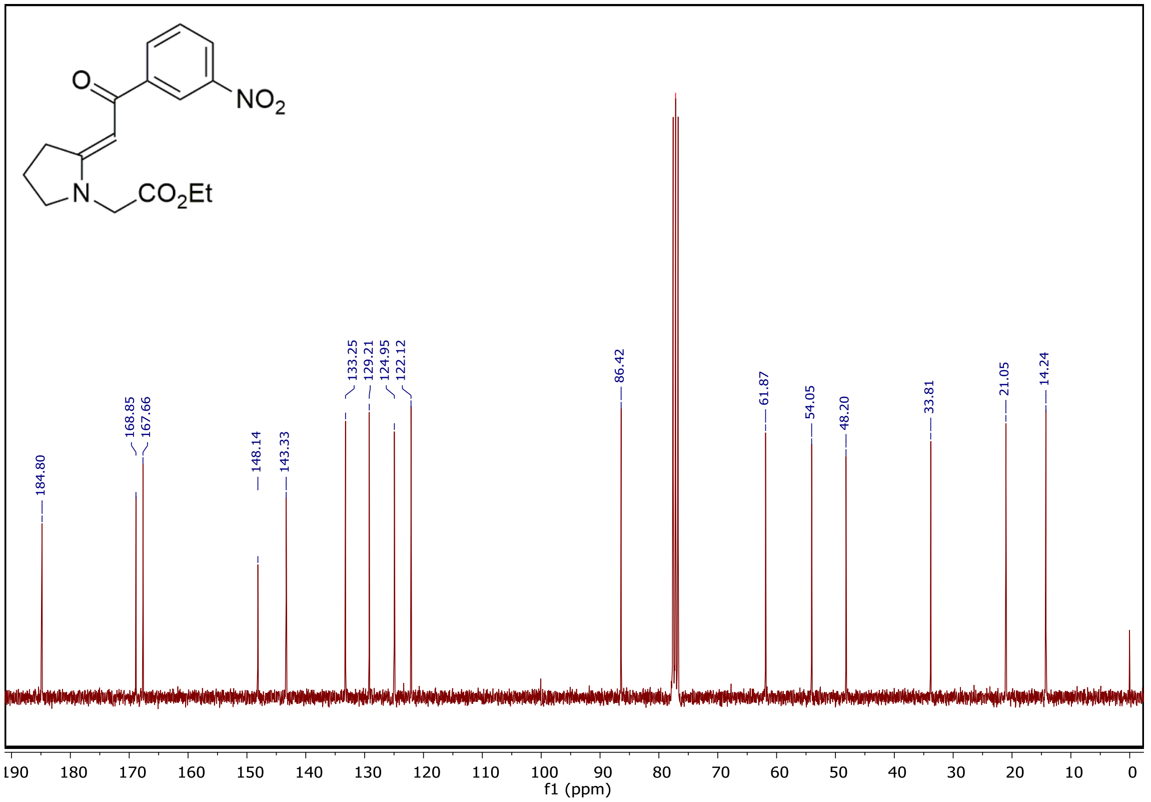

HSQC NMR spectrum of (*E*)-ethyl 2-{2-[2-(3-nitrophenyl)-2-oxoethylidene]pyrrolidin-1-yl}acetate (15h) ($CDCl_3$)

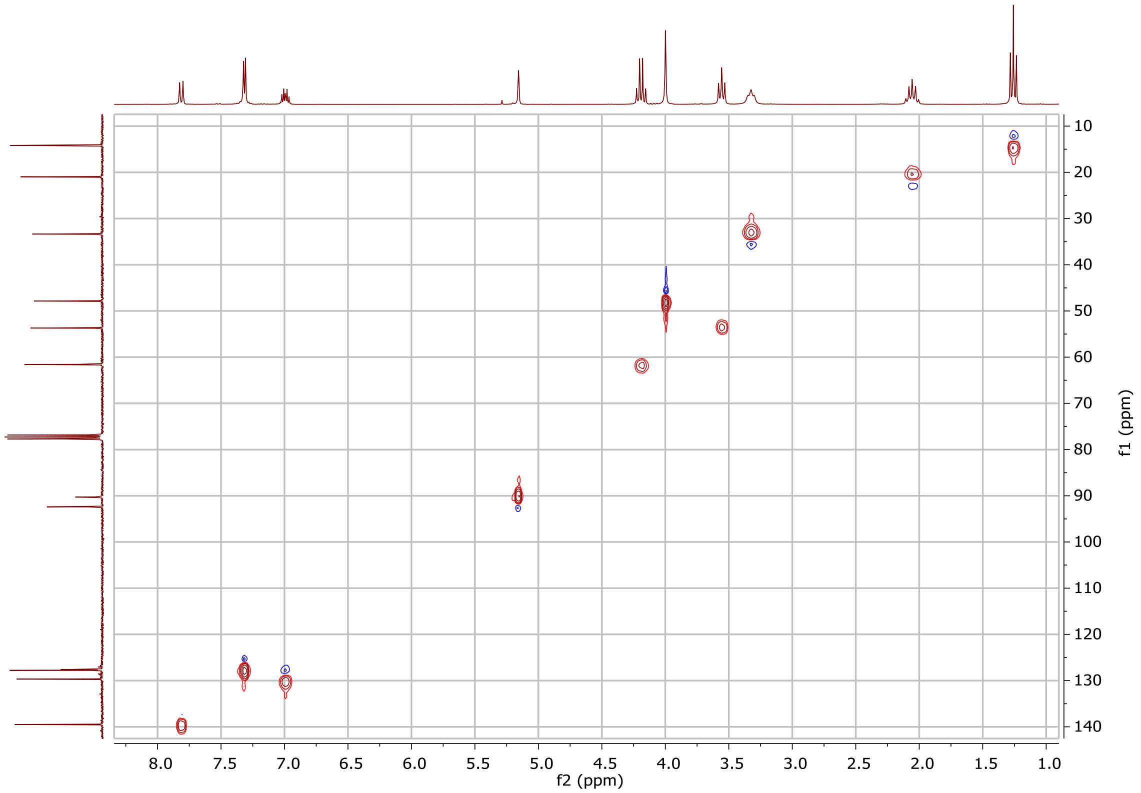

^{1}H NMR spectrum of (*E*)-ethyl 2-{2-[2-(3-methoxyphenyl)-2-oxoethylidene]pyrrolidin-1-yl}acetate (15i)* (300 MHz, $CDCl_3$)

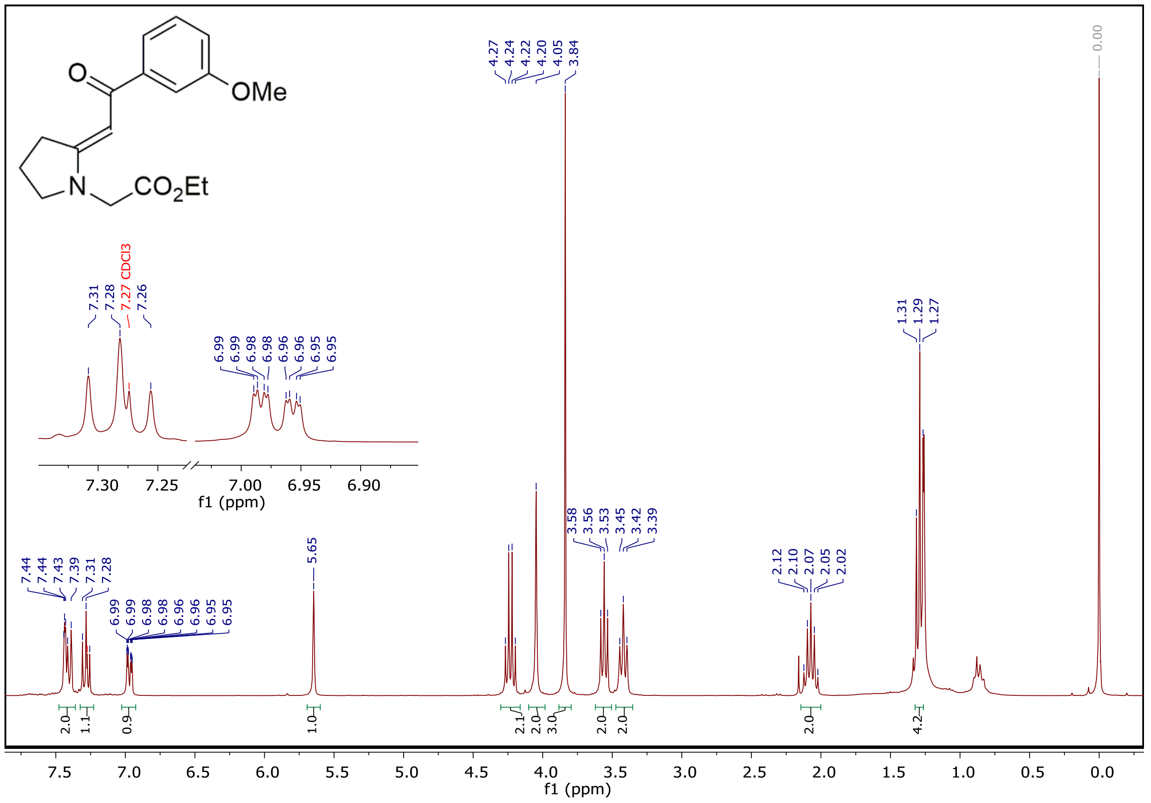

*Unpurified intermediate

^{1}H NMR spectrum of (*E*)-ethyl 2-{2-[2-(3,4-dimethoxyphenyl)-2-oxoethylidene]pyrrolidin-1-yl}acetate (15j)* (300 MHz, CDCl$_3$)

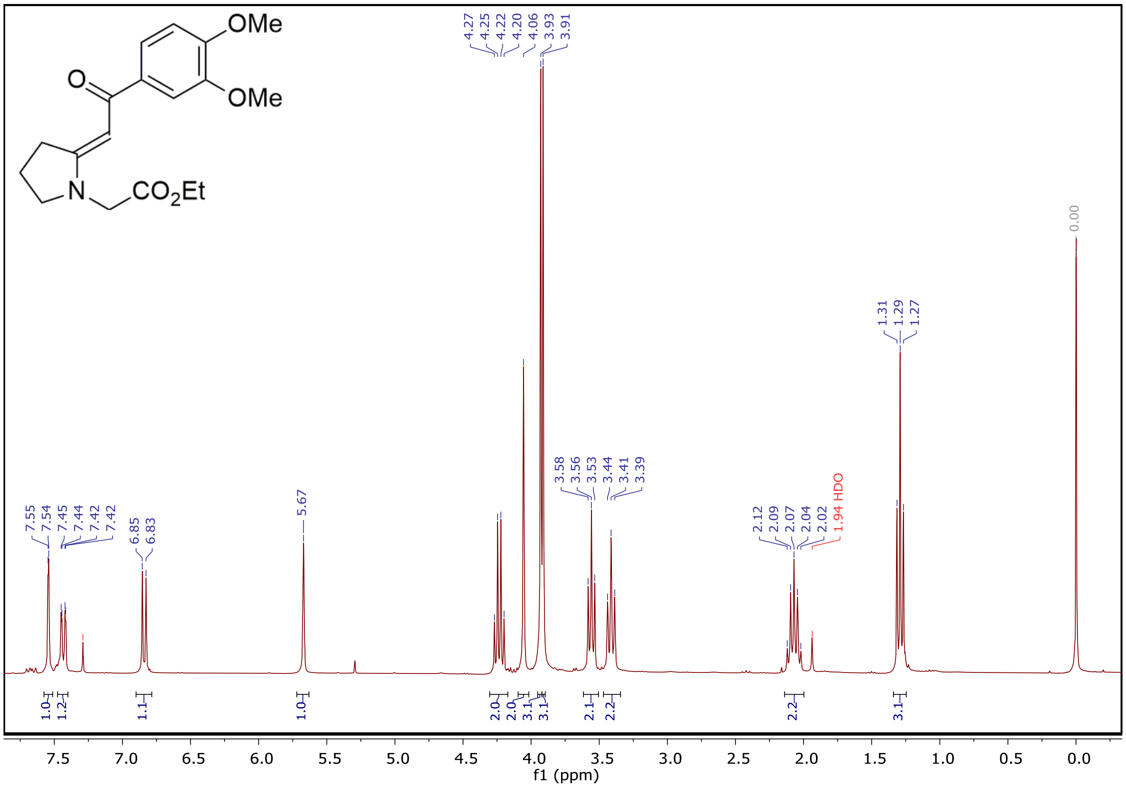

*Unpurified intermediate with P-containing by-products

^{13}C NMR spectrum of (*E*)-ethyl 2-{2-[2-(3,4-dimethoxyphenyl)-2-oxoethylidene]pyrrolidin-1-yl}acetate (15j)* (75 MHz, CDCl$_3$)

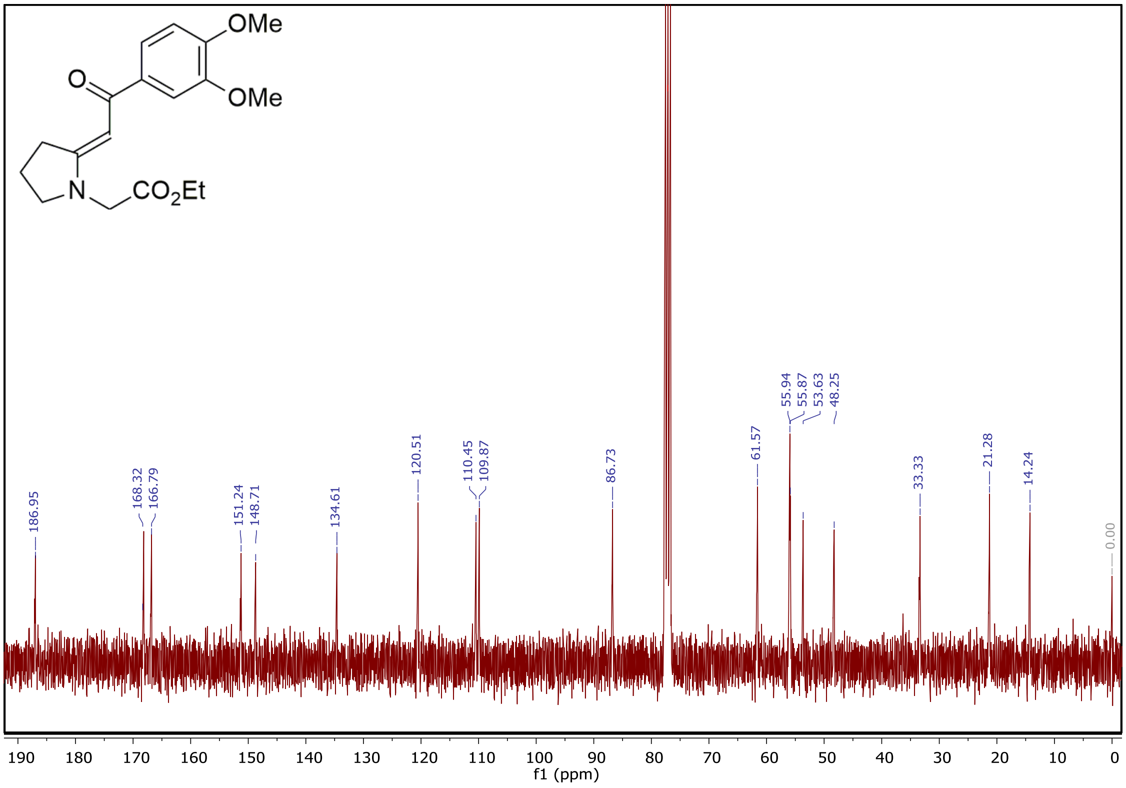

*Unpurified intermediate with P-containing by-products

^{1}H NMR spectrum of (*E*)-ethyl 2-{2-[2-(2-nitrophenyl)-2-oxoethylidene]pyrrolidin-1-yl}acetate (15k) (300 MHz, $CDCl_3$)

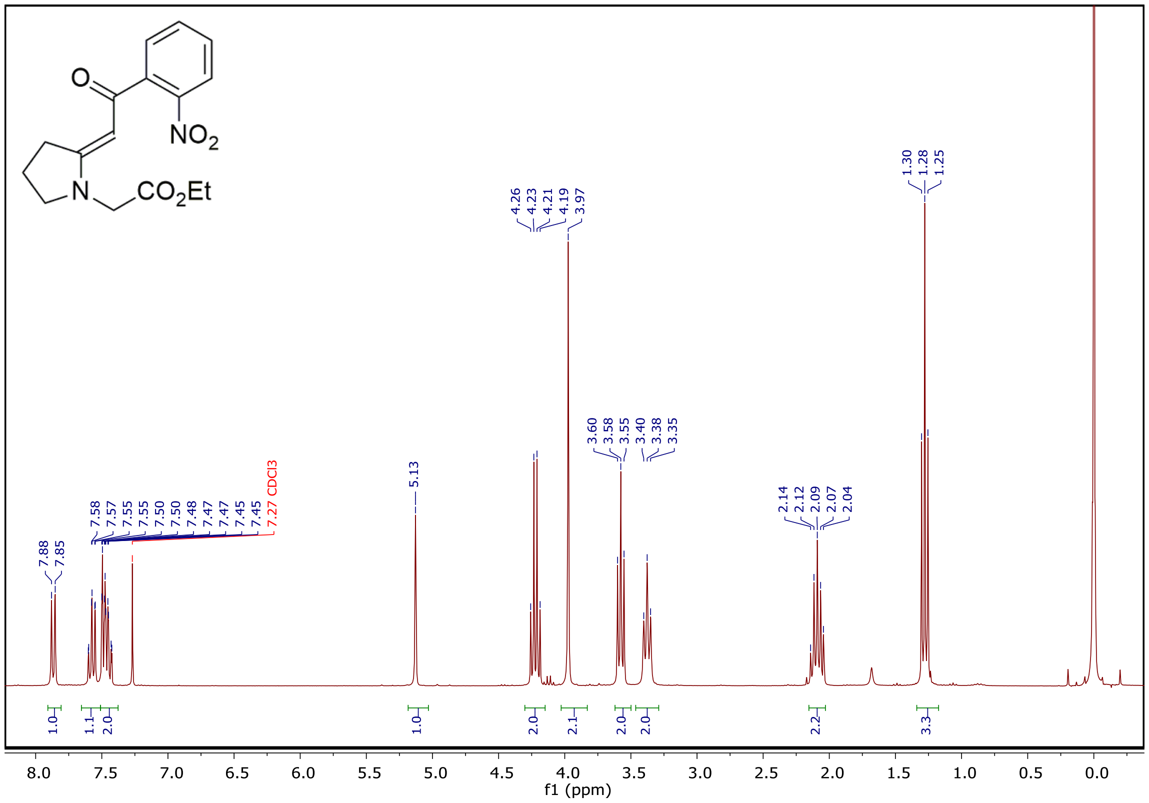

^{13}C NMR spectrum of (*E*)-ethyl 2-{2-[2-(2-nitrophenyl)-2-oxoethylidene]pyrrolidin-1-yl}acetate (15k) (75 MHz, $CDCl_3$)

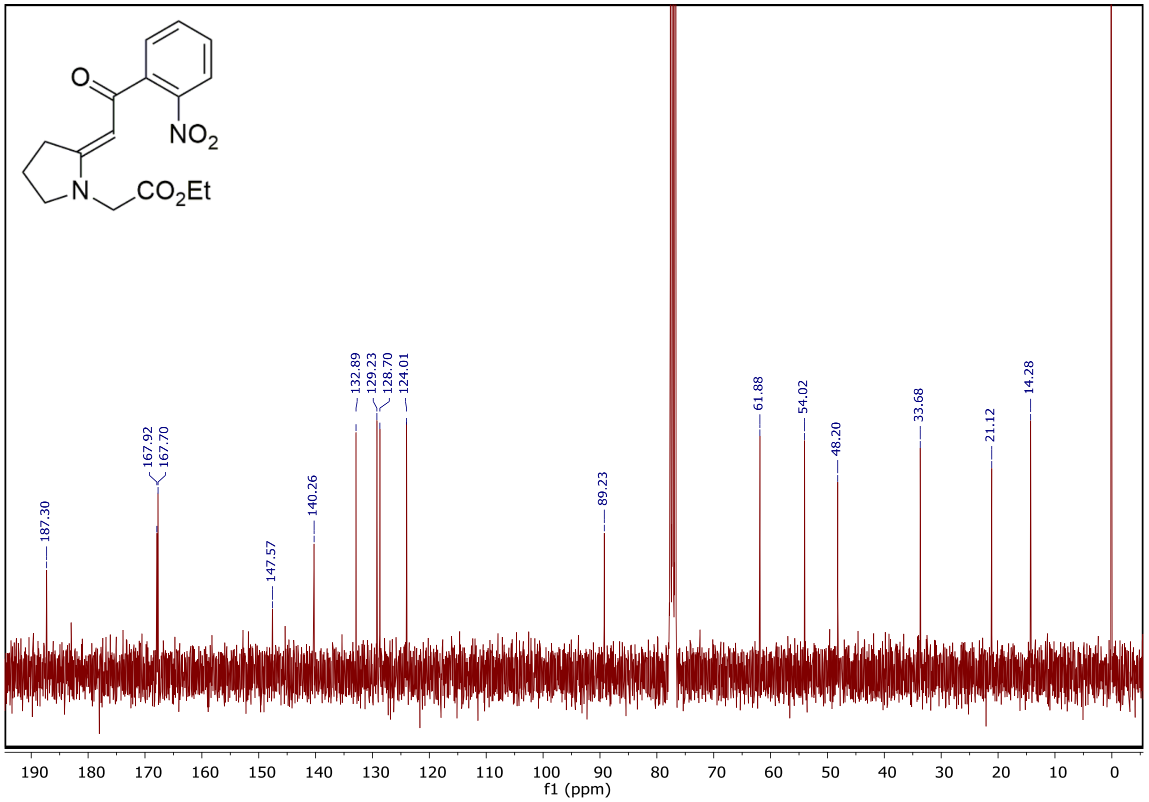

^{1}H NMR spectrum of (*E*)-ethyl 2-{2-[2-(2-iodophenyl)-2-oxoethylidene]pyrrolidin-1-yl}acetate (15l) (300 MHz, $CDCl_3$)

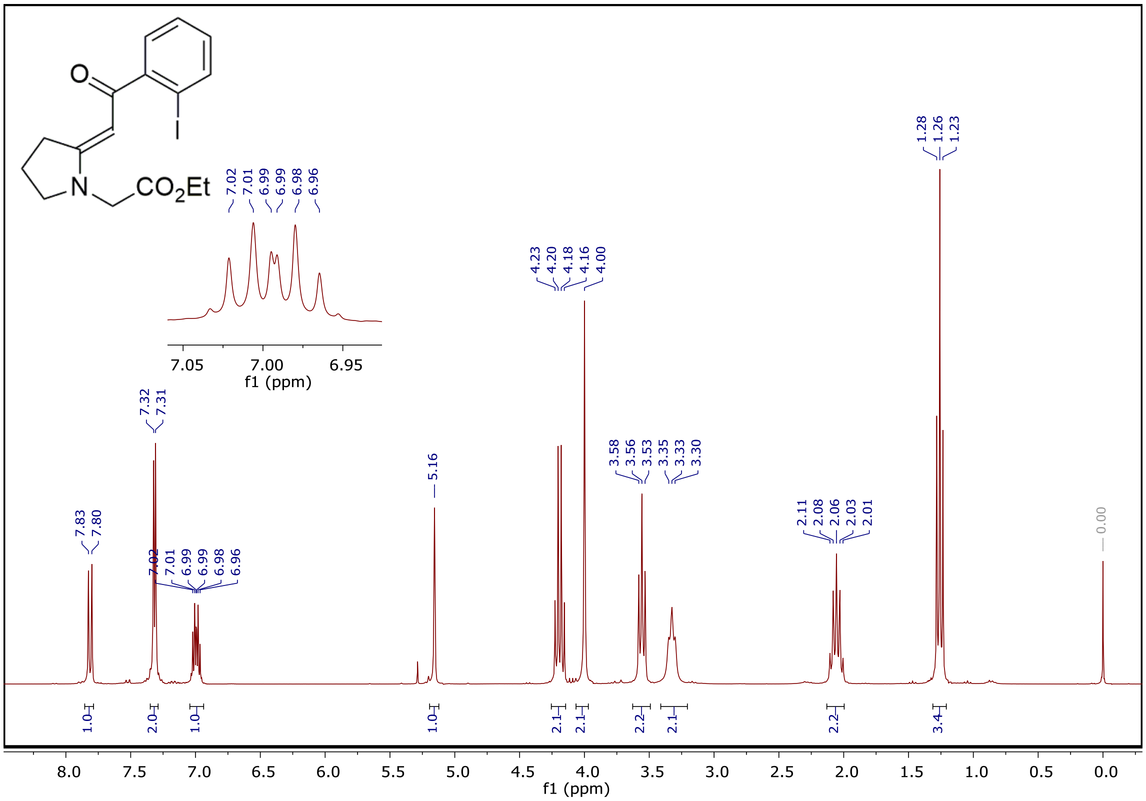

^{13}C NMR spectrum of (*E*)-ethyl 2-{2-[2-(2-iodophenyl)-2-oxoethylidene]pyrrolidin-1-yl}acetate (15l) (75 MHz, $CDCl_3$)

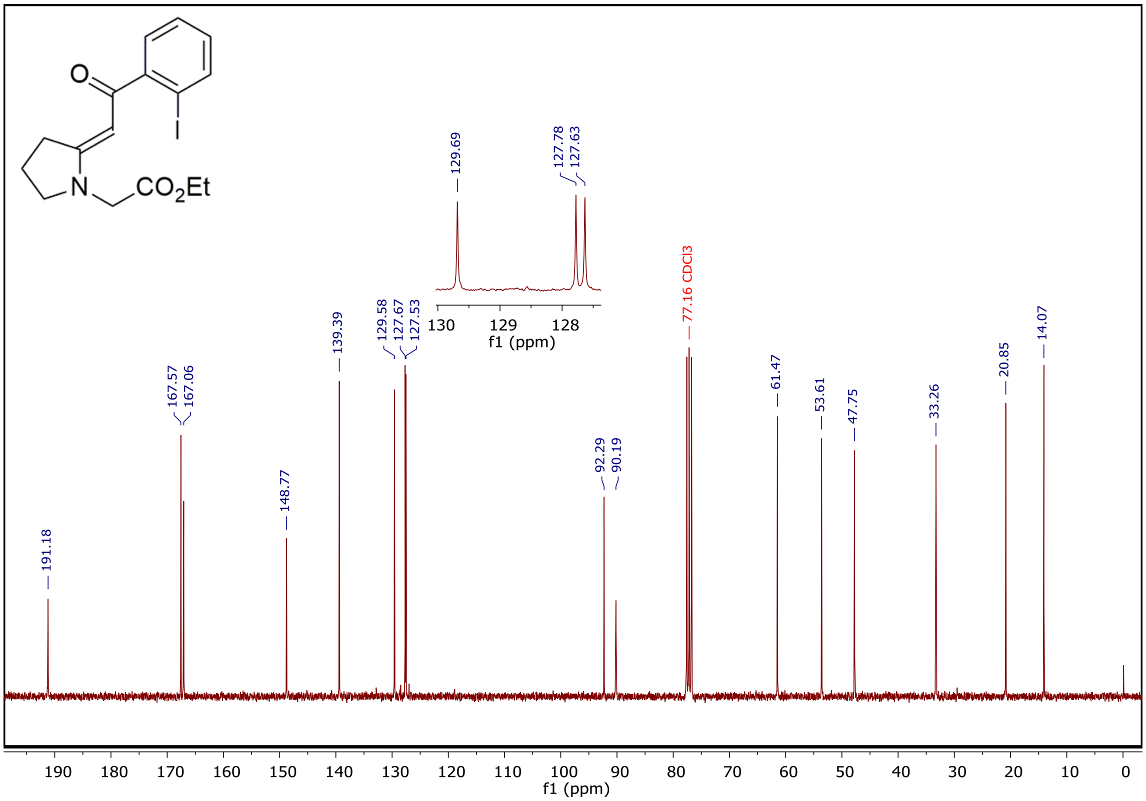

HSQC NMR spectrum of (*E*)-ethyl 2-{2-[2-(2-iodophenyl)-2-oxoethylidene]pyrrolidin-1-yl}acetate (15l) ($CDCl_3$)

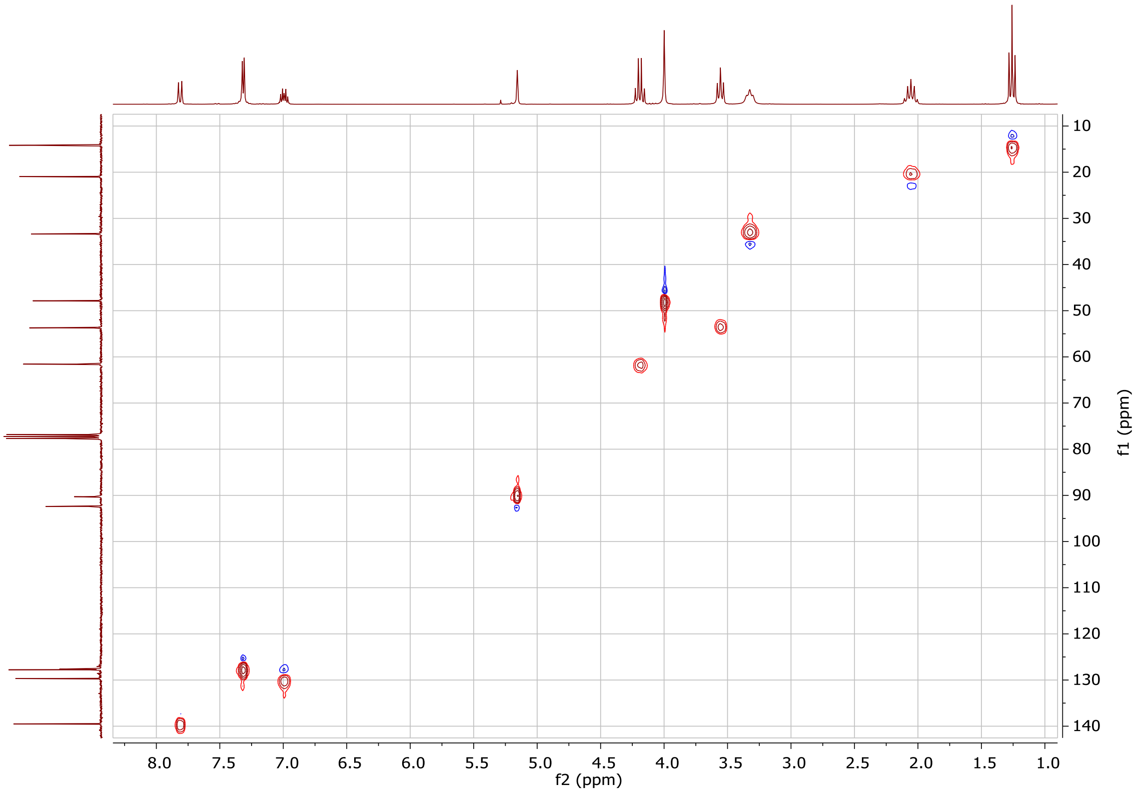

^{1}H NMR spectrum of (*E*)-ethyl 2-{2-[2-(2-bromophenyl)-2-oxoethylidene]pyrrolidin-1-yl}acetate (15m)* (300 MHz, $CDCl_3$)

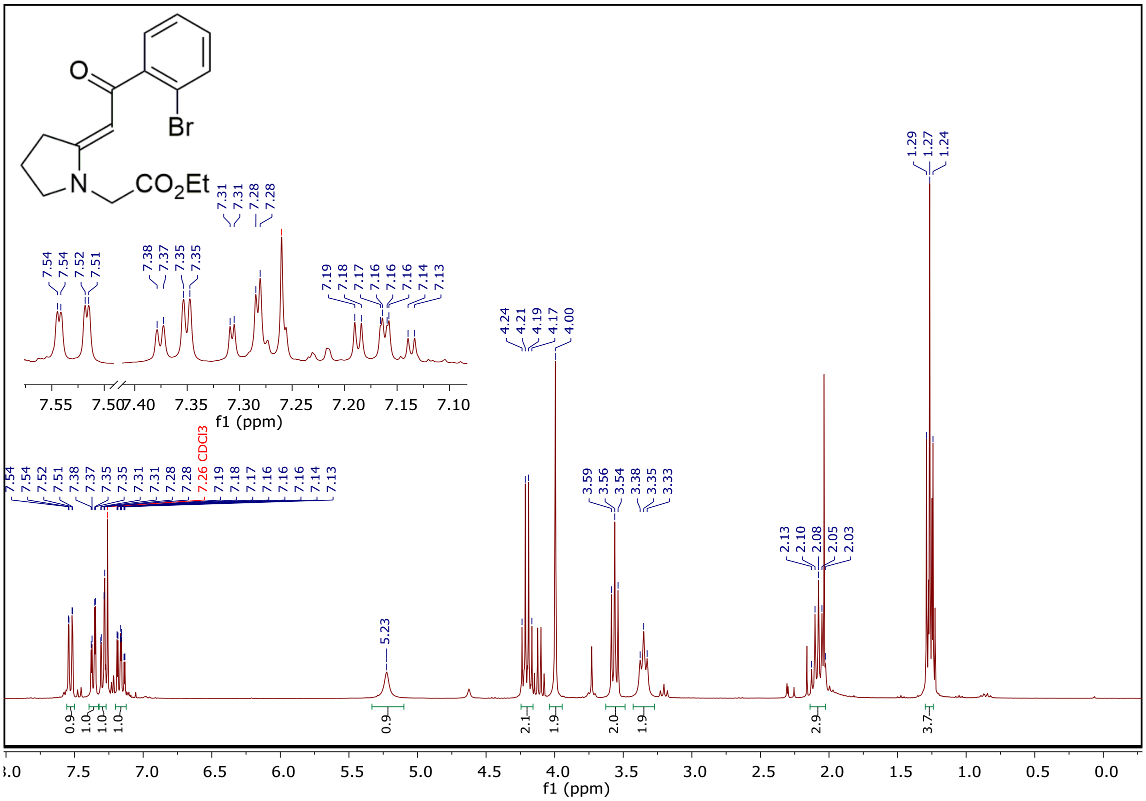

*Unpurified intermediate with P-containing by-products

^{1}H NMR spectrum of (*E*)-ethyl 2-{2-[2-(2-bromo-4,5-dimethoxyphenyl)-2-oxoethylidene]pyrrolidin-1-yl}acetate (15n)*
(300 MHz, $CDCl_3$)

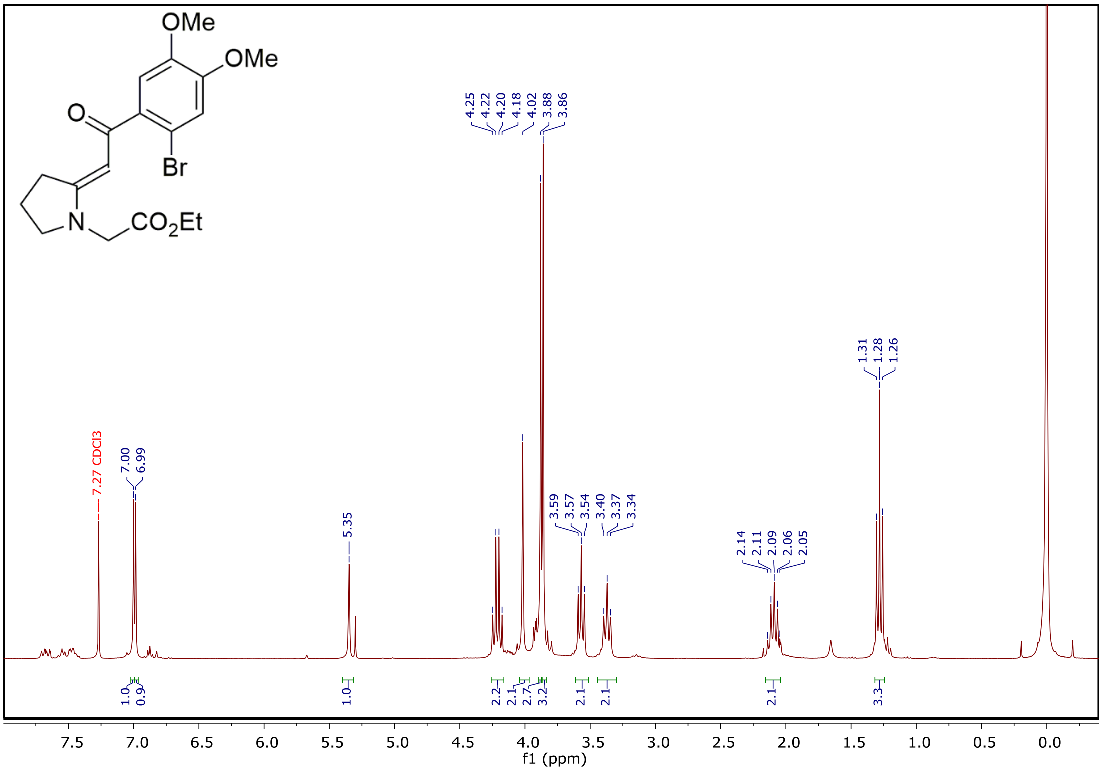

*Unpurified intermediate with P-containing by-products

^{13}C NMR spectrum of (*E*)-ethyl 2-{2-[2-(2-bromo-4,5-dimethoxyphenyl)-2-oxoethylidene]pyrrolidin-1-yl}acetate (15n; crude) (75 MHz, $CDCl_3$)

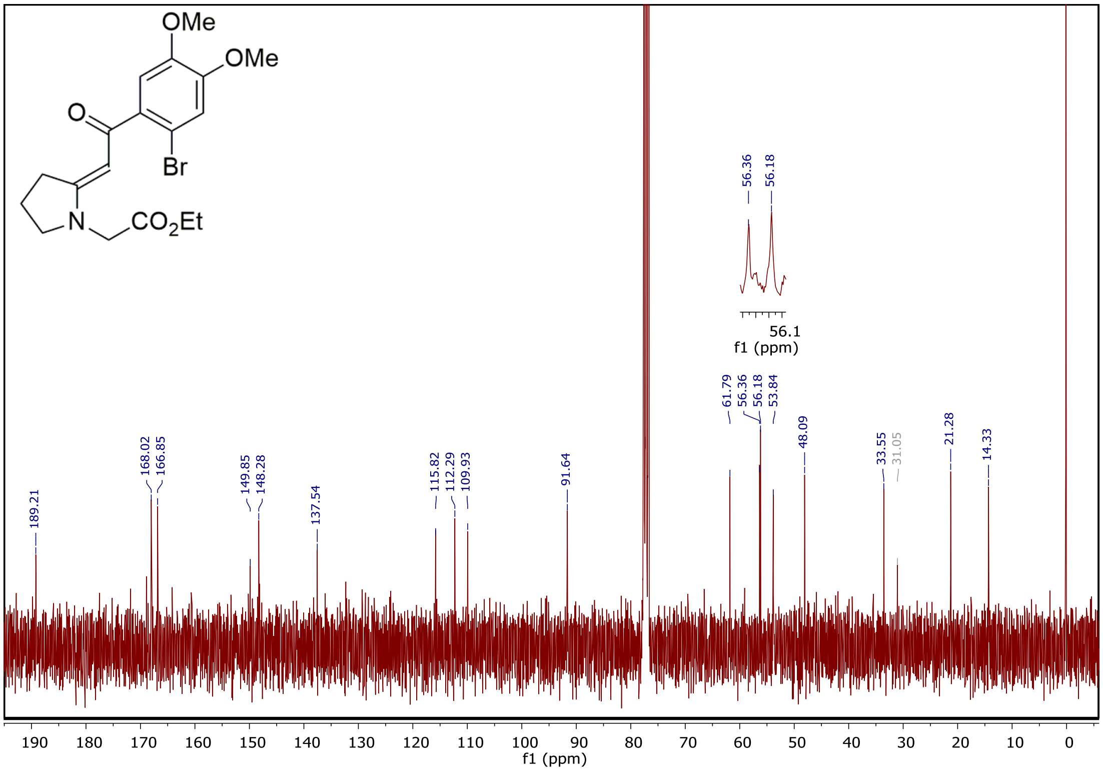

*Unpurified intermediate with P-containing by-products

^{1}H NMR spectrum of (*E*)-ethyl 2-{2-[2-(2-chlorophenyl)-2-oxoethylidene]pyrrolidin-1-yl}acetate (15o) (300 MHz, $CDCl_3$)

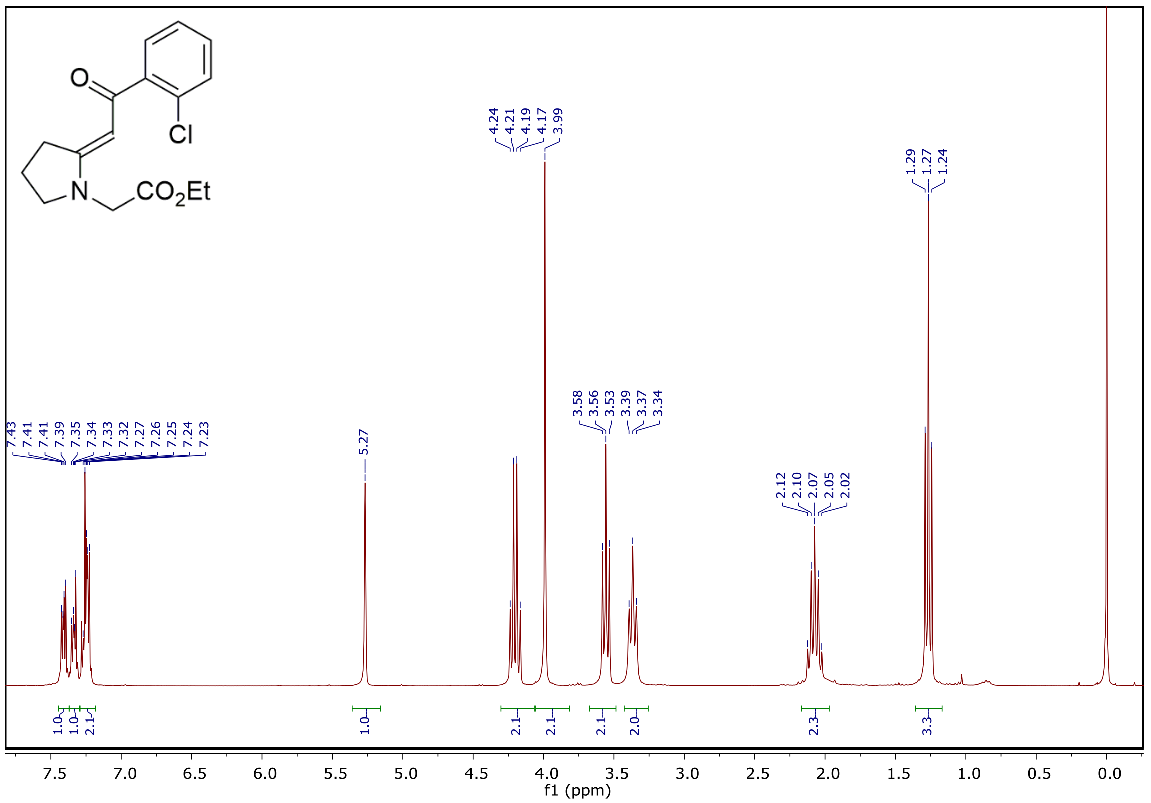

^{13}C NMR spectrum of (*E*)-ethyl 2-{2-[2-(2-chlorophenyl)-2-oxoethylidene]pyrrolidin-1-yl}acetate (15o) (75 MHz, $CDCl_3$)

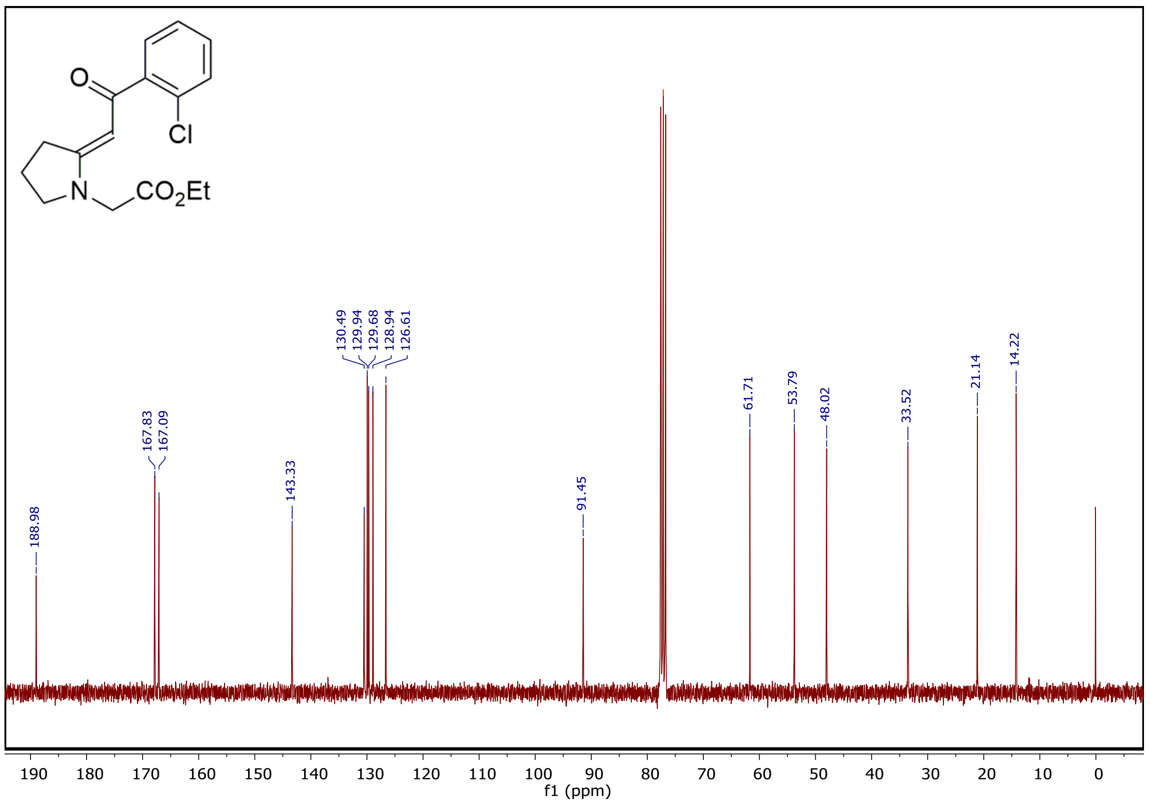

^{1}H NMR spectrum of (*E*)-ethyl 2-{2-[2-(2-methoxyphenyl)-2-oxoethylidene]pyrrolidin-1-yl}acetate (15p)* (300 MHz, $CDCl_3$)

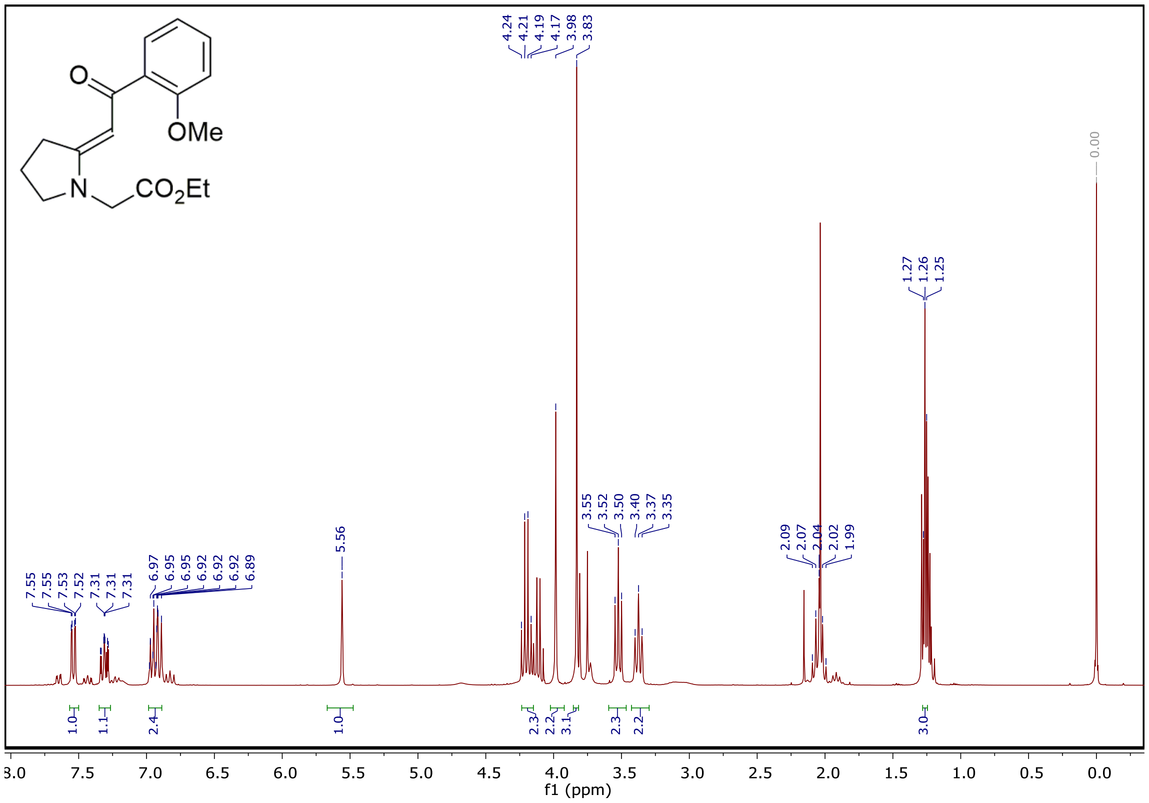

*Unpurified intermediate with P-containing by-products

^{13}C NMR spectrum of (*E*)-ethyl 2-{2-[2-(2-methoxyphenyl)-2-oxoethylidene]pyrrolidin-1-yl}acetate (15p)* (75 MHz, $CDCl_3$)

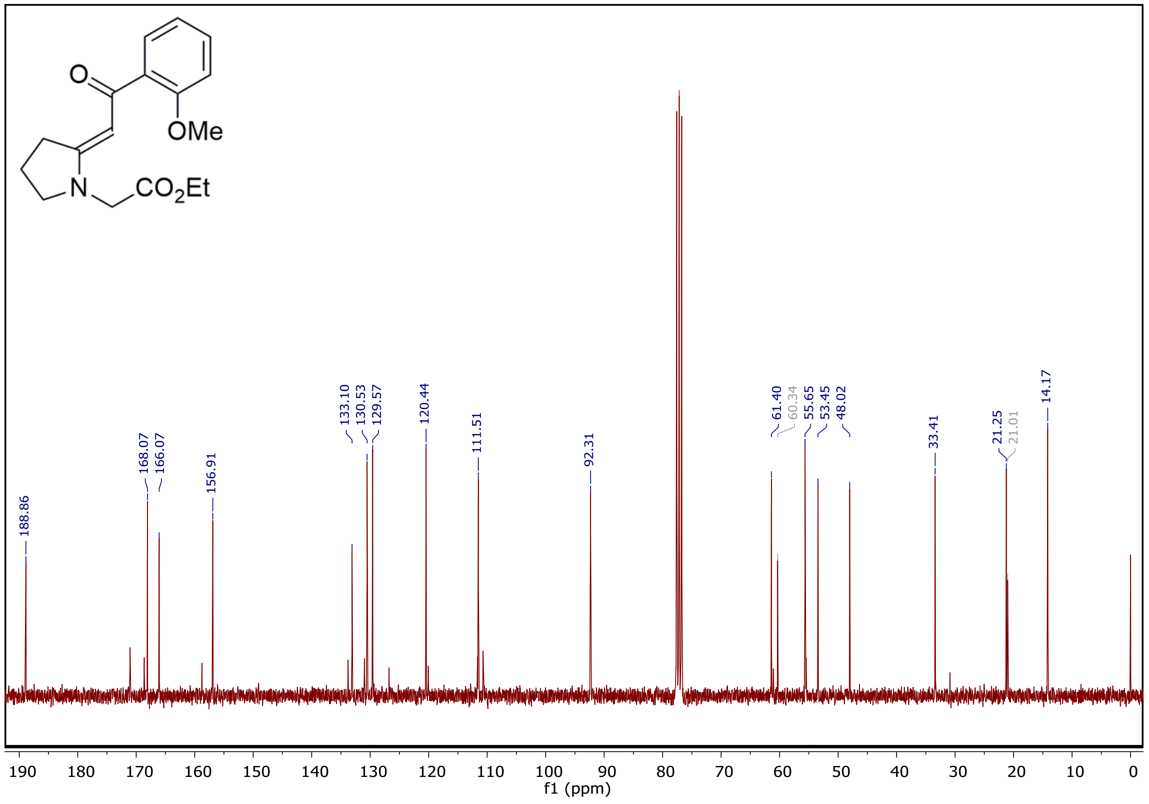

*Unpurified intermediate with P-containing by-products

^{1}H NMR spectrum of (*E*)-ethyl 2-{2-[2-(2,5-dimethoxyphenyl)-2-oxoethylidene]pyrrolidin-1-yl}acetate (15q)* (300 MHz, $CDCl_3$)

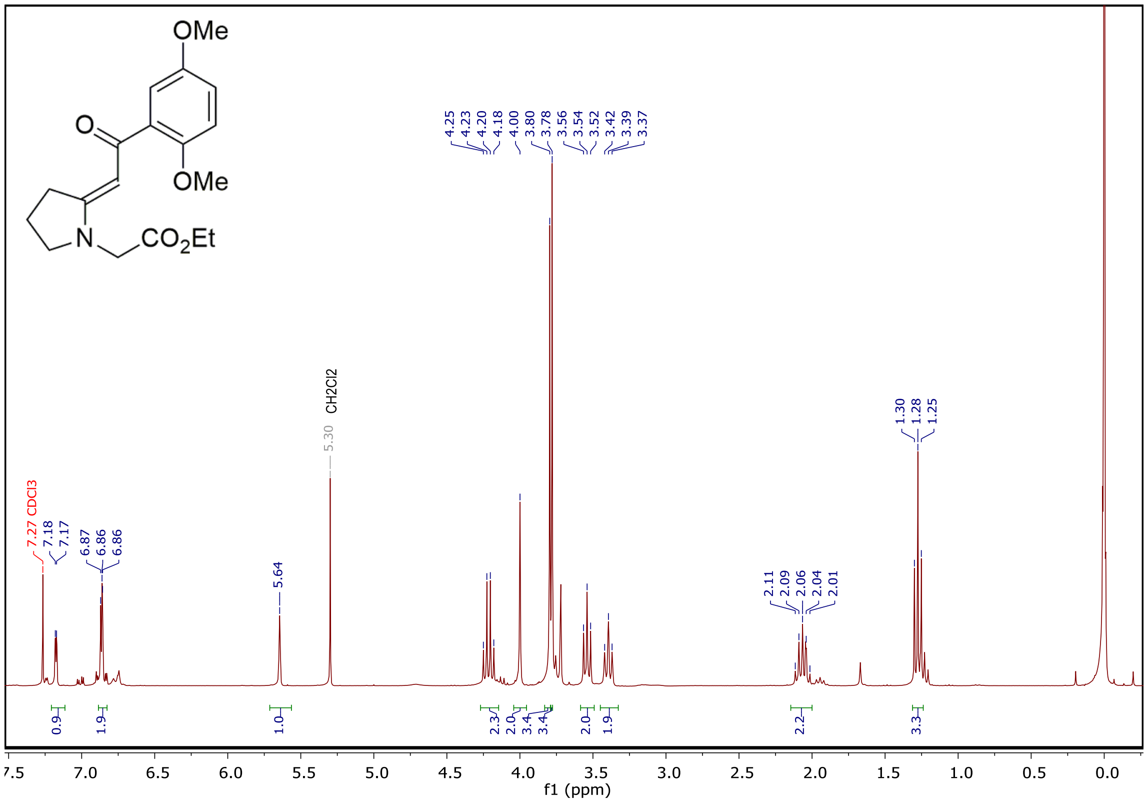

*Unpurified intermediate with P-containing by-products

^{13}C NMR spectrum of (*E*)-ethyl 2-{2-[2-(2,5-dimethoxyphenyl)-2-oxoethylidene]pyrrolidin-1-yl}acetate (15q)* (75 MHz, $CDCl_3$)

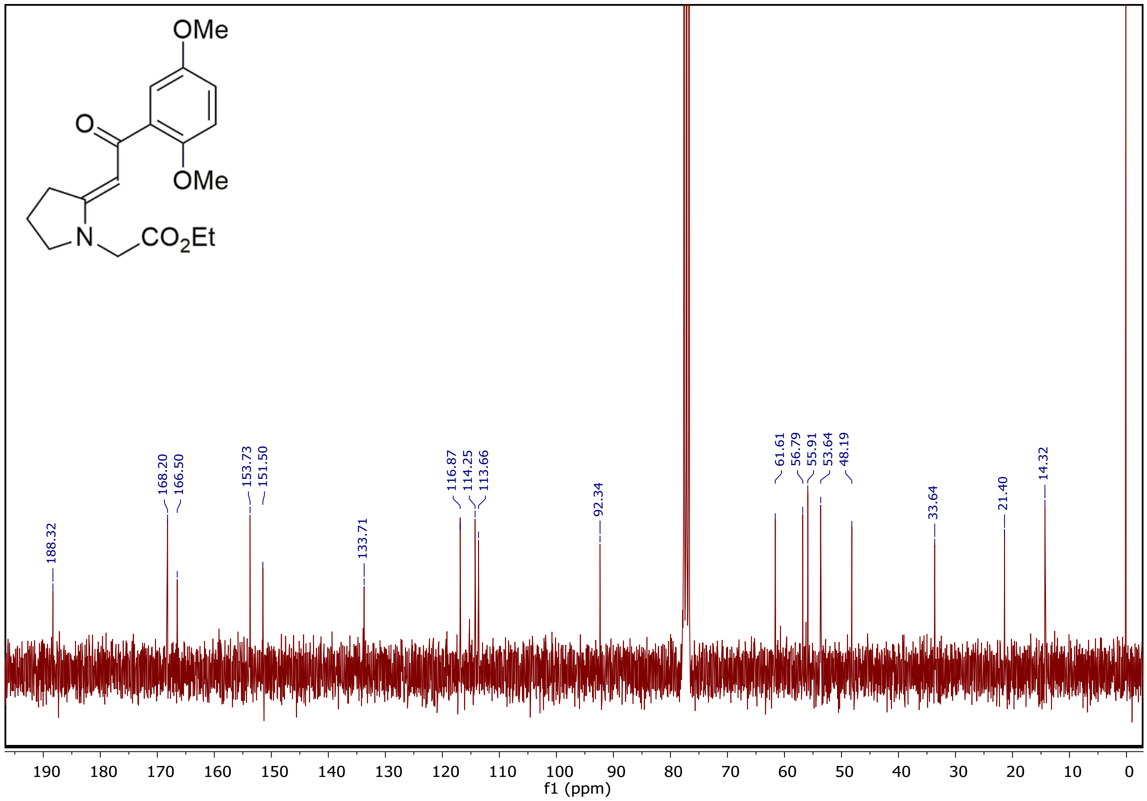

*Unpurified intermediate with P-containing by-products

^{1}H NMR spectrum of (*E*)-ethyl 2-{2-[2-(naphthalen-1-yl)-2-oxoethylidene]pyrrolidin-1-yl}acetate (15s)* (300 MHz, $CDCl_3$)

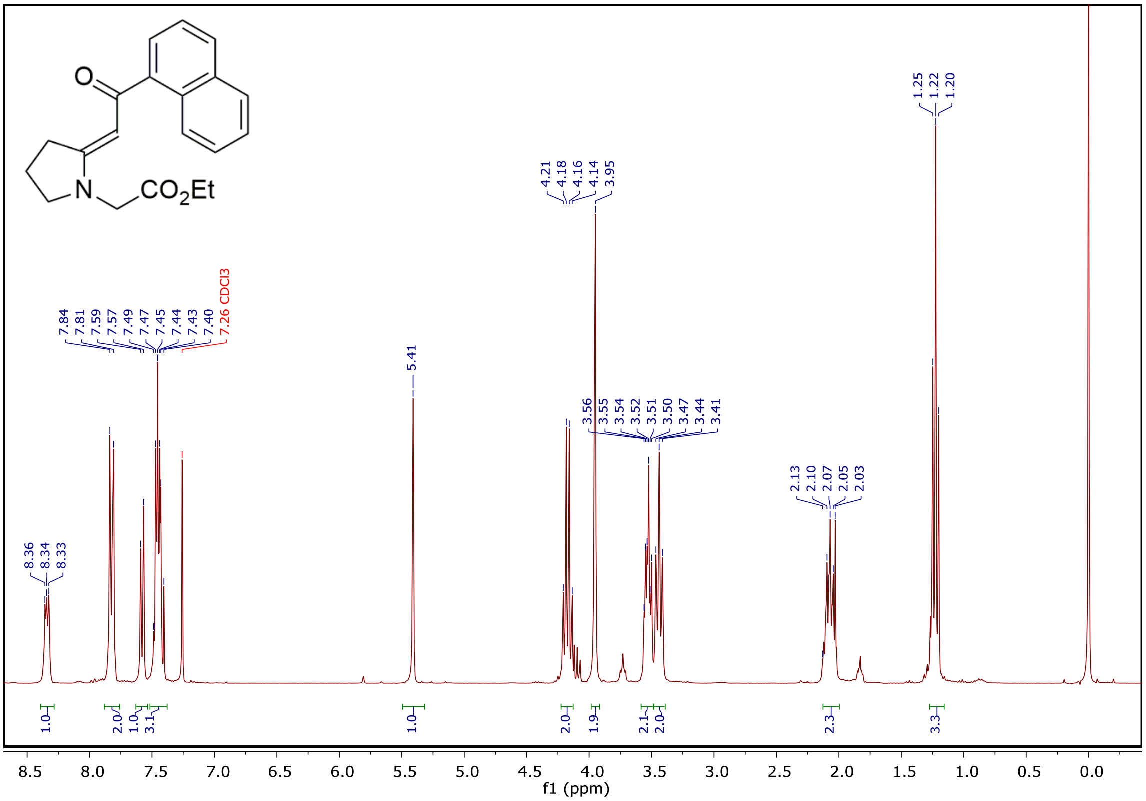

*Unpurified intermediate with P-containing by-products

^{1}H NMR spectrum of (*E*)-ethyl 2-{2-[2-oxo-2-(styryl)ethylidene]pyrrolidin-1-yl}acetate (15t) (300 MHz, $CDCl_3$)

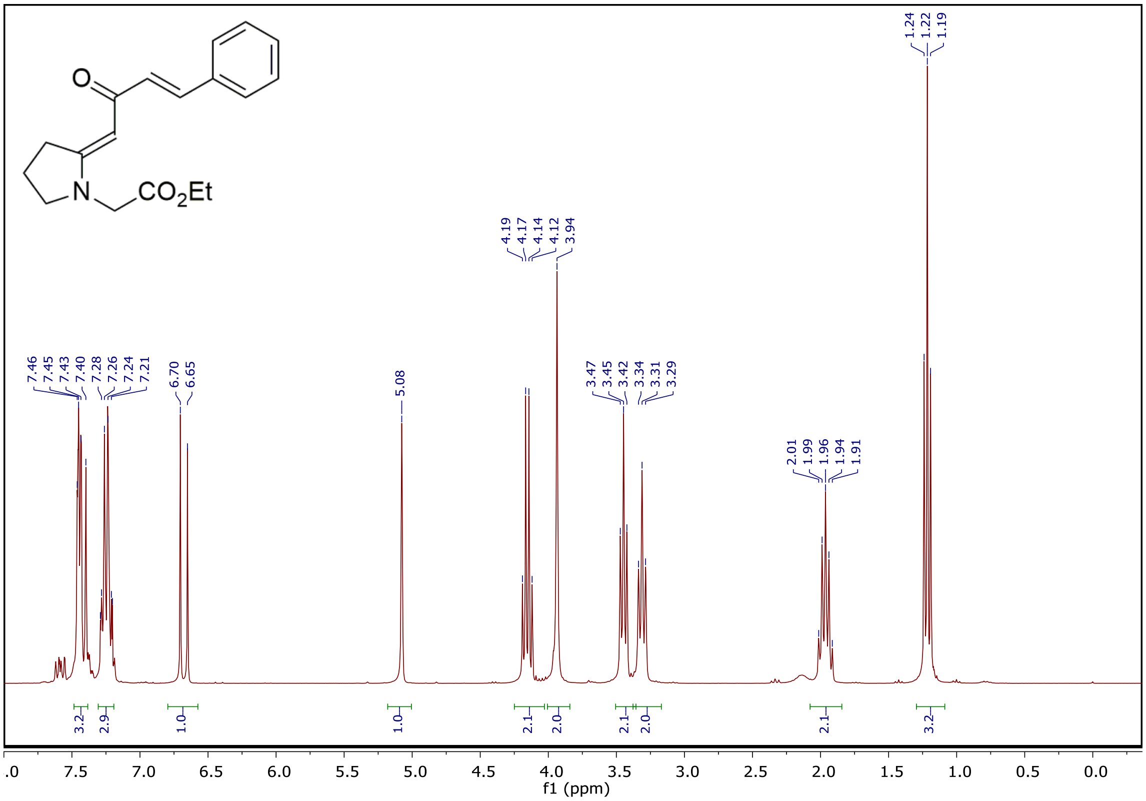

COSY NMR spectrum of (*E*)-ethyl 2-{2-[2-oxo-2-(styryl)ethylidene]pyrrolidin-1-yl}acetate (15t) (300 MHz, $CDCl_3$)

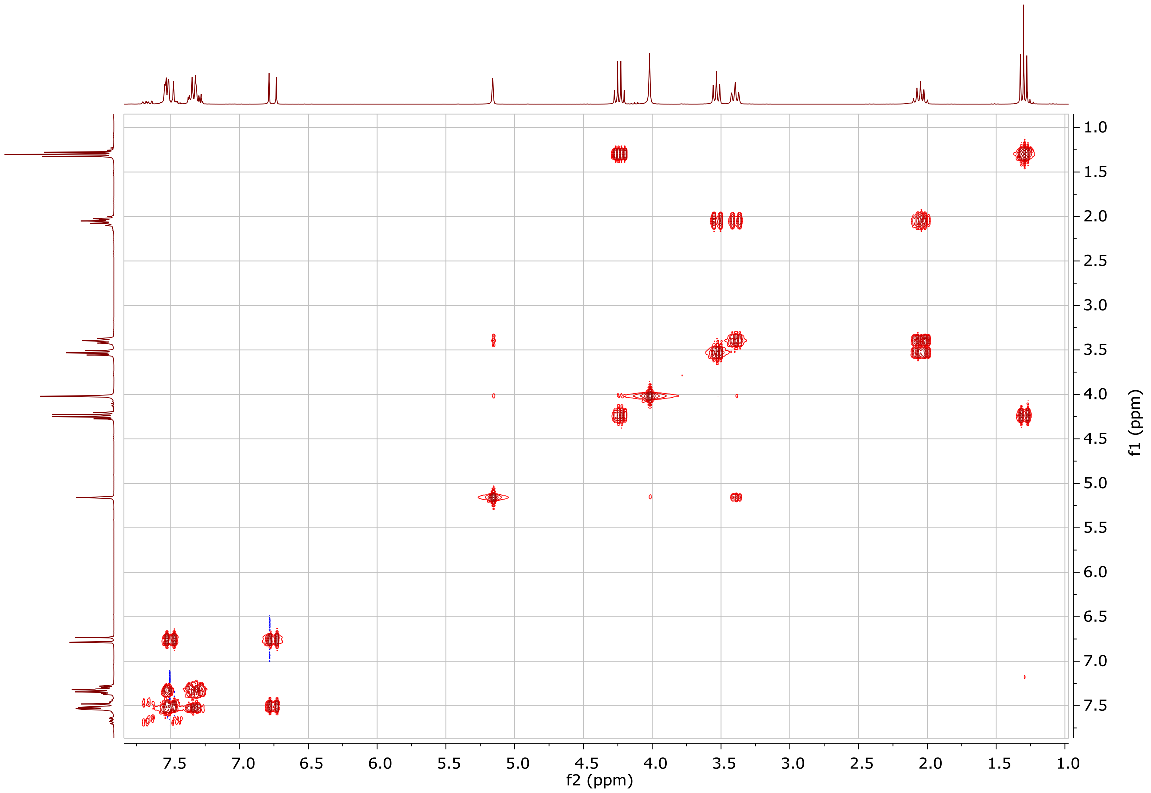

^{13}C NMR spectrum of ethyl (*E*)-ethyl 2-{2-[2-oxo-2-(styryl)ethylidene]pyrrolidin-1-yl}acetate (15t) (75 MHz, $CDCl_3$)

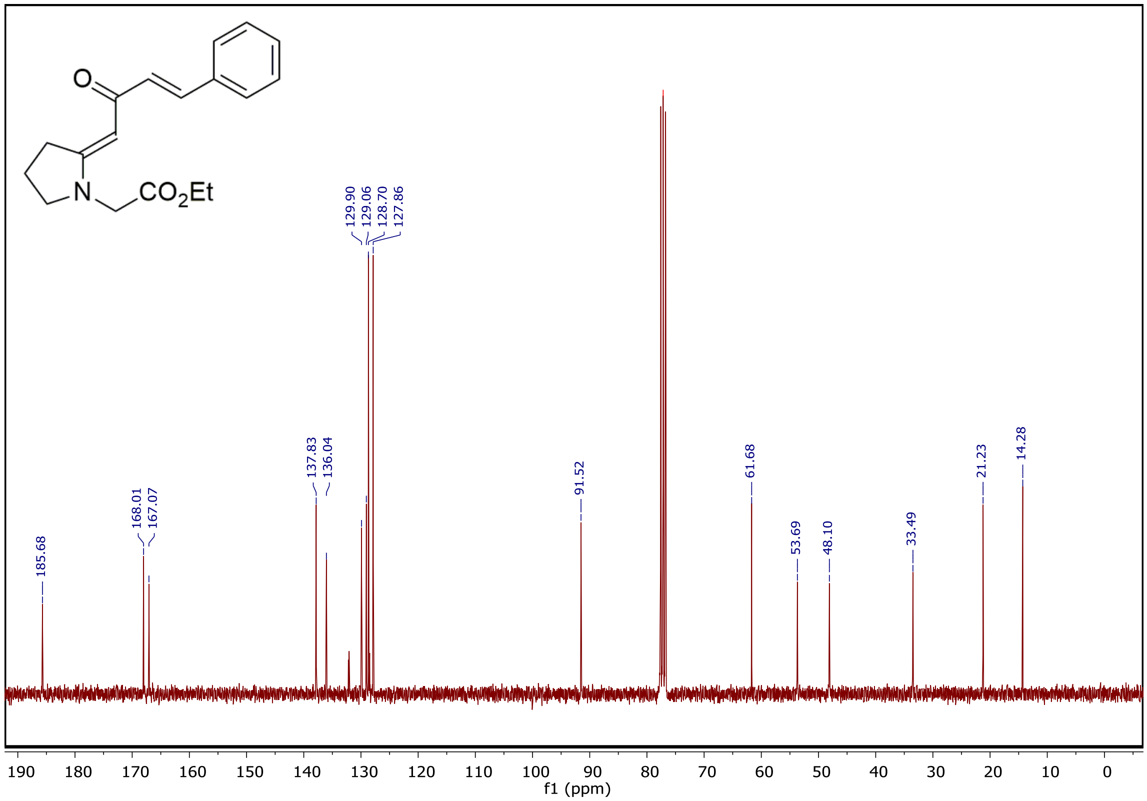

^{1}H NMR spectrum of (*E*)-ethyl 2-{2-[2-(furan-2-yl)-2-oxoethylidene]pyrrolidin-1-yl}acetate (15u) (300 MHz, $CDCl_3$)

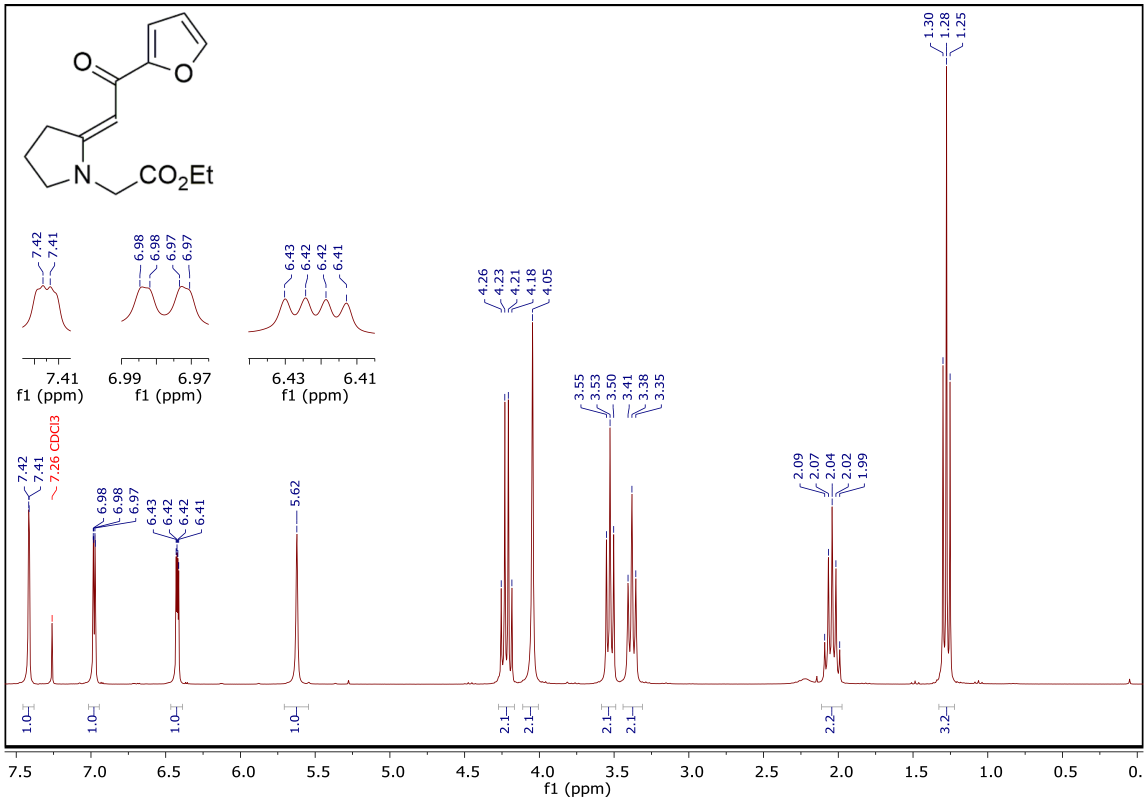

^{13}C NMR spectrum of (*E*)-ethyl 2-{2-[2-(furan-2-yl)-2-oxoethylidene]pyrrolidin-1-yl}acetate (15u) (75 MHz, $CDCl_3$)

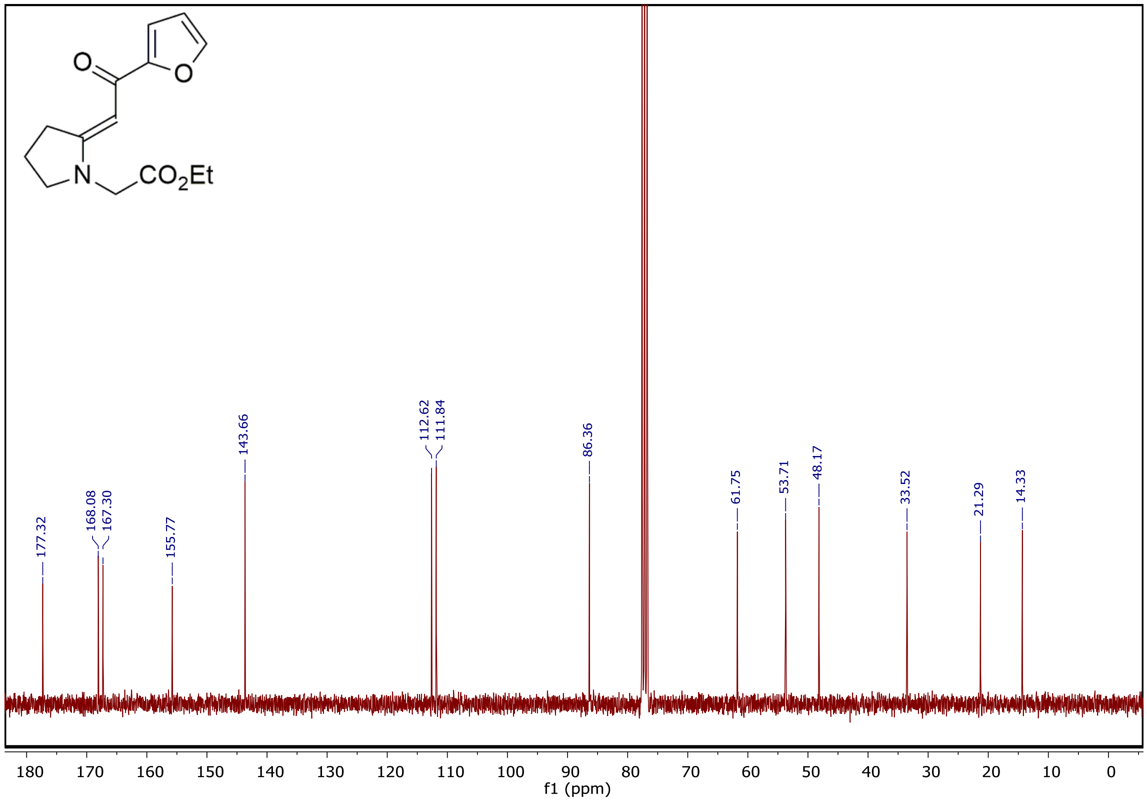

^{1}H NMR spectrum of (*E*)-ethyl 2-{2-[2-thiophen-2-yl)-2-oxoethylidene]pyrrolidin-1-yl}acetate (15v) (300 MHz, $CDCl_3$)

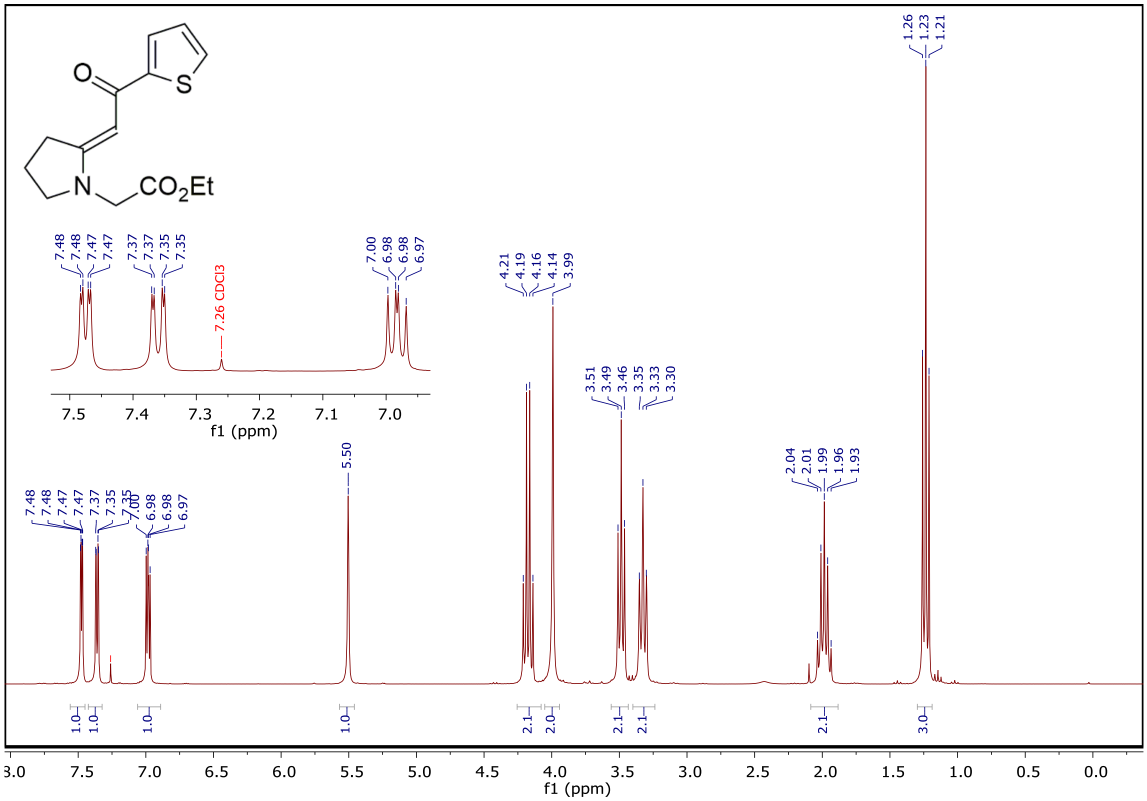

^{13}C NMR spectrum of (*E*)-ethyl 2-{2-[2-thiophen-2-yl)-2-oxoethylidene]pyrrolidin-1-yl}acetate (15v) (75 MHz, $CDCl_3$)

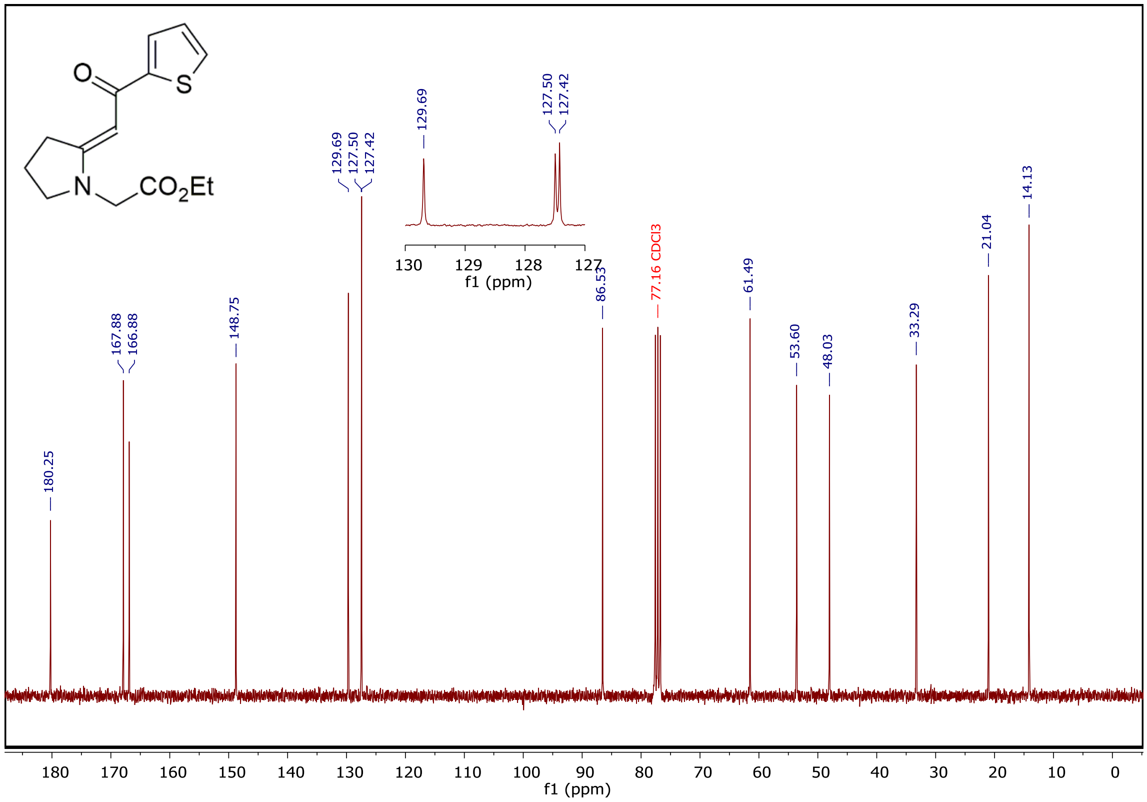

^{1}H NMR spectrum of (*E*)-ethyl 2-{2-[2-(benzofuran-2-yl)-2-oxoethylidene]pyrrolidin-1-yl}acetate (15w) (400 MHz, $CDCl_3$)

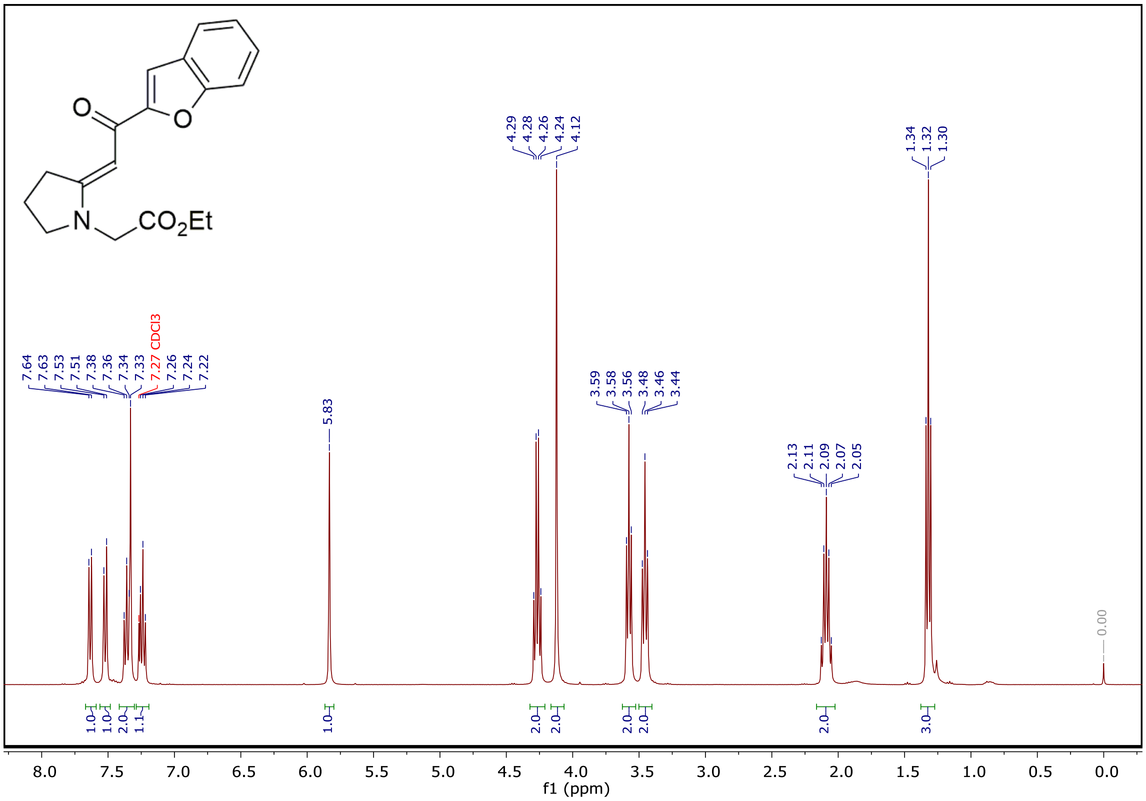

^{13}C NMR spectrum of (E)-ethyl 2-{2-[2-(benzofuran-2-yl)-2-oxoethylidene]pyrrolidin-1-yl}acetate (15w) (101 MHz, $CDCl_3$)

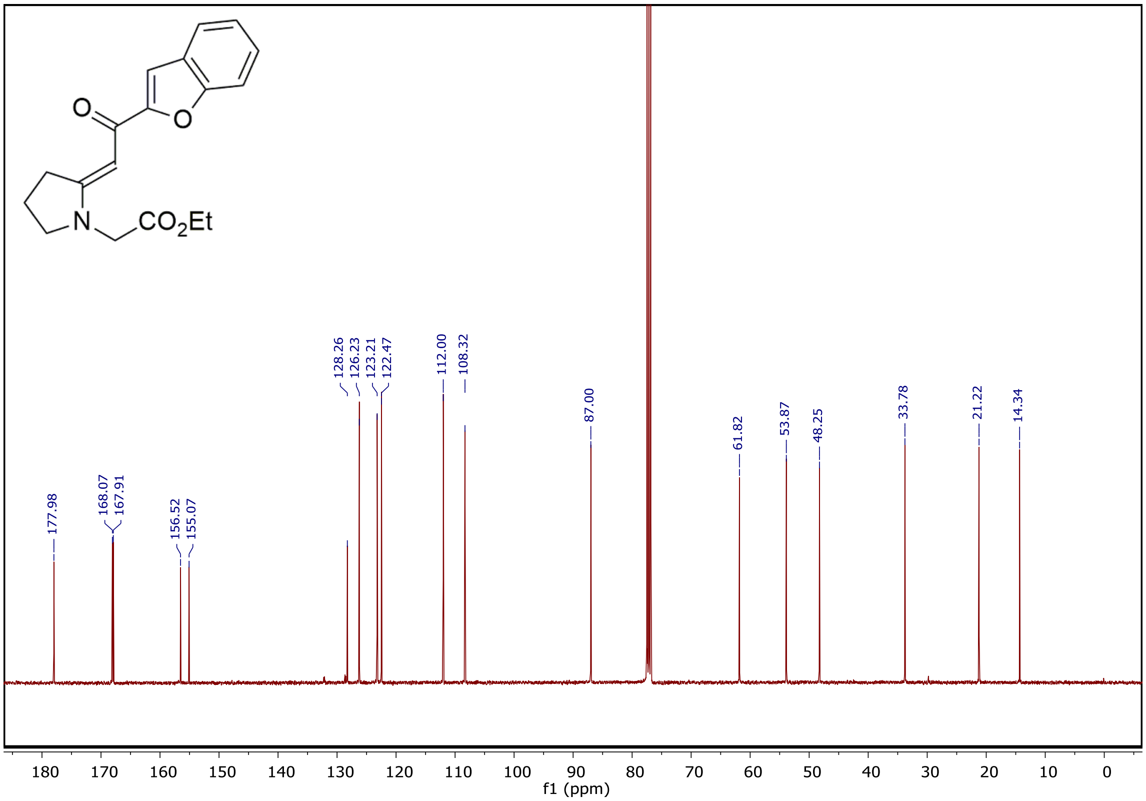

^{1}H NMR spectrum of (*E*)-ethyl 2-{2-[2-oxo-2-(1-tosyl-1*H*-indol-3-yl)ethylidene]pyrrolidin-1-yl}acetate (15x) (300 MHz, $CDCl_3$)

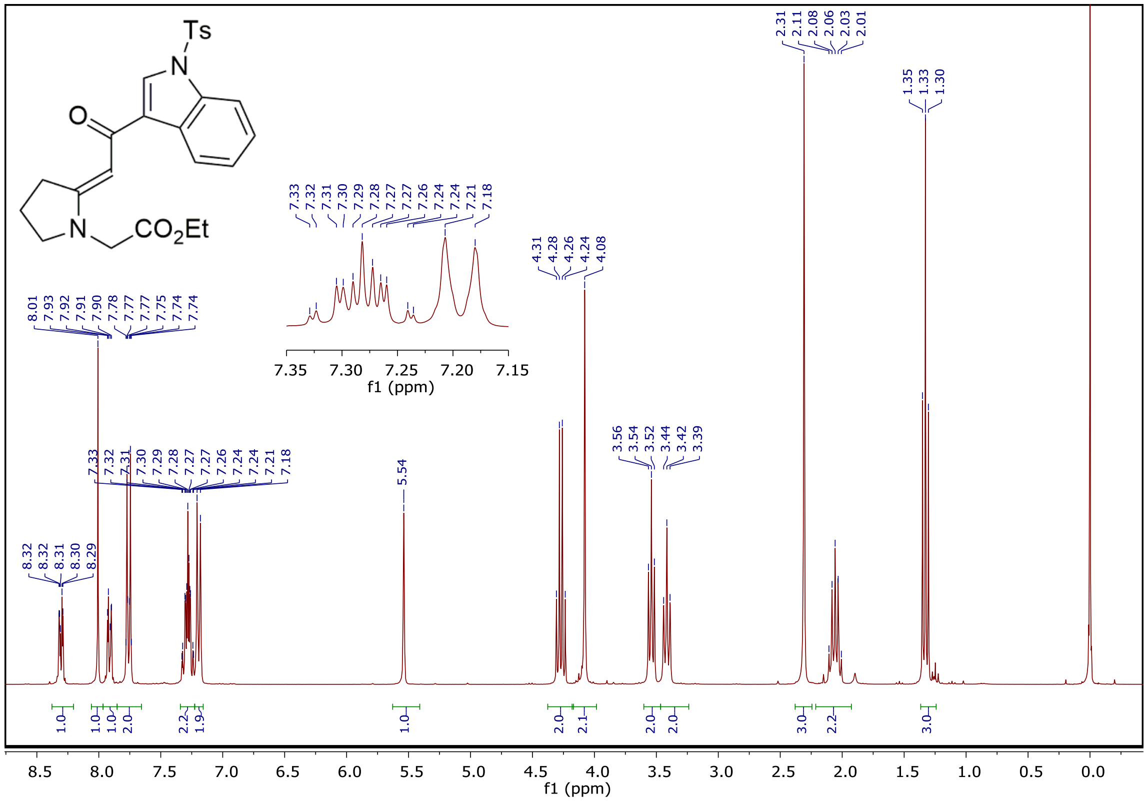

^{13}C NMR spectrum of (*E*)-ethyl 2-{2-[2-oxo-2-(1-tosyl-1*H*-indol-3-yl)ethylidene]pyrrolidin-1-yl}acetate (15x) (75 MHz, $CDCl_3$)

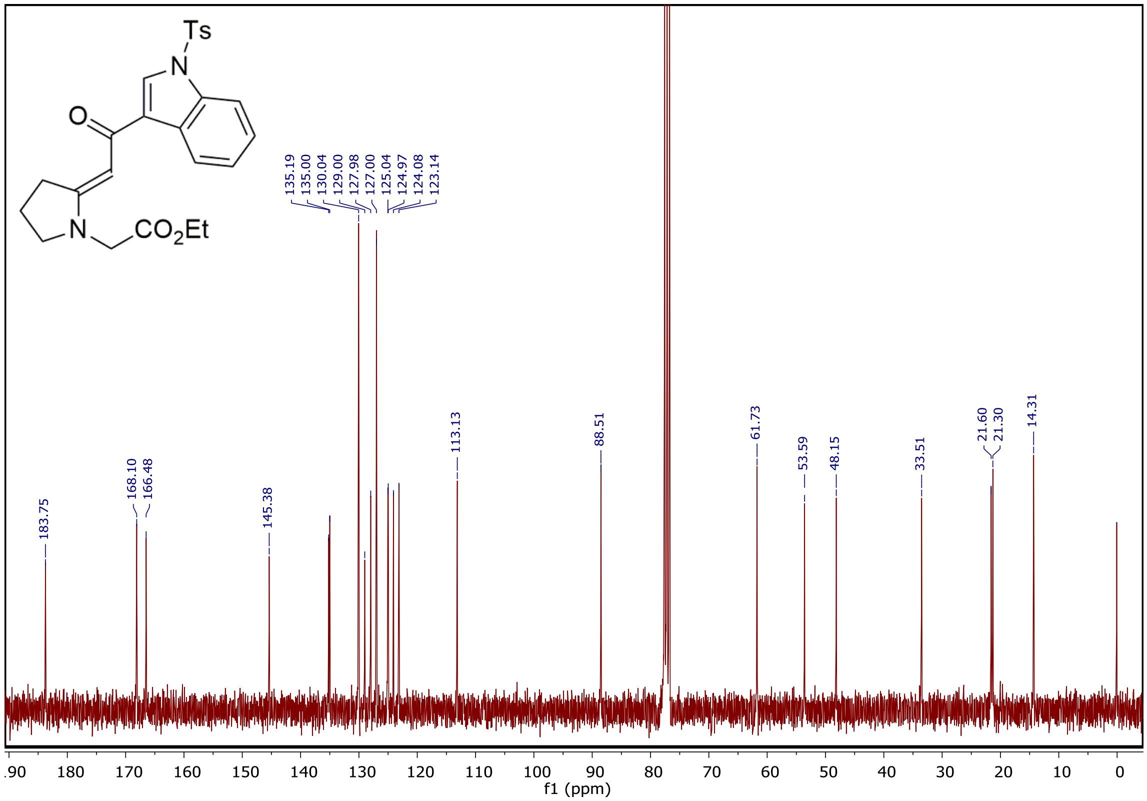

^{1}H NMR spectrum of (*E*)-ethyl 2-{2-[2-(*tert*-butyl)-2-oxoethylidene]pyrrolidin-1-yl}acetate (15y) (300 MHz, $CDCl_3$)

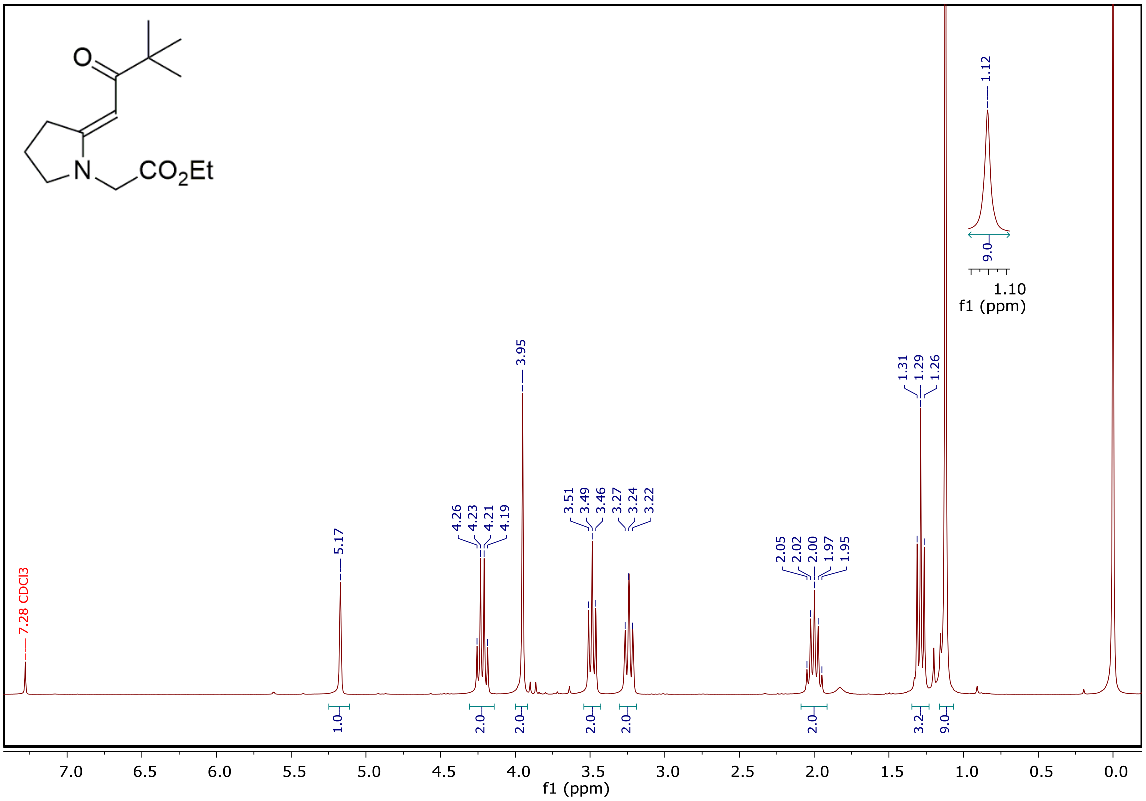

^{13}C NMR spectrum of (*E*)-ethyl 2-{2-[2-(*tert*-butyl)-2-oxoethylidene]pyrrolidin-1-yl}acetate (15y) (75 MHz, $CDCl_3$)

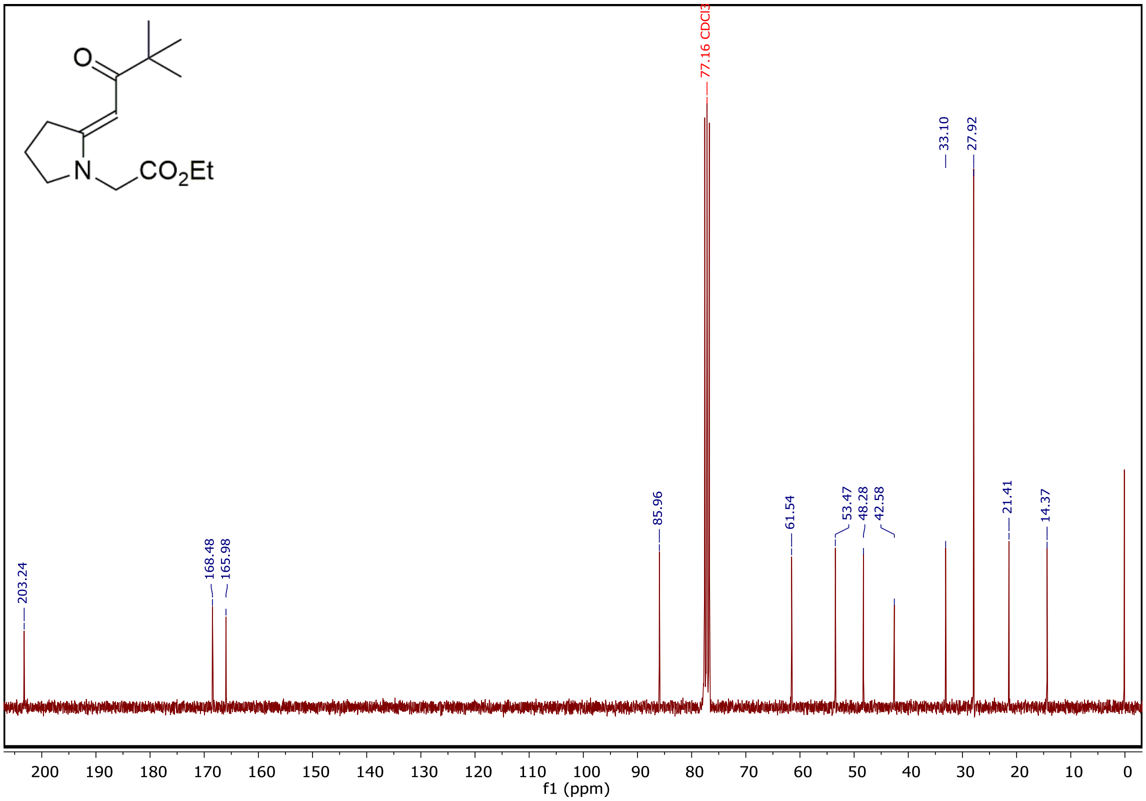

^{1}H NMR spectrum of ethyl 6-phenyl-2,3-dihydro-1*H*-pyrrolizine-5-carboxylate (19a) (300 MHz, $CDCl_3$)

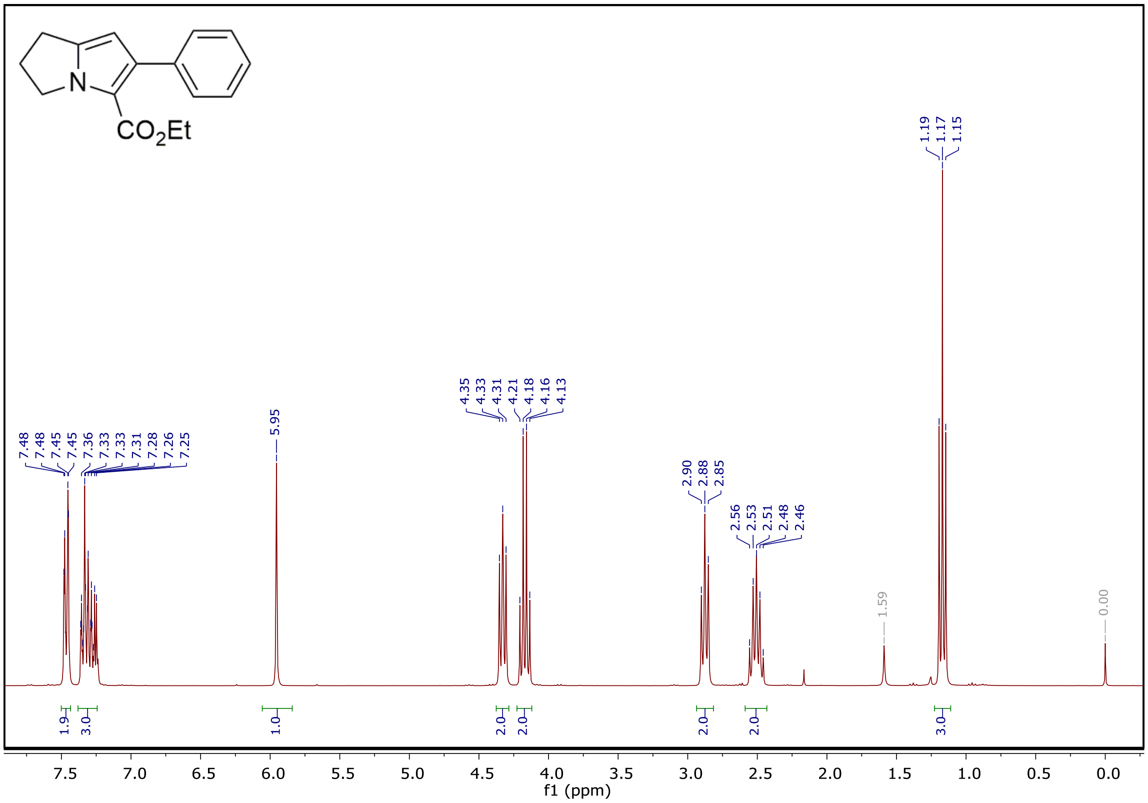

^{13}C NMR spectrum of ethyl 6-phenyl-2,3-dihydro-1*H*-pyrrolizine-5-carboxylate (19a) (75 MHz, $CDCl_3$)

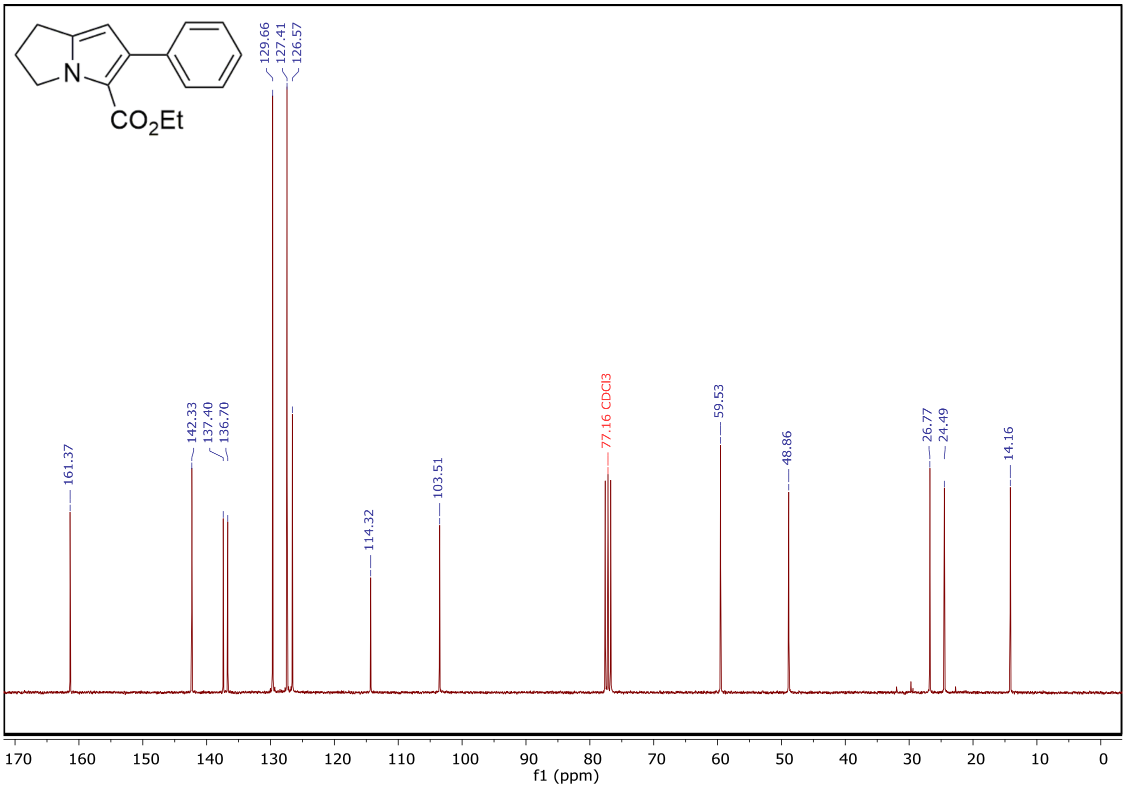

COSY NMR spectrum of ethyl 6-phenyl-2,3-dihydro-1*H*-pyrrolizine-5-carboxylate (19a) (300 MHz, $CDCl_3$)

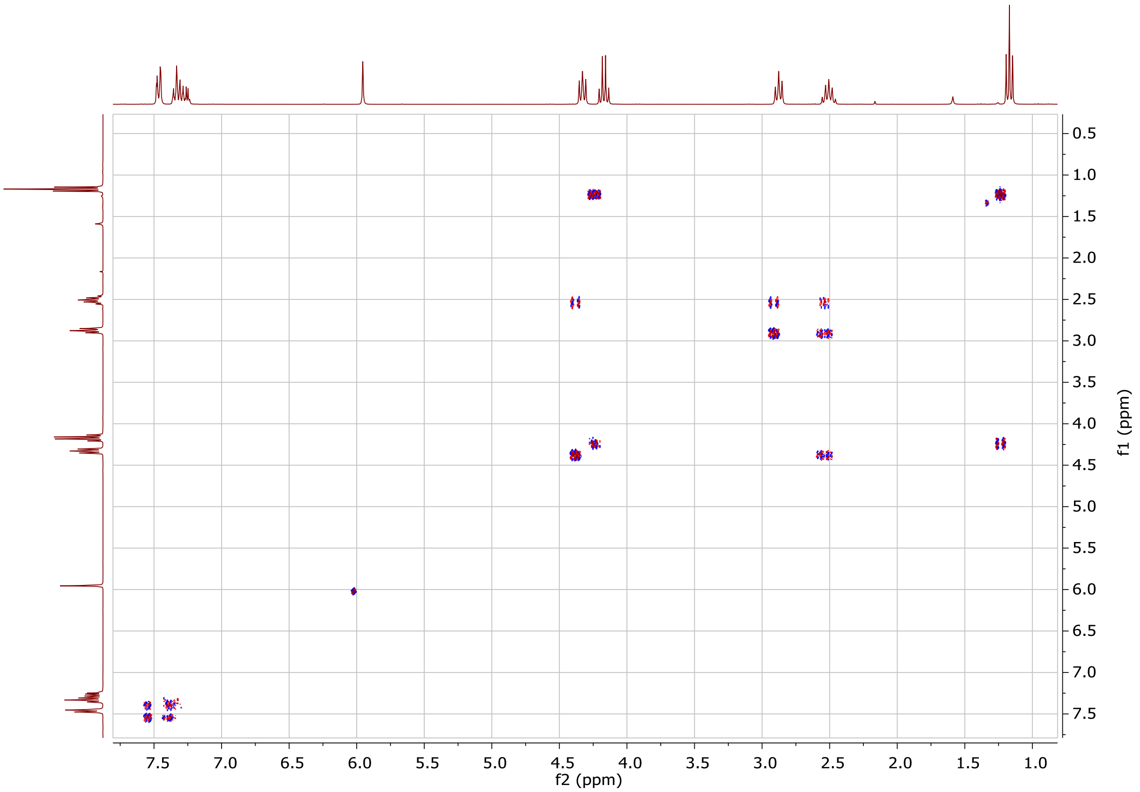

HSQC NMR spectrum of ethyl 6-phenyl-2,3-dihydro-1*H*-pyrrolizine-5-carboxylate (19a) ($CDCl_3$)

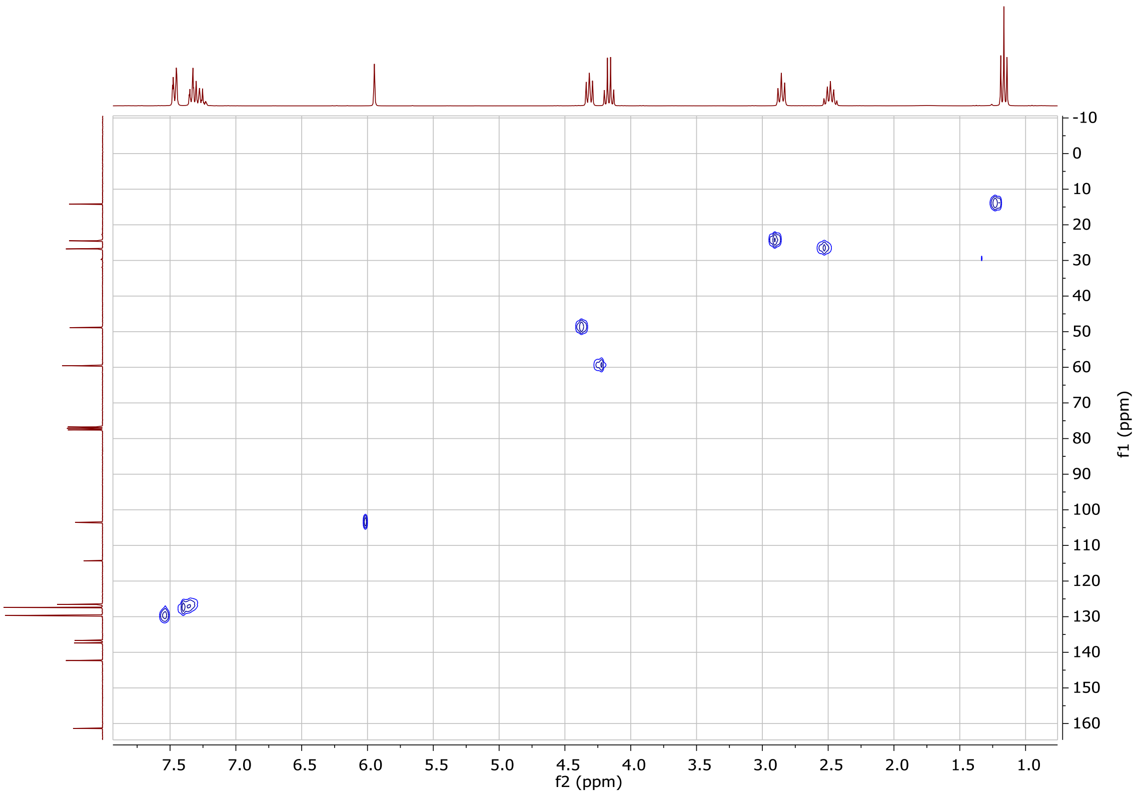

^{1}H NMR spectrum of ethyl 6-(4-nitrophenyl)-2,3-dihydro-1*H*-pyrrolizine-5-carboxylate (19b) (300 MHz, $CDCl_3$)

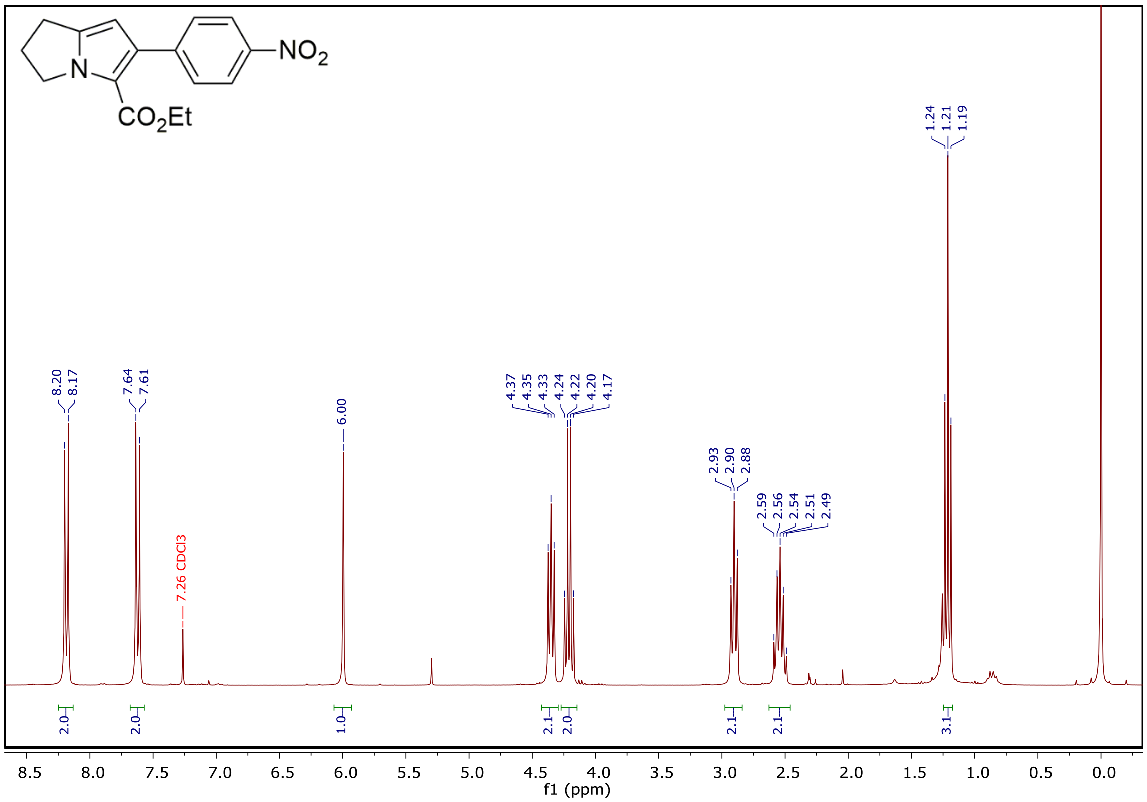

^{13}C NMR spectrum of ethyl 6-(4-nitrophenyl)-2,3-dihydro-1*H*-pyrrolizine-5-carboxylate (19b) (75 MHz, $CDCl_3$)

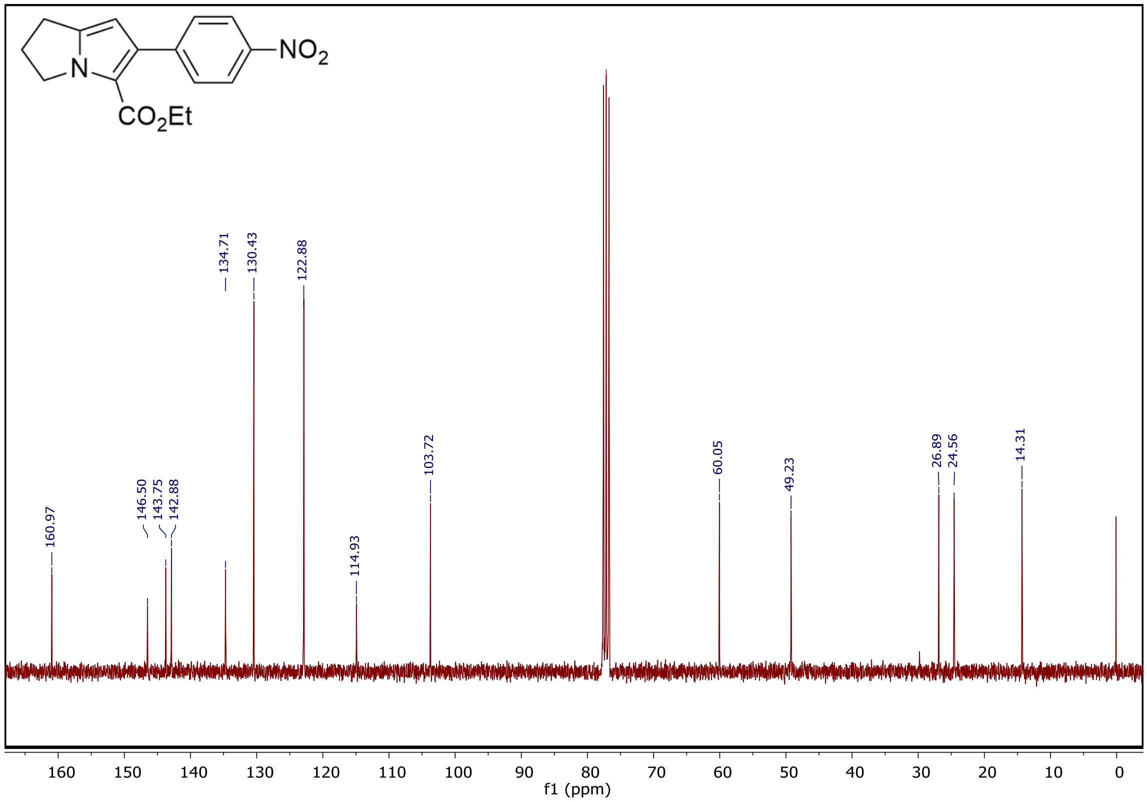

^{1}H NMR spectrum of ethyl 6-(4-cyanophenyl)-2,3-dihydro-1*H*-pyrrolizine-5-carboxylate (19c) (300 MHz, $CDCl_3$)

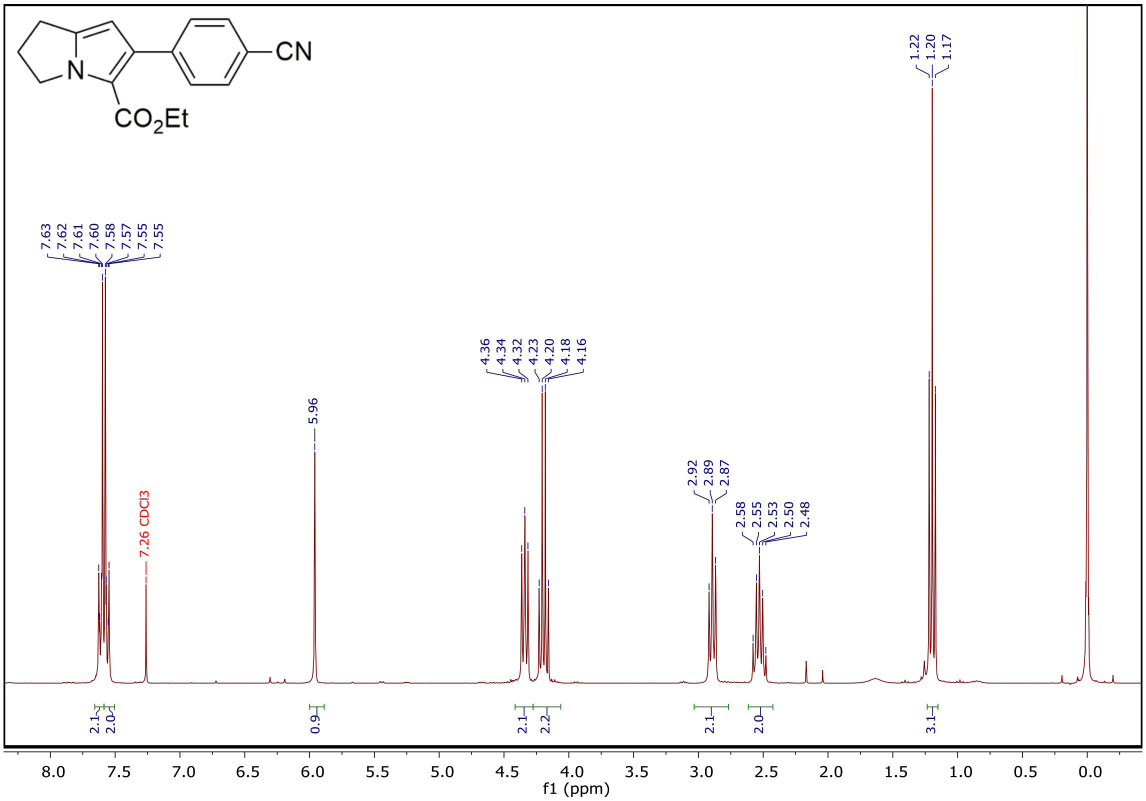

^{13}C NMR spectrum of ethyl 6-(4-cyanophenyl)-2,3-dihydro-1*H*-pyrrolizine-5-carboxylate (19c) (75 MHz, $CDCl_3$)

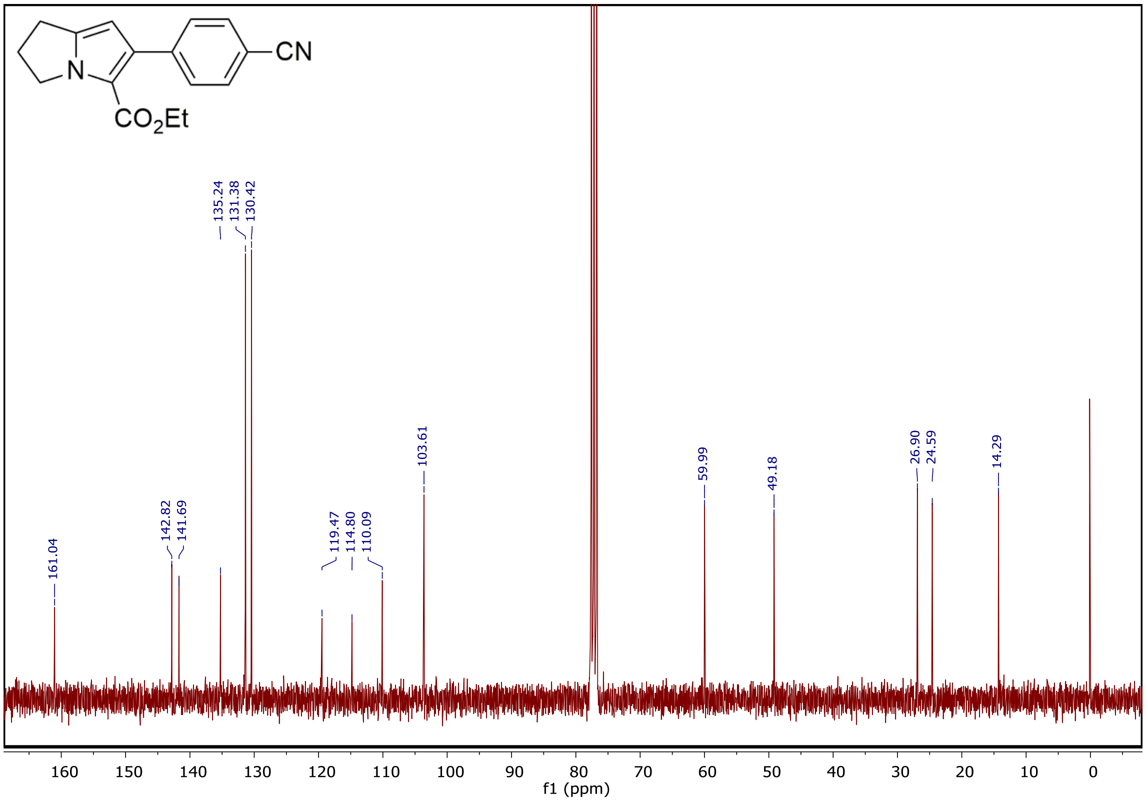

^{1}H NMR spectrum of ethyl 6-(4-fluorophenyl)-2,3-dihydro-1*H*-pyrrolizine-5-carboxylate (19d) (300 MHz, $CDCl_3$)

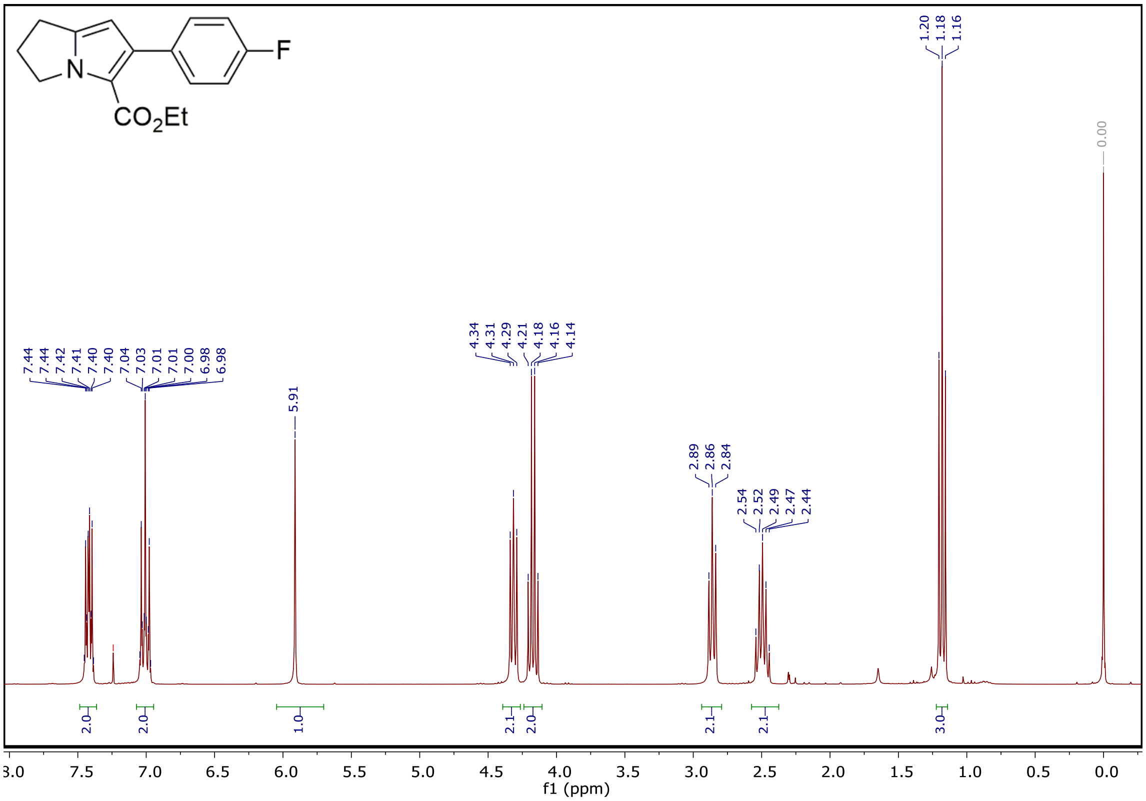

^{13}C NMR spectrum of ethyl 6-(4-fluorophenyl)-2,3-dihydro-1*H*-pyrrolizine-5-carboxylate (19d) (75 MHz, $CDCl_3$)

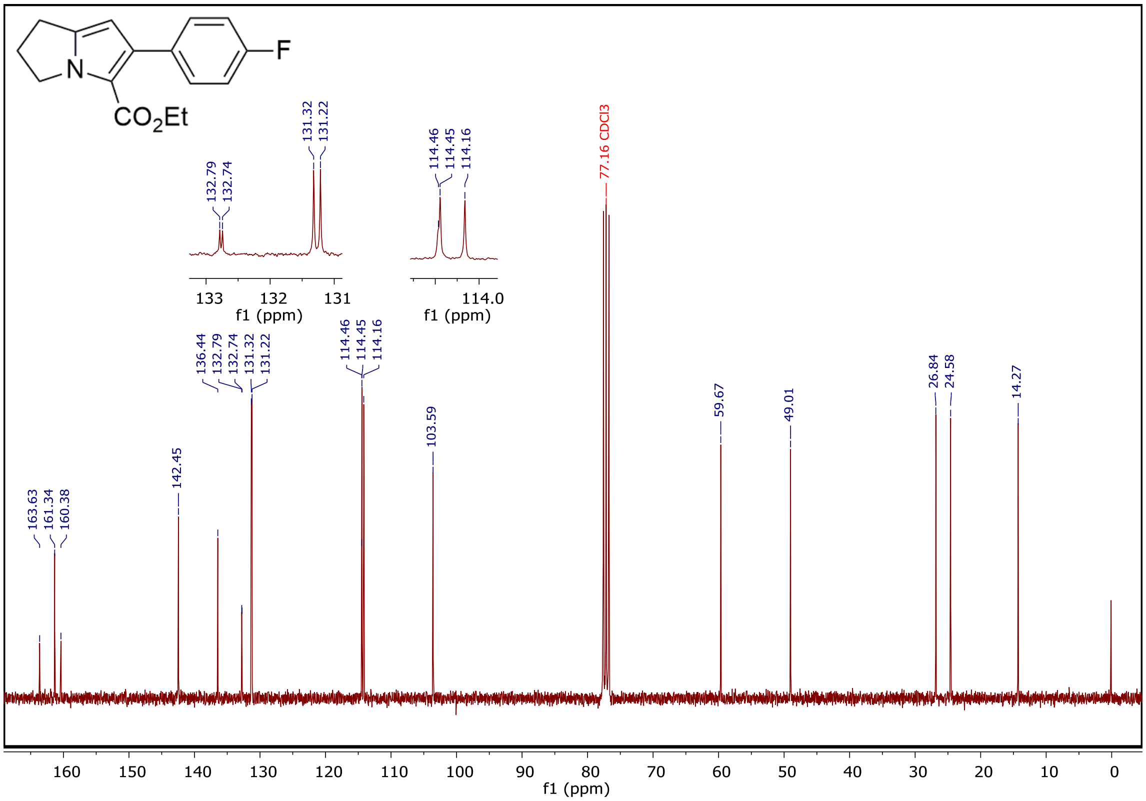

^{1}H NMR spectrum of ethyl 6-(4-bromophenyl)-2,3-dihydro-1*H*-pyrrolizine-5-carboxylate (19e) (300 MHz, $CDCl_3$)

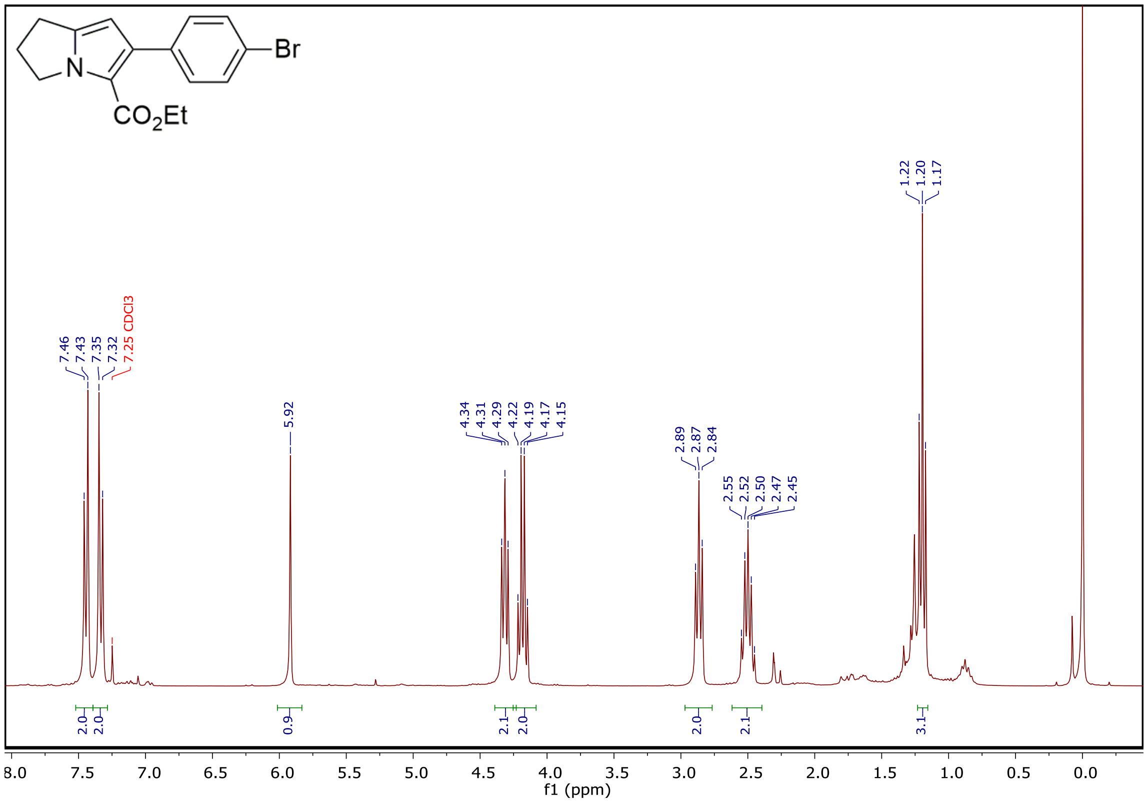

^{13}C NMR spectrum of ethyl 6-(4-bromophenyl)-2,3-dihydro-1*H*-pyrrolizine-5-carboxylate (19e) (75 MHz, $CDCl_3$)

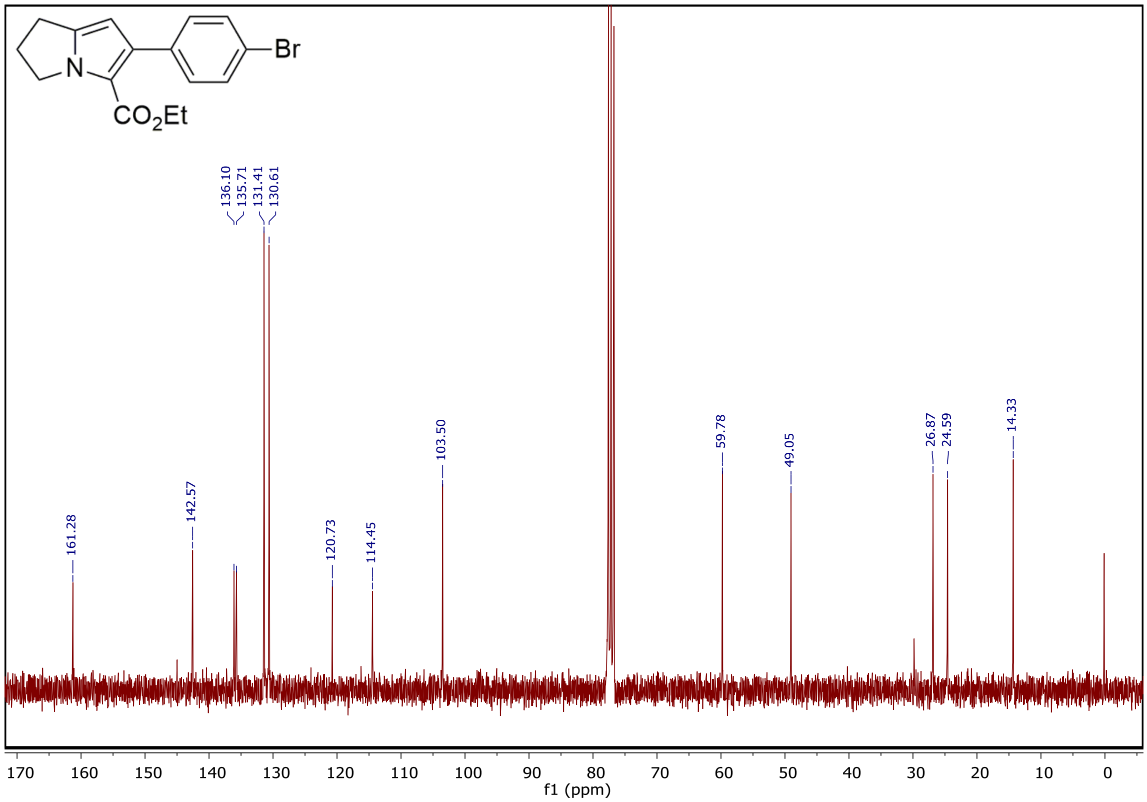

^{1}H NMR spectrum of ethyl 6-(4-methylphenyl)-2,3-dihydro-1*H*-pyrrolizine-5-carboxylate (19f) (300 MHz, $CDCl_3$)

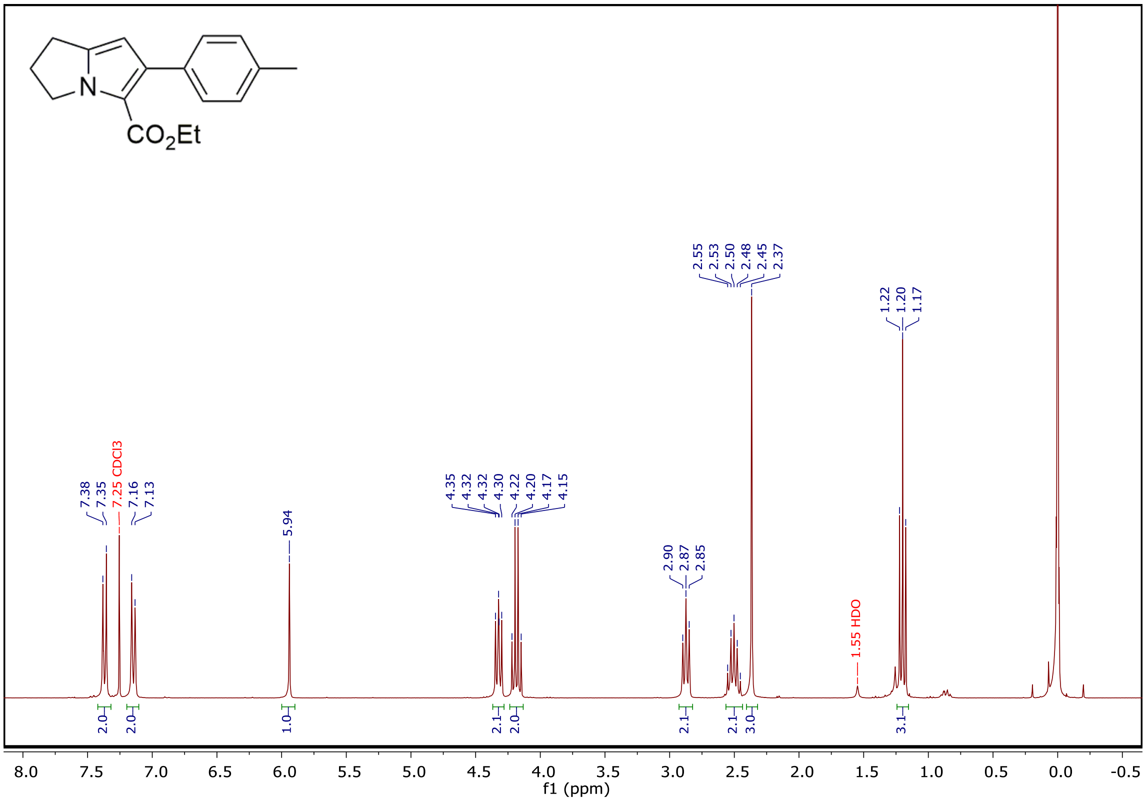

^{13}C NMR spectrum of ethyl 6-(4-methylphenyl)-2,3-dihydro-1*H*-pyrrolizine-5-carboxylate (19f) (75 MHz, $CDCl_3$)

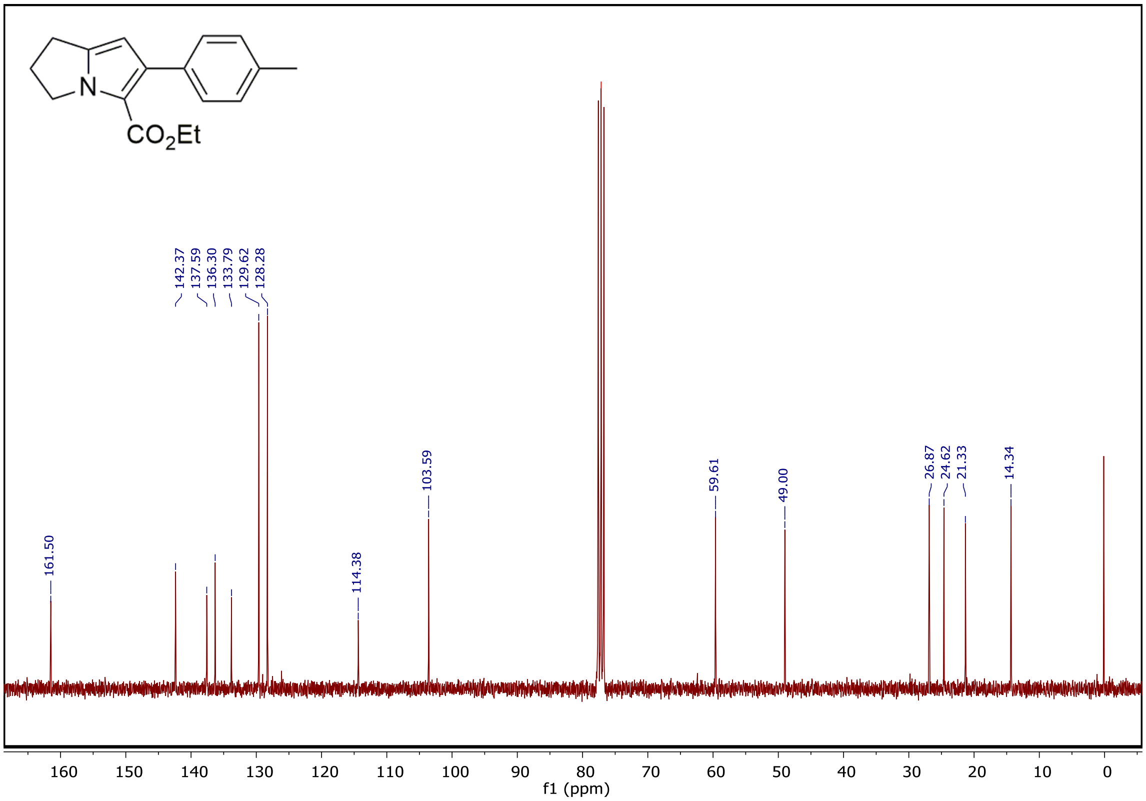

^{1}H NMR spectrum of ethyl 6-(4-methoxyphenyl)-2,3-dihydro-1*H*-pyrrolizine-5-carboxylate (19g) (300 MHz, $CDCl_3$)

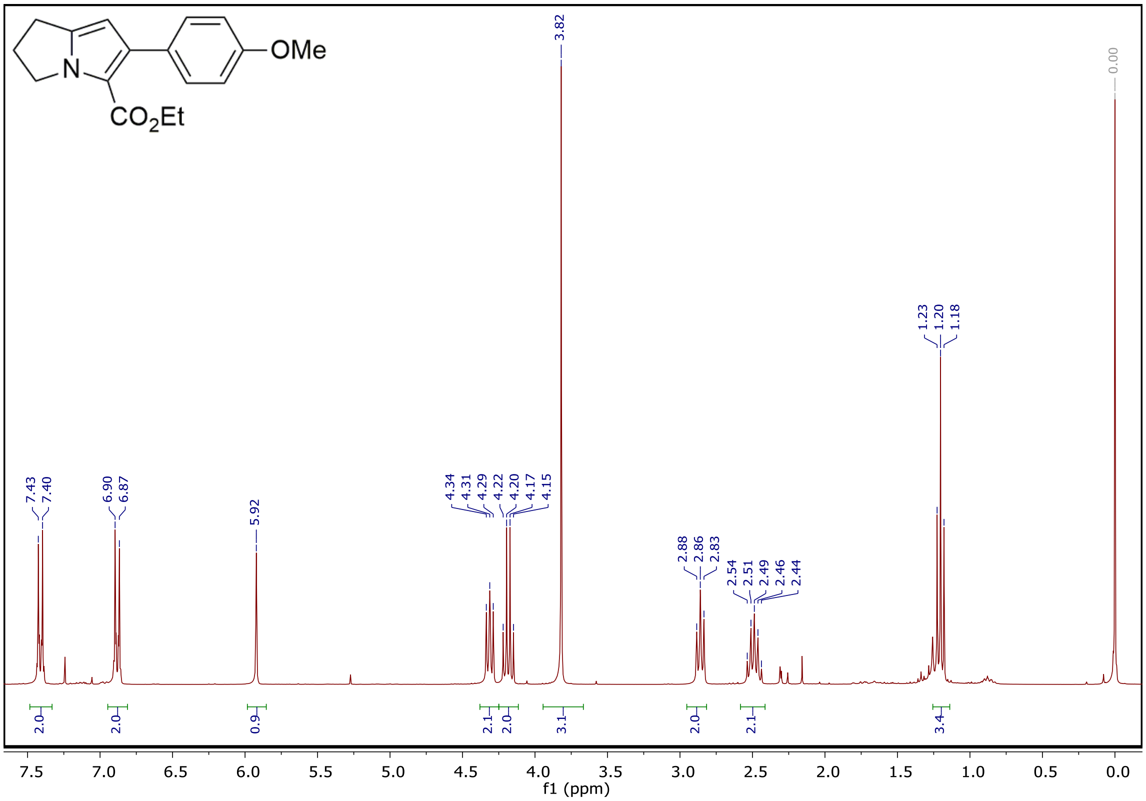

^{13}C NMR spectrum of ethyl 6-(4-methoxyphenyl)-2,3-dihydro-1*H*-pyrrolizine-5-carboxylate (19g) (75 MHz, $CDCl_3$)

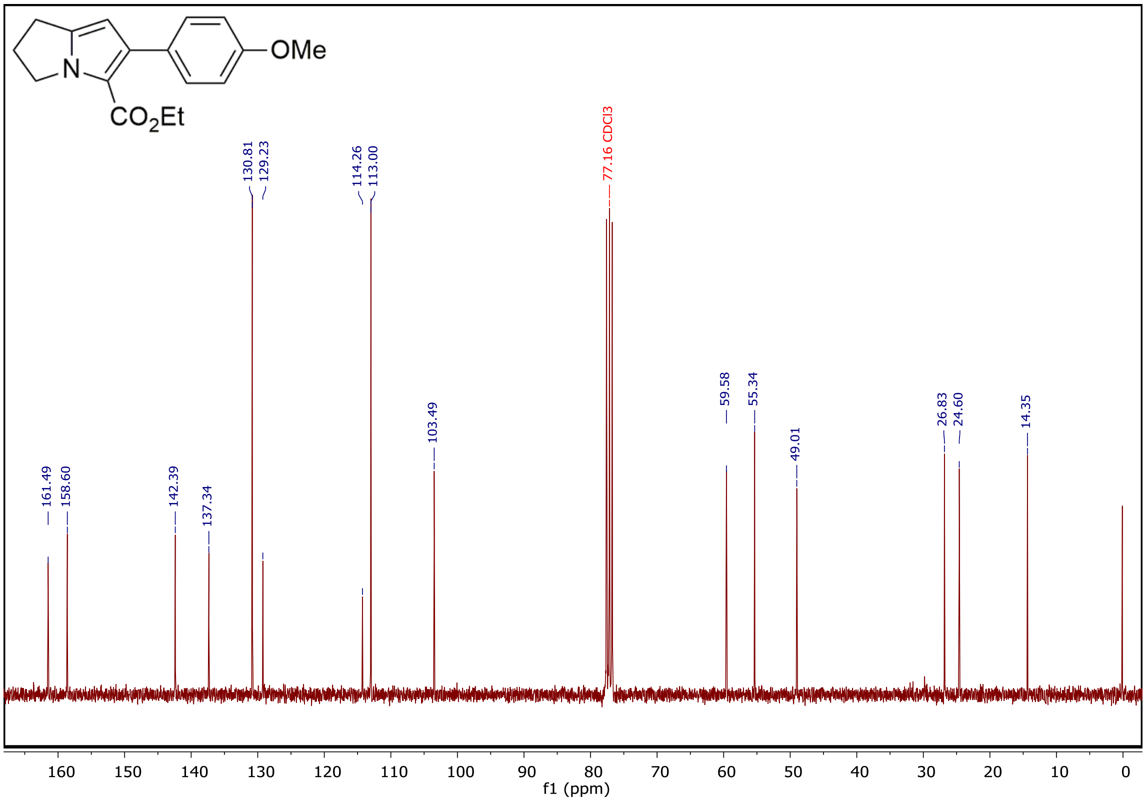

^{1}H NMR spectrum of ethyl 6-(3-nitrophenyl)-2,3-dihydro-1*H*-pyrrolizine-5-carboxylate (19h) (300 MHz, $CDCl_3$)

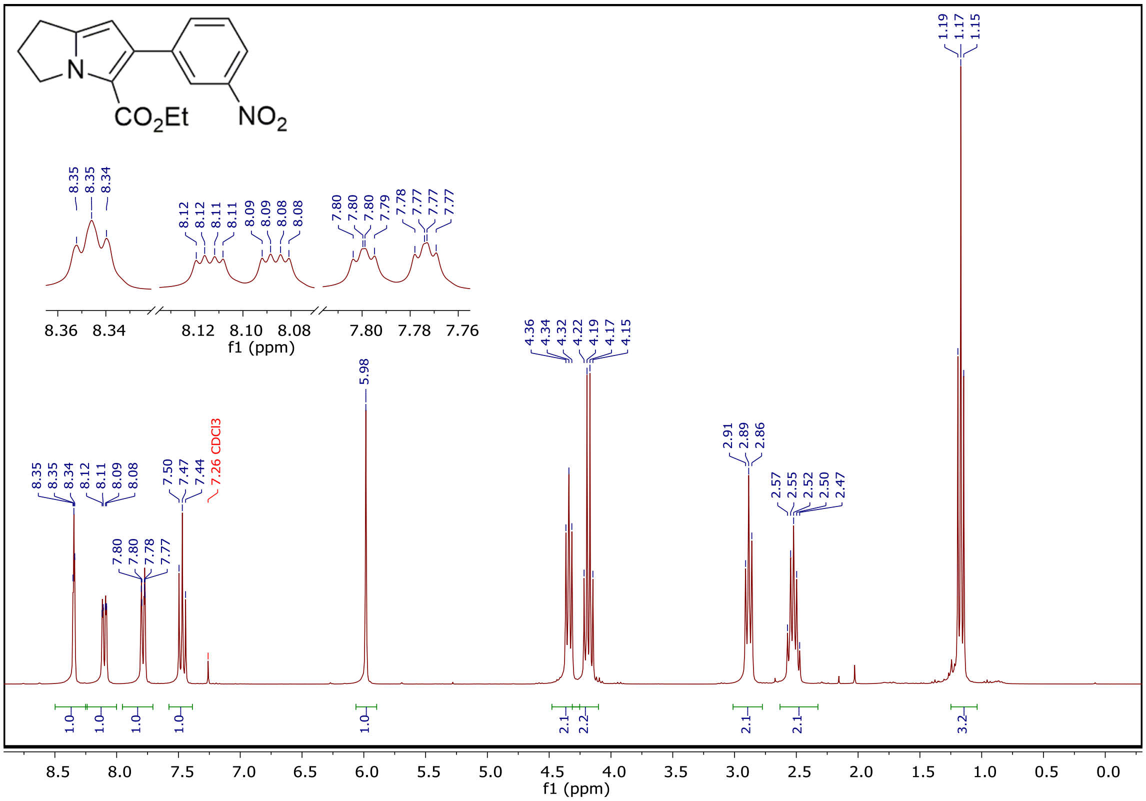

^{13}C NMR spectrum of ethyl 6-(3-nitrophenyl)-2,3-dihydro-1*H*-pyrrolizine-5-carboxylate (19h) (75 MHz, $CDCl_3$)

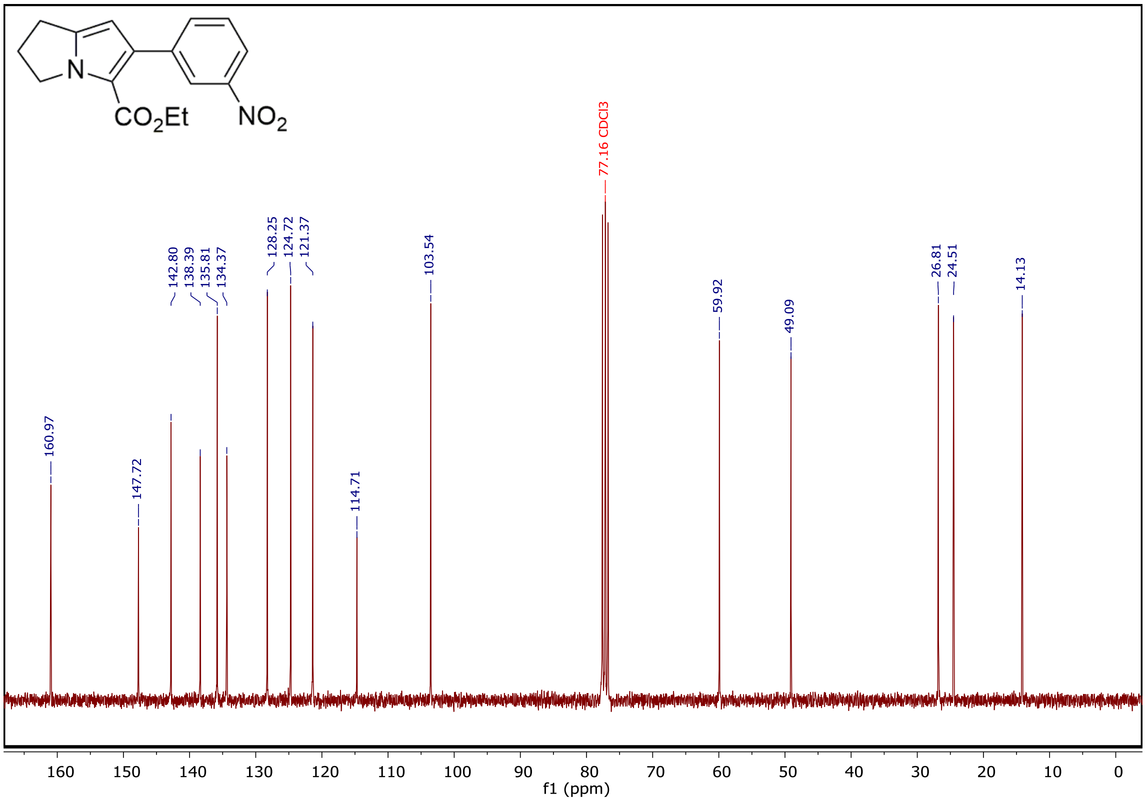

^{1}H NMR spectrum of ethyl 6-(3-methoxyphenyl)-2,3-dihydro-1*H*-pyrrolizine-5-carboxylate (19i) (400 MHz, $CDCl_3$)

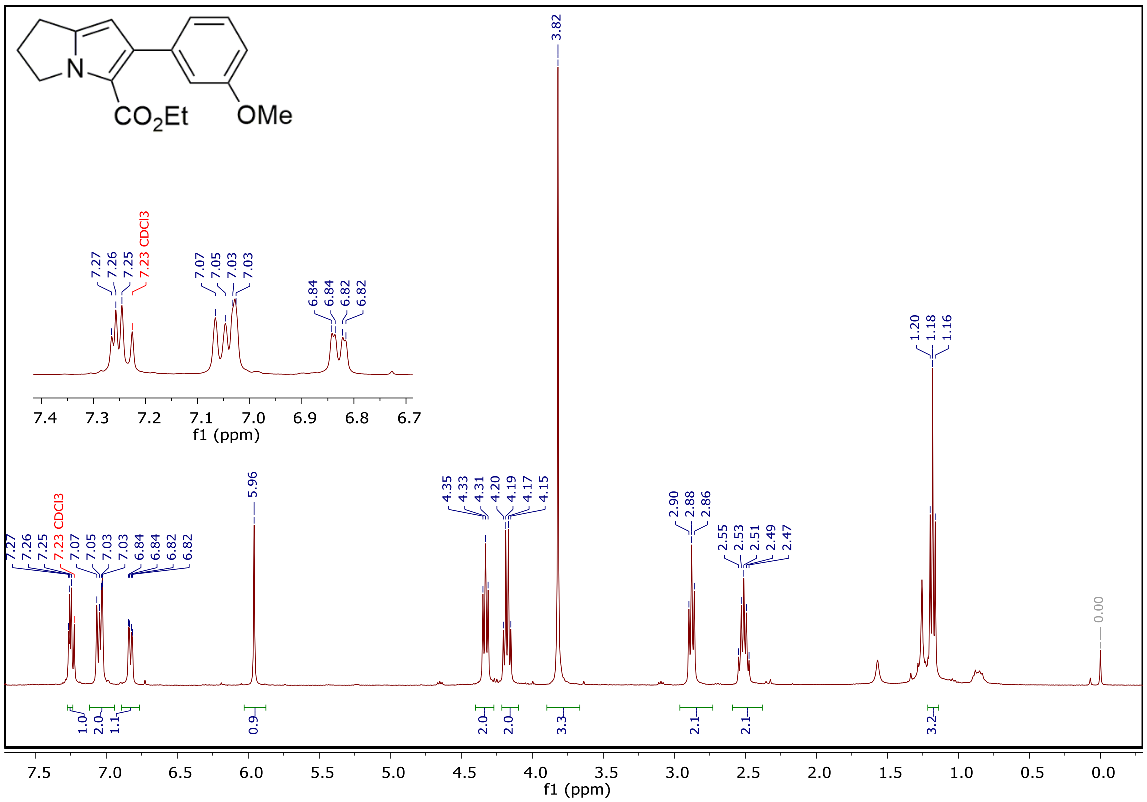

^{13}C NMR spectrum of ethyl 6-(3-methoxyphenyl)-2,3-dihydro-1*H*-pyrrolizine-5-carboxylate (19i) (101 MHz, $CDCl_3$)

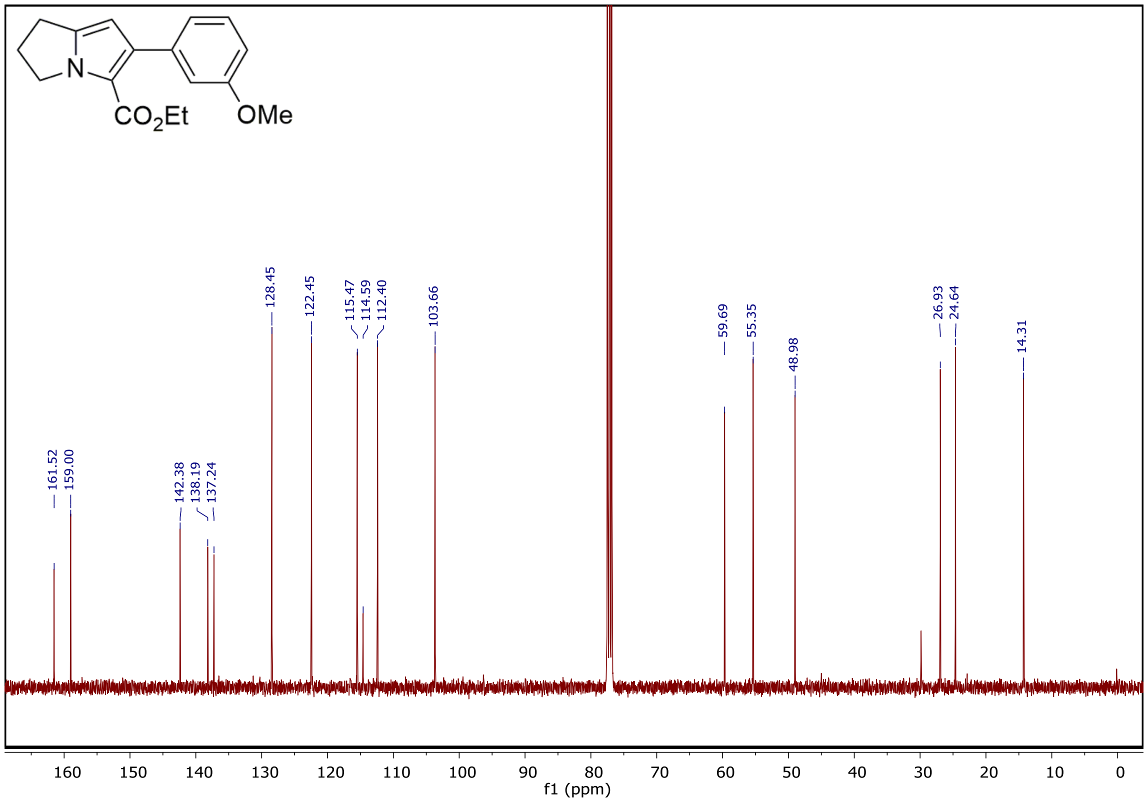

^{1}H NMR spectrum of ethyl 6-(3,4-dimethoxyphenyl)-2,3-dihydro-1*H*-pyrrolizine-5-carboxylate (19j) (400 MHz, CDCl3)

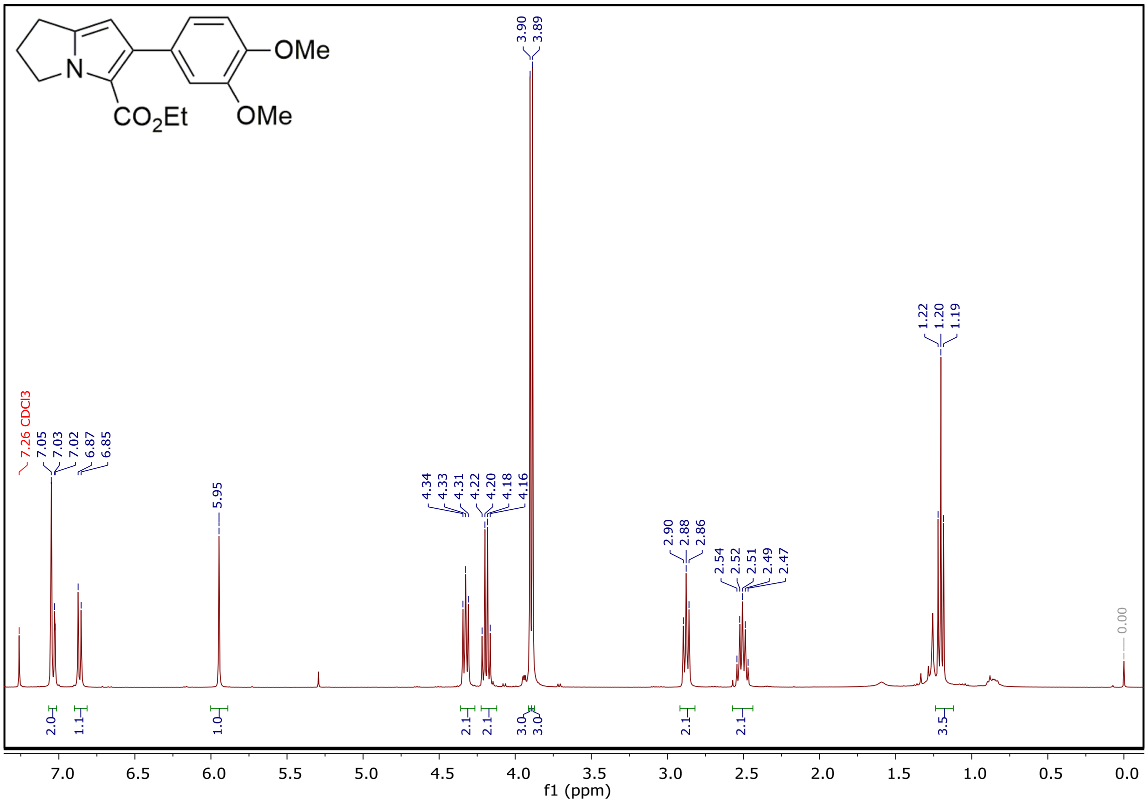

^{13}C NMR spectrum of ethyl 6-(3,4-dimethoxyphenyl)-2,3-dihydro-1*H*-pyrrolizine-5-carboxylate (19j) (101 MHz, CDCl3)

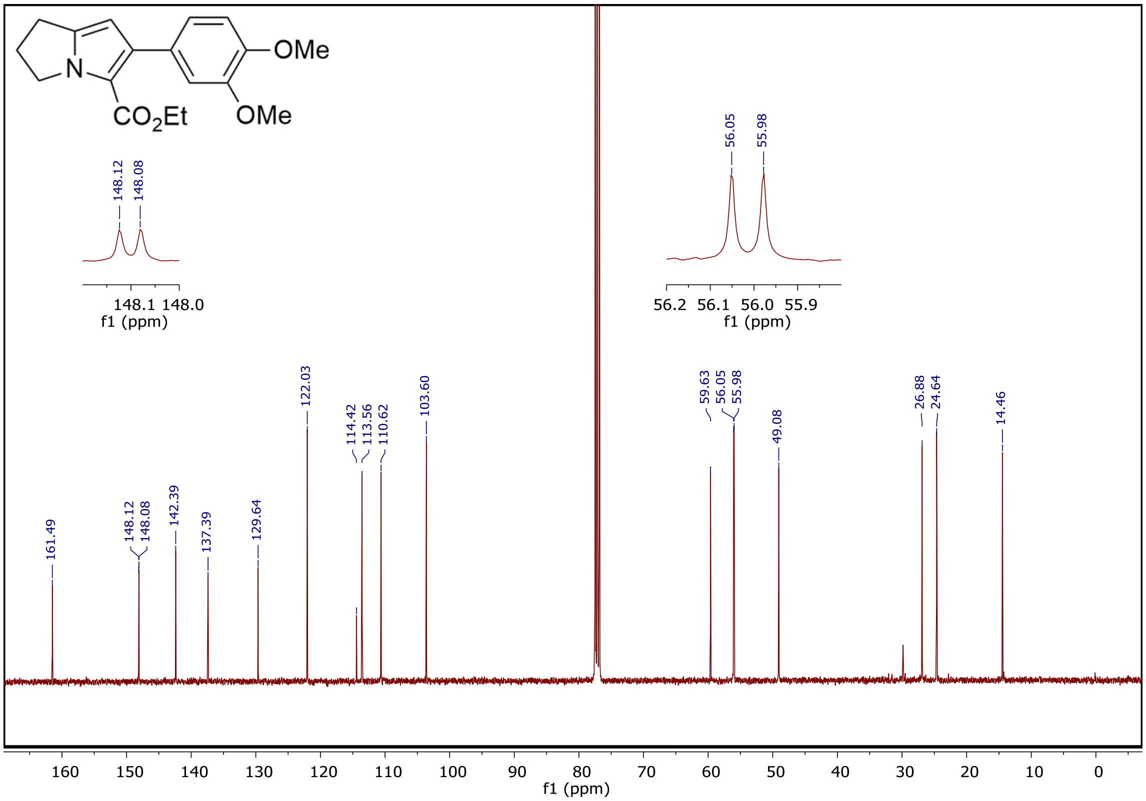

^{1}H NMR spectrum of ethyl 6-(2-bromophenyl)-2,3-dihydro-1*H*-pyrrolizine-5-carboxylate (19m) (300 MHz, $CDCl_3$)

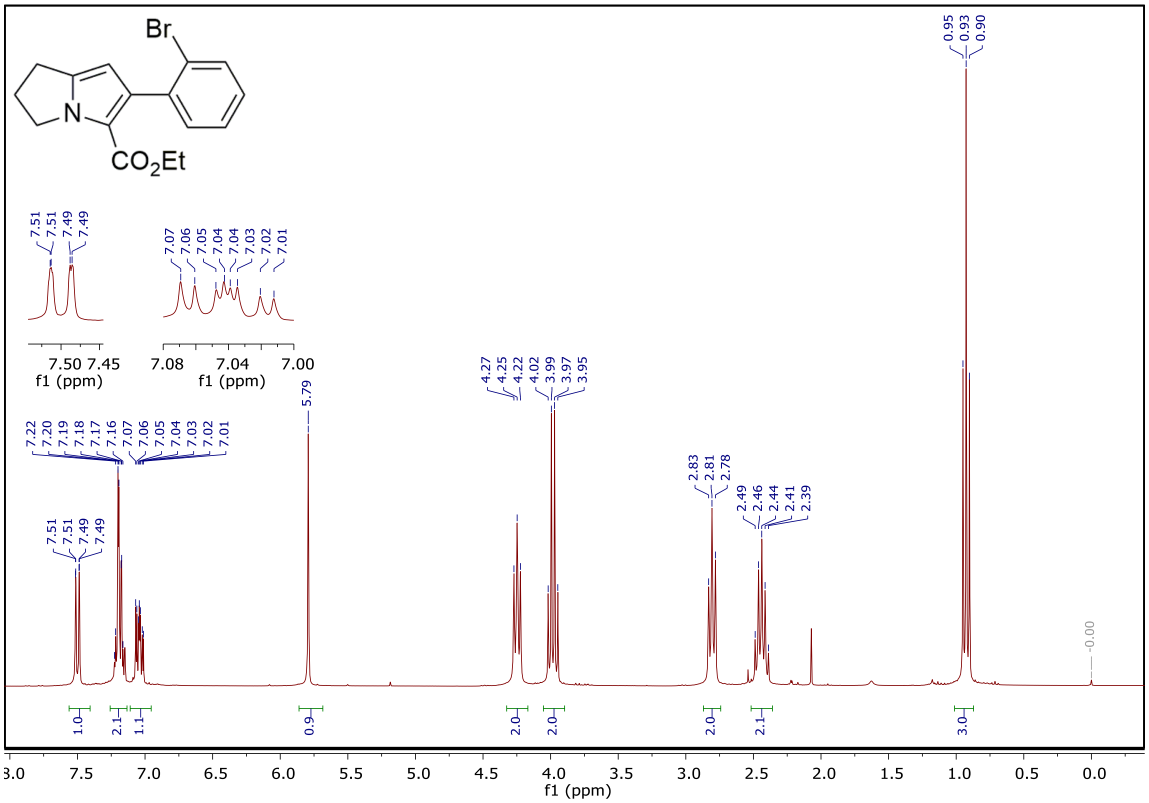

^{13}C NMR spectrum of ethyl 6-(2-bromophenyl)-2,3-dihydro-1*H*-pyrrolizine-5-carboxylate (19m) (75 MHz, $CDCl_3$)

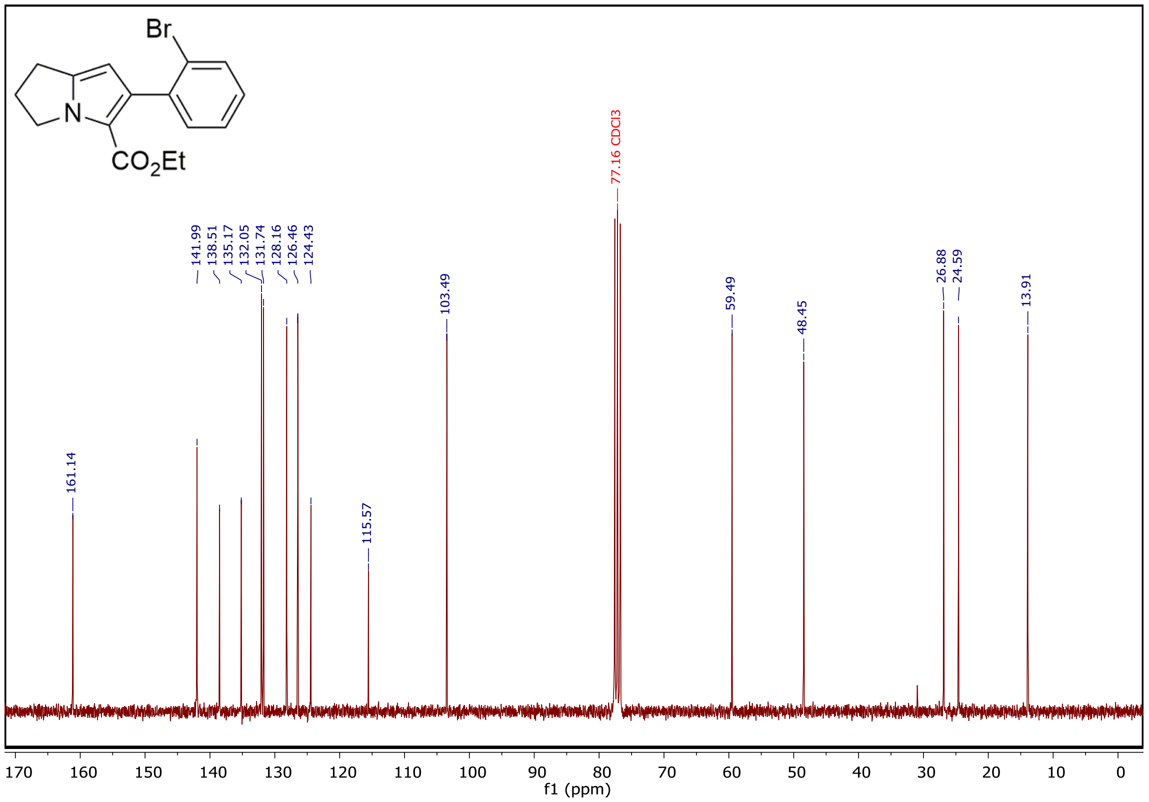

^{1}H NMR spectrum of ethyl 6-(2-bromo-4,5-dimethoxyphenyl)-2,3-dihydro-1*H*-pyrrolizine-5-carboxylate (19n) (400 MHz, $CDCl_3$)

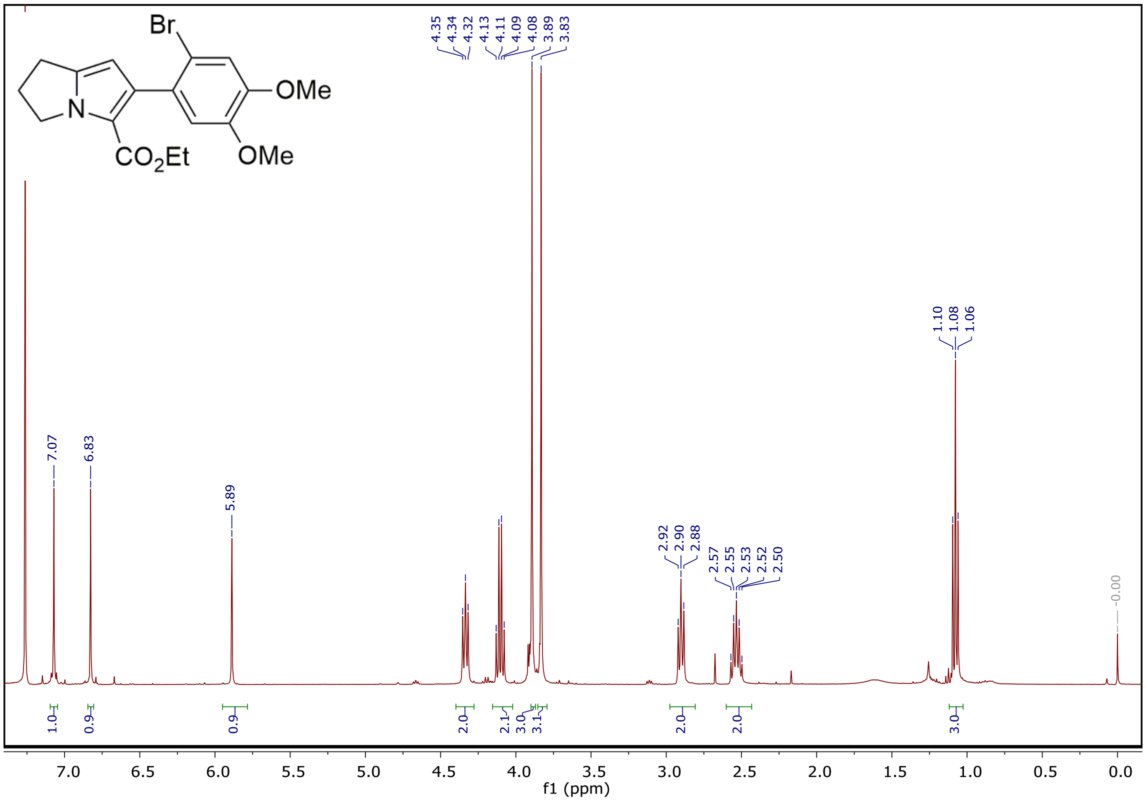

^{13}C NMR spectrum of ethyl 6-(2-bromo-4,5-dimethoxyphenyl)-2,3-dihydro-1*H*-pyrrolizine-5-carboxylate (19n) (101 MHz, $CDCl_3$)

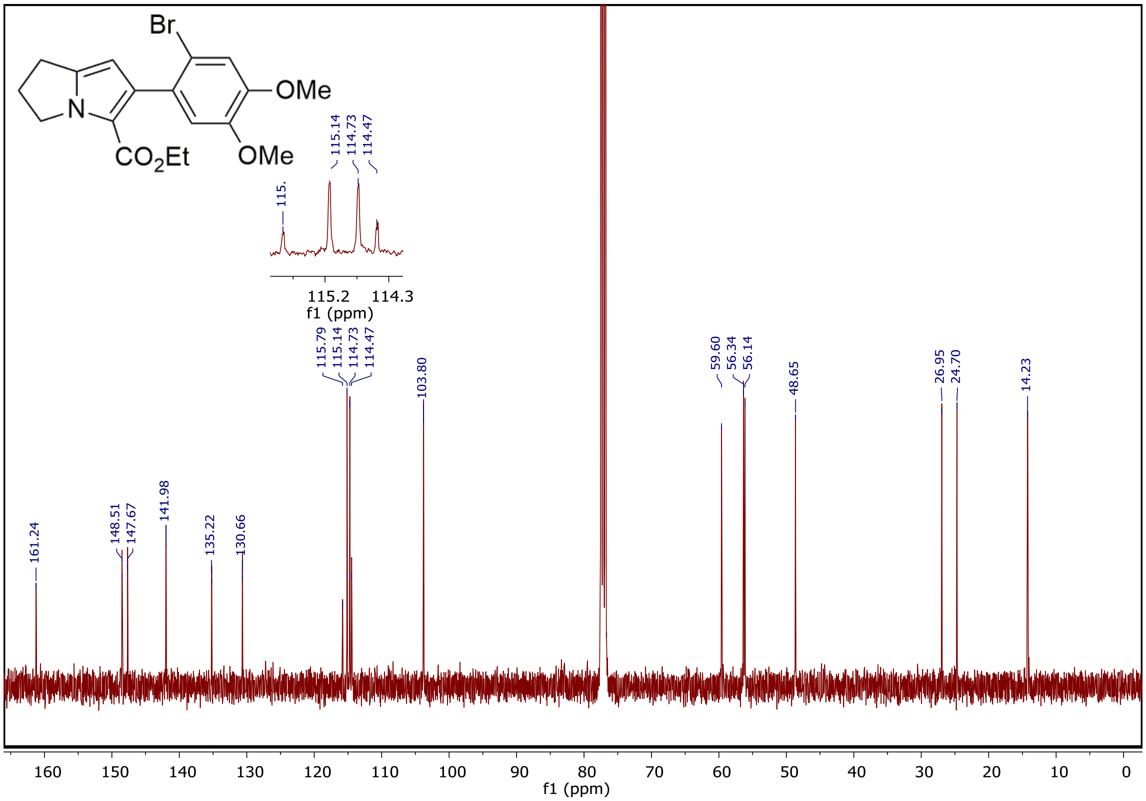

^{1}H NMR spectrum of ethyl 6-(2-chlorophenyl)-2,3-dihydro-1*H*-pyrrolizine-5-carboxylate (19o) (300 MHz, $CDCl_3$)

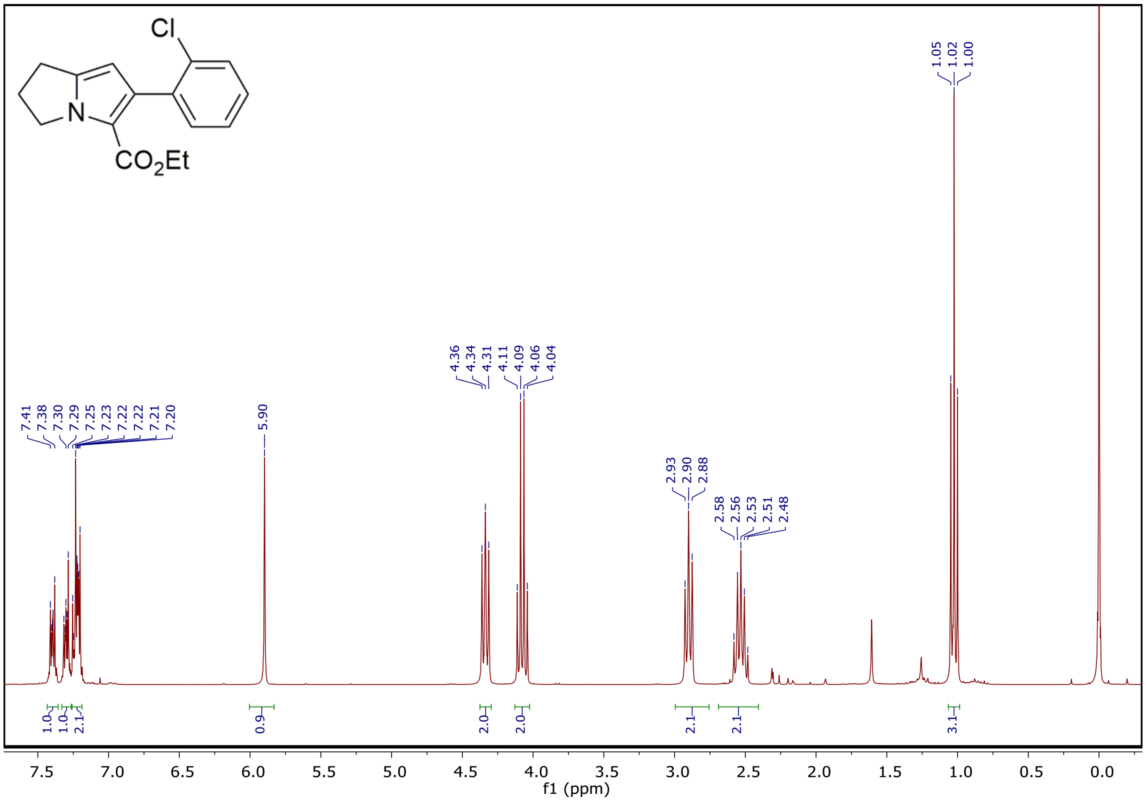

^{13}C NMR spectrum of ethyl 6-(2-chlorophenyl)-2,3-dihydro-1*H*-pyrrolizine-5-carboxylate (19o) (75 MHz, $CDCl_3$)

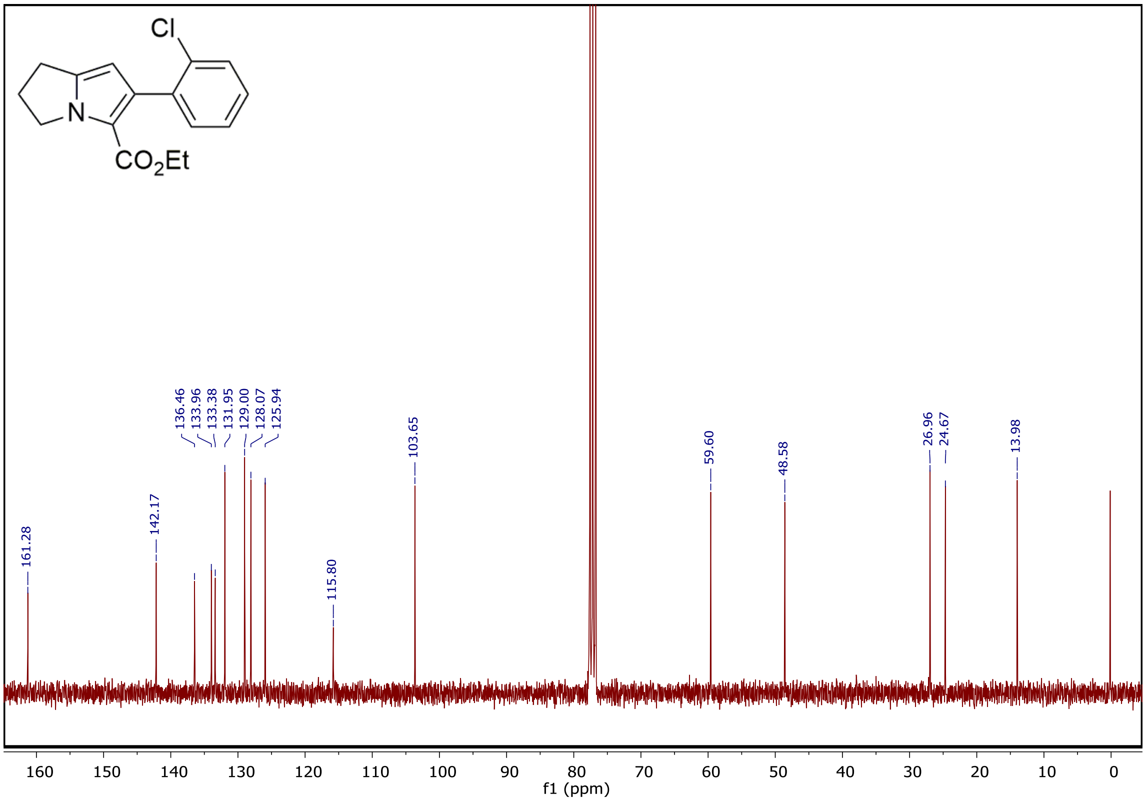

^{1}H NMR spectrum of ethyl 6-(2-methoxyphenyl)-2,3-dihydro-1*H*-pyrrolizine-5-carboxylate (19p) (300 MHz, $CDCl_3$)

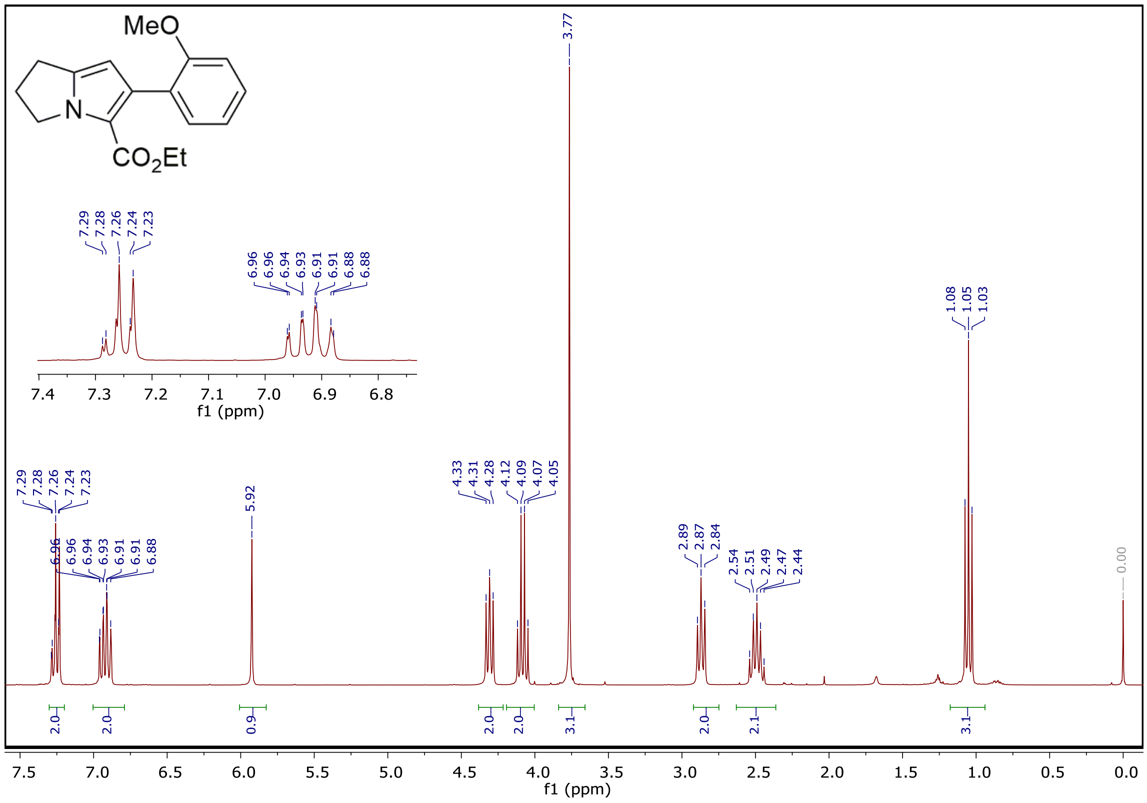

^{13}C NMR spectrum of ethyl 6-(2-methoxyphenyl)-2,3-dihydro-1*H*-pyrrolizine-5-carboxylate (19p) (75 MHz, $CDCl_3$)

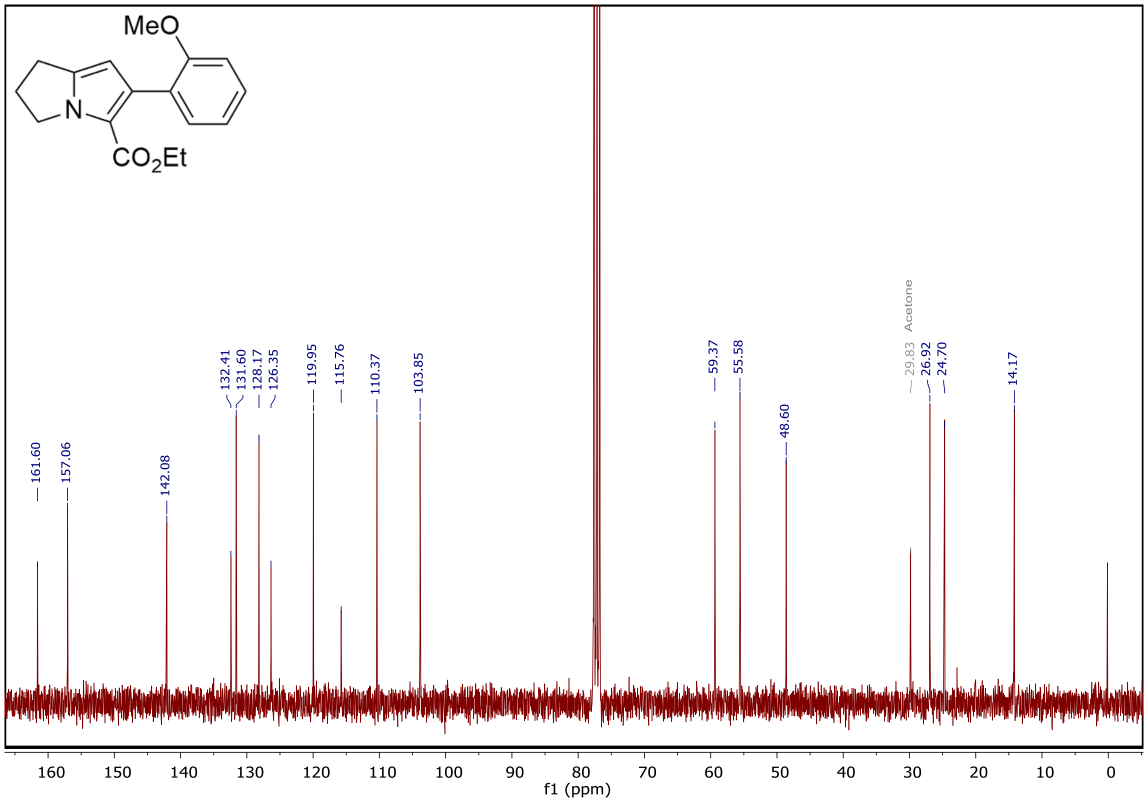

^{1}H NMR spectrum of ethyl 6-(2,5-dimethoxyphenyl)-2,3-dihydro-1*H*-pyrrolizine-5-carboxylate (19q) (300 MHz, $CDCl_3$)

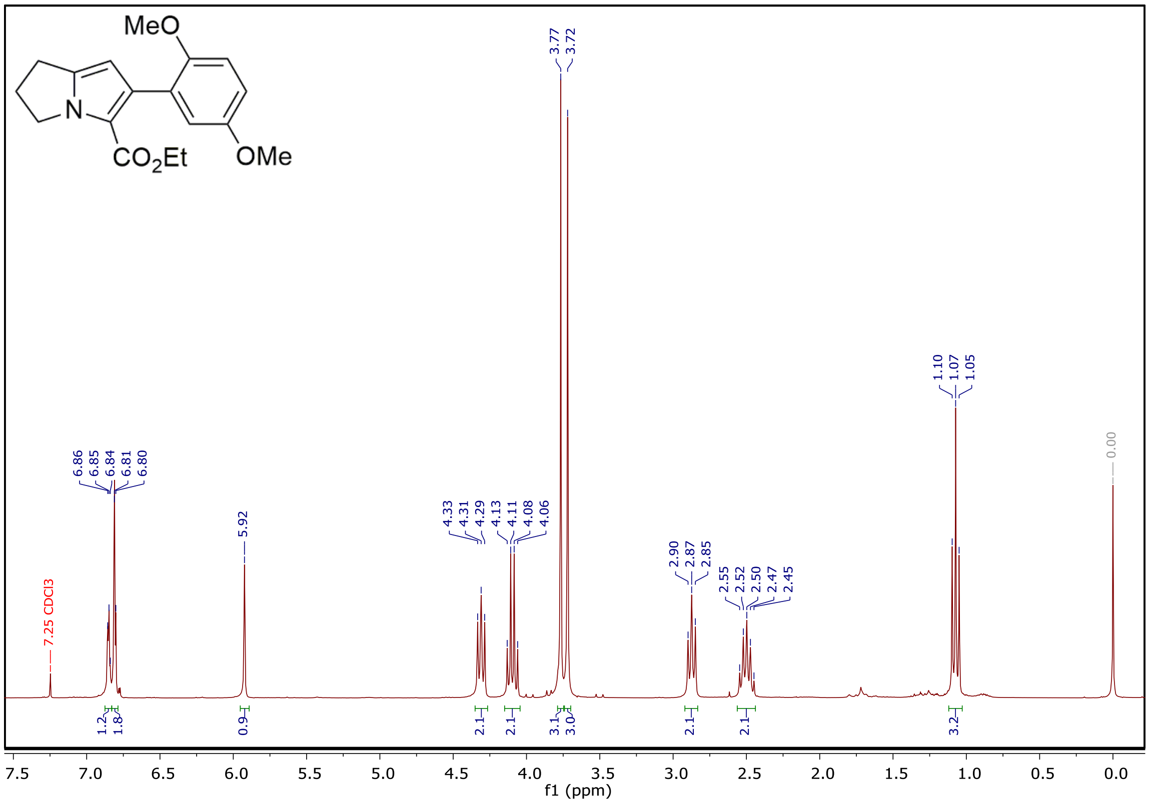

^{13}C NMR spectrum of ethyl 6-(2,5-dimethoxyphenyl)-2,3-dihydro-1*H*-pyrrolizine-5-carboxylate (19q) (75 MHz, $CDCl_3$)

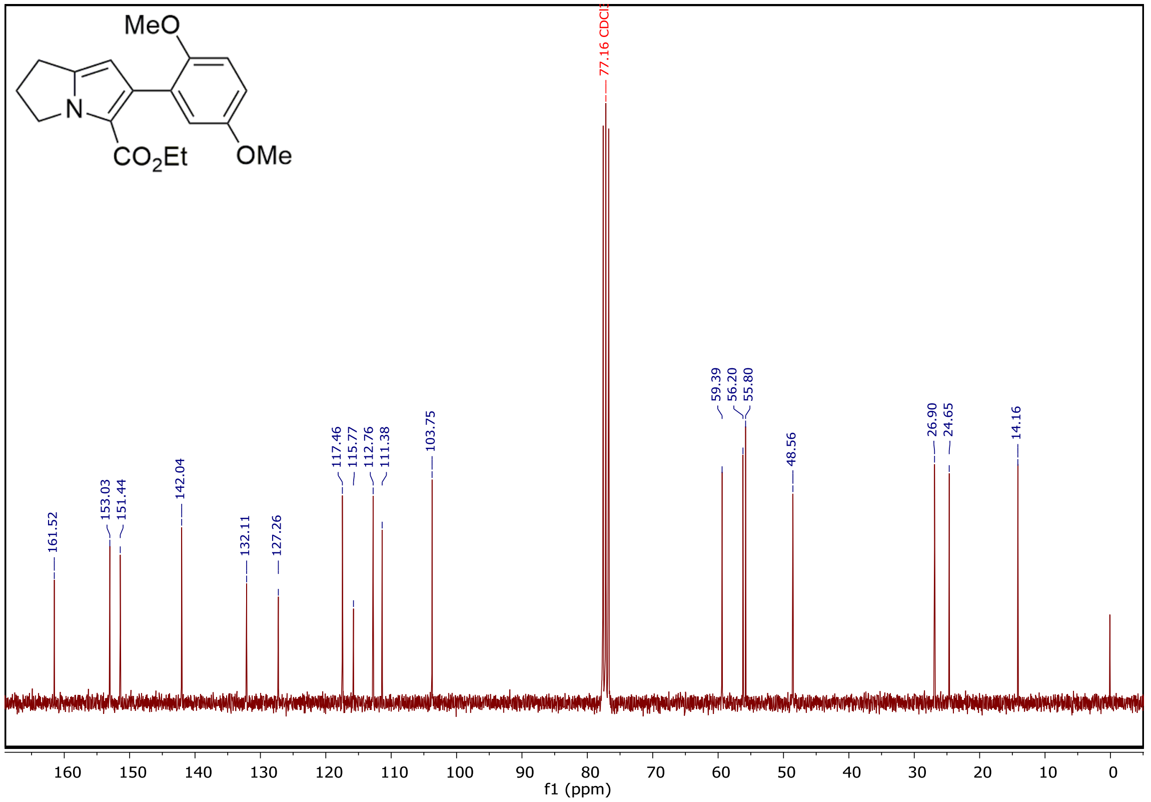

^{1}H NMR spectrum of ethyl 6-(2-fluorophenyl)-2,3-dihydro-1*H*-pyrrolizine-5-carboxylate (19r) (300 MHz, $CDCl_3$)

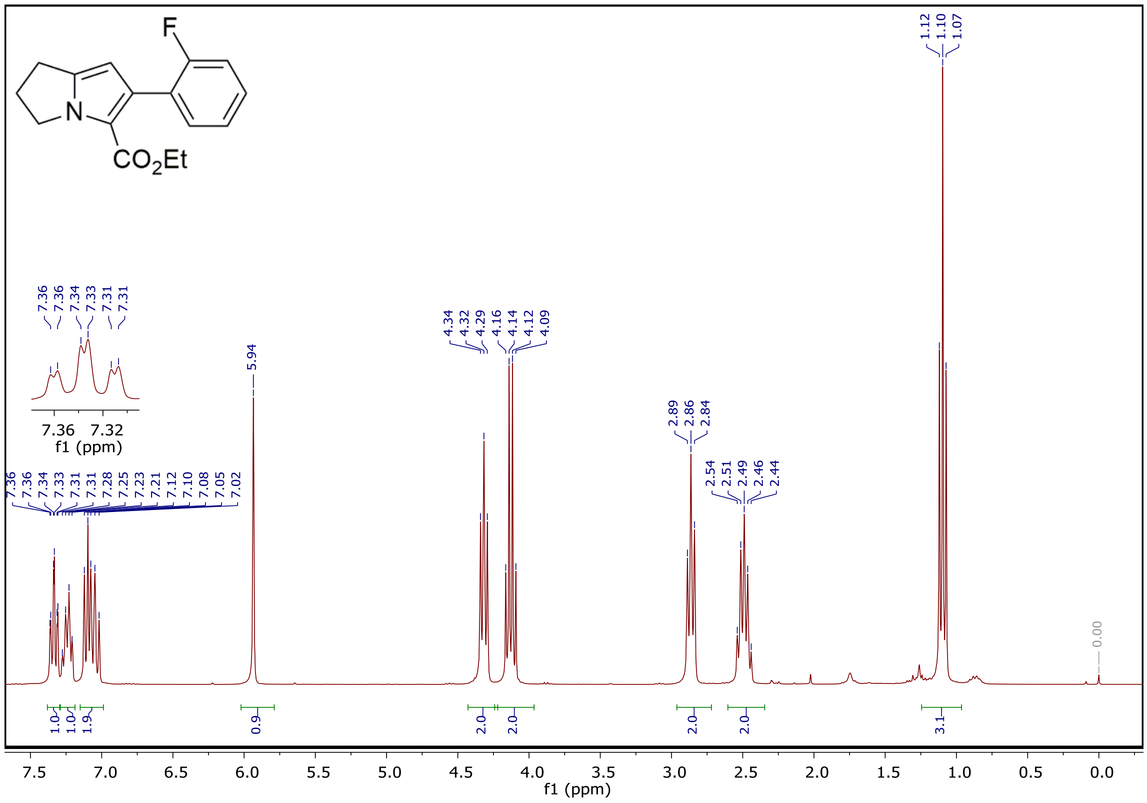

^{13}C NMR spectrum of ethyl 6-(2-fluorophenyl)-2,3-dihydro-1*H*-pyrrolizine-5-carboxylate (19r) (75 MHz, $CDCl_3$)

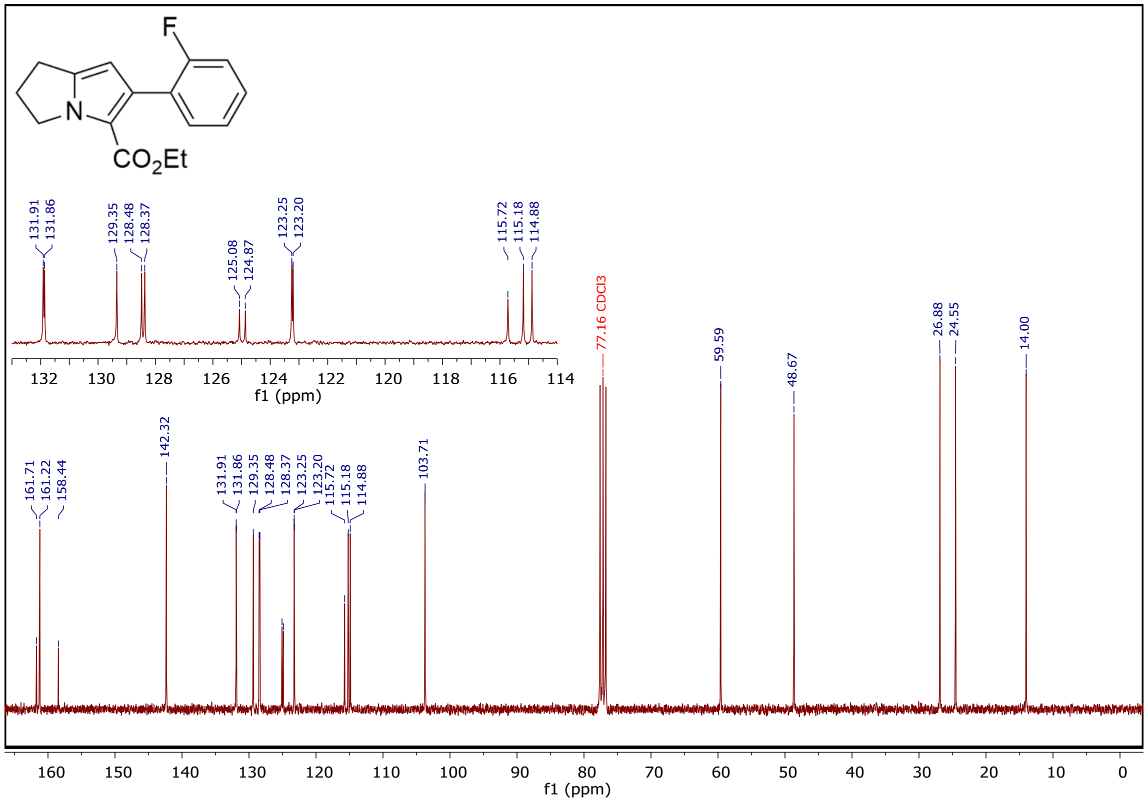

^{1}H NMR spectrum of ethyl 6-(naphthalen-1-yl)-2,3-dihydro-1*H*-pyrrolizine-5-carboxylate (19s) (300 MHz, $CDCl_3$)

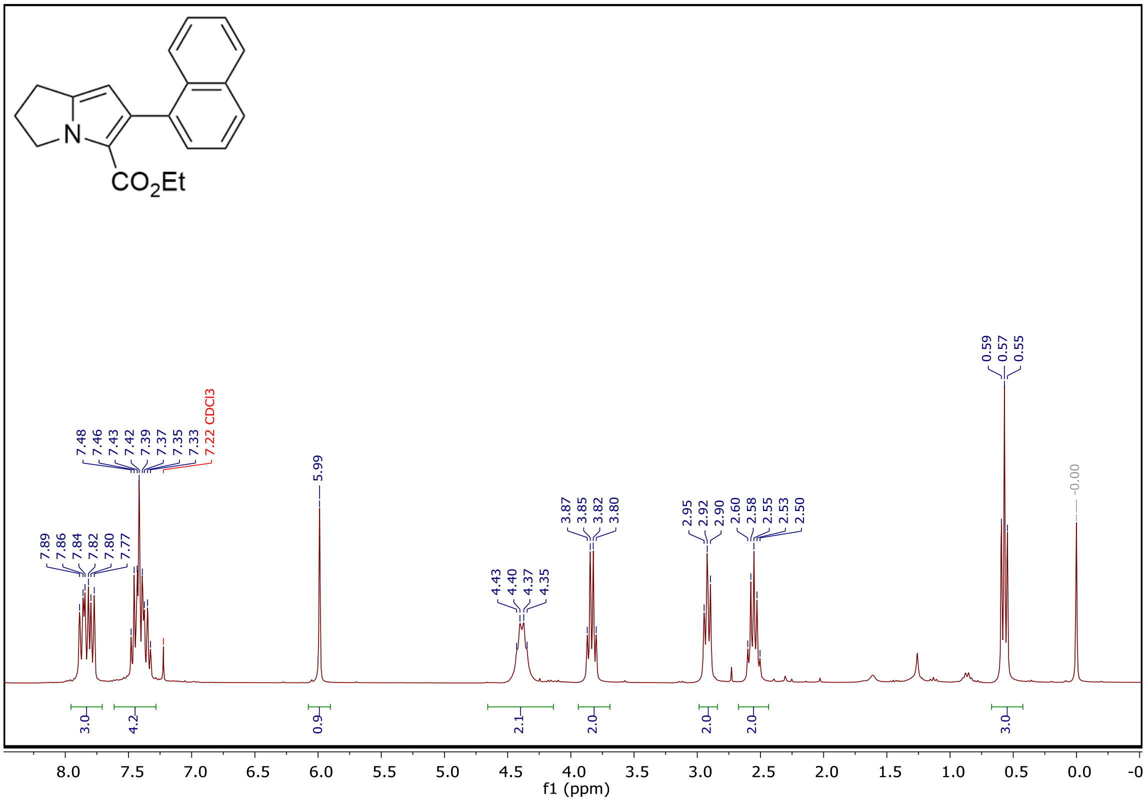

^{13}C NMR spectrum of ethyl 6-(naphthalen-1-yl)-2,3-dihydro-1*H*-pyrrolizine-5-carboxylate (19s) (75 MHz, $CDCl_3$)

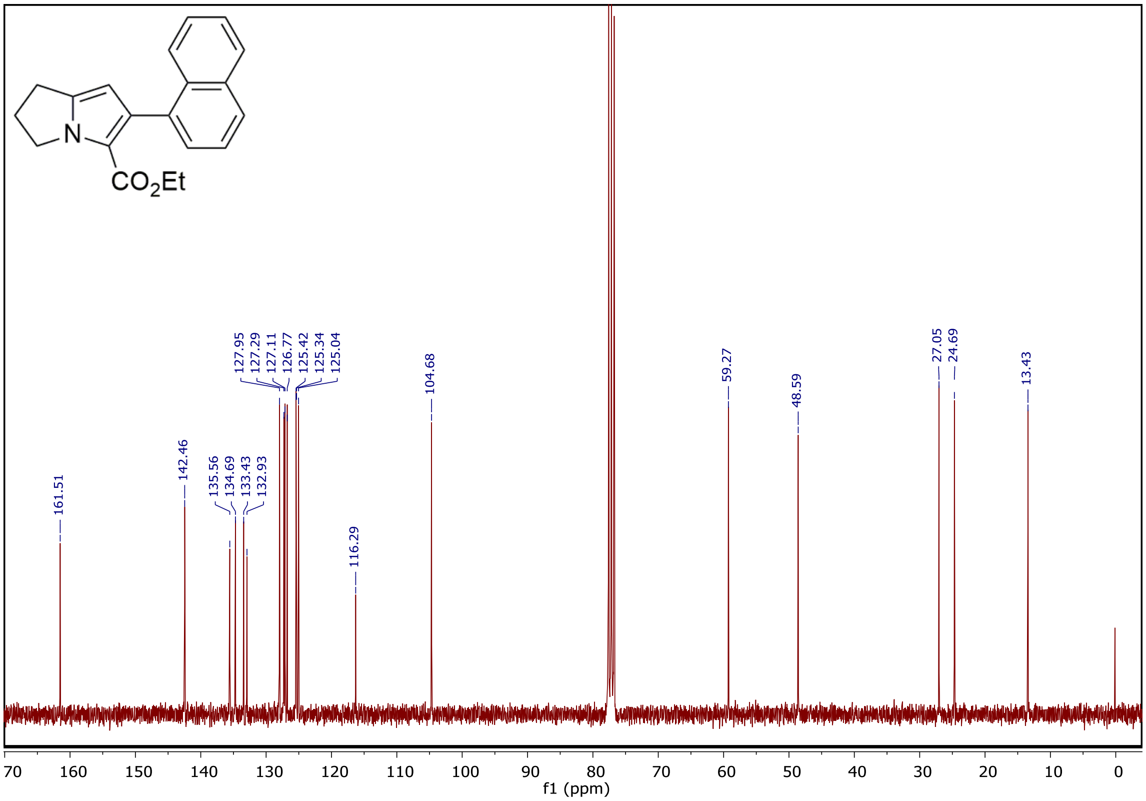

^{1}H NMR spectrum of (*E*)-ethyl 6-styryl-2,3-dihydro-1*H*-pyrrolizine-5-carboxylate (19t) (300 MHz, CDCl3)

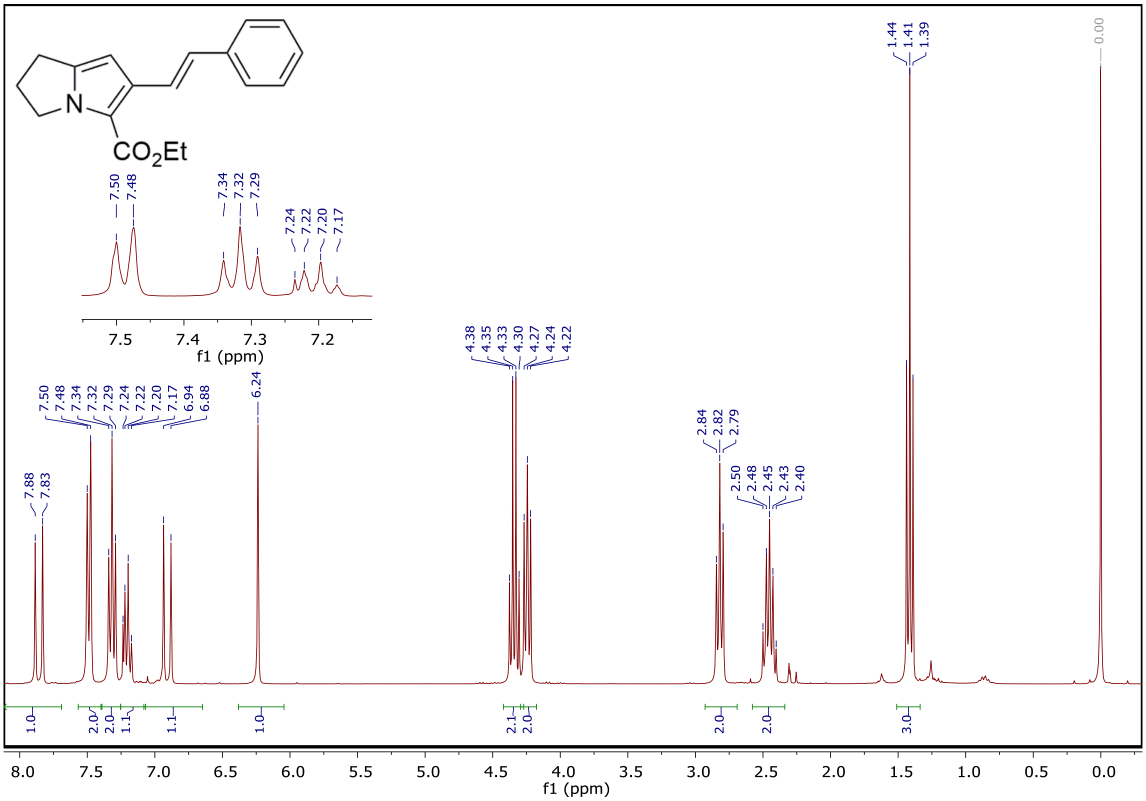

^{13}C NMR spectrum of (*E*)-ethyl 6-styryl-2,3-dihydro-1*H*-pyrrolizine-5-carboxylate (19t) (75 MHz, CDCl3)

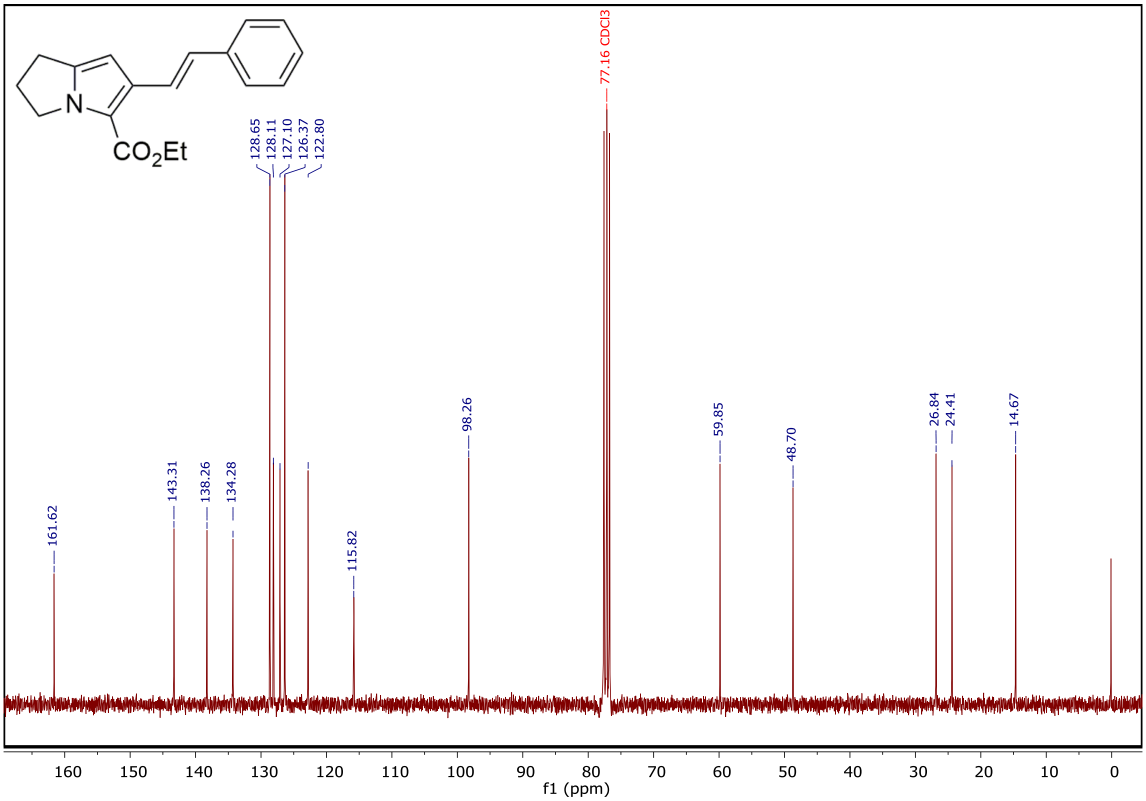

^{1}H NMR spectrum of ethyl 6-(furan-2-yl)-2,3-dihydro-1*H*-pyrrolizine-5-carboxylate (19u) (300 MHz, $CDCl_3$)

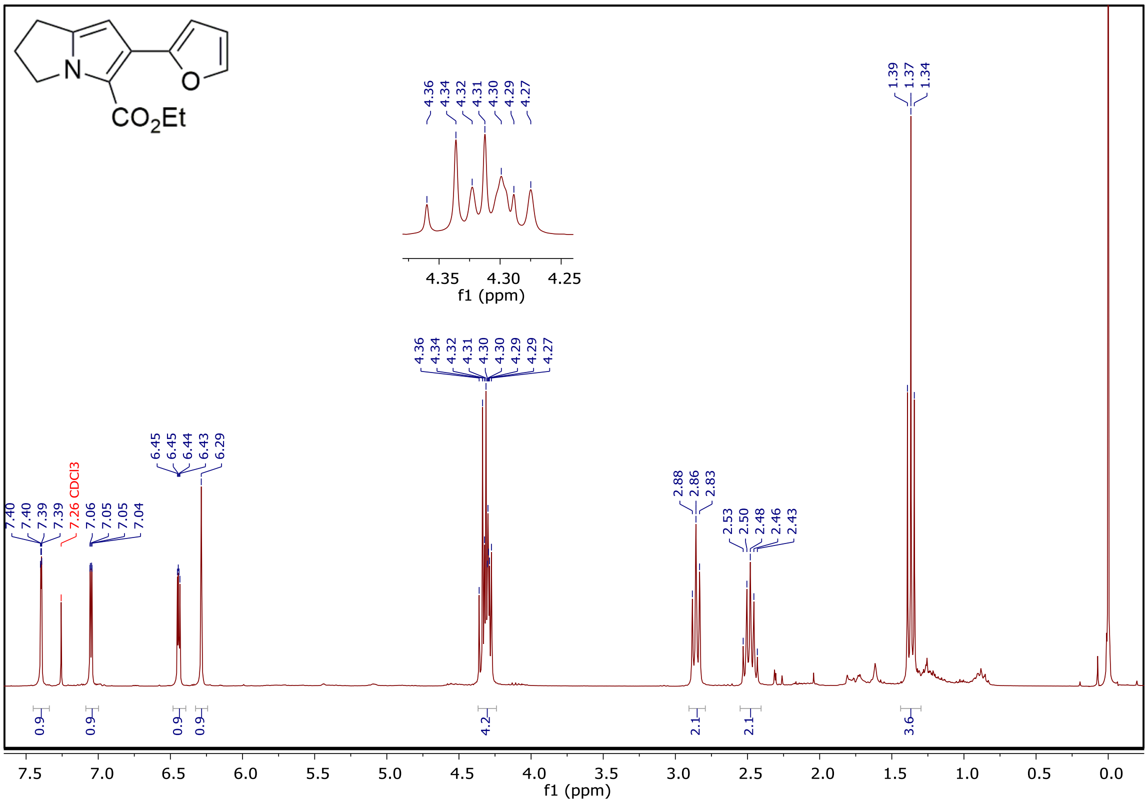

^{13}C NMR spectrum of ethyl 6-(furan-2-yl)-2,3-dihydro-1*H*-pyrrolizine-5-carboxylate (19u) (101 MHz, $CDCl_3$)

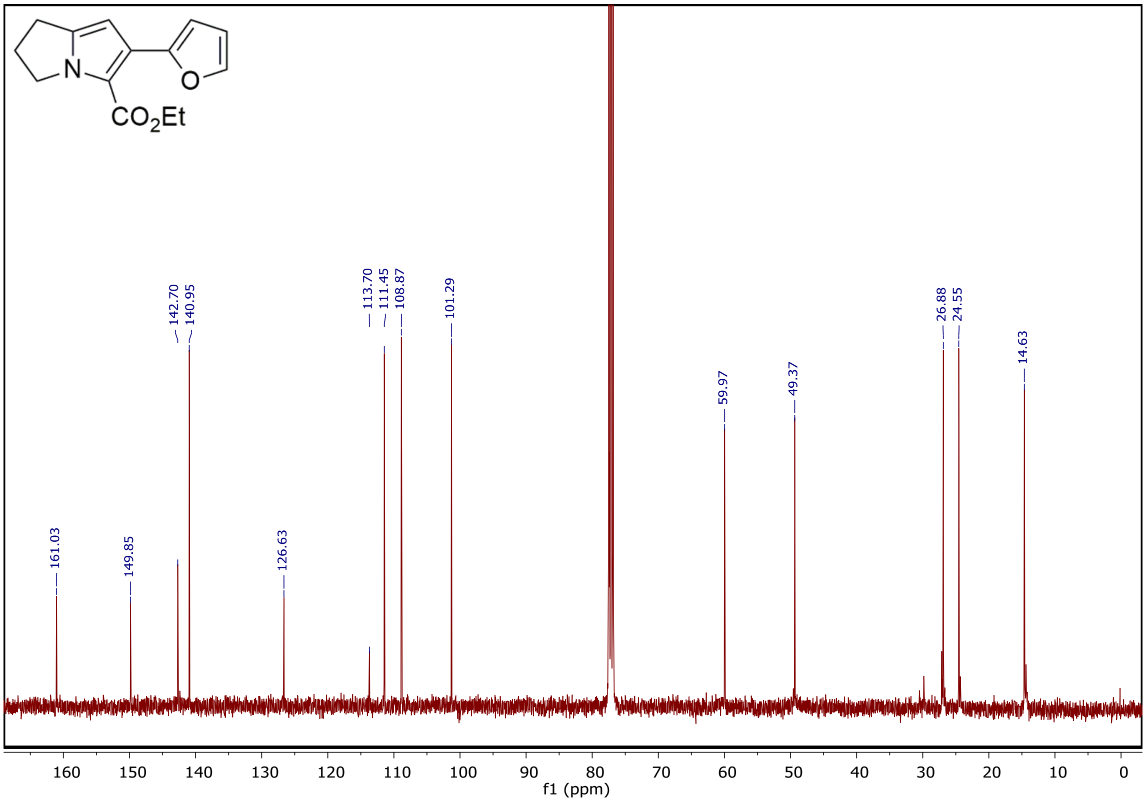

^{1}H NMR spectrum of ethyl 6-(thiophen-2-yl)-2,3-dihydro-*1H*-pyrrolizine-5-carboxylate (19v) (300 MHz, $CDCl_3$)

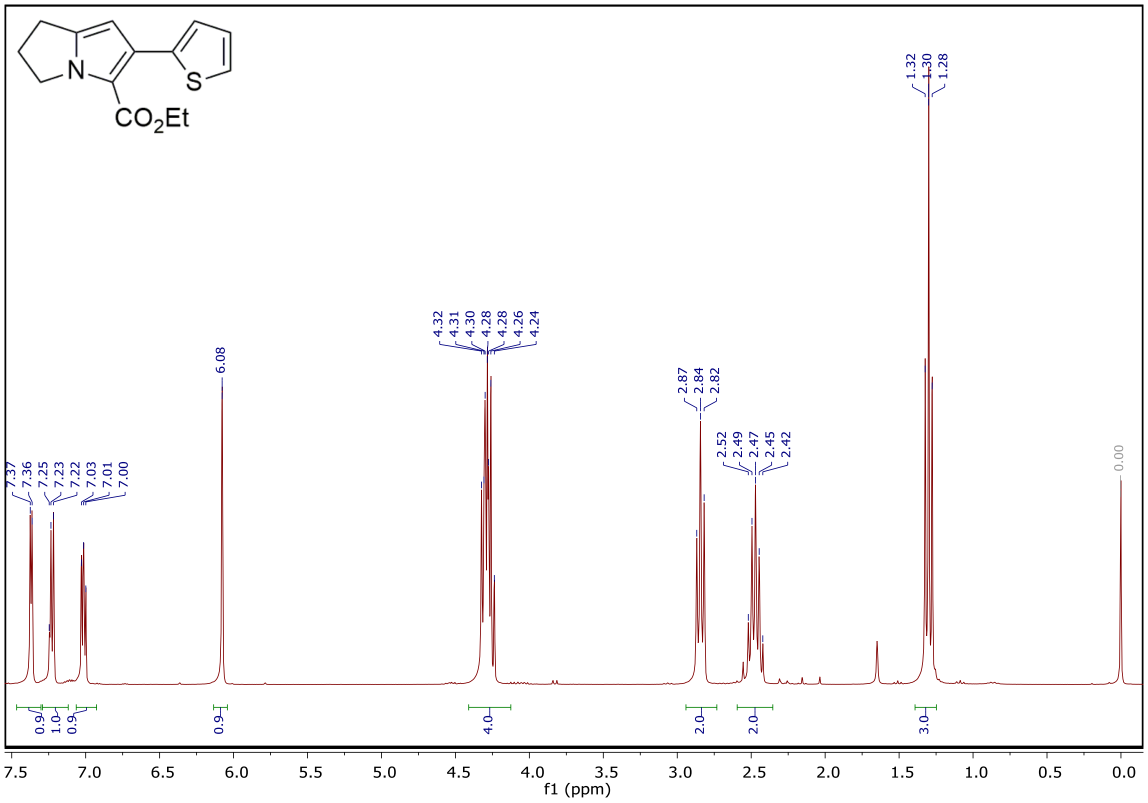

^{13}C NMR spectrum of ethyl 6-(thiophen-2-yl)-2,3-dihydro-1*H*-pyrrolizine-5-carboxylate (19v) (75 MHz, $CDCl_3$)

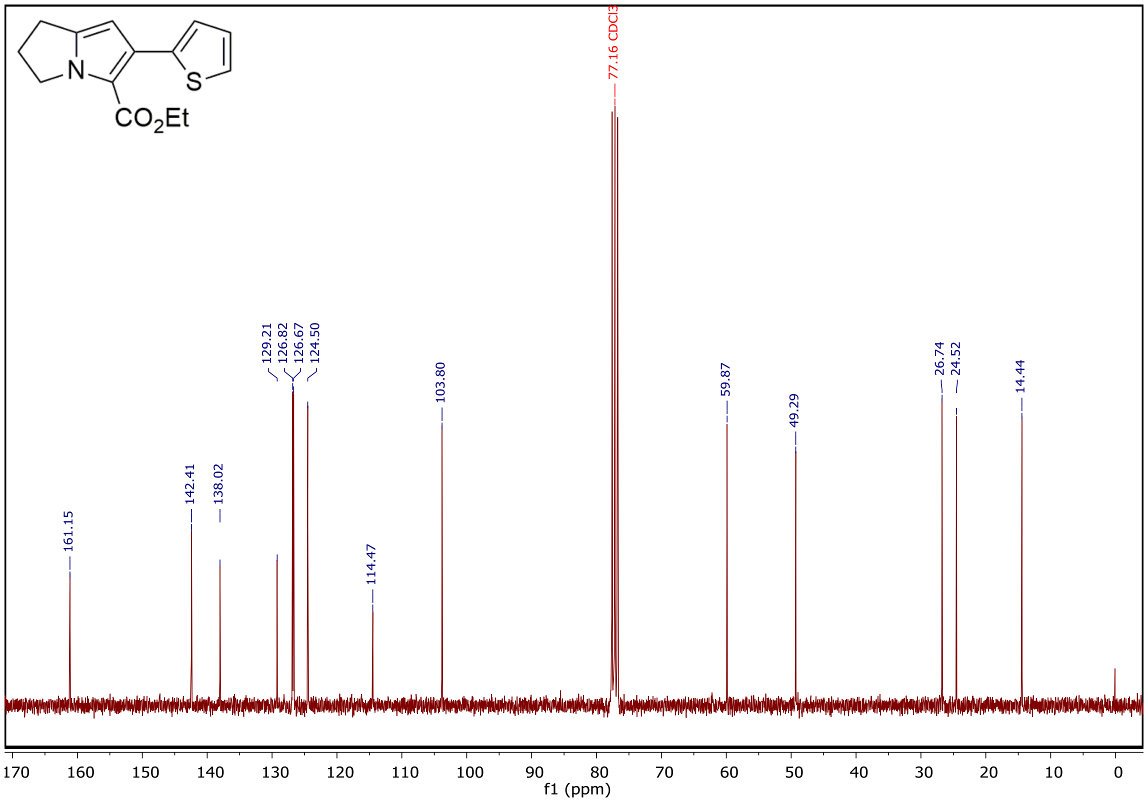

^{1}H NMR spectrum of ethyl 6-(benzofuran-2-yl)-2,3-dihydro-1*H*-pyrrolizine-5-carboxylate (19w) (300 MHz, $CDCl_3$)

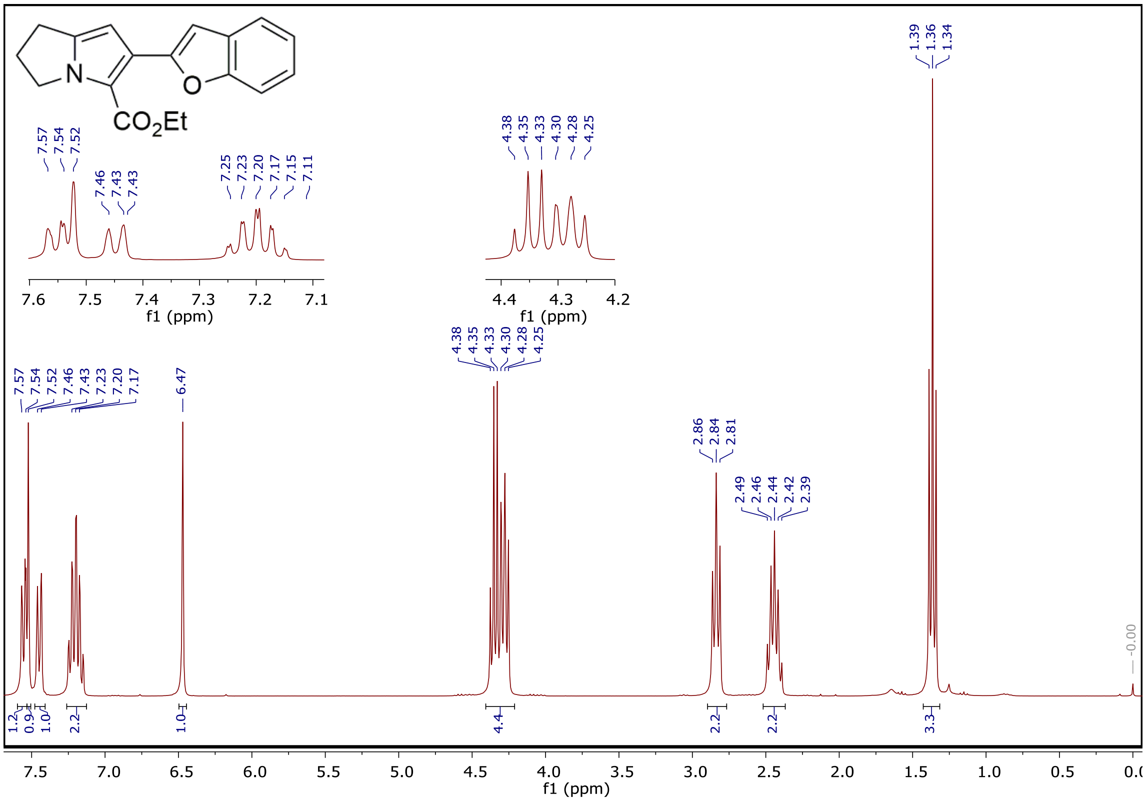

^{13}C NMR spectrum of ethyl 6-(benzofuran-2-yl)-2,3-dihydro-1*H*-pyrrolizine-5-carboxylate (19w) (75 MHz, $CDCl_3$)

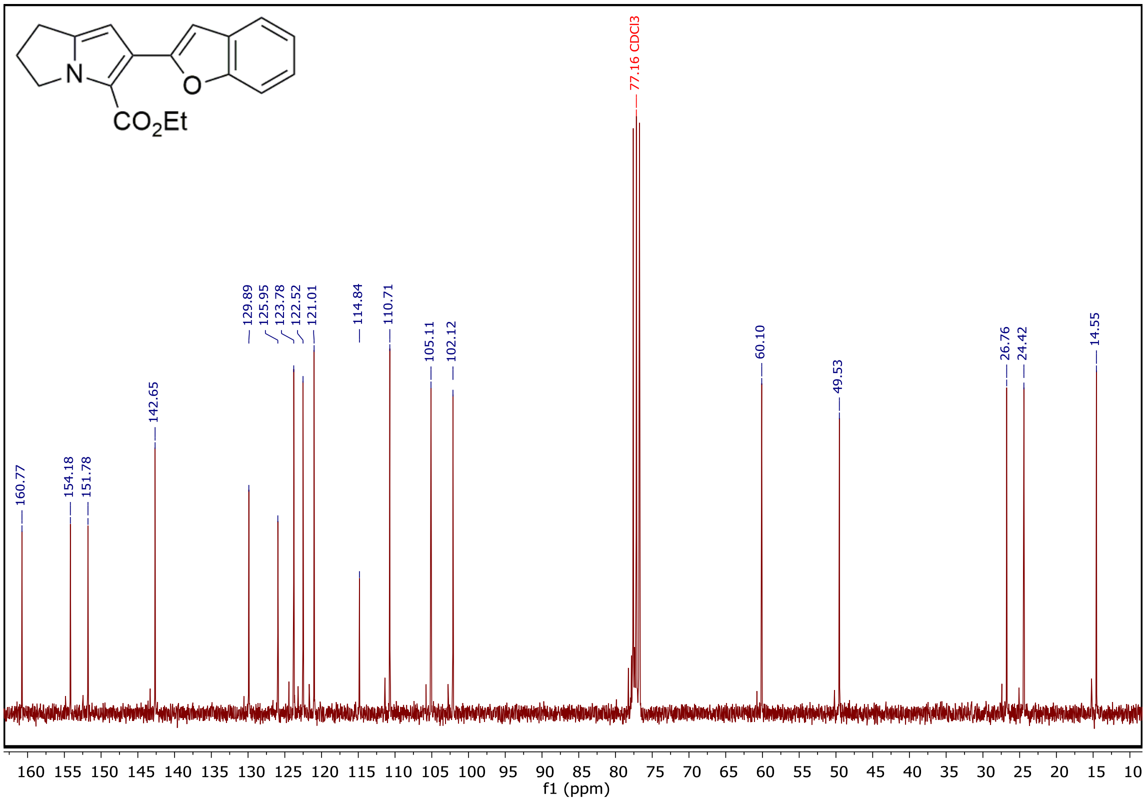

^{1}H NMR spectrum of ethyl 6-(1-tosyl-1*H*-indol-3-yl)-2,3-dihydro-1*H*-pyrrolizine-5-carboxylate (19x) (300 MHz, $CDCl_3$)

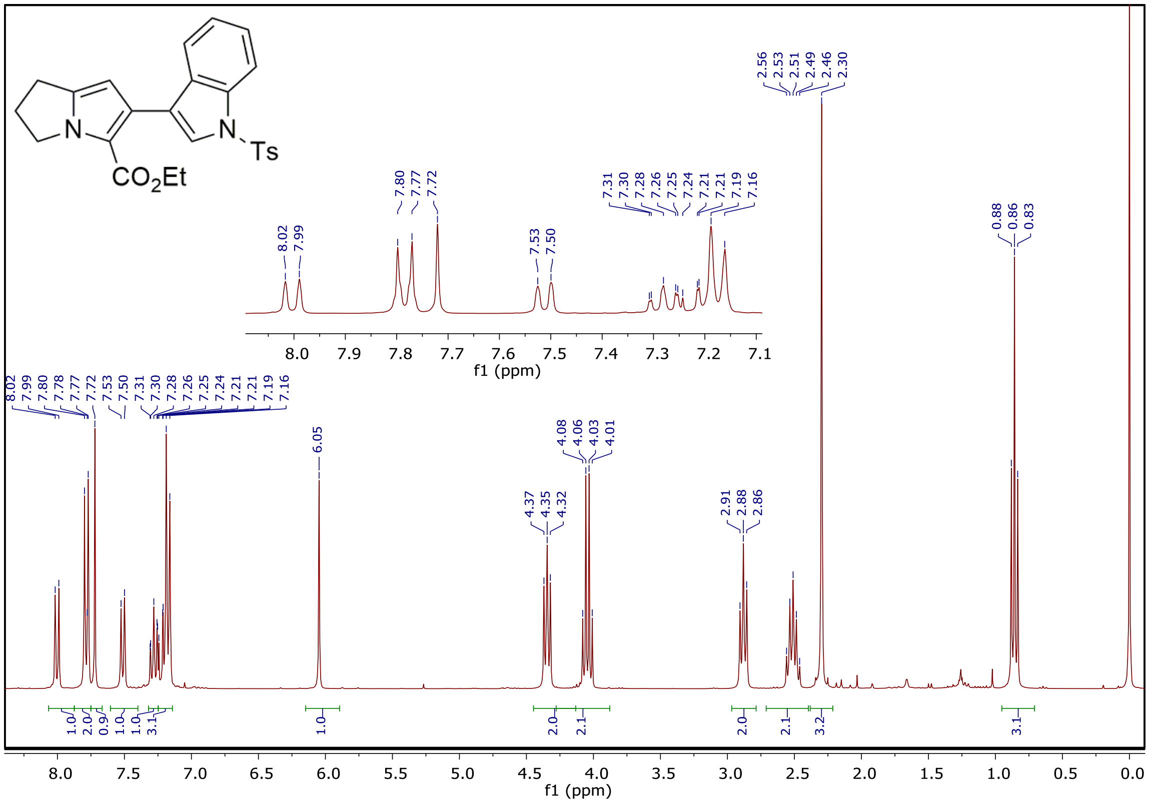

^{13}C NMR spectrum of ethyl 6-(1-tosyl-1*H*-indol-3-yl)-2,3-dihydro-*1H*-pyrrolizine-5-carboxylate (19x) (75 MHz, $CDCl_3$)

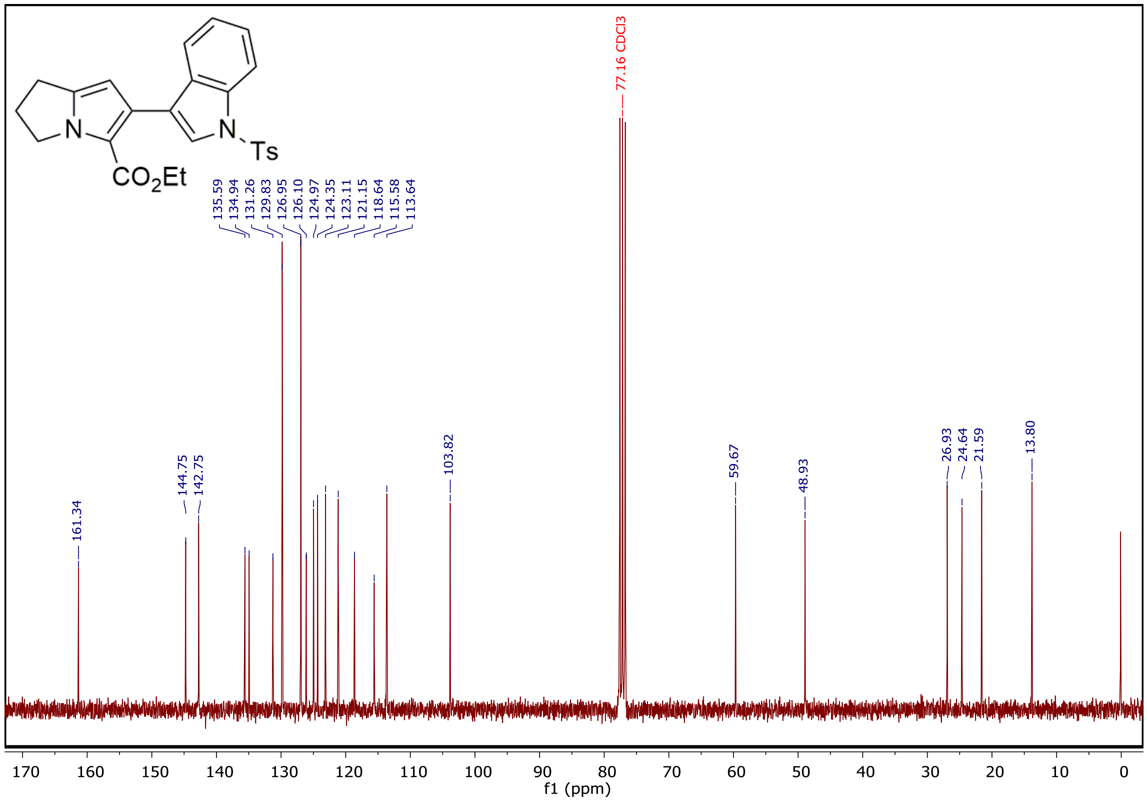

^{1}H NMR spectrum of ethyl 6-(*tert*-butyl)-2,3-dihydro-1*H*-pyrrolizine-5-carboxylate (19y) (300 MHz, $CDCl_3$)

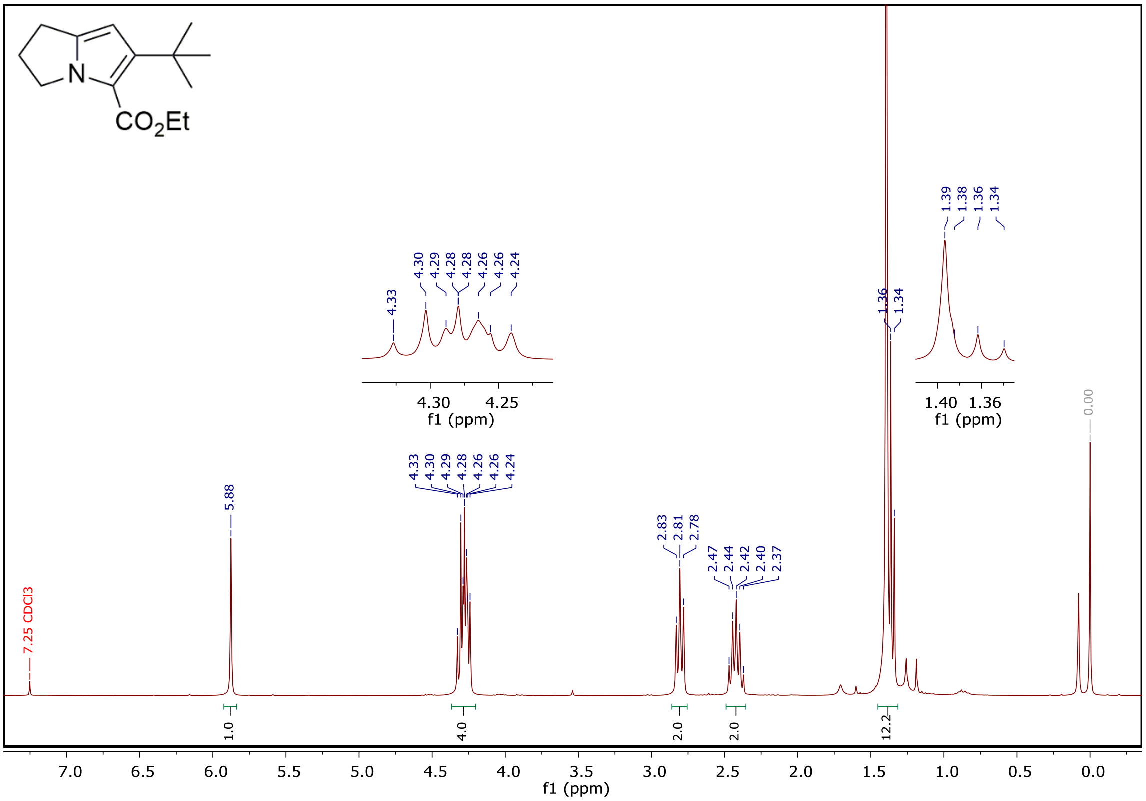

^{13}C NMR spectrum of ethyl 6-(*tert*-butyl)-2,3-dihydro-1*H*-pyrrolizine-5-carboxylate (19y) (75 MHz, $CDCl_3$)

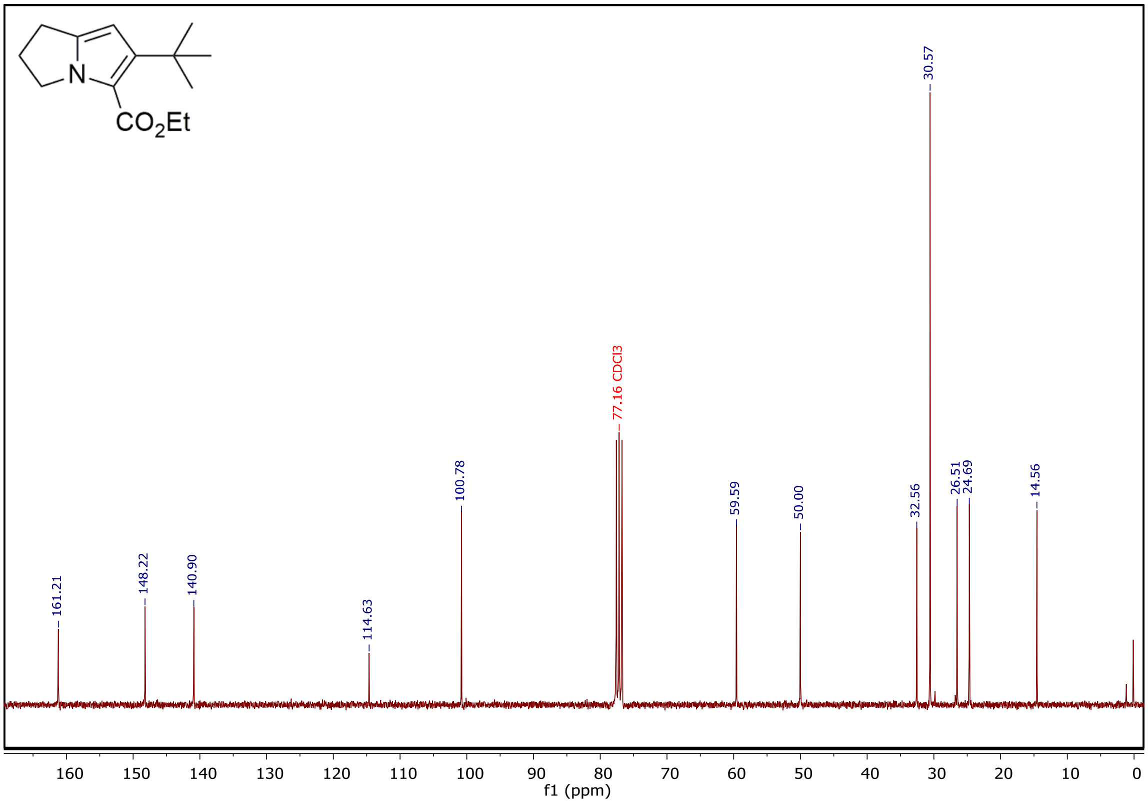

^{1}H NMR spectrum of (*E*)-ethyl 2-[2-(2-oxo-2-phenylethylidene)piperidin-1-yl]acetate (25a) (300 MHz, $CDCl_3$)

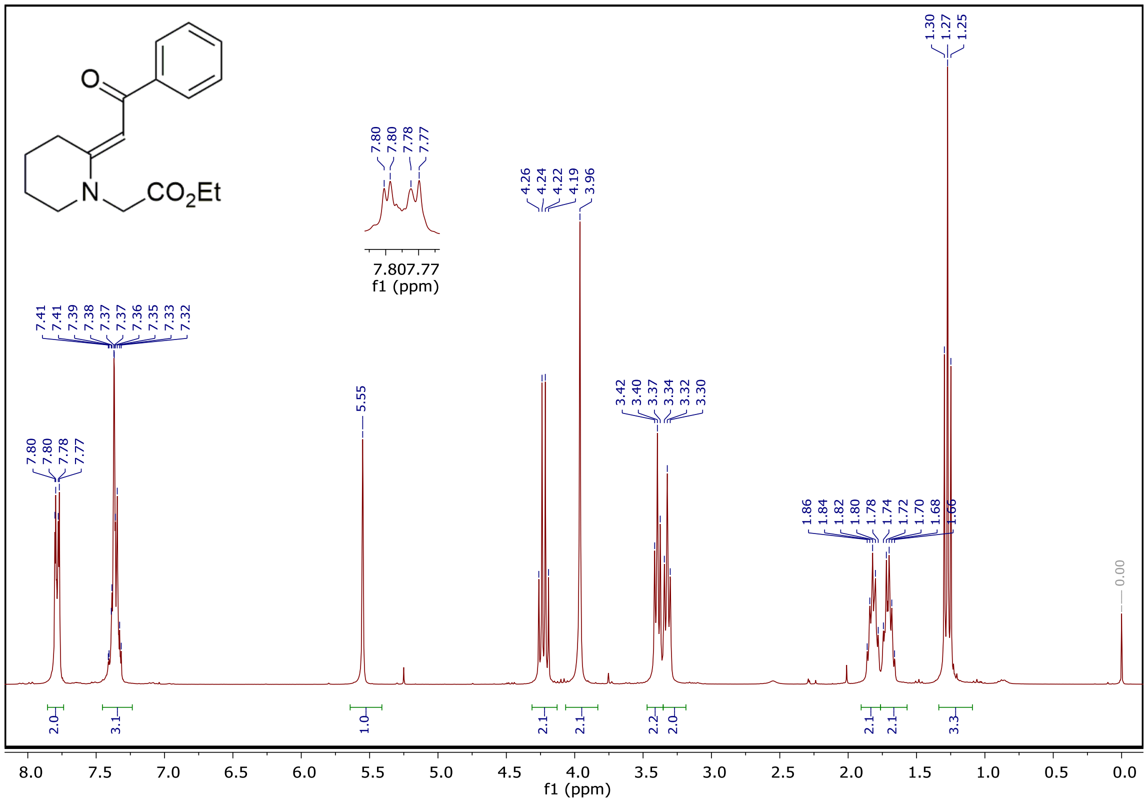

^{13}C NMR spectrum of (*E*)-ethyl 2-[2-(2-oxo-2-phenylethylidene)piperidin-1-yl]acetate (25a) (75 MHz, $CDCl_3$)

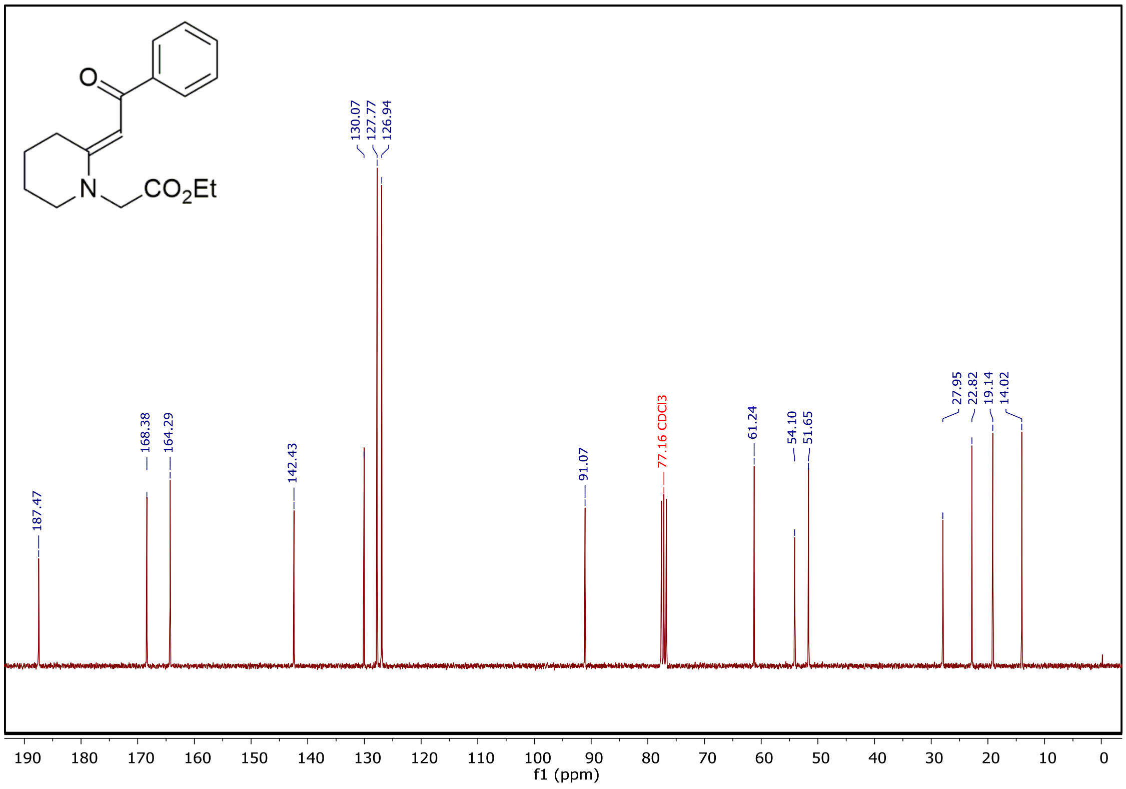

COSY NMR spectrum of (*E*)-ethyl 2-[2-(2-oxo-2-phenylethylidene)piperidin-1-yl]acetate (25a) (300 MHz, $CDCl_3$)

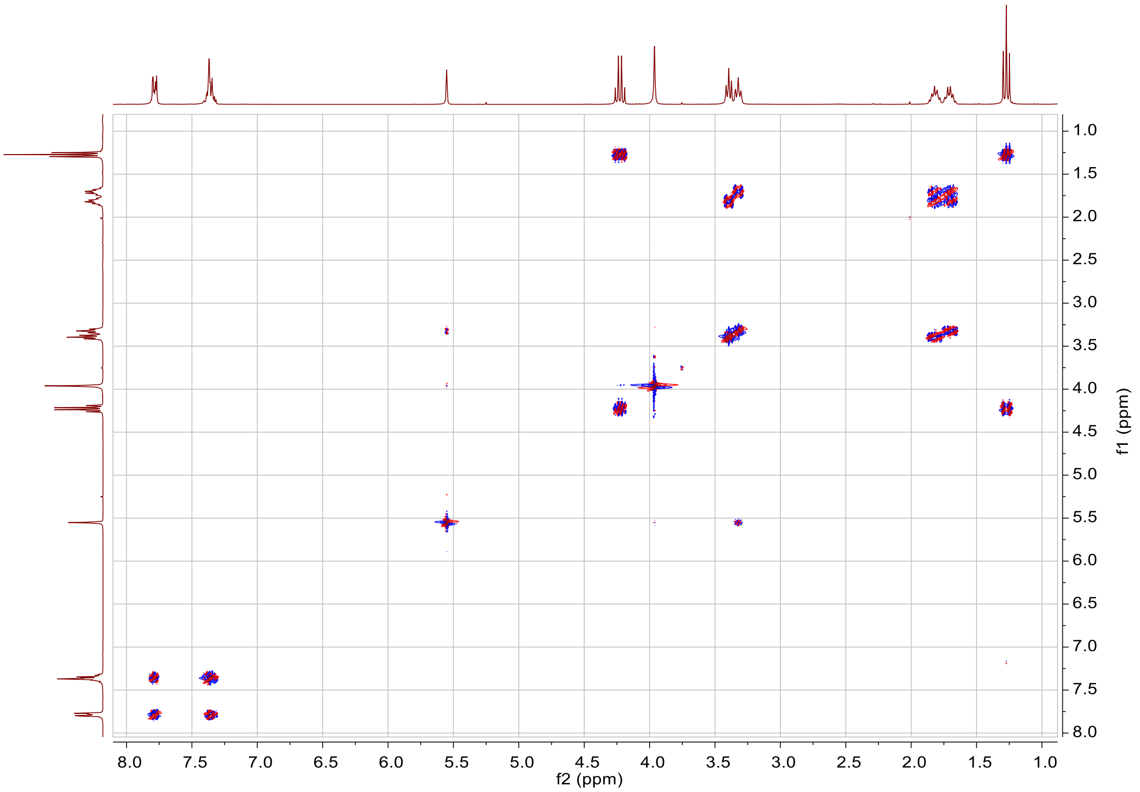

NOESY NMR spectrum of (*E*)-ethyl 2-[2-(2-oxo-2-phenylethylidene)piperidin-1-yl]acetate (25a) (300 MHz, $CDCl_3$)

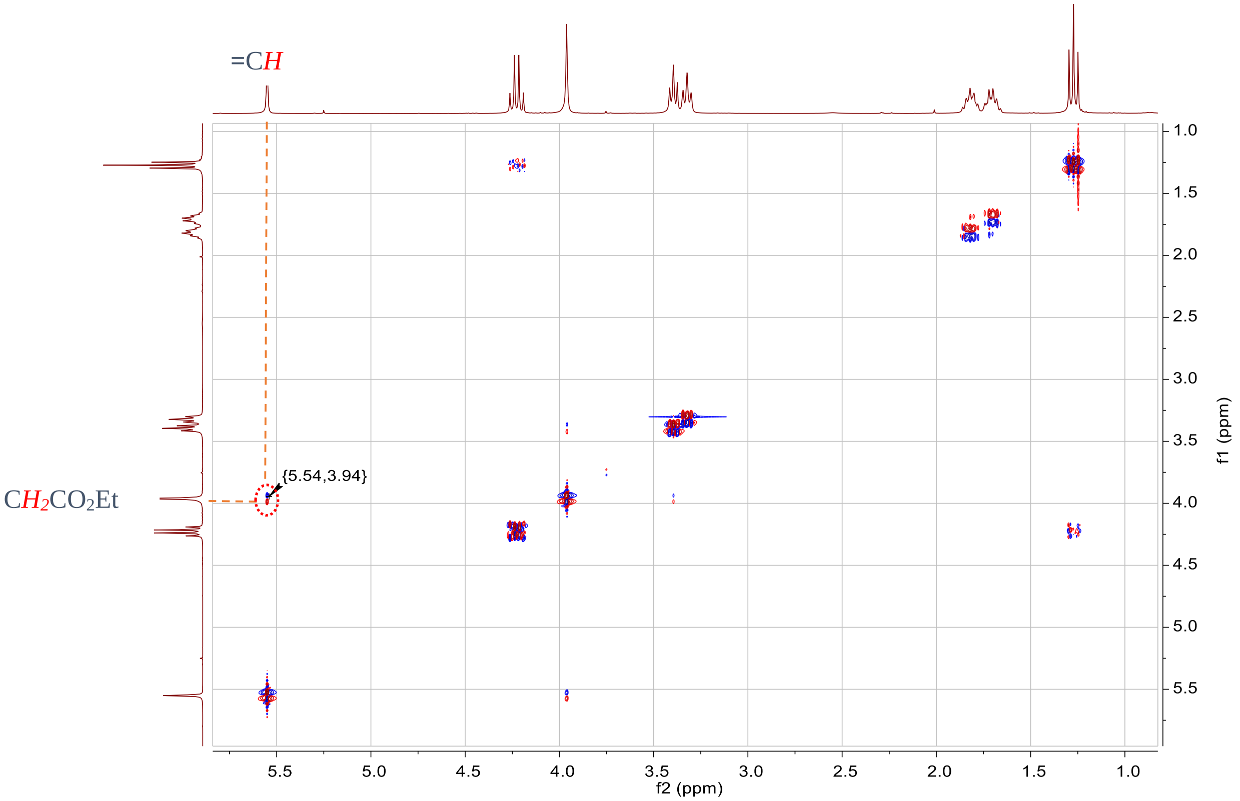

^{1}H NMR spectrum of (*E*)-ethyl 2-{2-[2-(4-methoxyphenyl)-2-oxoethylidene]piperidin-1-yl}acetate (25b) (300 MHz, $CDCl_3$)

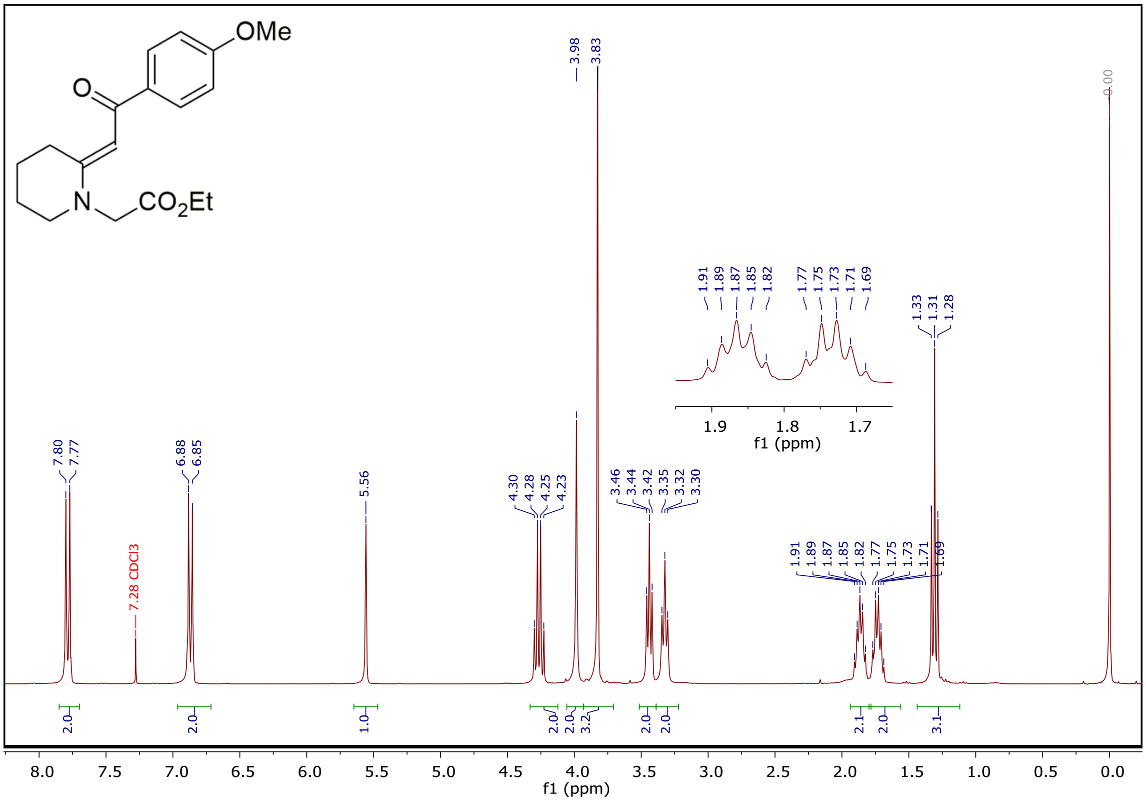

^{13}C NMR spectrum of (*E*)-ethyl 2-{2-[2-(4-methoxyphenyl)-2-oxoethylidene]piperidin-1-yl}acetate (25b) (75 MHz, $CDCl_3$)

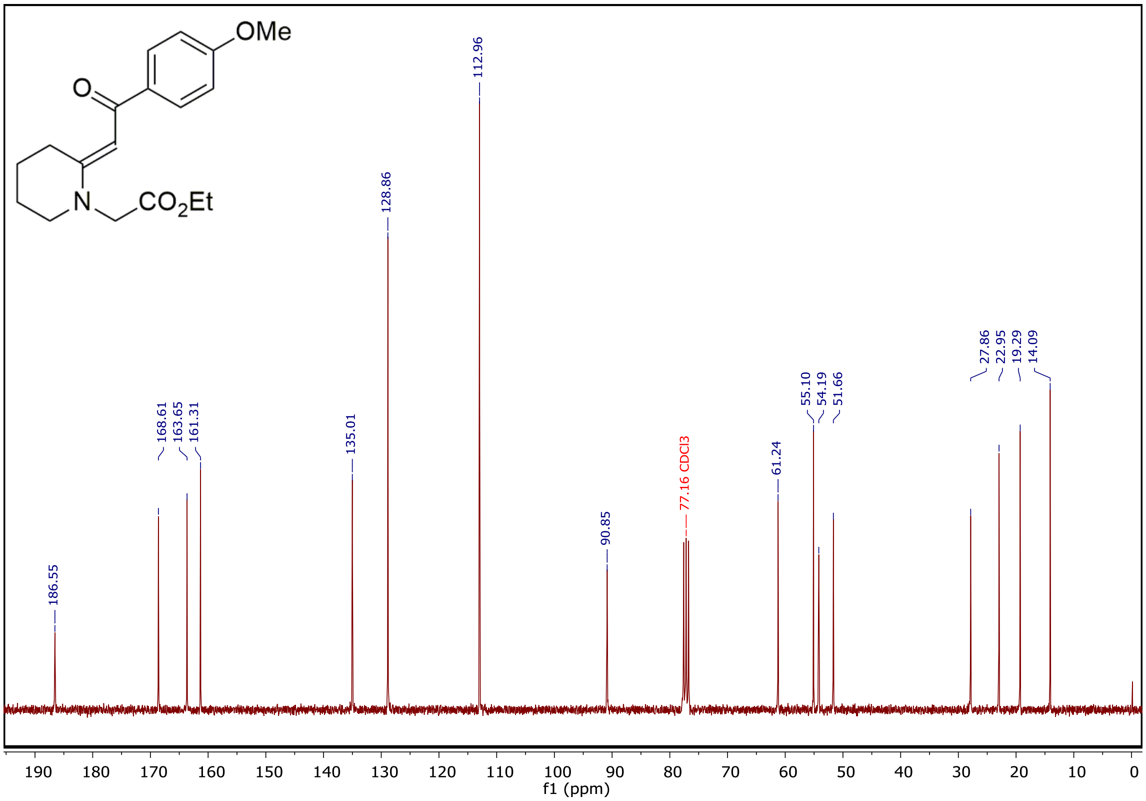

^{1}H NMR spectrum of (*E*)-ethyl 2-{2-[2-(4-nitrophenyl)-2-oxoethylidene]piperidin-1-yl}acetate (25c) (300 MHz, $CDCl_3$)

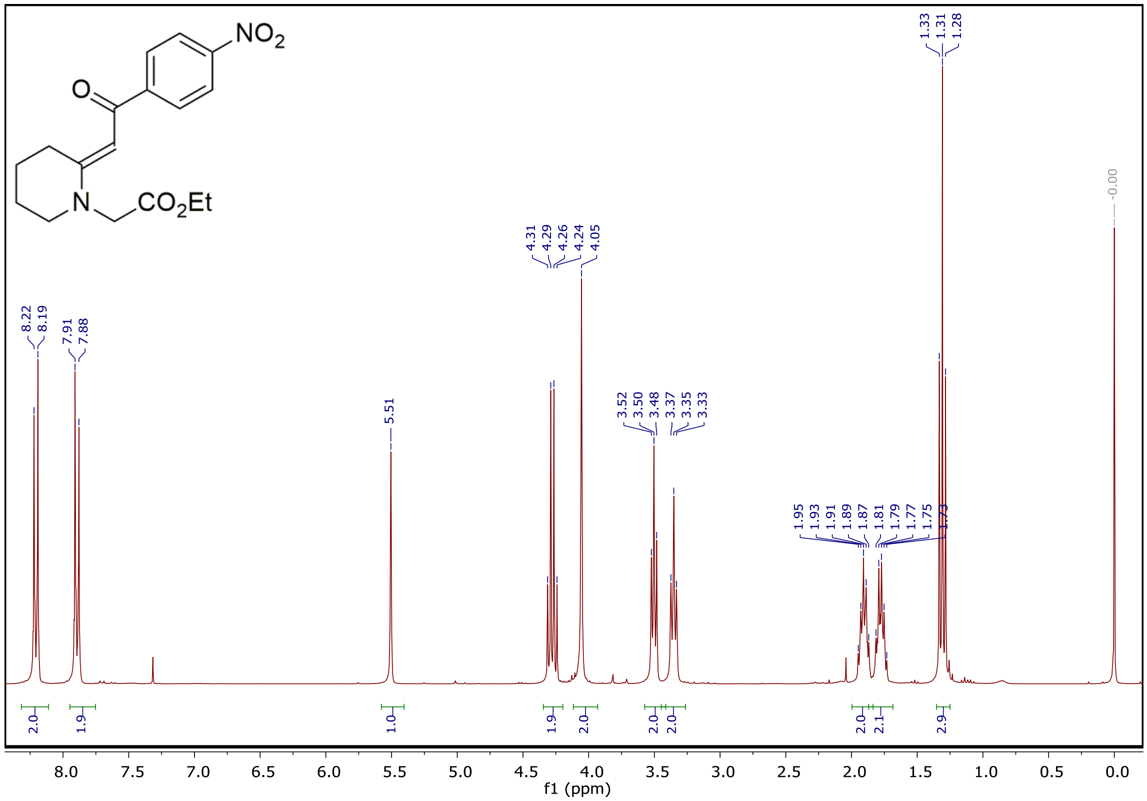

^{13}C NMR spectrum of (*E*)-ethyl 2-{2-[2-(4-nitrophenyl)-2-oxoethylidene]piperidin-1-yl}acetate (25c) (75 MHz, $CDCl_3$)

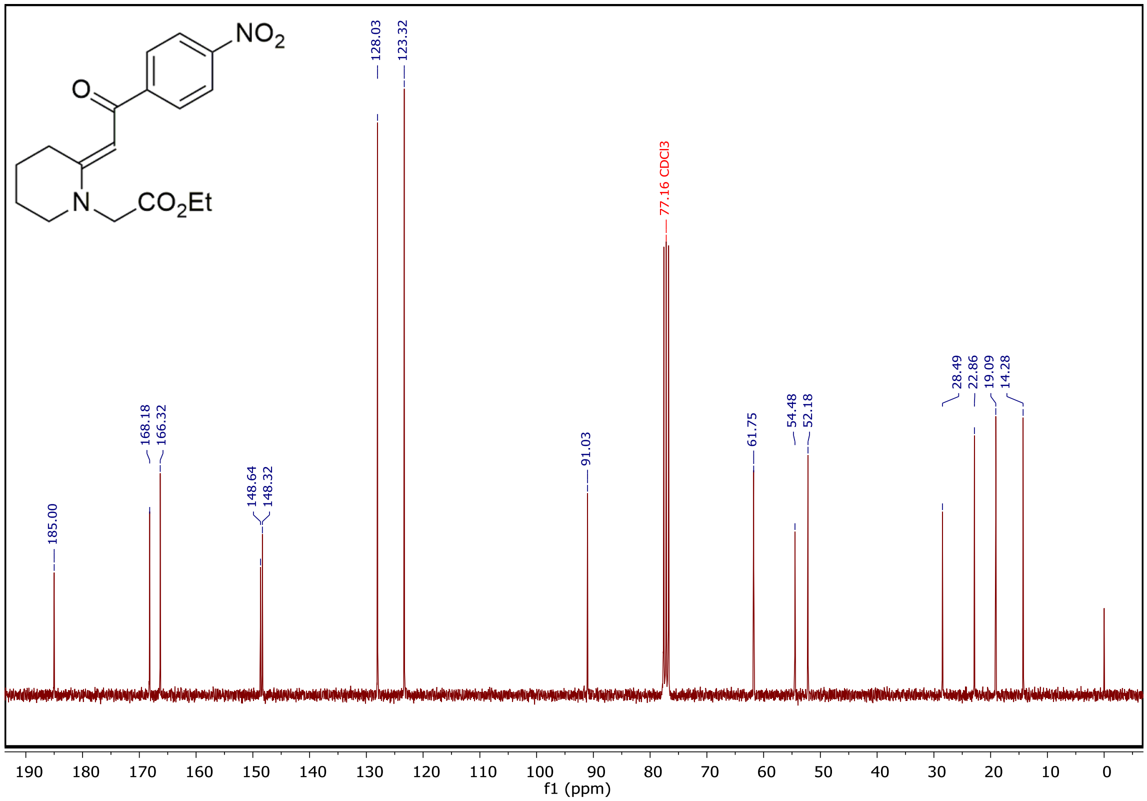

^{1}H NMR spectrum of ethyl 2-phenyl-5,6,7,8-tetrahydroindolizine-3-carboxylate (26a) (300 MHz, $CDCl_3$)

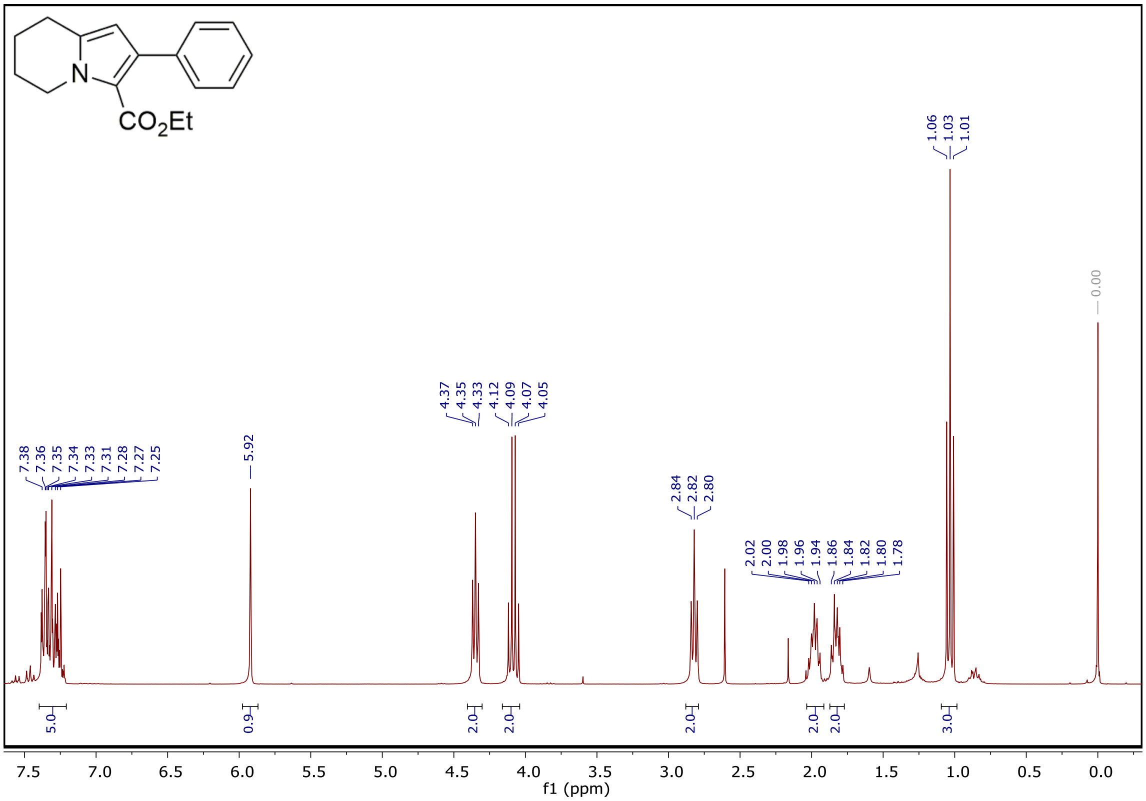

^{13}C NMR spectrum of ethyl 2-phenyl-5,6,7,8-tetrahydroindolizine-3-carboxylate (26a) (75 MHz, $CDCl_3$)

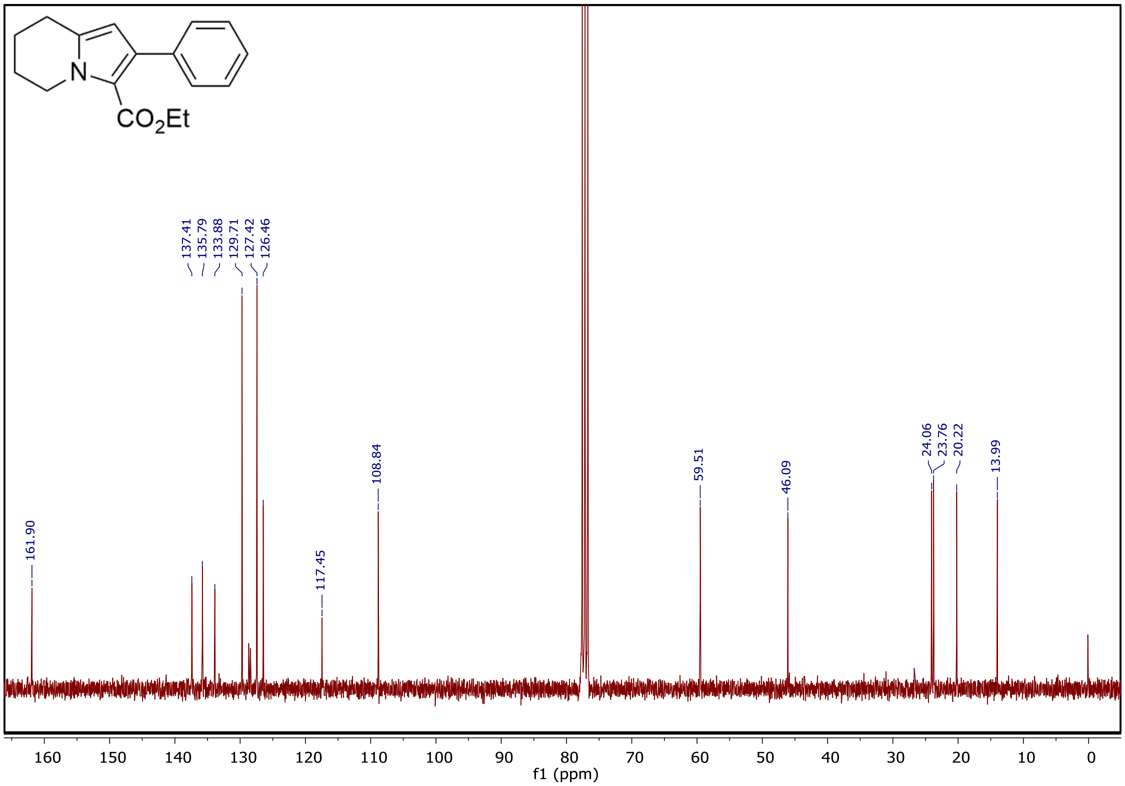

COSY NMR spectrum of ethyl 2-phenyl-5,6,7,8-tetrahydroindolizine-3-carboxylate (26a) (300 MHz, $CDCl_3$)

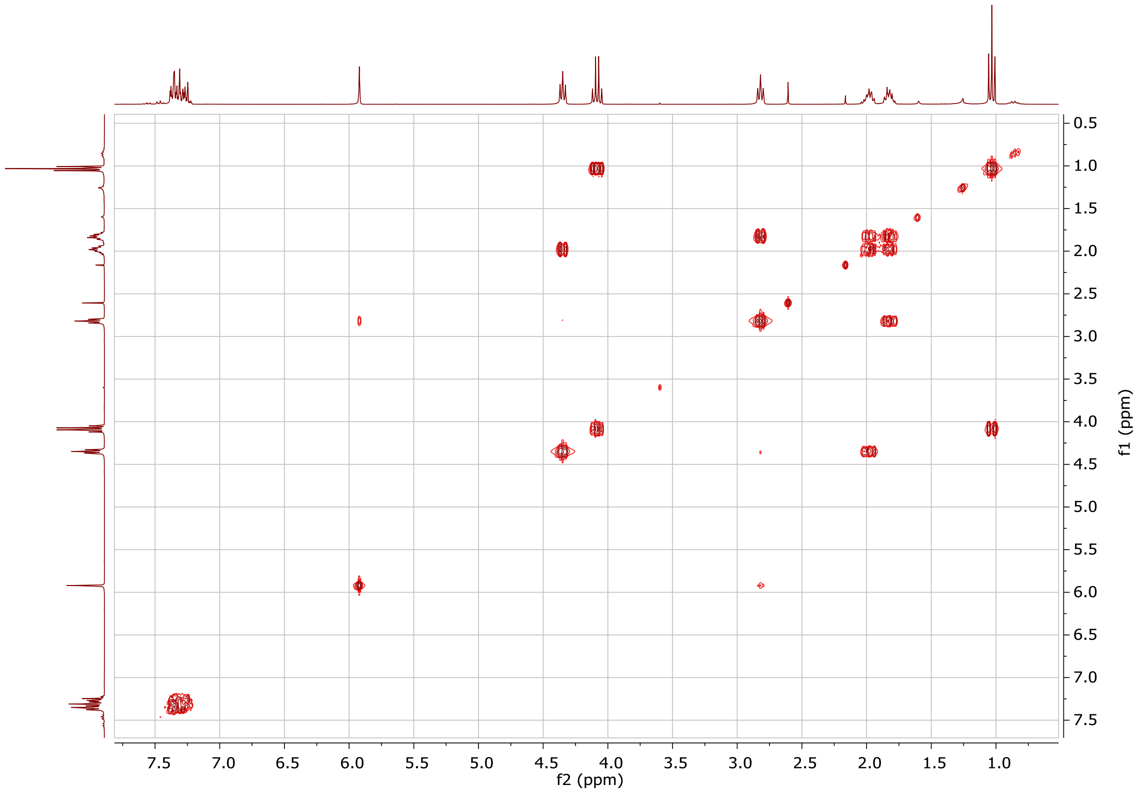

HSQC NMR spectrum of ethyl 2-phenyl-5,6,7,8-tetrahydroindolizine-3-carboxylate (26a) ($CDCl_3$)

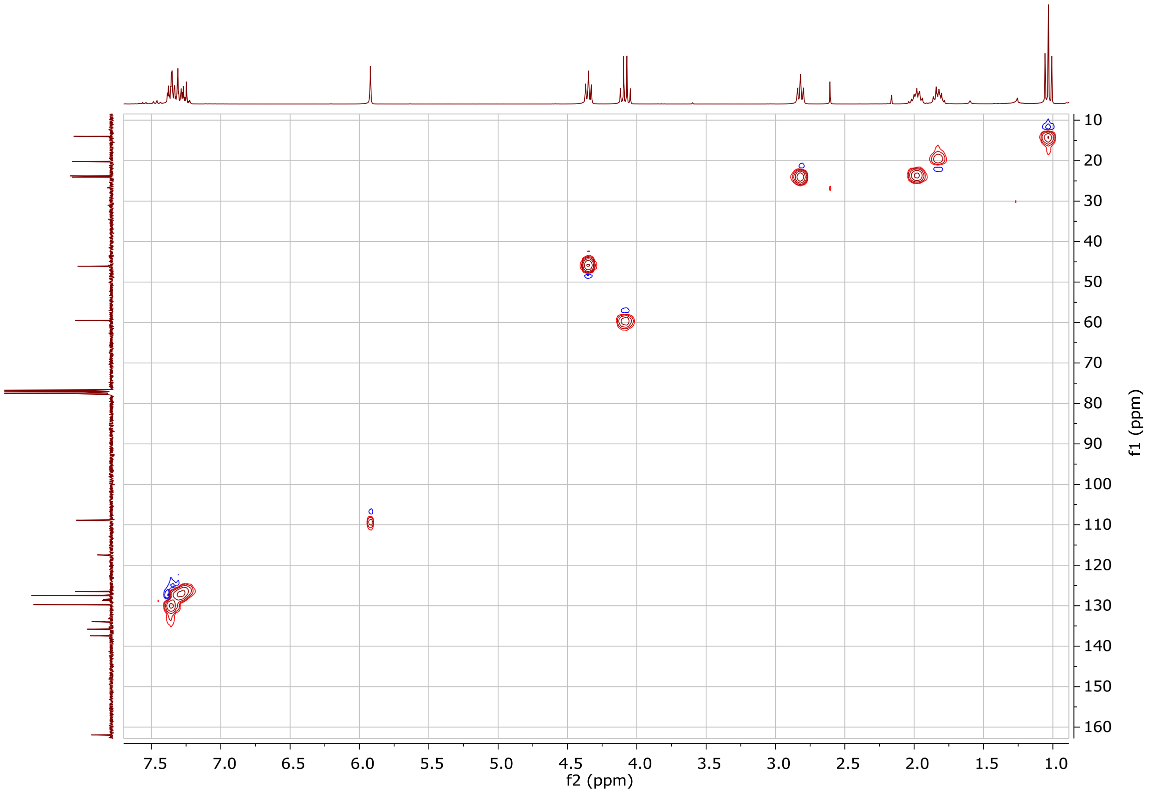

^{1}H NMR spectrum of ethyl 2-(4-methoxyphenyl)-5,6,7,8-tetrahydroindolizine-3-carboxylate (26b) (300 MHz, $CDCl_3$)

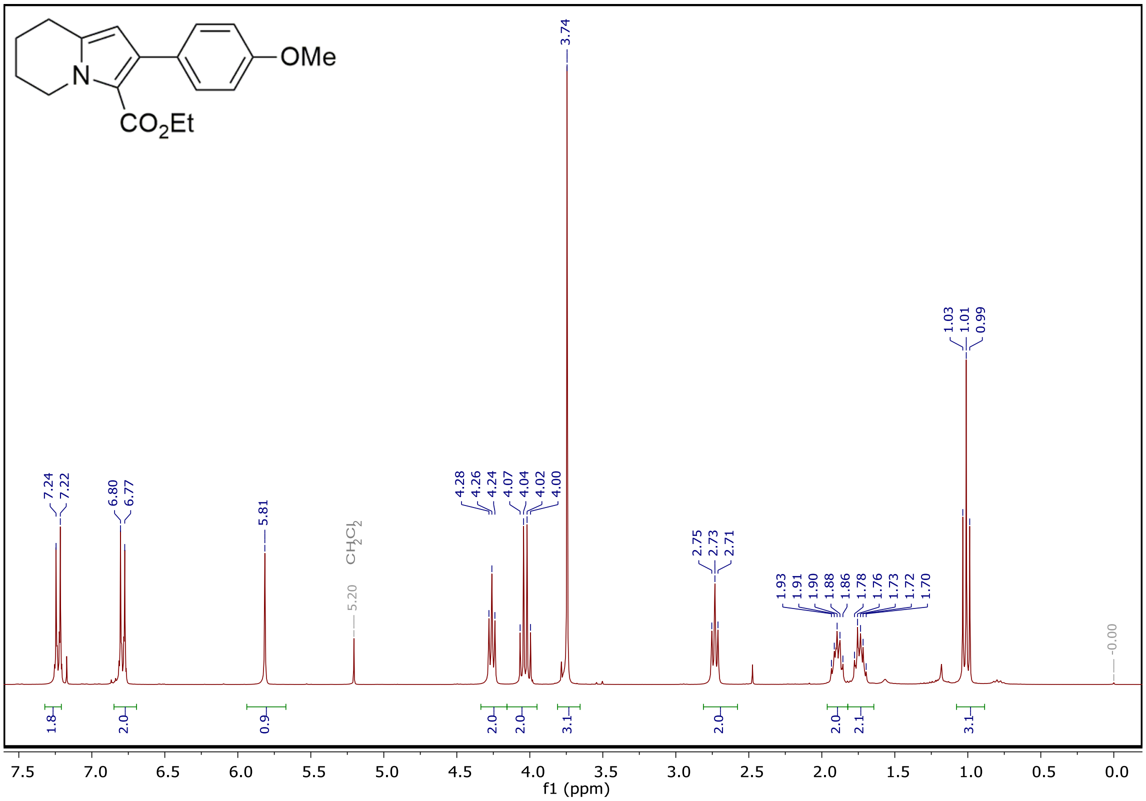

^{13}C NMR spectrum of ethyl 2-(4-methoxyphenyl)-5,6,7,8-tetrahydroindolizine-3-carboxylate (26b) (75 MHz, $CDCl_3$)

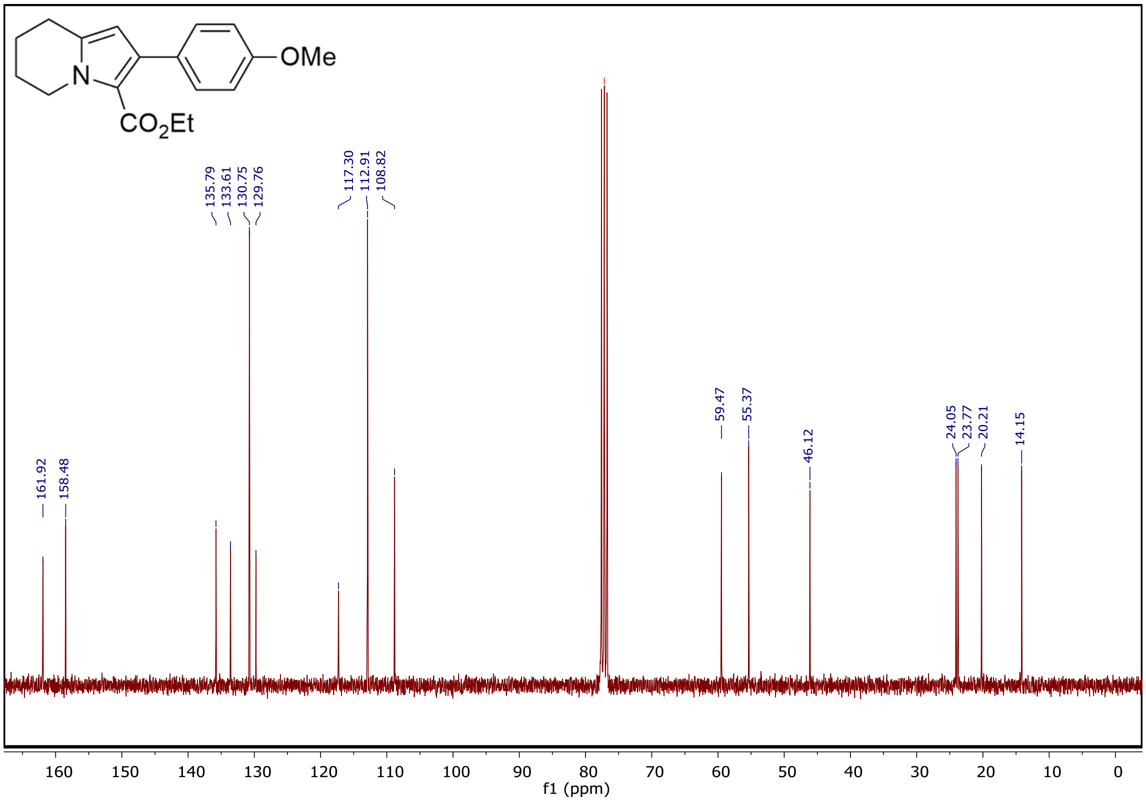

^{1}H NMR spectrum of ethyl 2-(4-nitrophenyl)-5,6,7,8-tetrahydroindolizine-3-carboxylate (26c) (300 MHz, $CDCl_3$)

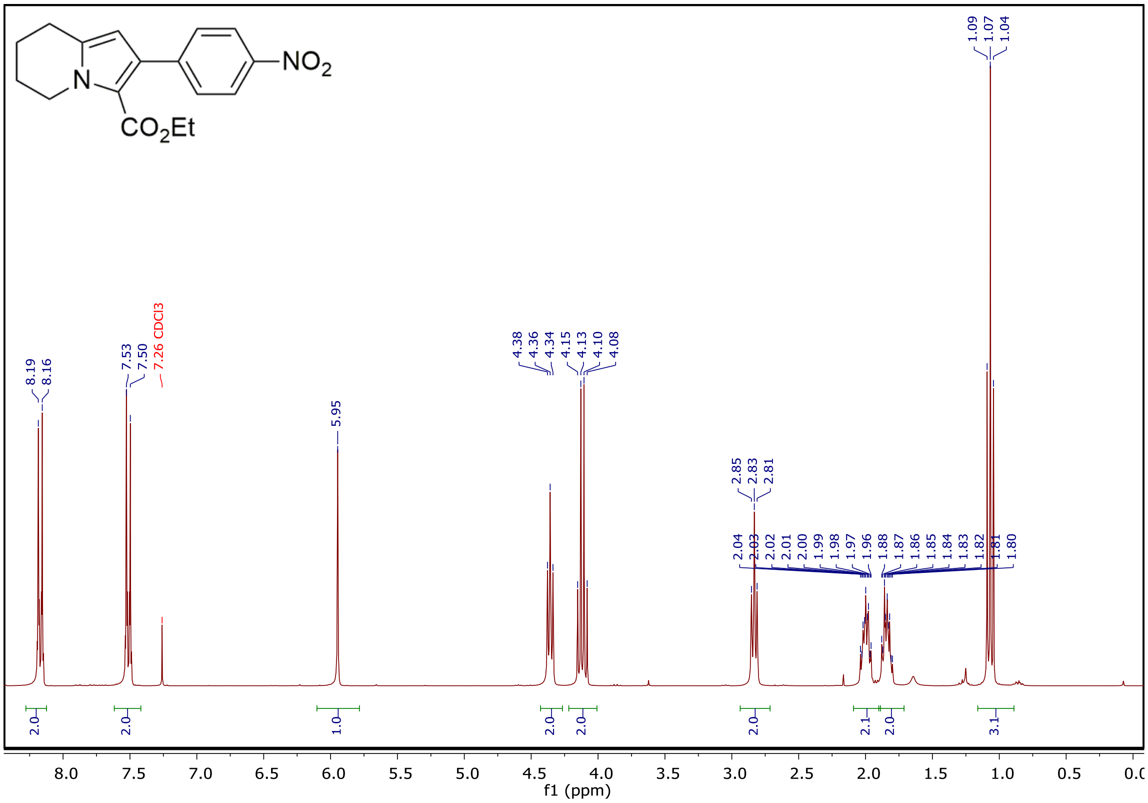

^{13}C NMR spectrum of ethyl 2-(4-nitrophenyl)-5,6,7,8-tetrahydroindolizine-3-carboxylate (26c) (75 MHz, $CDCl_3$)

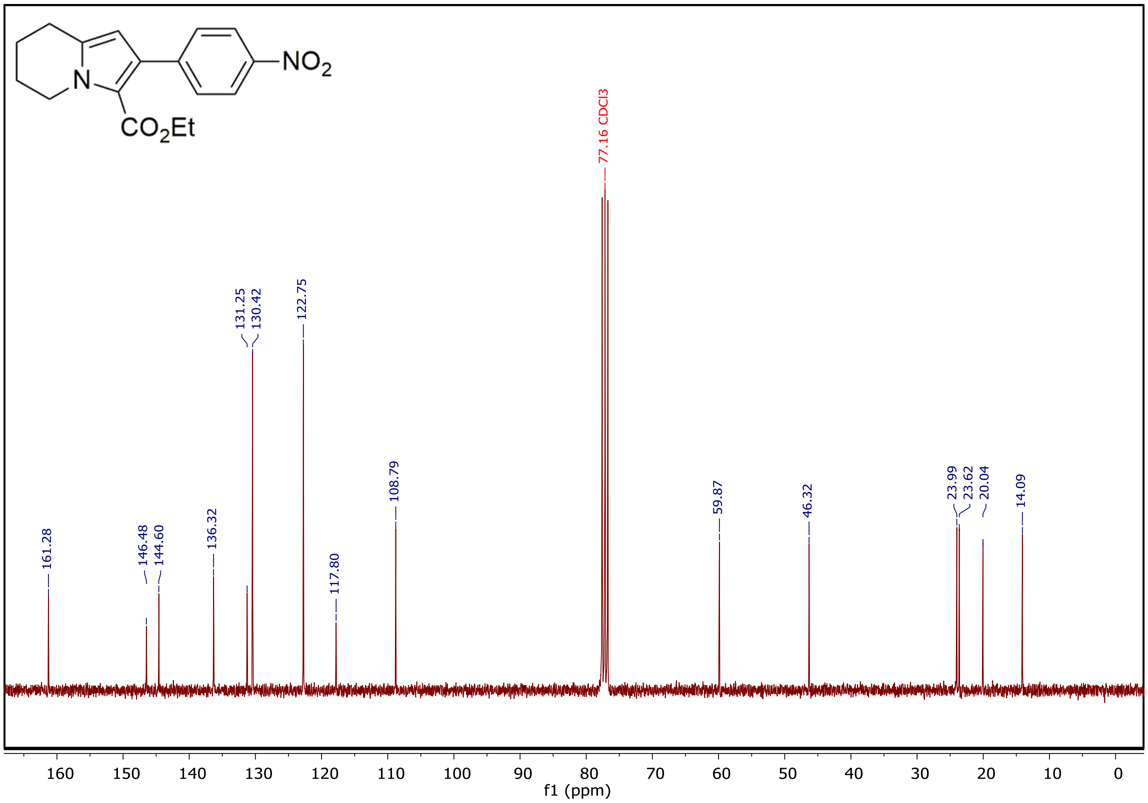

^{1}H NMR spectrum of ethyl 7-bromo-6-phenyl-2,3-dihydro-1*H*-pyrrolizine-5-carboxylate (28) (300 MHz, $CDCl_3$)

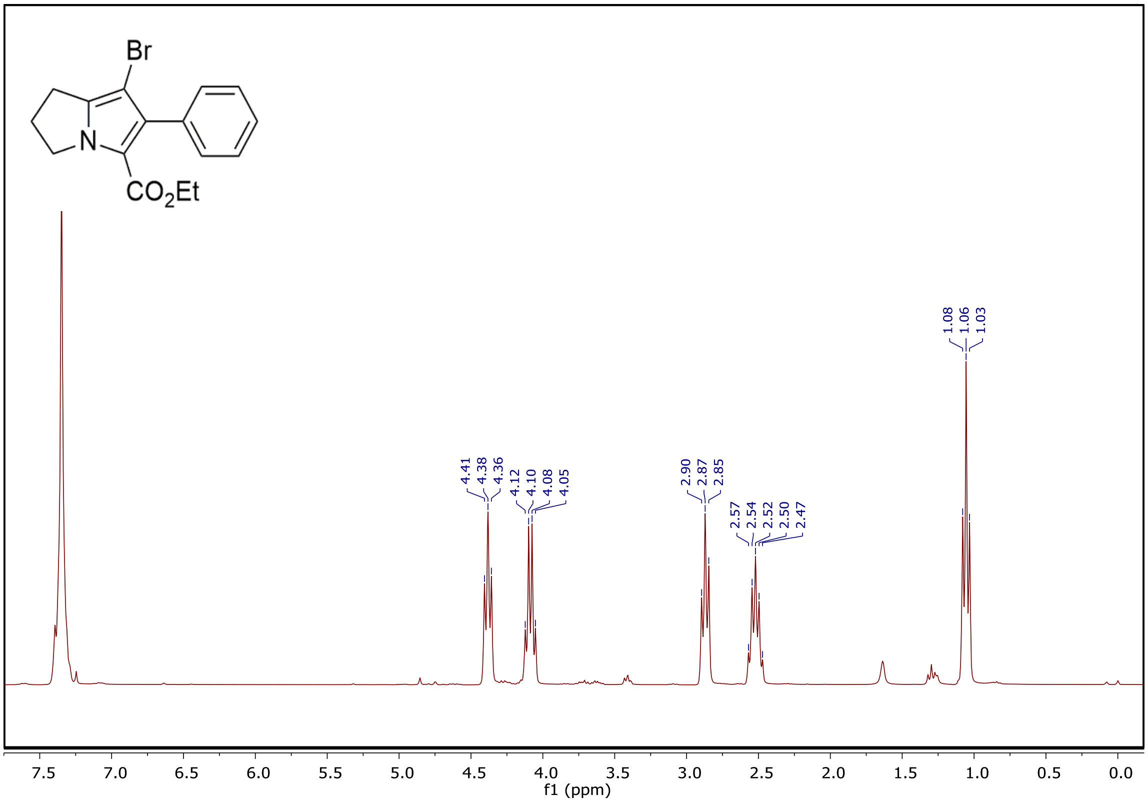

^{13}C NMR spectrum of ethyl 7-bromo-6-phenyl-2,3-dihydro-1*H*-pyrrolizine-5-carboxylate (28) (75 MHz, $CDCl_3$)

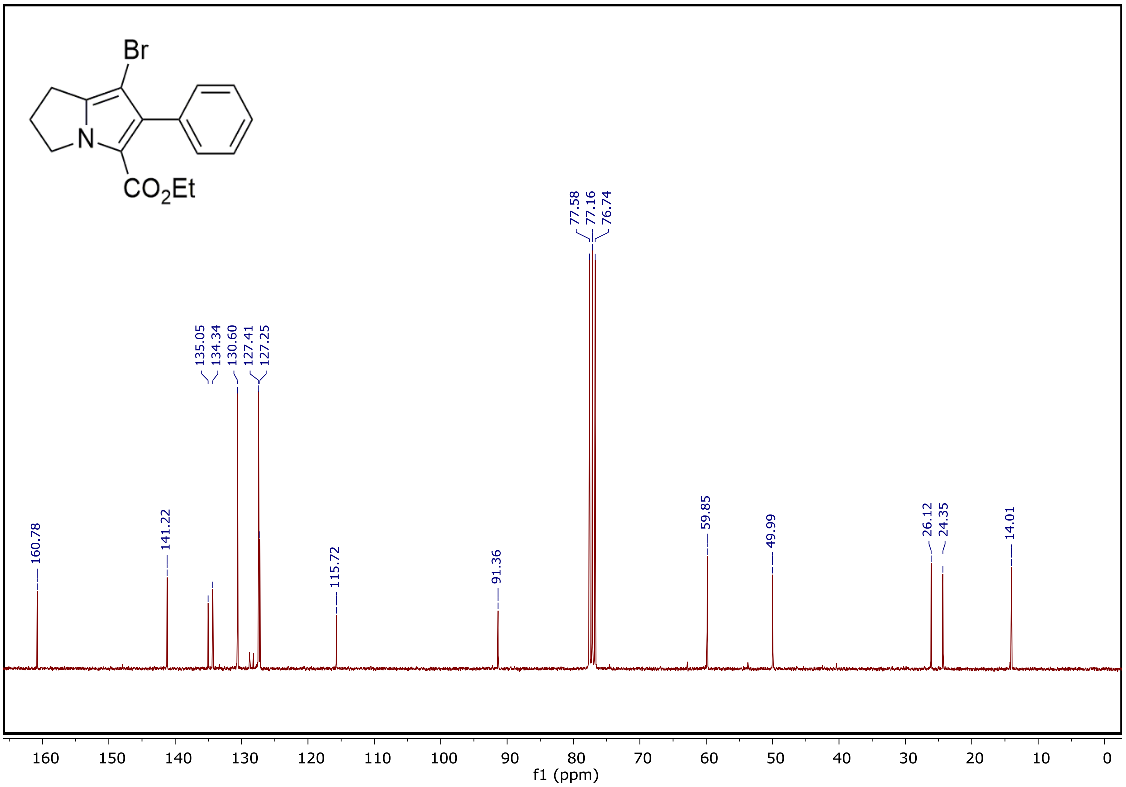

^{1}H NMR spectrum of ethyl 6,7-diphenyl-2,3-dihydro-1*H*-pyrrolizine-5-carboxylate (29) (300 MHz, $CDCl_3$)

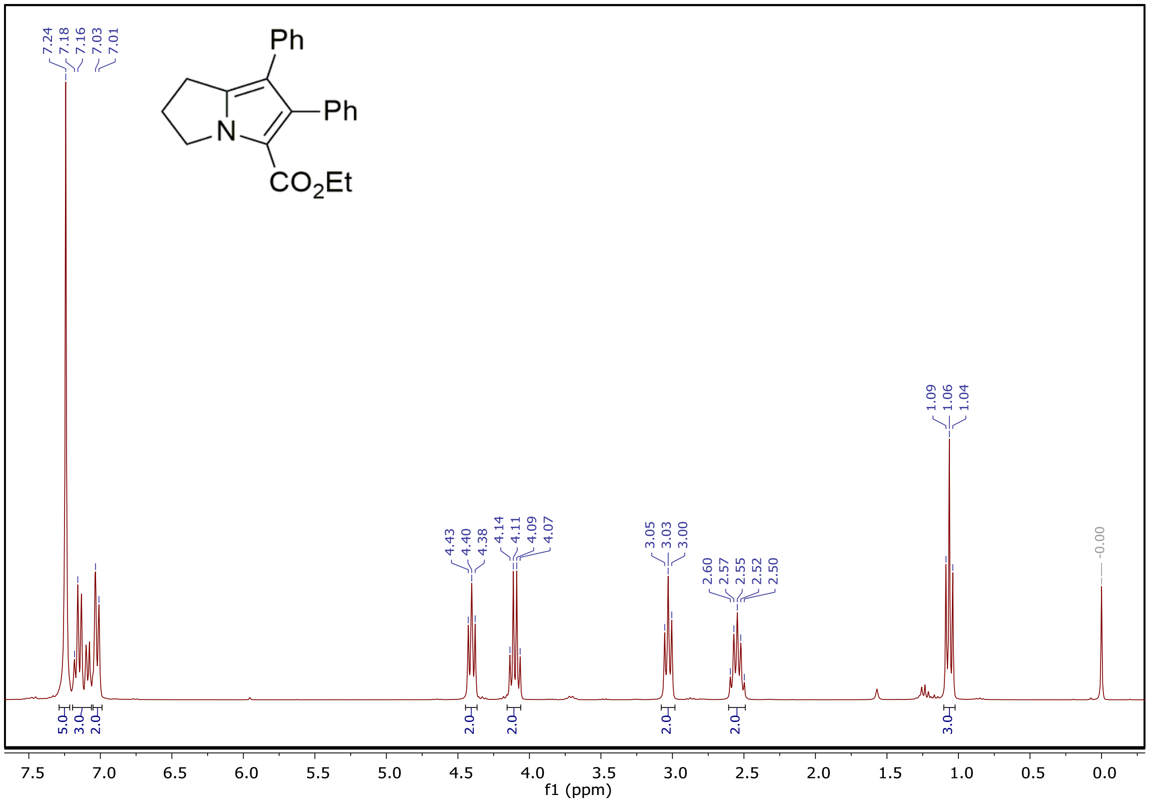

^{13}C NMR spectrum of ethyl 6,7-diphenyl-2,3-dihydro-1*H*-pyrrolizine-5-carboxylate (29) (75 MHz, $CDCl_3$)

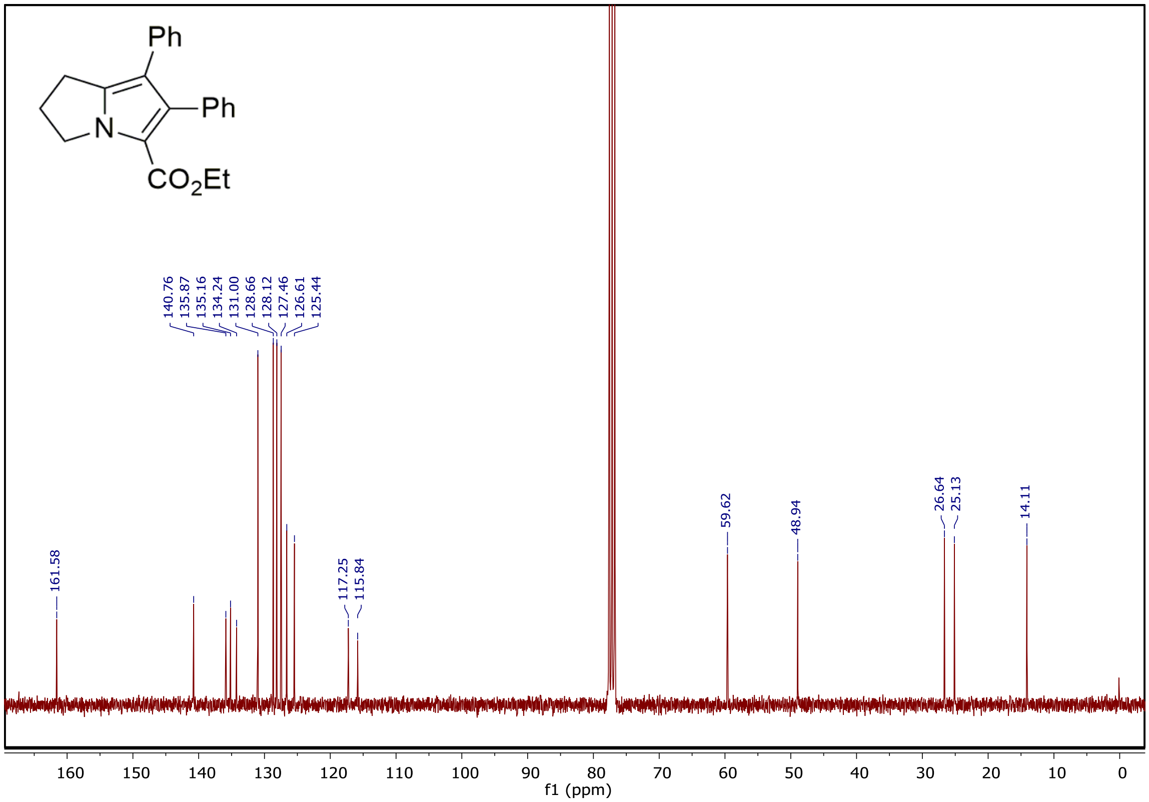

Supplement: File 1 — Experimental details for the synthesis and characterization of all compounds, and copies of 1H NMR and 13C NMR spectra. [file Beilstein_J_Org_Chem-17-2543-s001.pdf]
